# Supplementary material for: HDAC6 deacetylates ENKD1 to regulate mitotic spindle behavior and corneal epithelial homeostasis
Source: EMBO Rep. 2025 Mar 28;26(10):2597–621. doi: 10.1038/s44319-025-00438-0 (PMC12116779; doi:10.1038/s44319-025-00438-0)
Supplement: Supplementary file 1 — Appendix [file 44319_2025_438_MOESM1_ESM.pdf]

Table of Contents

Appendix Figure S1	Knockdown of HDAC6 does not affect spindle orientation or positioning.	Page 2-3
Appendix Figure S2	Effects of HDAC6 mutants on its deacetylase activity.	Page 4
Appendix Figure S3	The centrosomal localization of GFP-HDAC6 is weakened in metaphase.	Page 5
Appendix Figure S4	ENKD1 is crucial for spindle orientation in HCE-2 cells.	Page 6

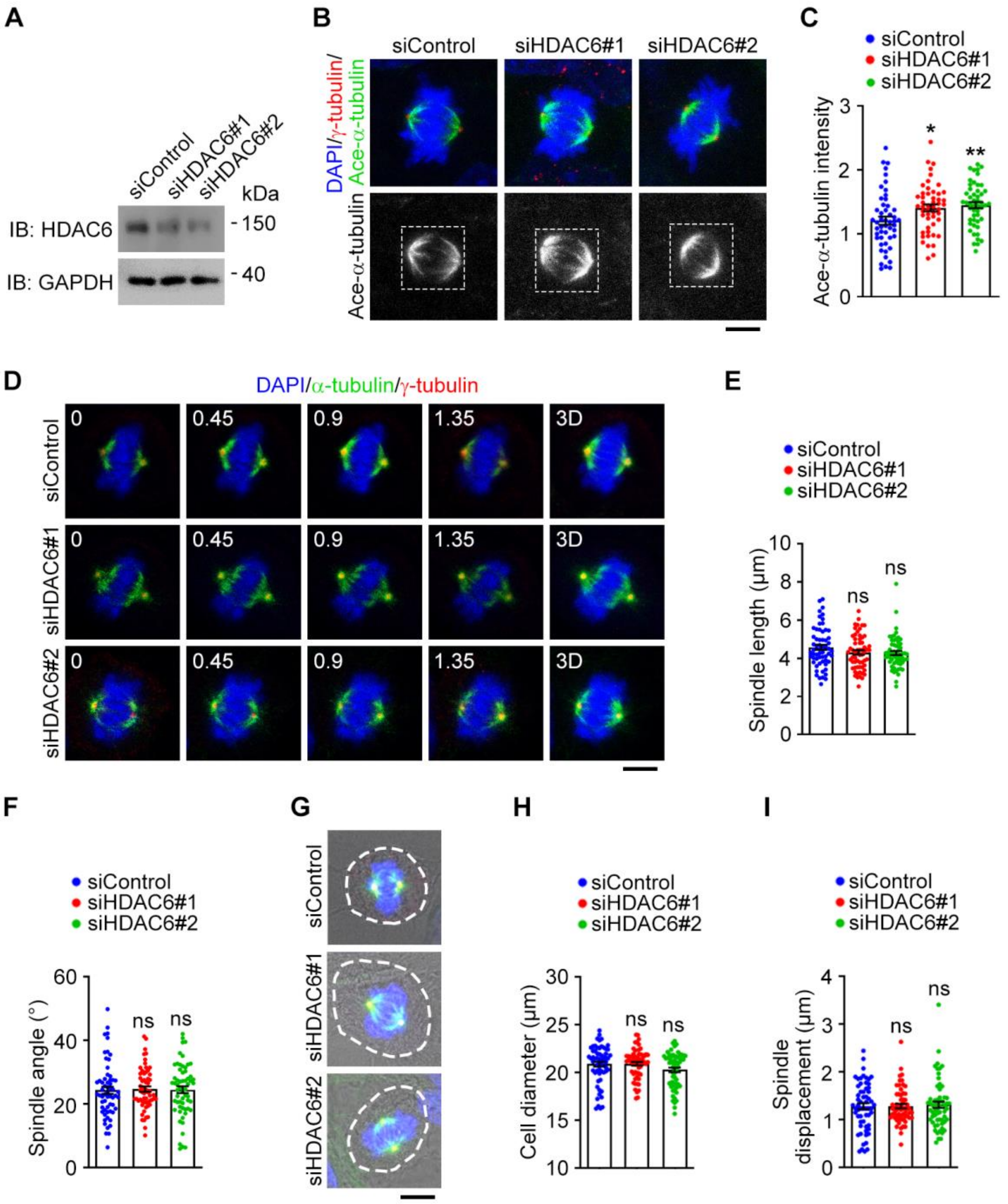

Appendix Figure S1. Knockdown of HDAC6 does not affect spindle orientation or positioning.

(A) Immunoblotting of HDAC6 and GAPDH in HCE-2 cells transfected with HDAC6 or control siRNAs. (**B** and **C**) Immunofluorescence images (B) and quantification of the intensity of acetylated α-tubulin (C, n = 50 images from three independent experiments) of metaphase HCE-2 cells transfected with HDAC6 or control siRNAs, and stained with antibodies against acetylated α-tubulin and γ-tubulin and DAPI. The area inside the white box was used for quantitative analysis (B). Scale bar, 4 μm. siHDAC6#1, $p = 0.0226$; siHDAC6#2, $p = 0.0044$. (**D**-**F**) Immunofluorescence images (D) and quantifications of spindle length (E, n = 60 images from three independent experiments) and spindle angle (F, n = 60 images from three independent experiments) of metaphase HCE-2 cells transfected with HDAC6 or control siRNAs, and stained with antibodies against α-tubulin and γ-tubulin and DAPI. Scale bar, 4 μm. (**G-I**) Immunofluorescence/bright-field images (G) and quantifications of cell diameter (H, n = 60 images from three independent experiments) and spindle displacement distance (I, n = 60 images from three independent experiments) of metaphase HCE-2 cells transfected with HDAC6 or control siRNAs, and stained with antibodies against α-

tubulin and γ-tubulin and DAPI. The white circle indicates the cell boundary (G). Scale bar, 6 μm.

Data are presented as mean ± SEM. Non-parametric one-way ANOVA with post hoc analysis was performed. *$p < 0.05$; **$p < 0.01$; ns, not significant.

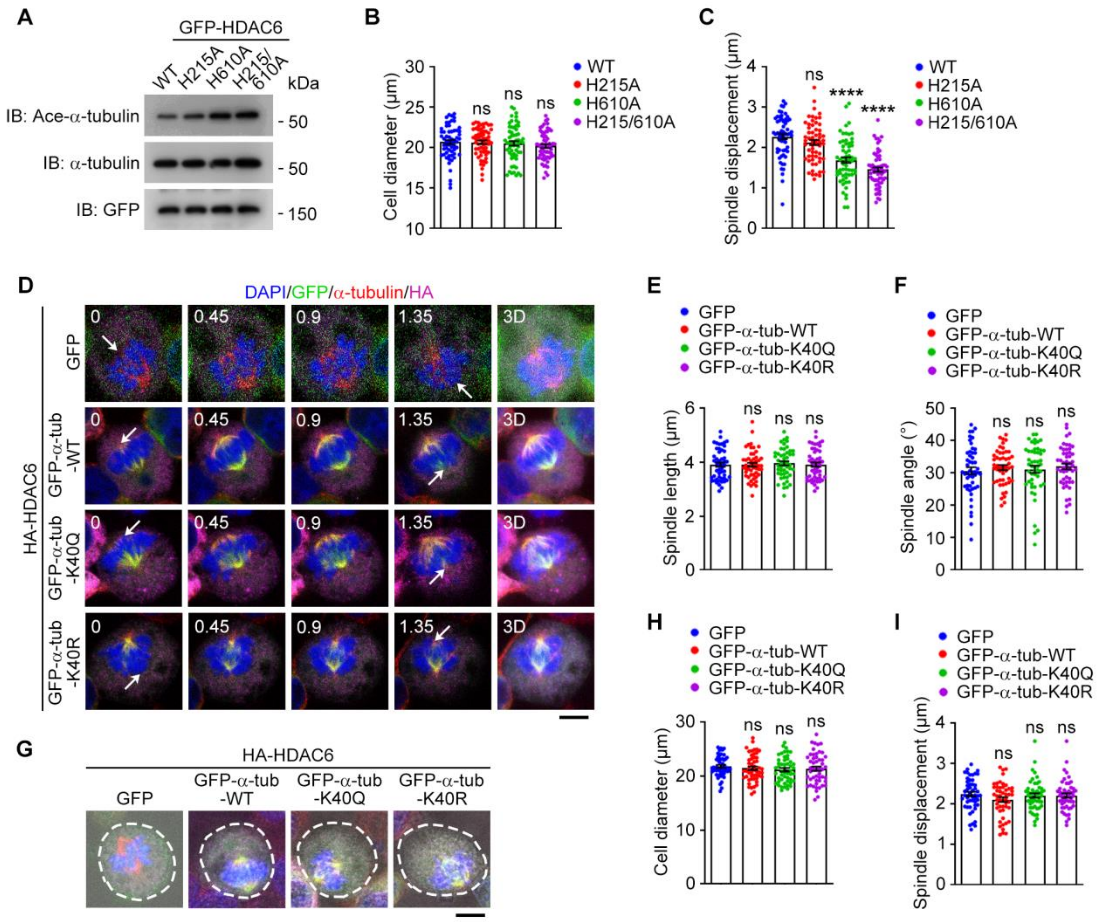

Appendix Figure S2. Effects of HDAC6 mutants on its deacetylase activity.

(**A**) Immunoblot analysis of acetylated α-tubulin, α-tubulin, and GFP in HCE-2 cells transfected with GFP-HDAC6 wild-type, H215A, H610A, or H215/610A. (**B** and **C**) Quantification of cell diameter (B, n = 60 images from three independent experiments) and spindle displacement distance (C, n = 60 images from three independent experiments) of metaphase HCE-2 cells transfected with GFP-HDAC6 wild-type, H215A, H610A, or H215/610A. H215A, $p = 0.2492$; H610A, $p < 0.0001$; H215/610A, $p < 0.0001$. (**D-F**) Immunofluorescence images (D) and quantification of spindle length (E, n = 50 images from three independent experiments) and spindle angle (F, n = 50 images from three independent experiments) of metaphase HCE-2 cells transfected with HA-HDAC6 and GFP, GFP-α-tubulin wild-type, K40Q, or K40R, and stained with antibodies against α-tubulin and HA and DAPI. The white arrow refers to the spindle pole that does not appear on the corresponding focal plane (D). Scale bar, 6 μm. (**G-I**) Immunofluorescence/bright-field images (G) and quantification of cell diameter (H, n = 50 images from three independent experiments) and spindle displacement distance (I, n = 50 images from three independent experiments) of metaphase HCE-2 cells transfected with HA-HDAC6 and GFP, GFP-α-tubulin wild-type, K40Q, or K40R, and stained with antibodies against α-tubulin and HA and DAPI. The white circle indicates the cell boundary (G). Scale bar, 6 μm.

Data are presented as mean ± SEM. Non-parametric one-way ANOVA with post hoc analysis was performed. ****$p < 0.0001$; ns, not significant.

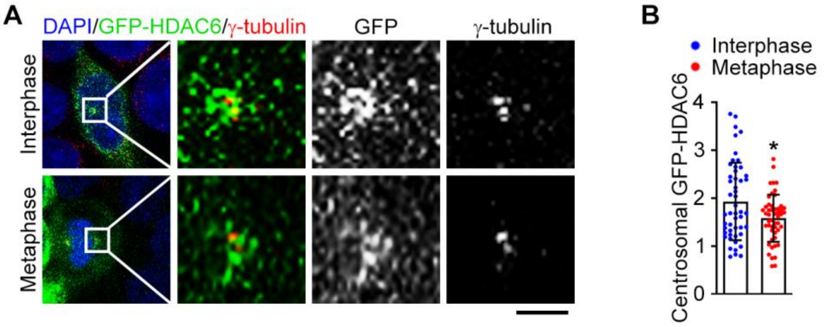

Appendix Figure S3. The centrosomal localization of GFP-HDAC6 is weakened in metaphase.

(**A** and **B**) Immunofluorescence images (A) and quantification of GFP-HDAC6 centrosomal intensity (B, n = 50 cells from three independent experiments) for interphase and metaphase HCE-2 cells transfected with GFP-HDAC6 and stained with antibody against γ-tubulin and DAPI. Scale bar, 2 μm. $p = 0.0103$.

Data are presented as mean ± SEM. Unpaired two-tailed Student's *t*-test was performed. *$p < 0.05$.

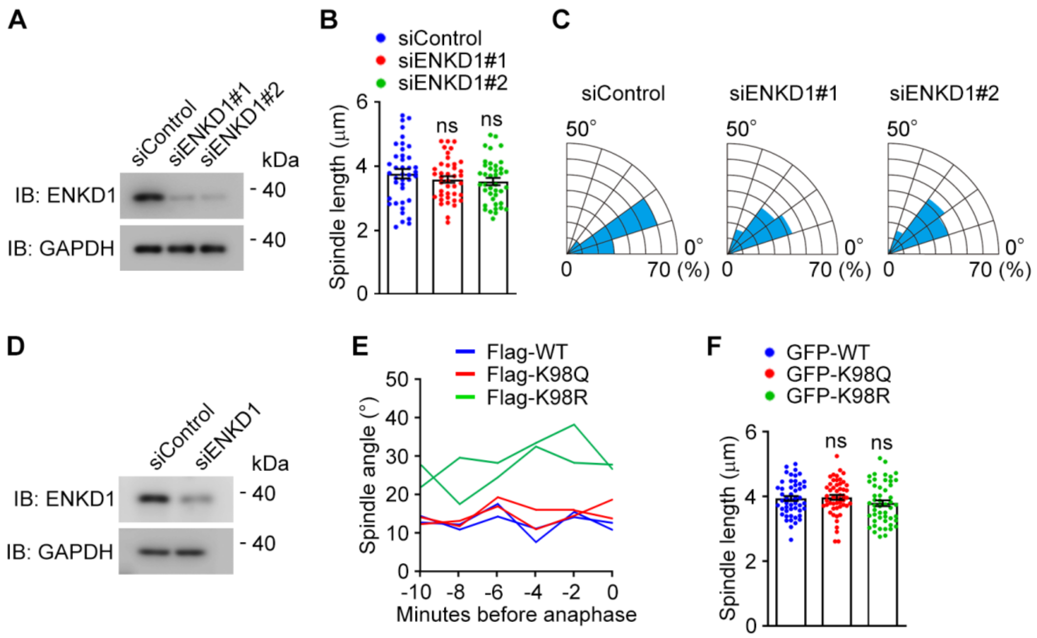

Appendix Figure S4. ENKD1 is crucial for spindle orientation in HCE-2 cells. **(A)** Immunoblot analysis of ENKD1 and GAPDH in HCE-2 cells treated with control or ENKD1 siRNAs. (**B**) Quantification of spindle length (n = 40 images from three independent experiments) in metaphase HCE-2 cells treated with control or ENKD1 siRNAs. (**C**) Spindle angle distribution (n = 40 images from three independent experiments) in metaphase HCE-2 cells treated with control or ENKD1 siRNAs. (**D**) Immunoblot analysis of ENKD1 and GAPDH in HeLa cells treated with control or ENKD1 siRNAs. (**E**) Quantification of spindle angles in mitotic HeLa cells transfected with GFP-histone 2B and ENKD1 siRNAs, together with Flag-ENKD1 wild-type, K98Q, or K98R. Anaphase onset was set at 0 minute. (**F**) Quantification of spindle length (n = 50 images from three independent experiments) in metaphase HCE-2 cells treated with ENKD1 siRNA, together with GFP-ENKD1-wild-type, K98Q, or K98R. Data are presented as mean ± SEM. Non-parametric one-way ANOVA with post hoc analysis was performed. ns, not significant.
