# Supplementary figures and images for: HDAC6 deacetylates ENKD1 to regulate mitotic spindle behavior and corneal epithelial homeostasis (part 1 of 2)
Source: EMBO Rep. 2025 Mar 28;26(10):2597–621. doi: 10.1038/s44319-025-00438-0 (PMC12116779; doi:10.1038/s44319-025-00438-0)

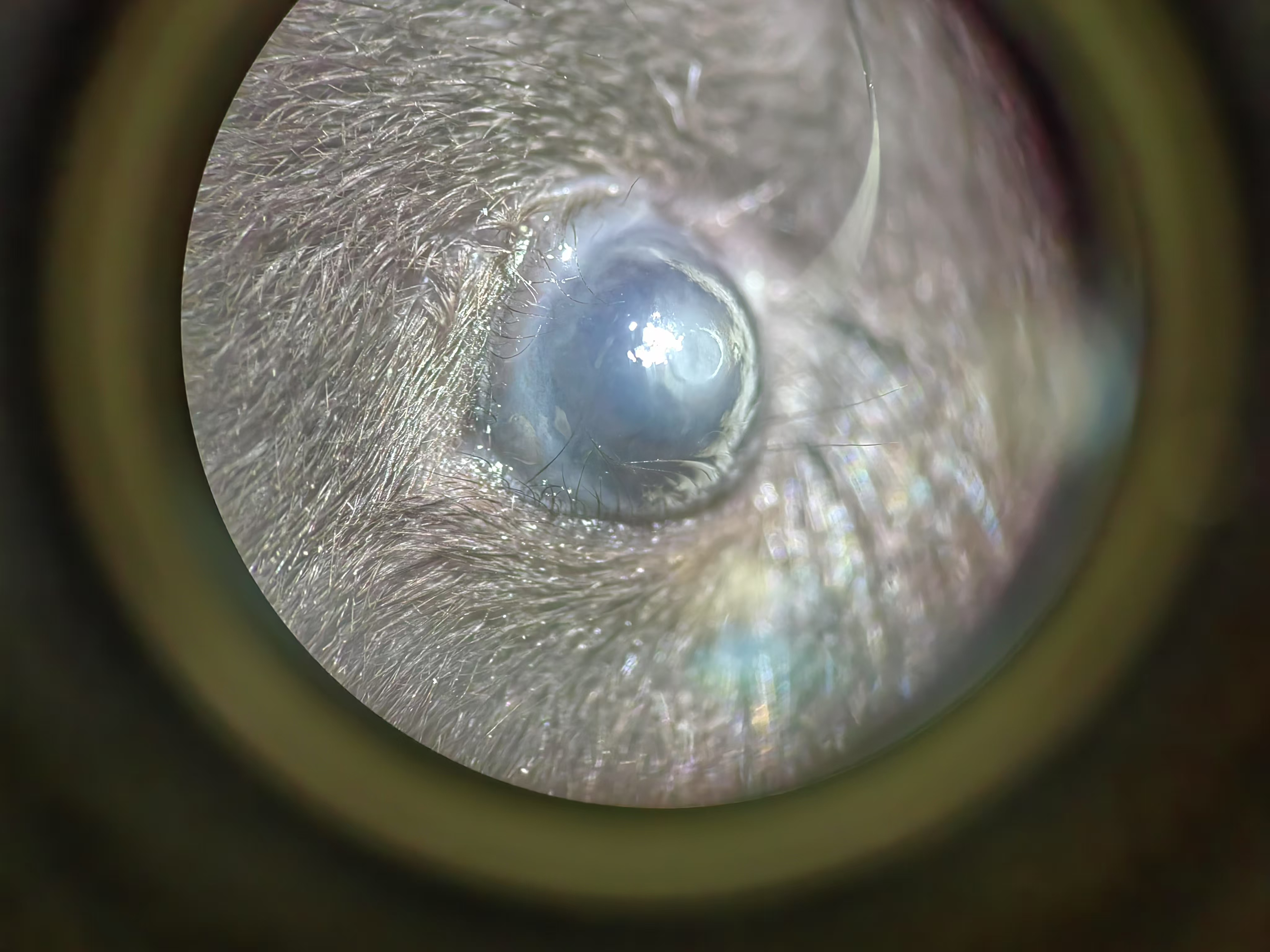

Supplement: Supplementary file 3 — Source data Fig. 1 [file 44319_2025_438_MOESM3_ESM.zip › SD figure 1/Fig. 1D/BK.jpg]

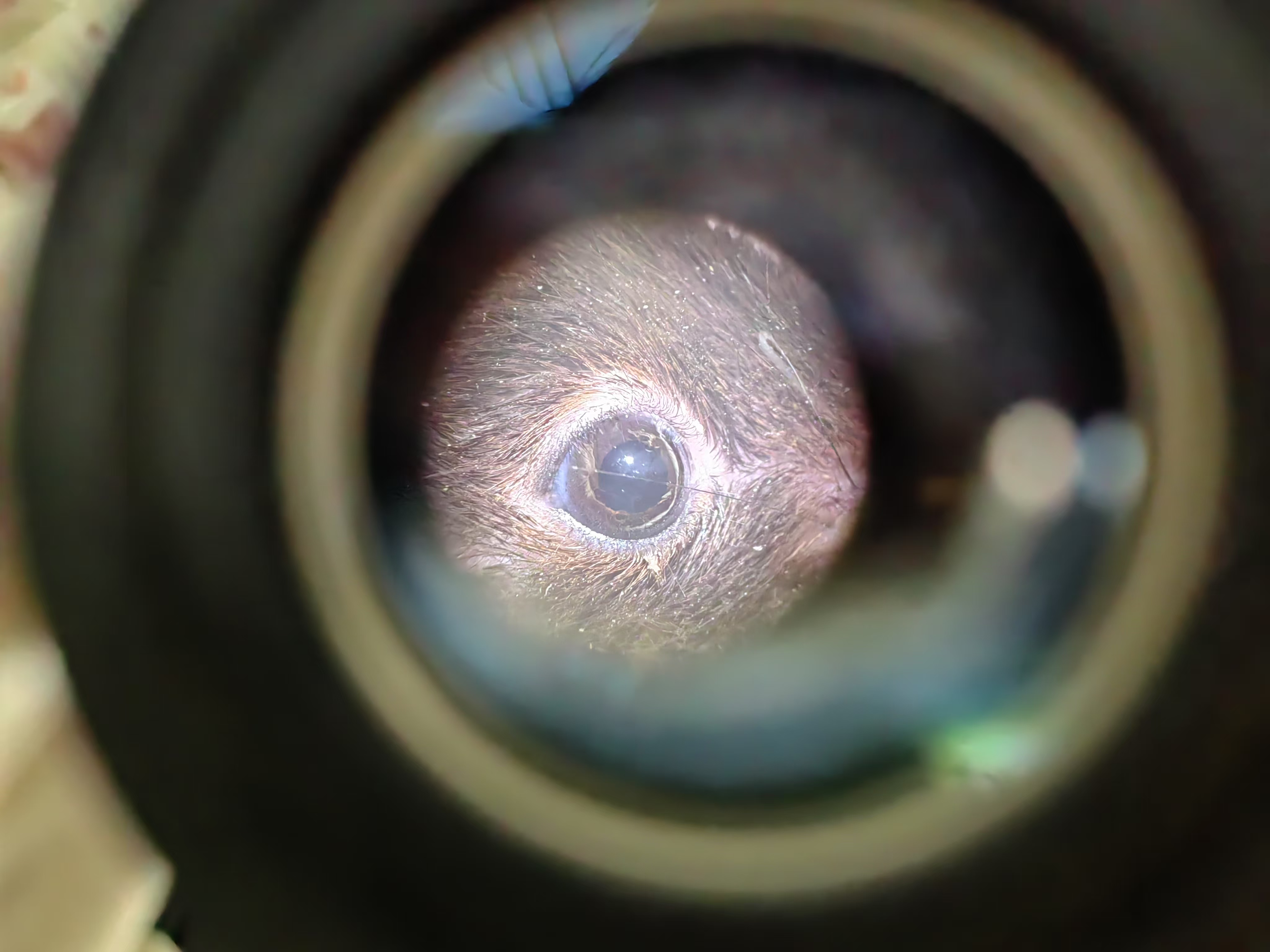

Supplement: Supplementary file 3 — Source data Fig. 1 [file 44319_2025_438_MOESM3_ESM.zip › SD figure 1/Fig. 1D/Healthy.jpg]

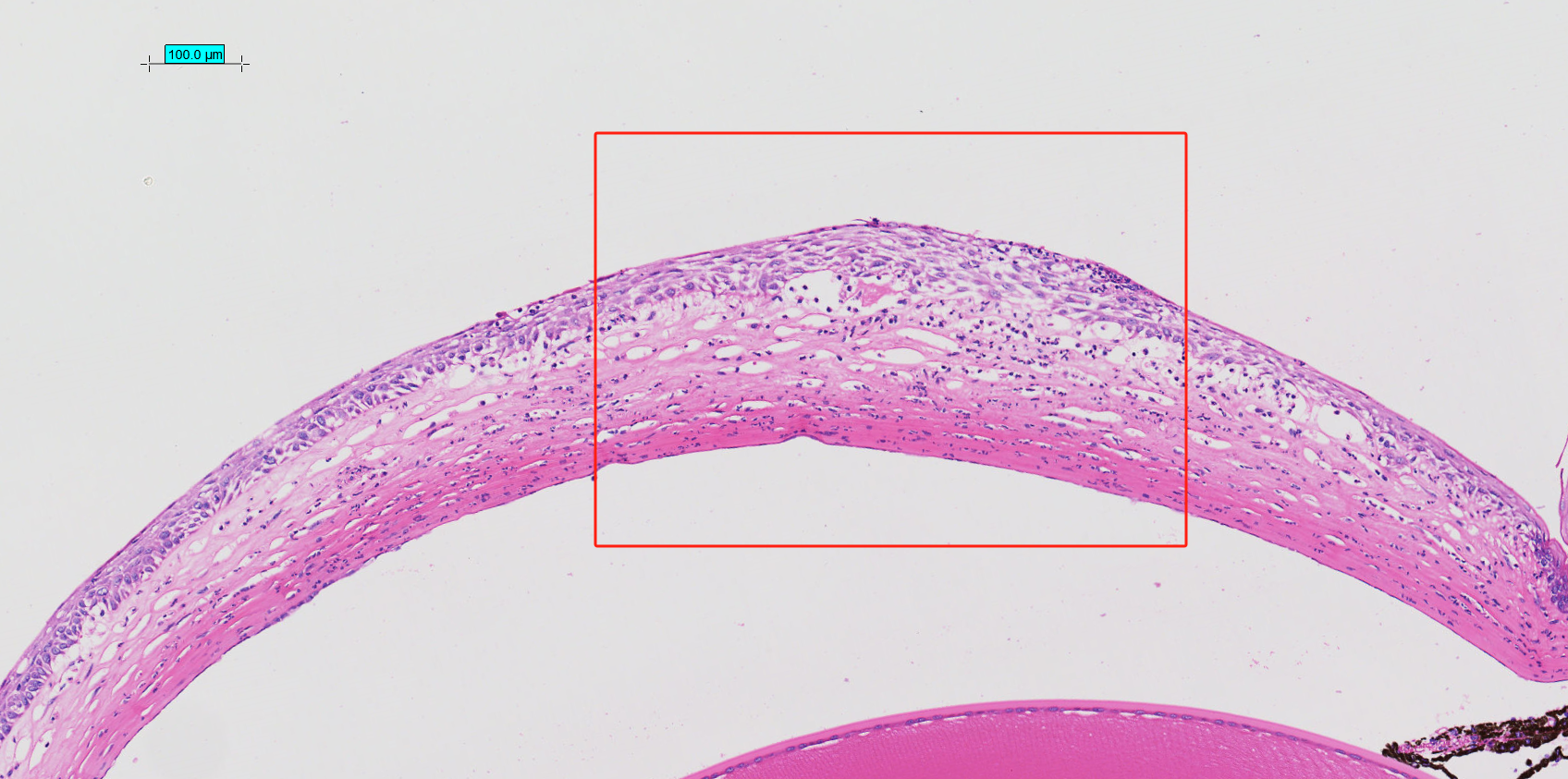

Supplement: Supplementary file 3 — Source data Fig. 1 [file 44319_2025_438_MOESM3_ESM.zip › SD figure 1/Fig. 1E/BK.png]

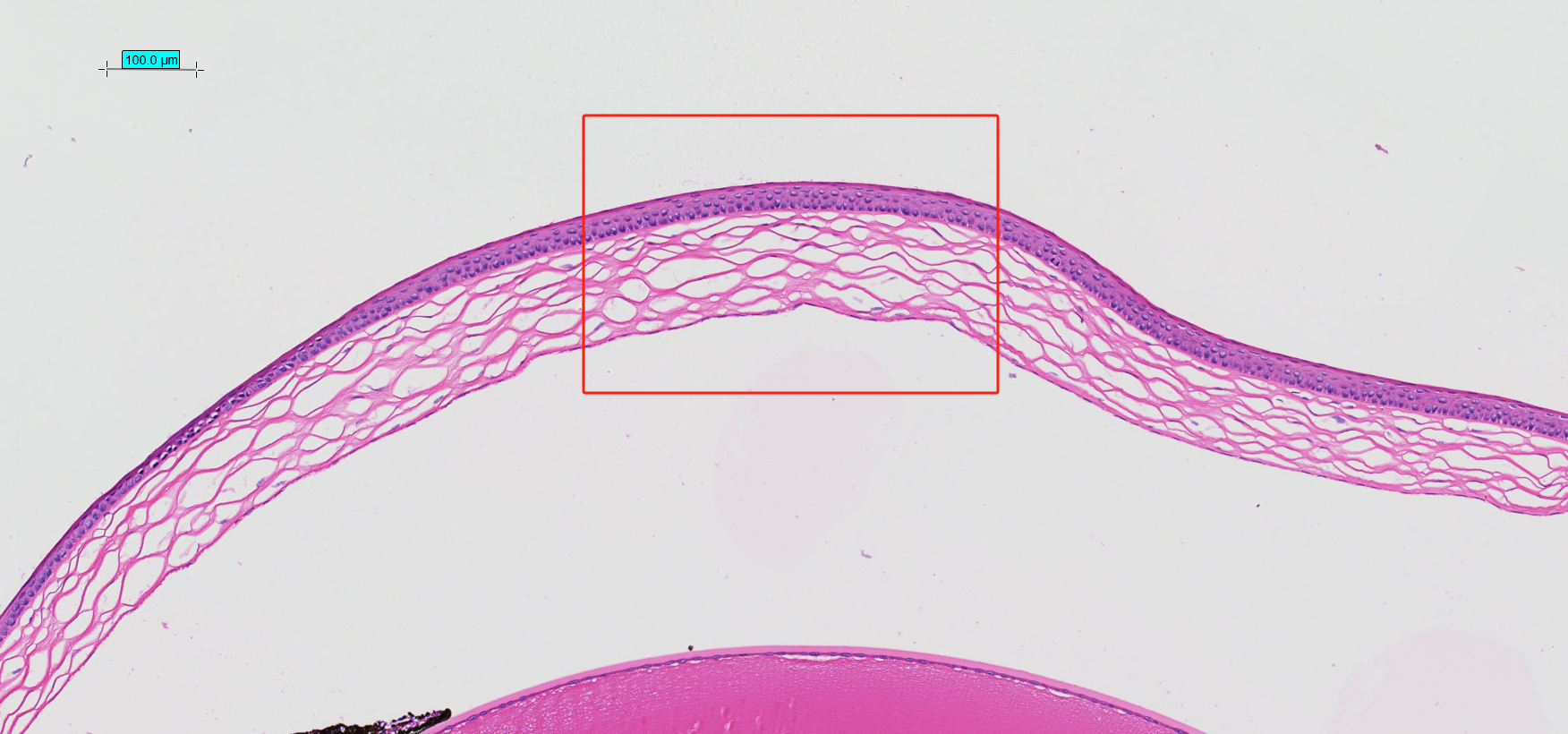

Supplement: Supplementary file 3 — Source data Fig. 1 [file 44319_2025_438_MOESM3_ESM.zip › SD figure 1/Fig. 1E/Healthy.png]

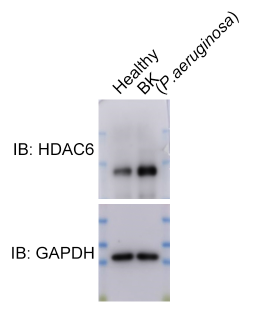

Supplement: Supplementary file 3 — Source data Fig. 1 [file 44319_2025_438_MOESM3_ESM.zip › SD figure 1/Fig. 1F/Fig. 1F.png]

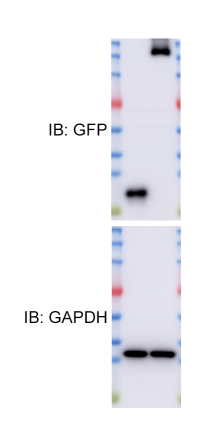

Supplement: Supplementary file 3 — Source data Fig. 1 [file 44319_2025_438_MOESM3_ESM.zip › SD figure 1/Fig. 1H/Fig. 1H.png]

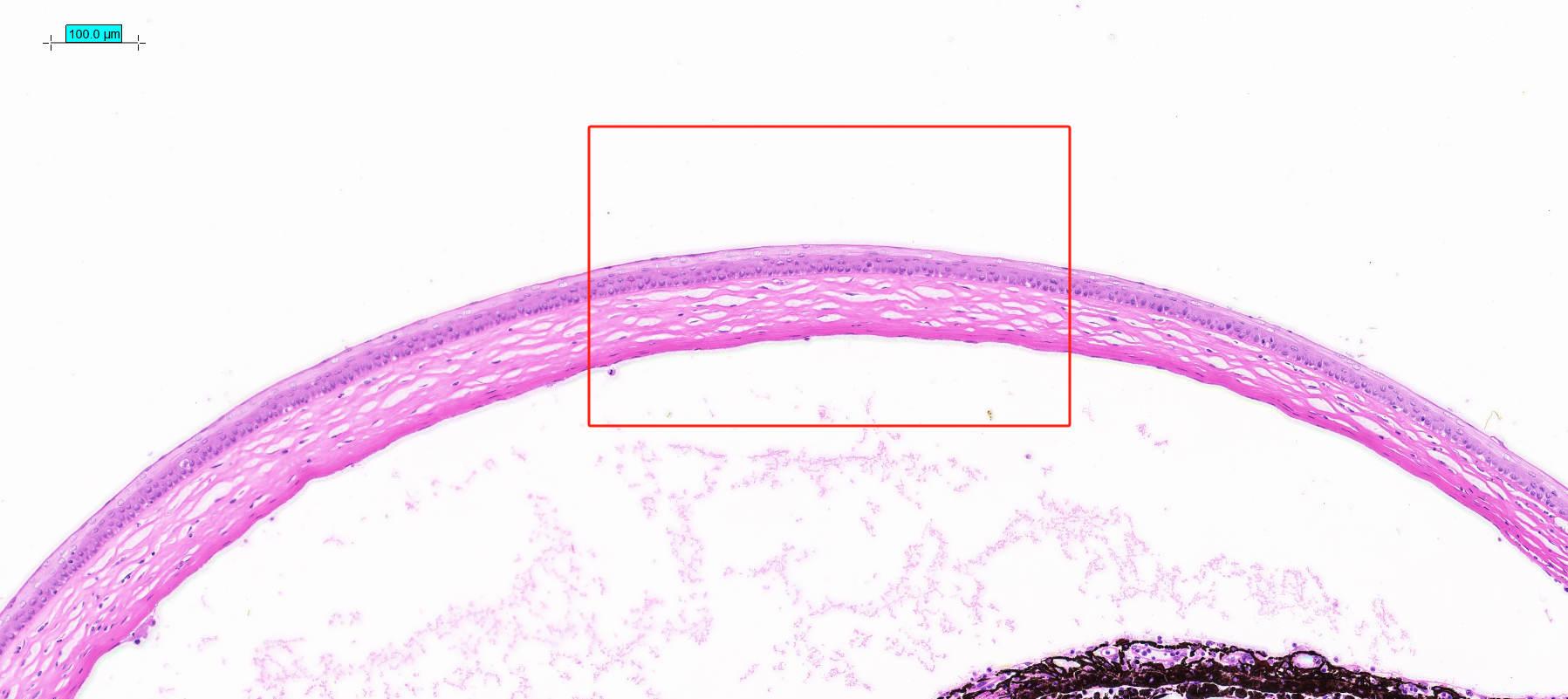

Supplement: Supplementary file 3 — Source data Fig. 1 [file 44319_2025_438_MOESM3_ESM.zip › SD figure 1/Fig. 1I/GFP.png]

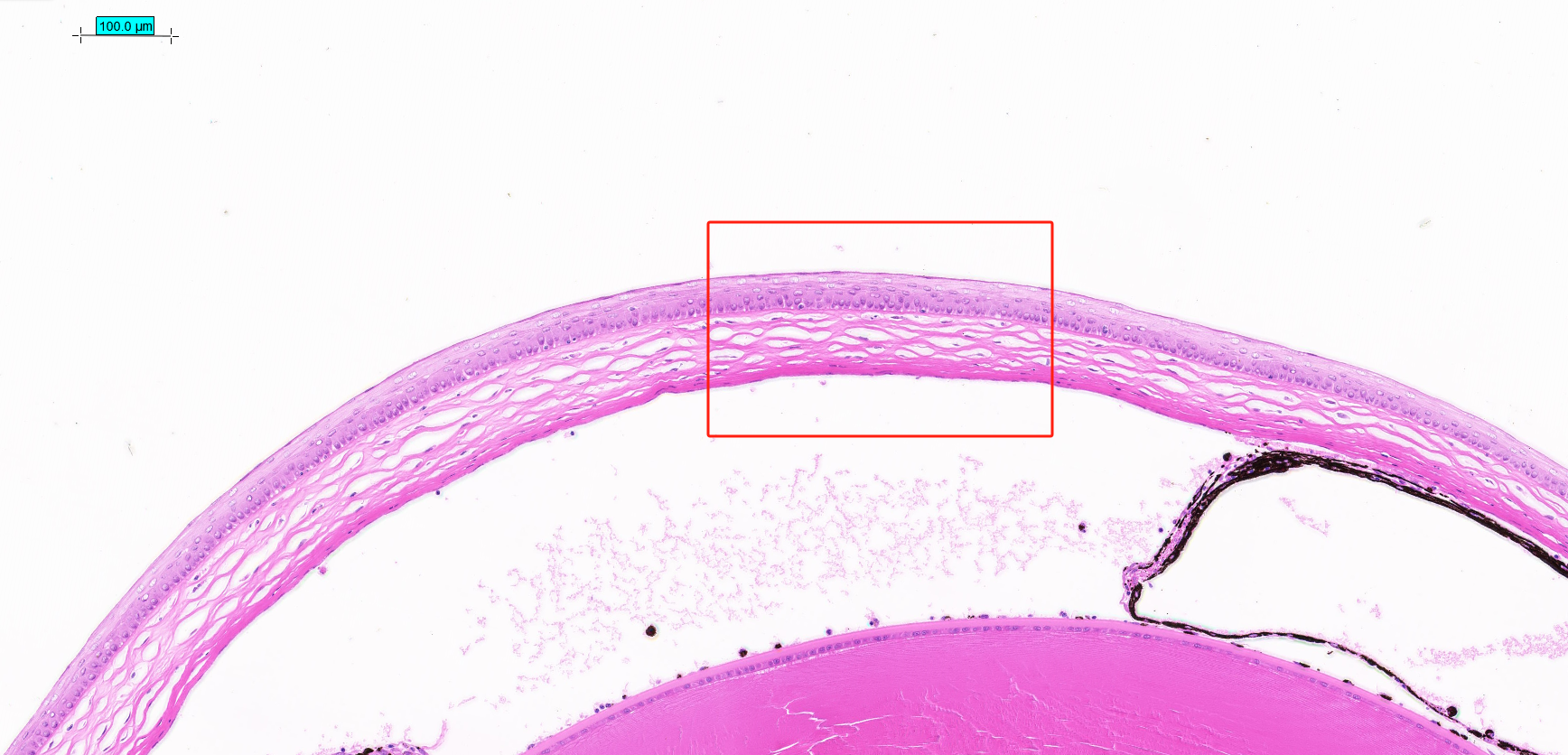

Supplement: Supplementary file 3 — Source data Fig. 1 [file 44319_2025_438_MOESM3_ESM.zip › SD figure 1/Fig. 1I/HDAC6.png]

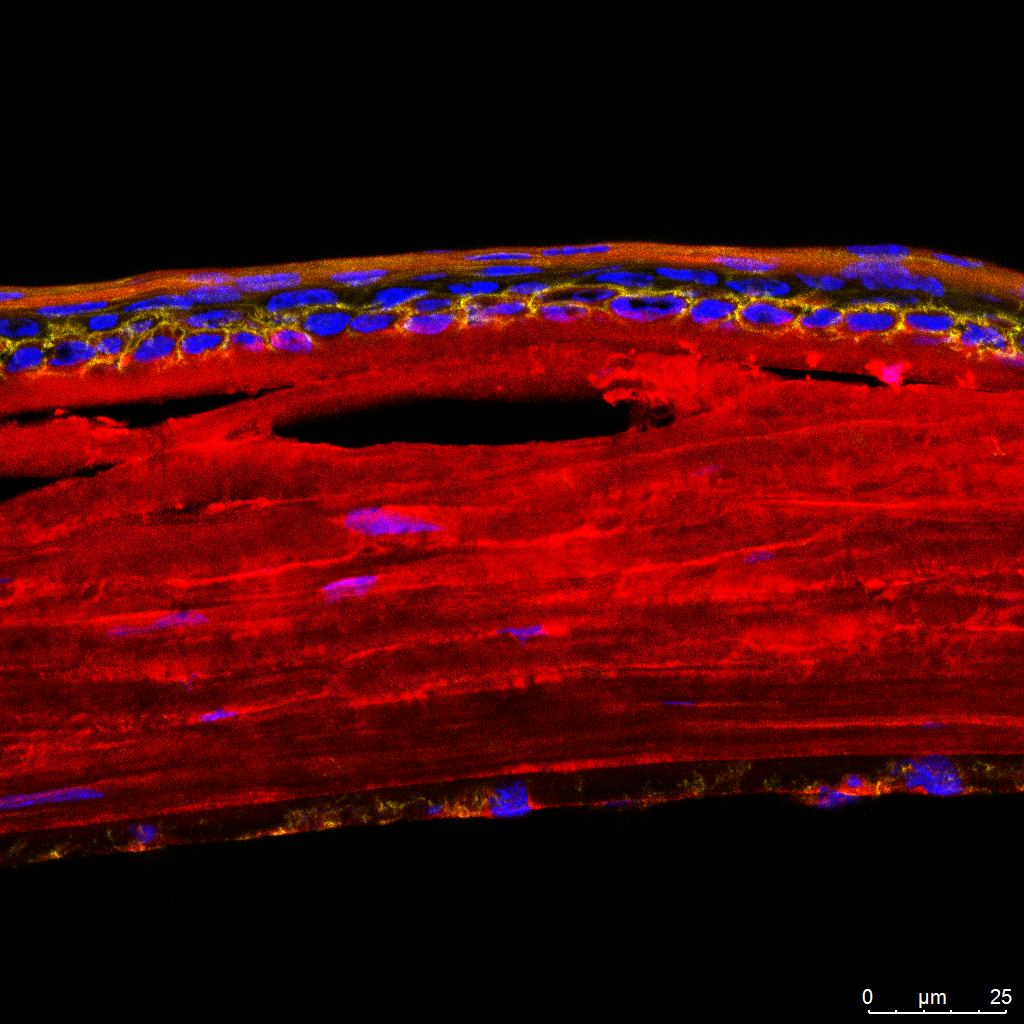

Supplement: Supplementary file 3 — Source data Fig. 1 [file 44319_2025_438_MOESM3_ESM.zip › SD figure 1/Fig. 1K/GFP.tif]

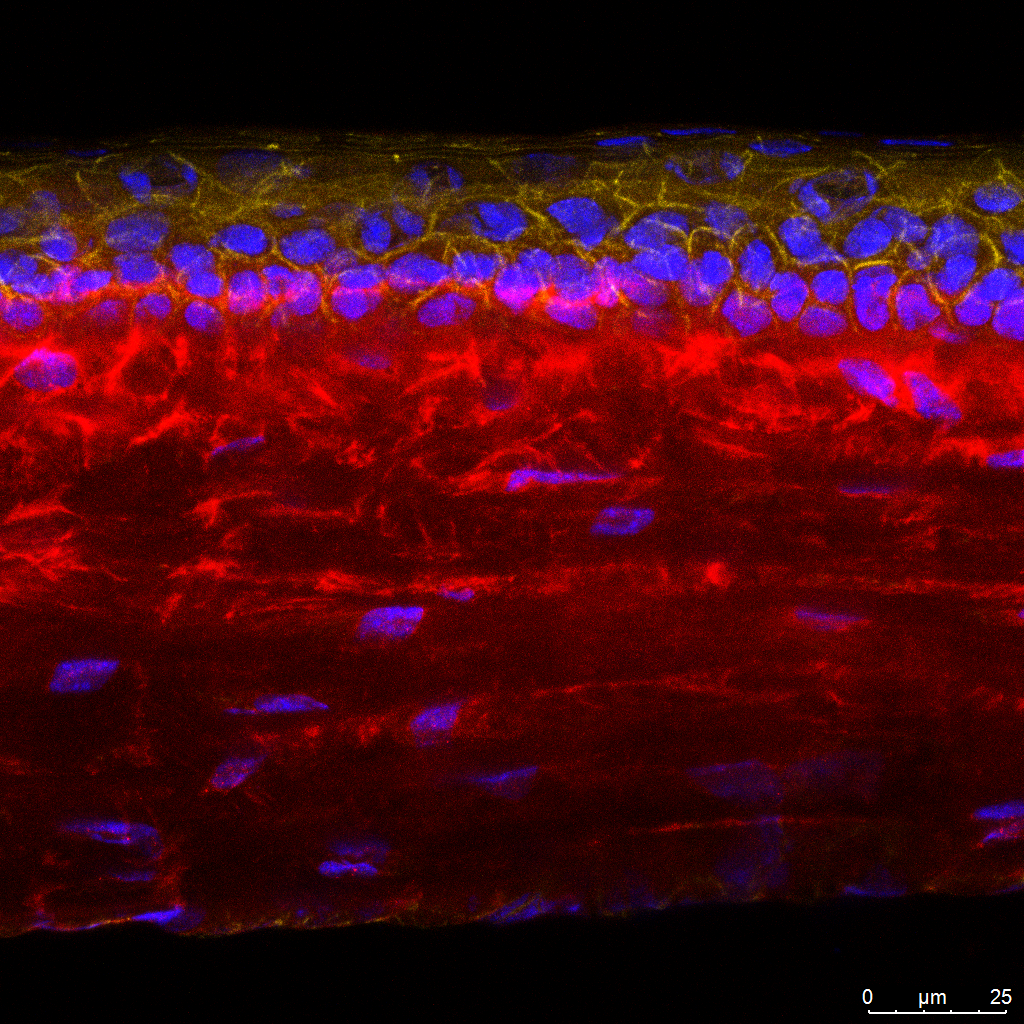

Supplement: Supplementary file 3 — Source data Fig. 1 [file 44319_2025_438_MOESM3_ESM.zip › SD figure 1/Fig. 1K/GFP-HDAC6.tif]

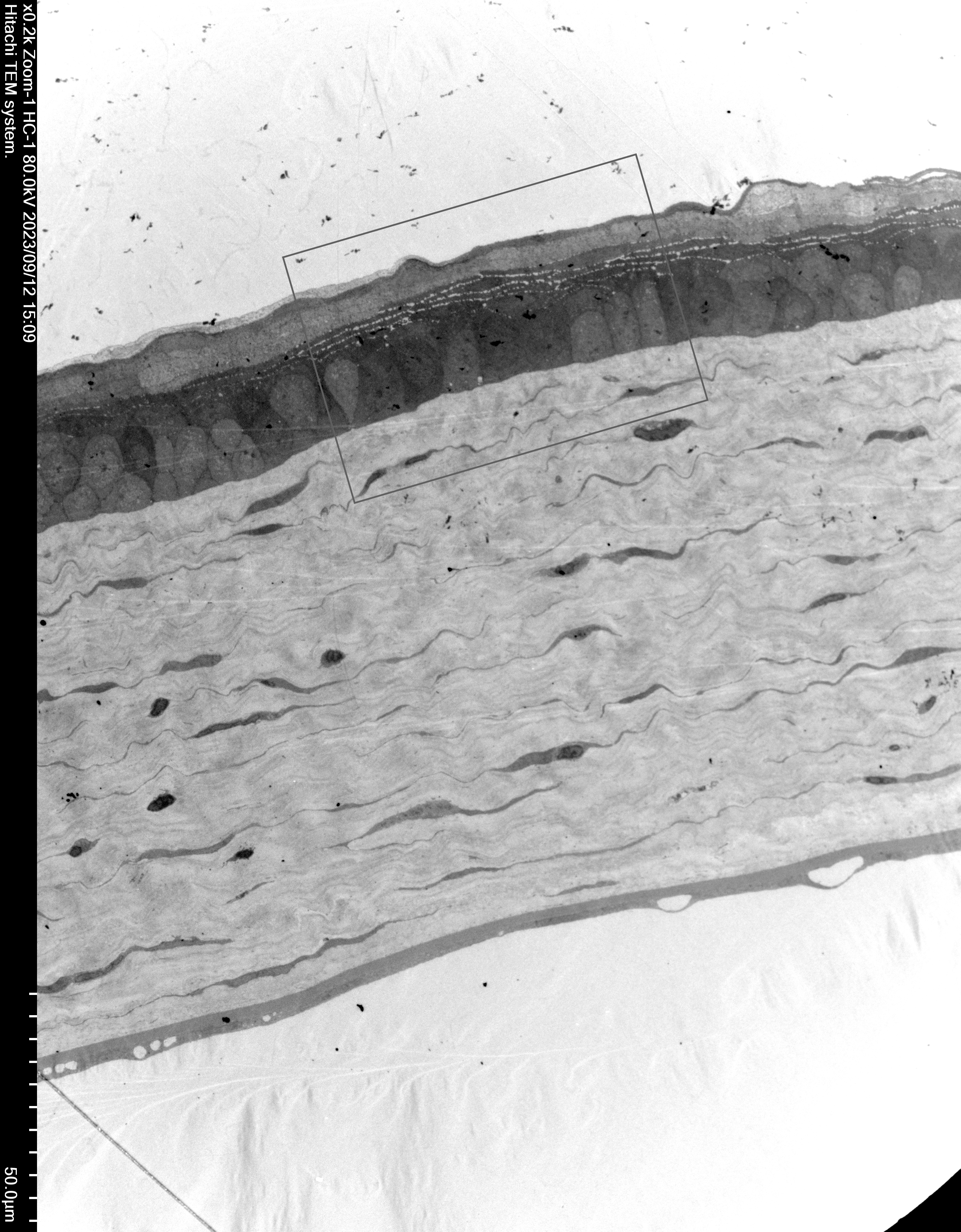

Supplement: Supplementary file 3 — Source data Fig. 1 [file 44319_2025_438_MOESM3_ESM.zip › SD figure 1/Fig. 1M/GFP.tif]

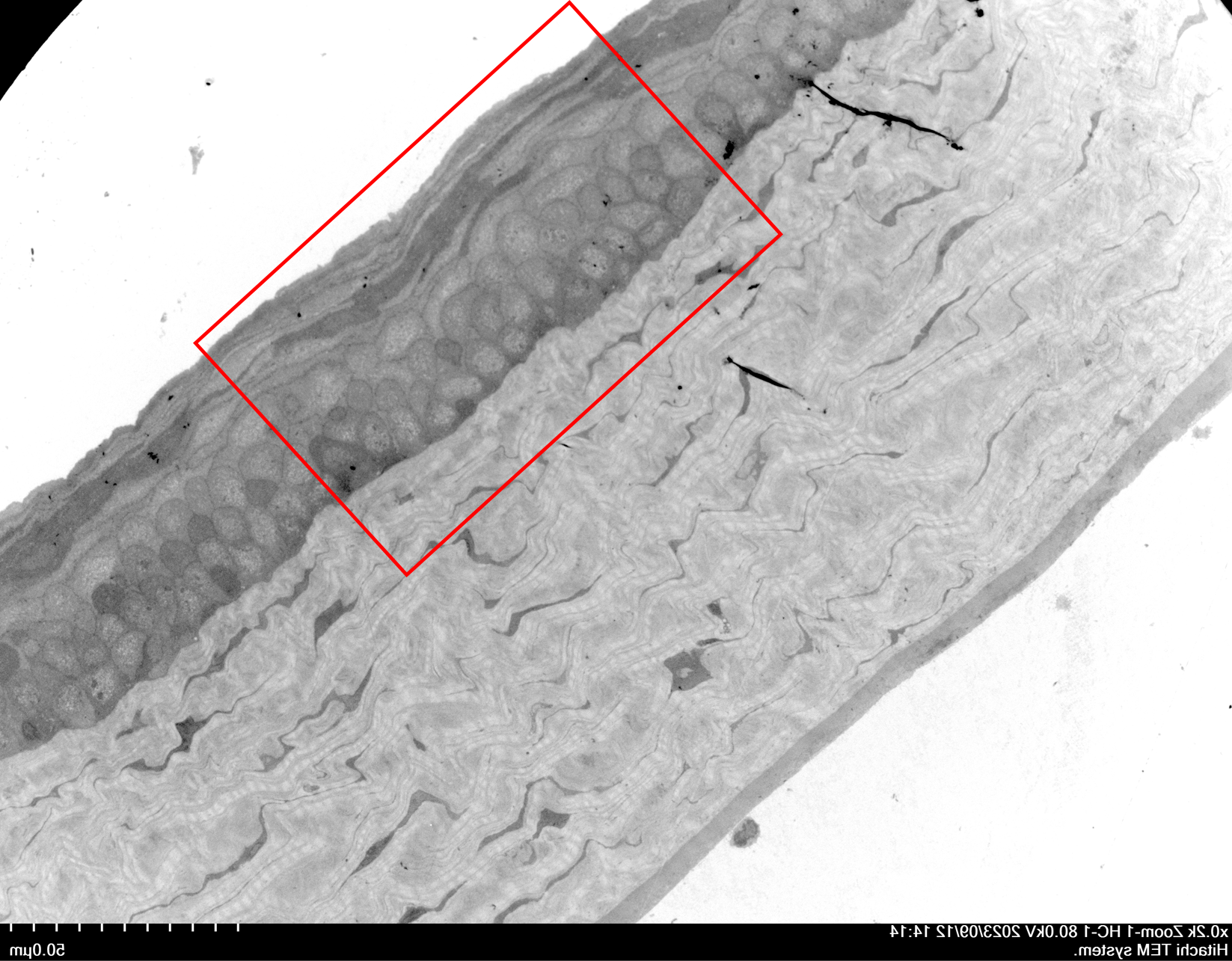

Supplement: Supplementary file 3 — Source data Fig. 1 [file 44319_2025_438_MOESM3_ESM.zip › SD figure 1/Fig. 1M/GFP-HDAC6.tif]

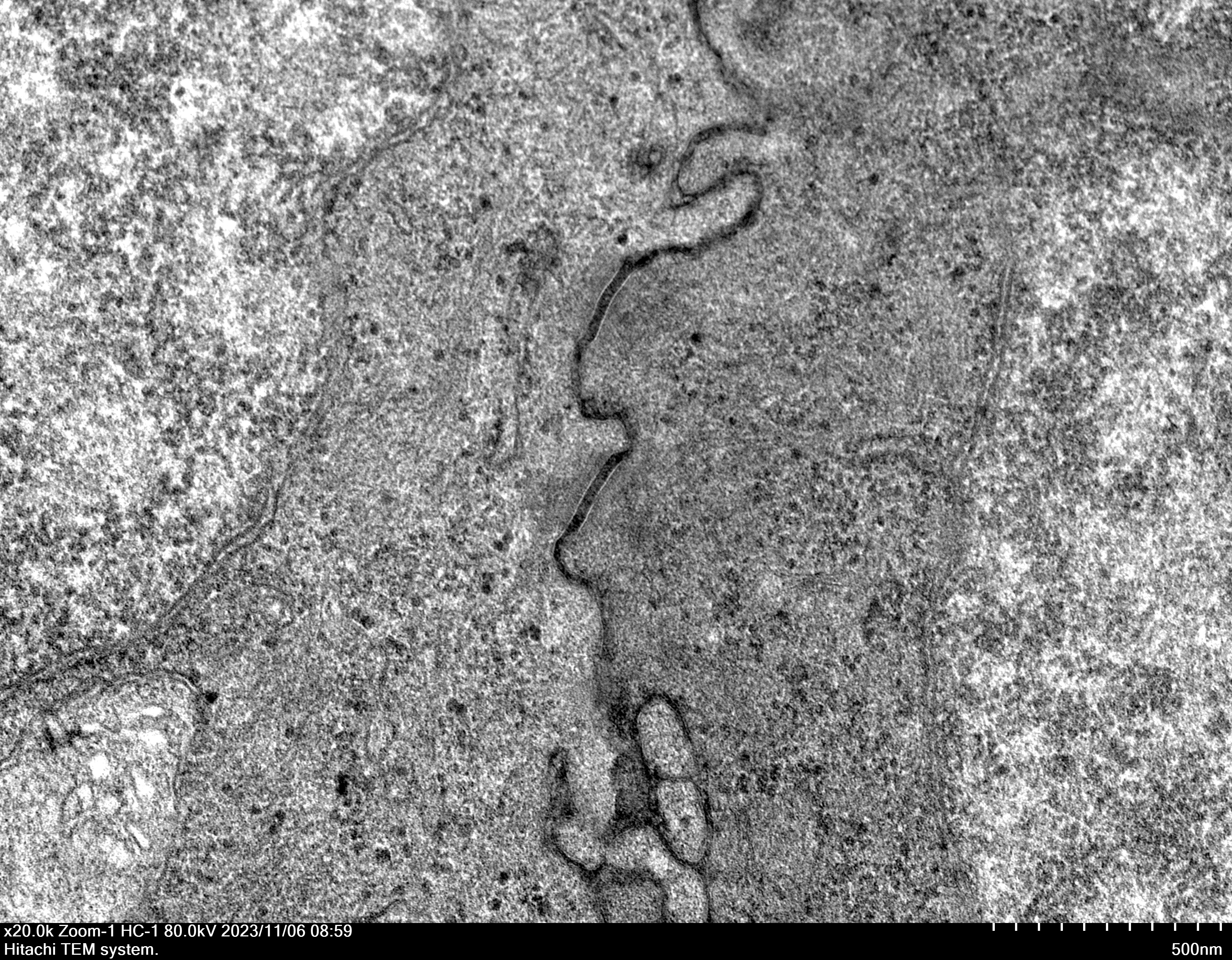

Supplement: Supplementary file 3 — Source data Fig. 1 [file 44319_2025_438_MOESM3_ESM.zip › SD figure 1/Fig. 1N/GFP.tif]

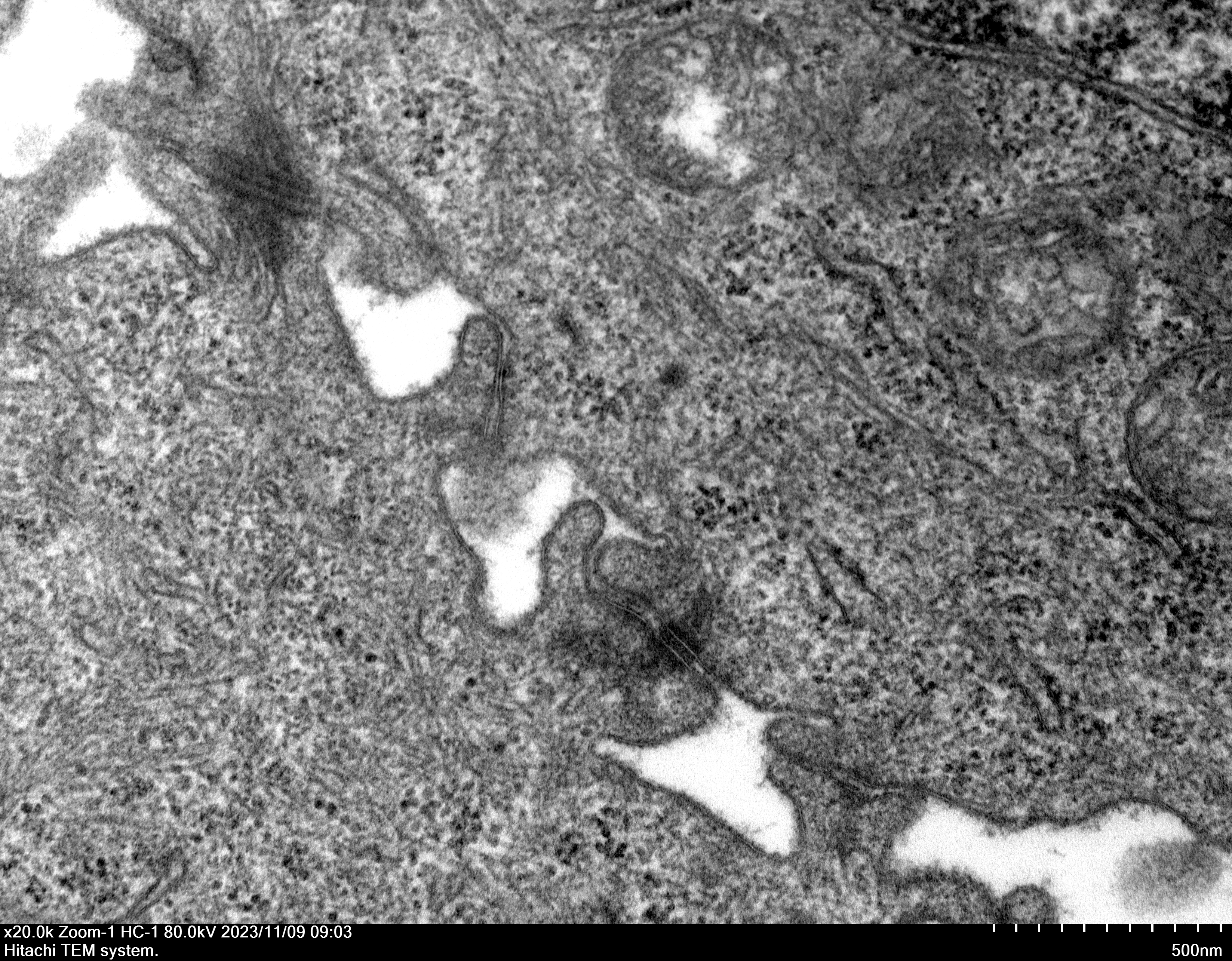

Supplement: Supplementary file 3 — Source data Fig. 1 [file 44319_2025_438_MOESM3_ESM.zip › SD figure 1/Fig. 1N/GFP-HDAC6.tif]

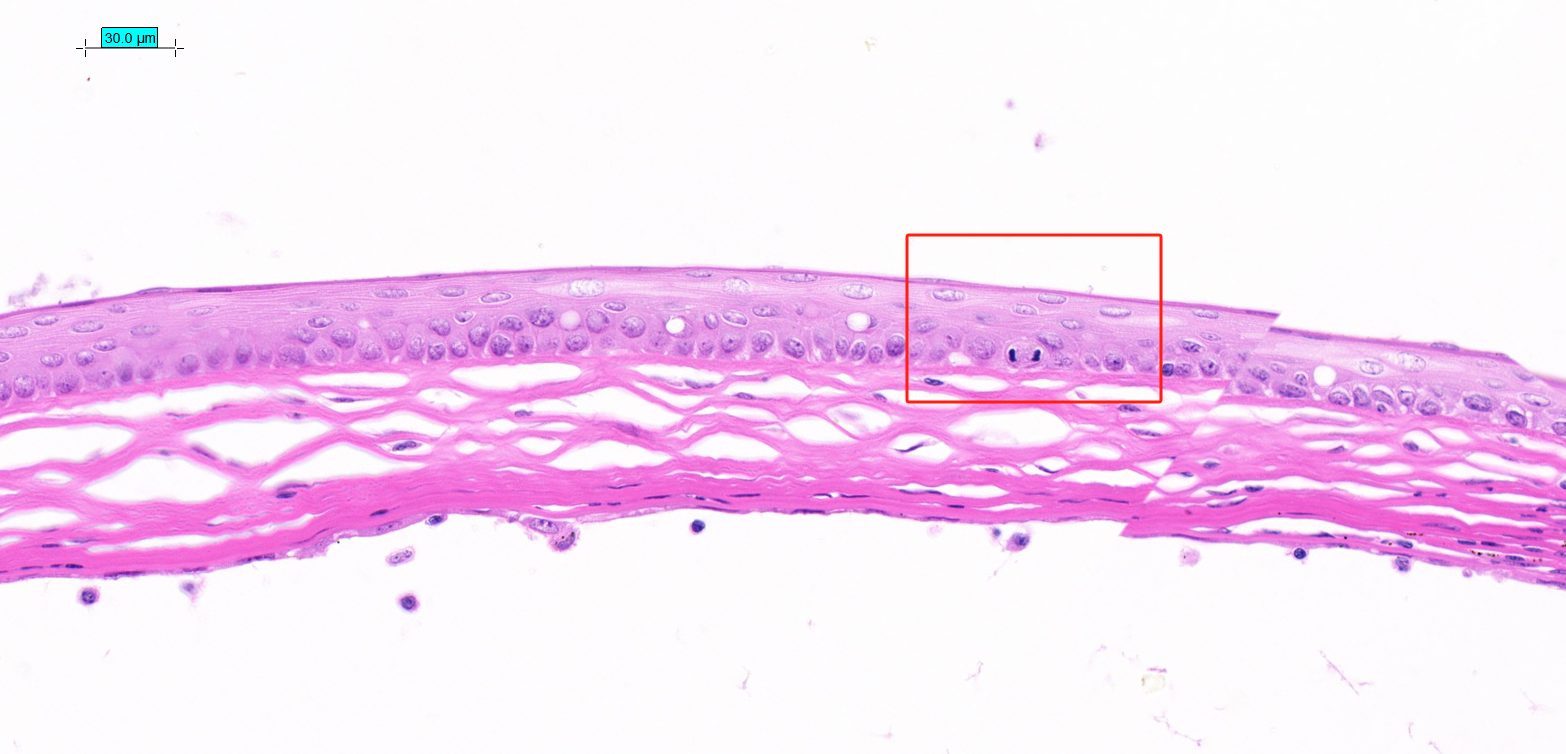

Supplement: Supplementary file 3 — Source data Fig. 1 [file 44319_2025_438_MOESM3_ESM.zip › SD figure 1/Fig. 1P/GFP.png]

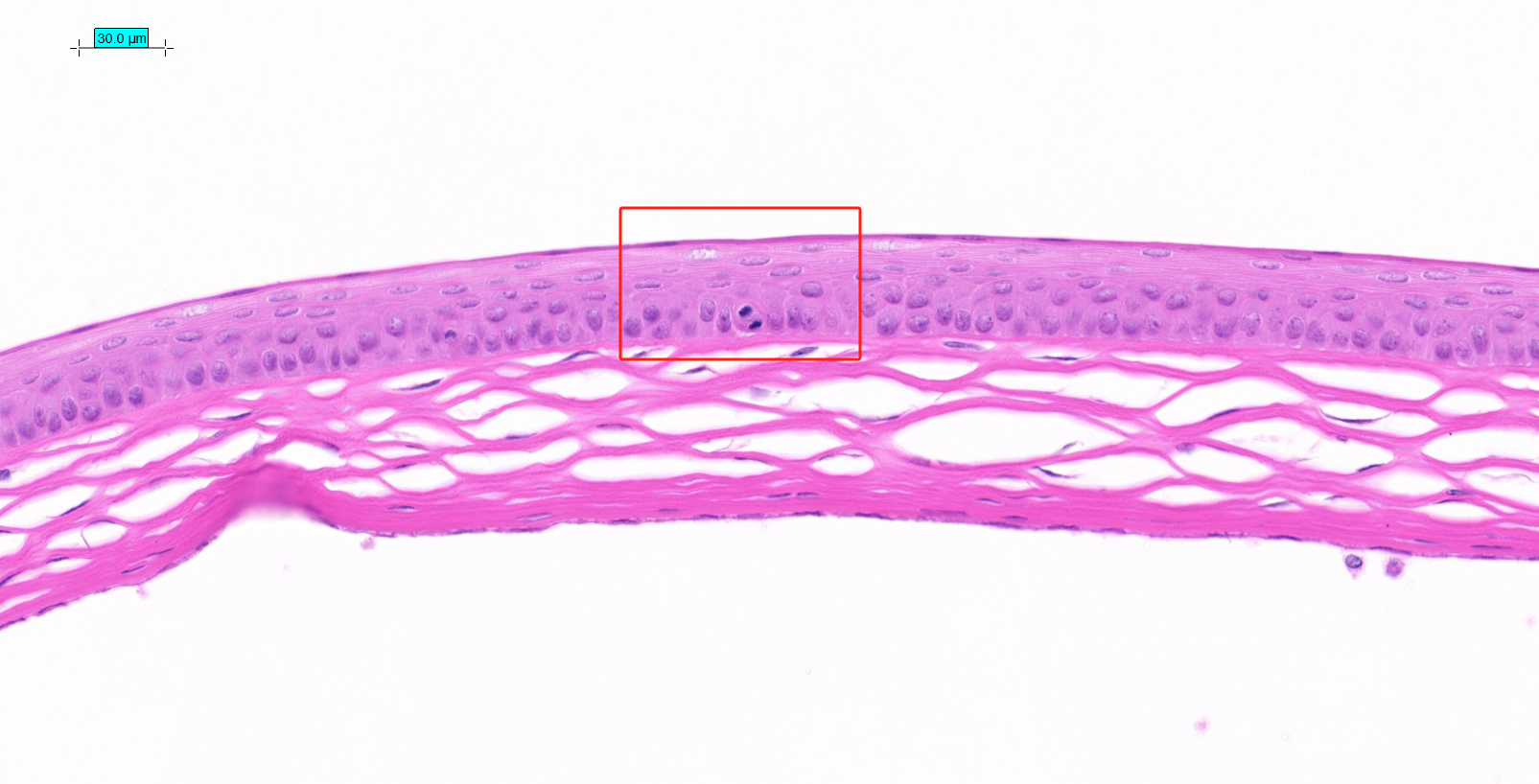

Supplement: Supplementary file 3 — Source data Fig. 1 [file 44319_2025_438_MOESM3_ESM.zip › SD figure 1/Fig. 1P/GFP-HDAC6.png]

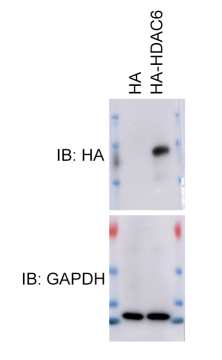

Supplement: Supplementary file 4 — Source data Fig. 2 [file 44319_2025_438_MOESM4_ESM.zip › SD figure 2/Fig. 2A/Fig. 2A.png]

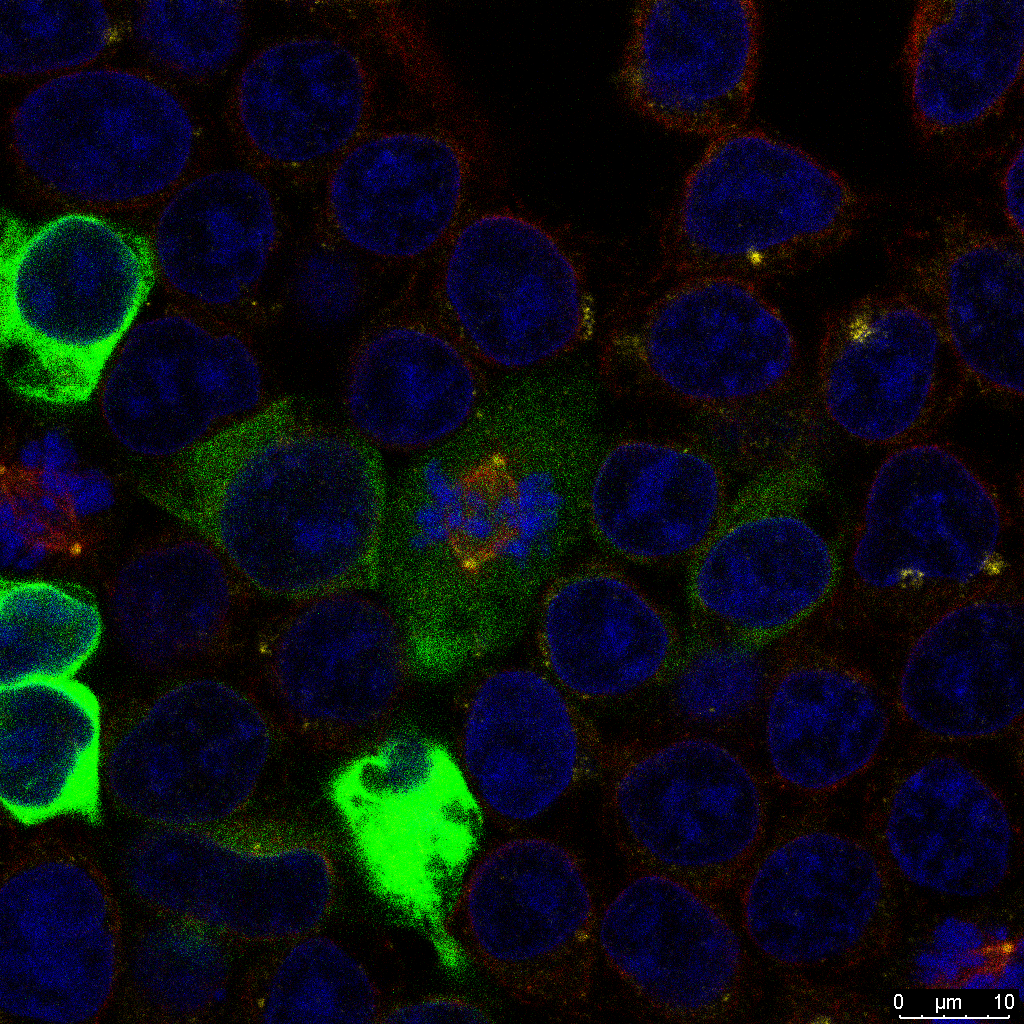

Supplement: Supplementary file 4 — Source data Fig. 2 [file 44319_2025_438_MOESM4_ESM.zip › SD figure 2/Fig. 2E/GFP-0.45.tif]

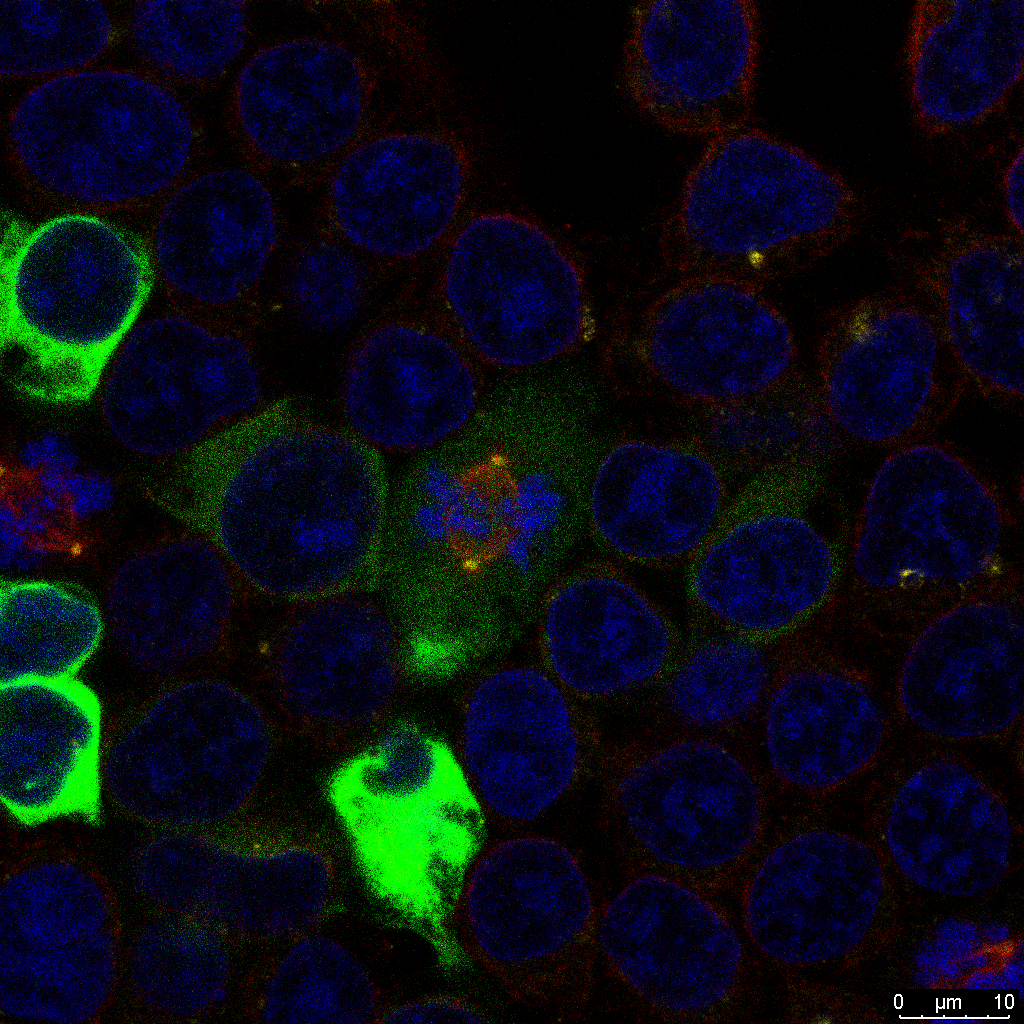

Supplement: Supplementary file 4 — Source data Fig. 2 [file 44319_2025_438_MOESM4_ESM.zip › SD figure 2/Fig. 2E/GFP-0.9.tif]

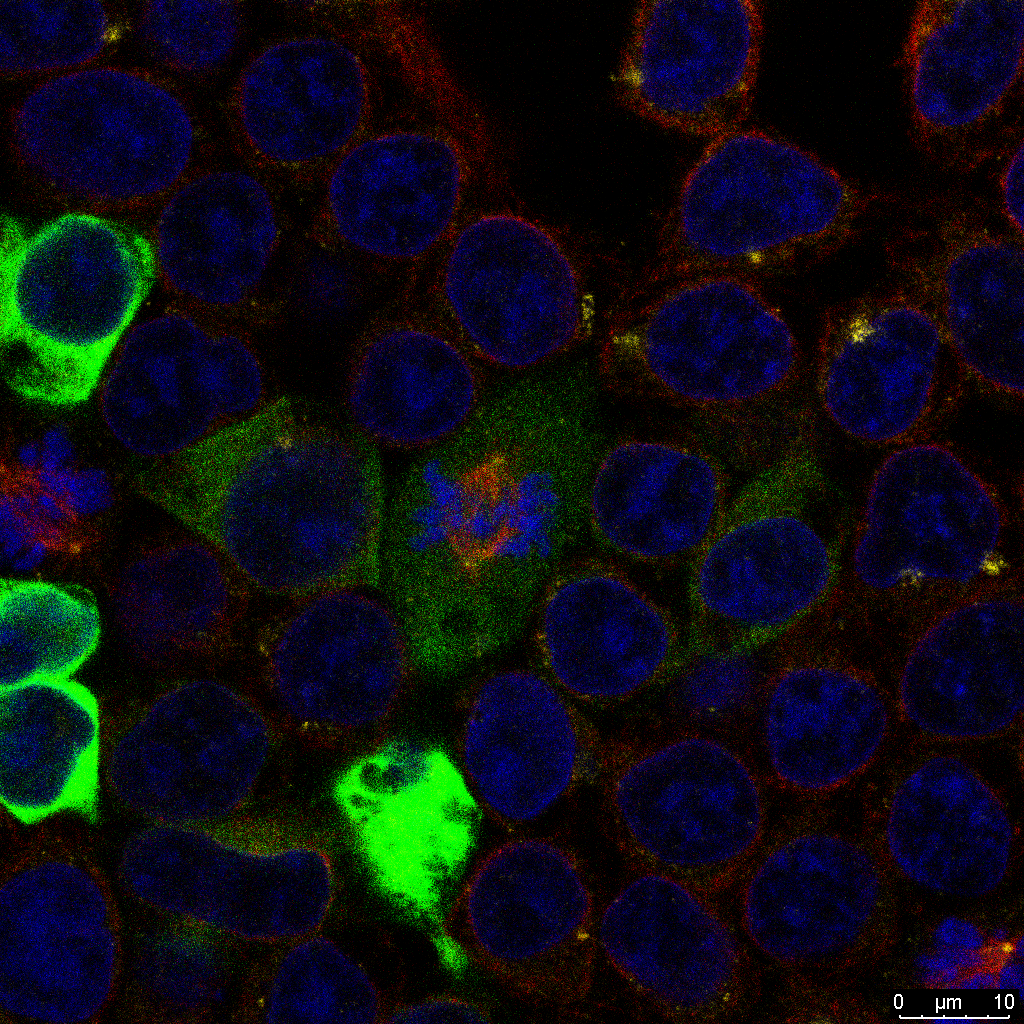

Supplement: Supplementary file 4 — Source data Fig. 2 [file 44319_2025_438_MOESM4_ESM.zip › SD figure 2/Fig. 2E/GFP-0.tif]

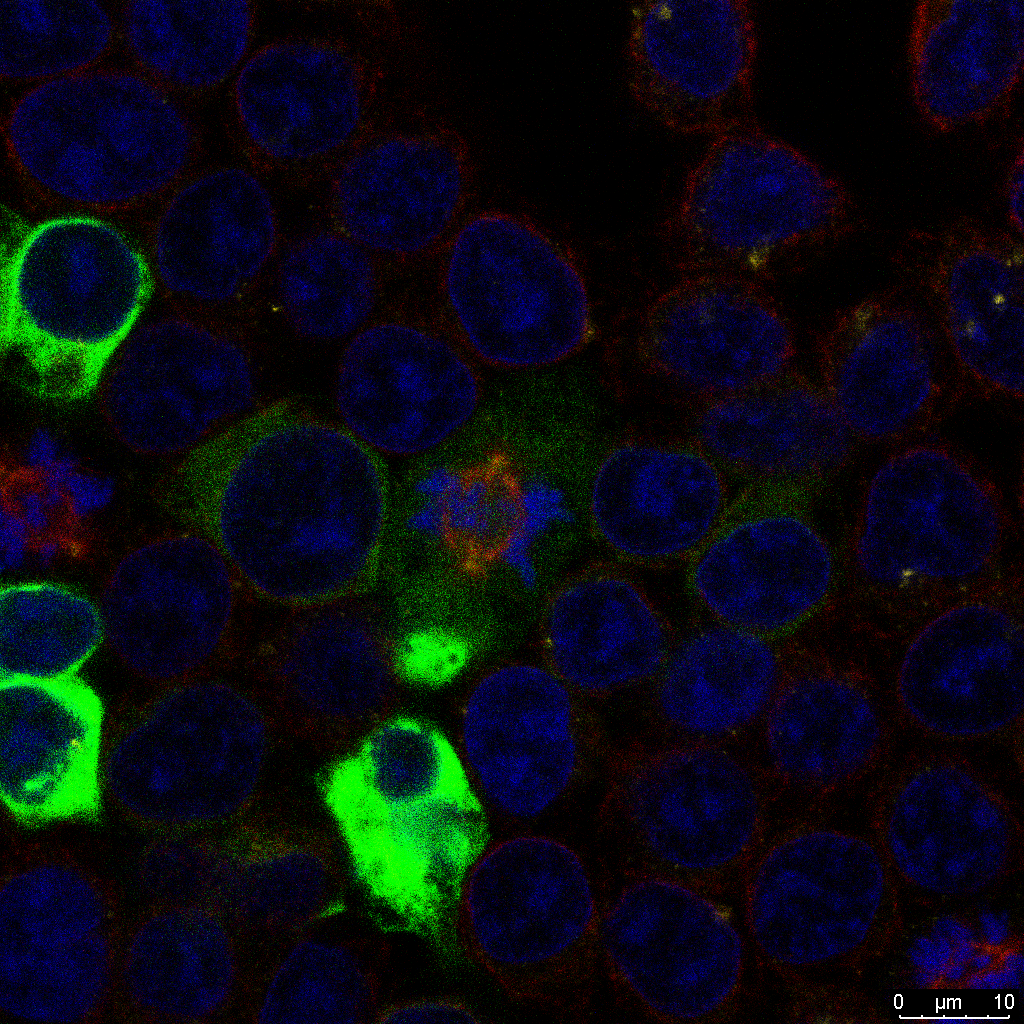

Supplement: Supplementary file 4 — Source data Fig. 2 [file 44319_2025_438_MOESM4_ESM.zip › SD figure 2/Fig. 2E/GFP-1.35.tif]

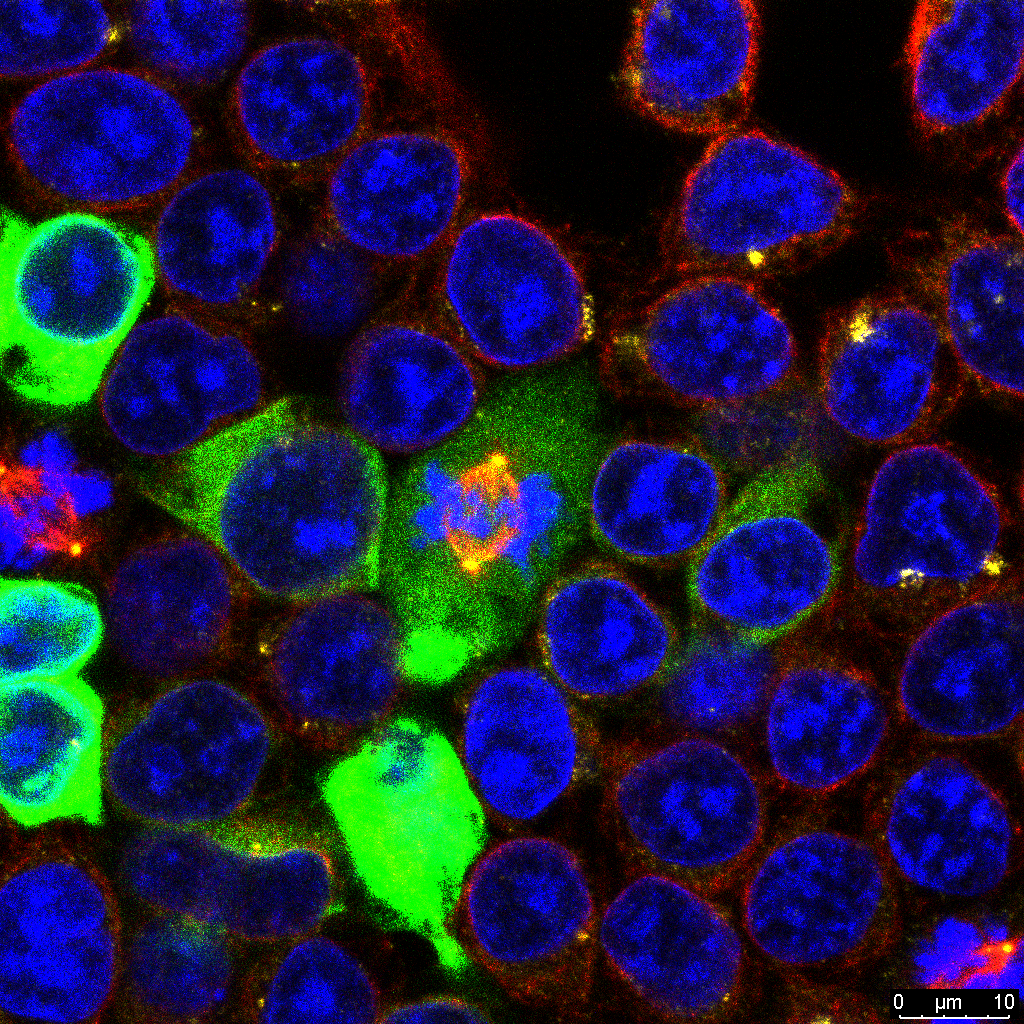

Supplement: Supplementary file 4 — Source data Fig. 2 [file 44319_2025_438_MOESM4_ESM.zip › SD figure 2/Fig. 2E/GFP-3D.tif]

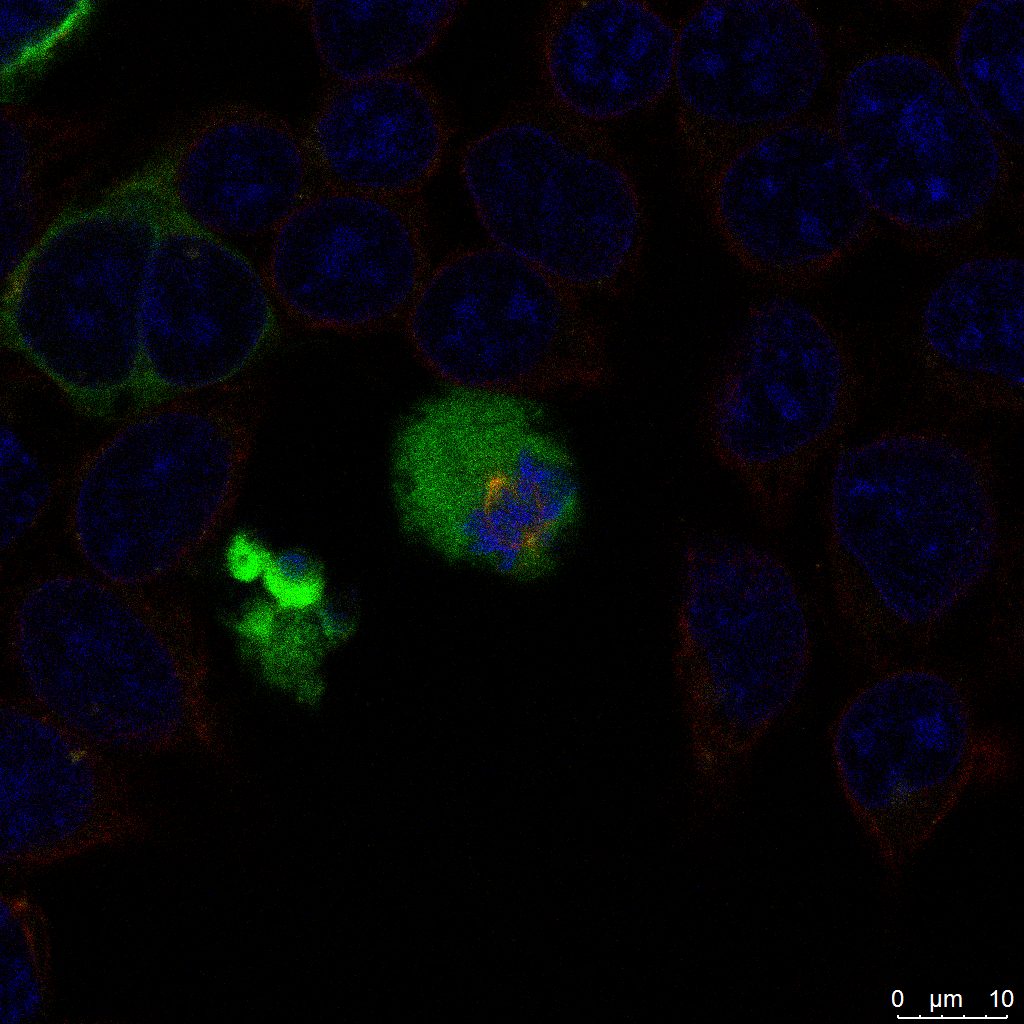

Supplement: Supplementary file 4 — Source data Fig. 2 [file 44319_2025_438_MOESM4_ESM.zip › SD figure 2/Fig. 2E/GFP-HDAC6-0.45.tif]

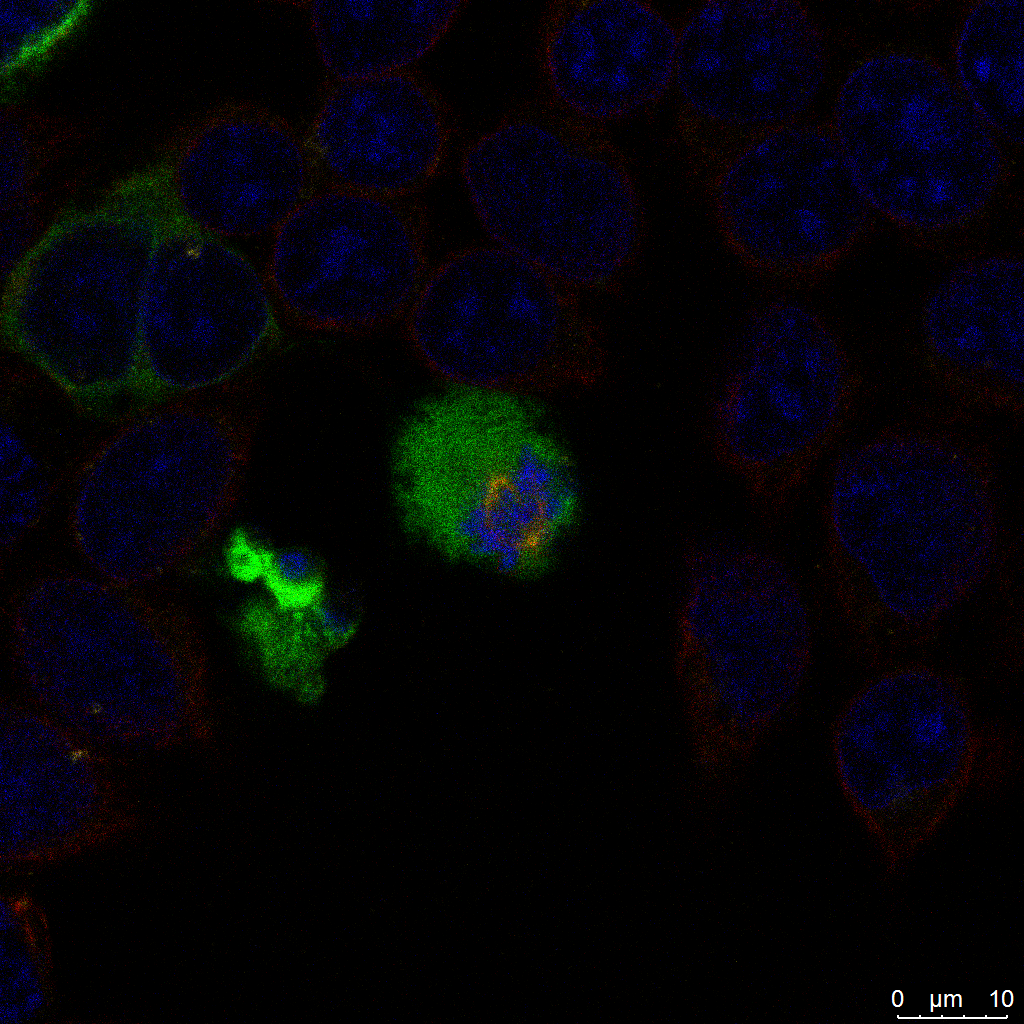

Supplement: Supplementary file 4 — Source data Fig. 2 [file 44319_2025_438_MOESM4_ESM.zip › SD figure 2/Fig. 2E/GFP-HDAC6-0.9.tif]

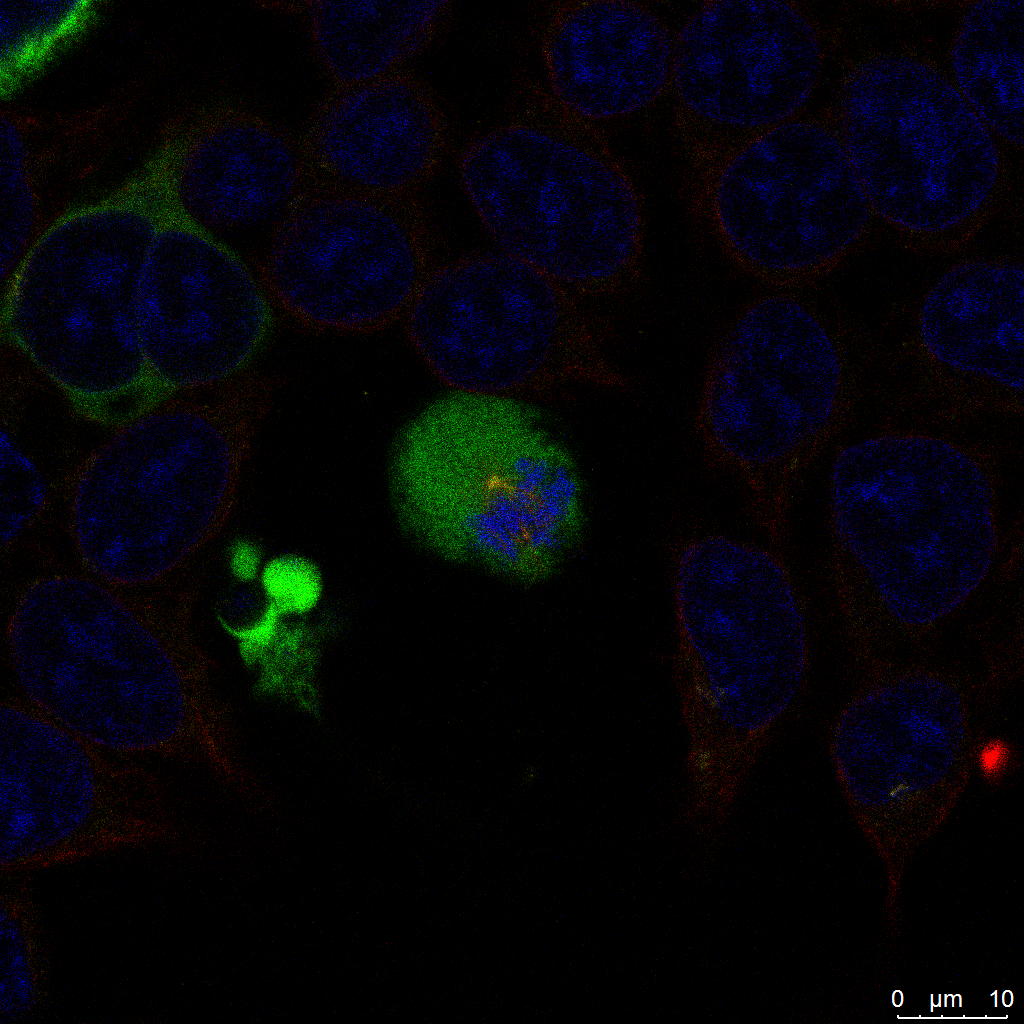

Supplement: Supplementary file 4 — Source data Fig. 2 [file 44319_2025_438_MOESM4_ESM.zip › SD figure 2/Fig. 2E/GFP-HDAC6-0.tif]

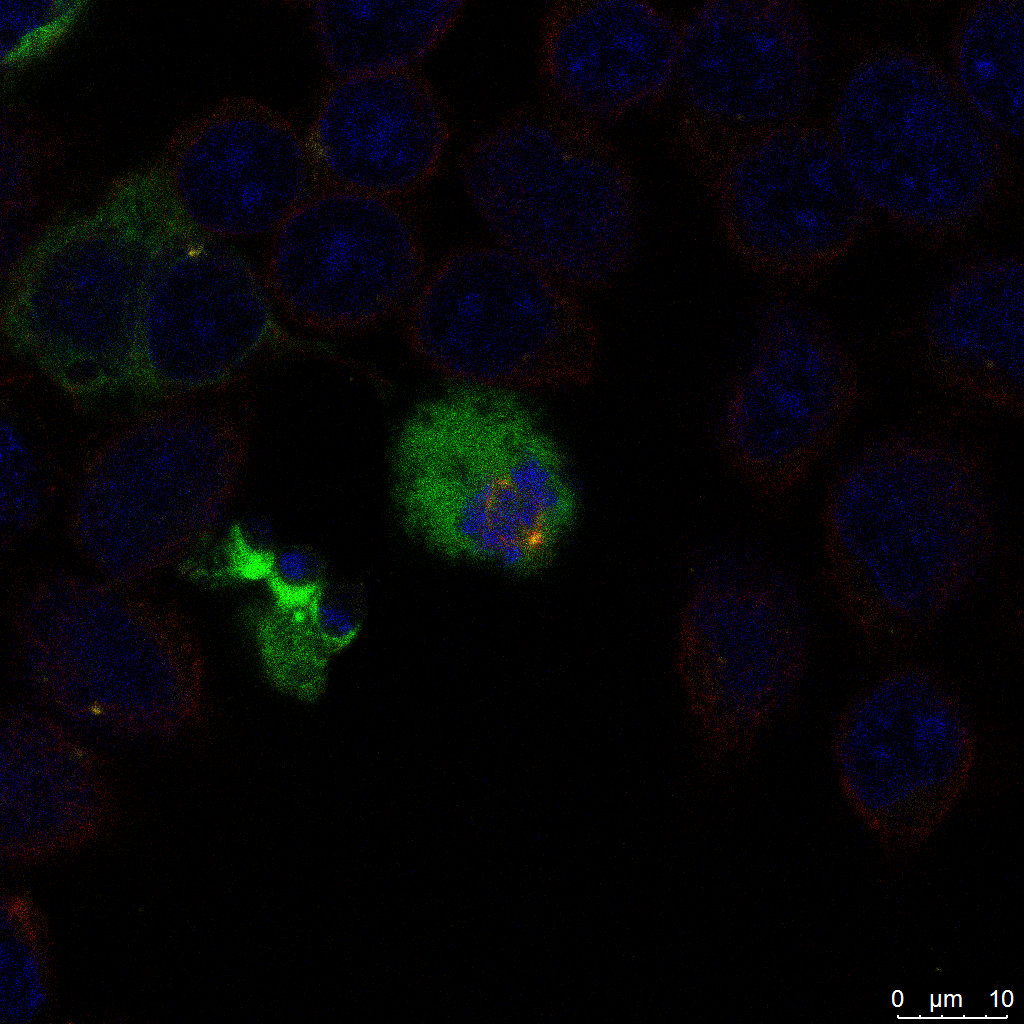

Supplement: Supplementary file 4 — Source data Fig. 2 [file 44319_2025_438_MOESM4_ESM.zip › SD figure 2/Fig. 2E/GFP-HDAC6-1.35.tif]

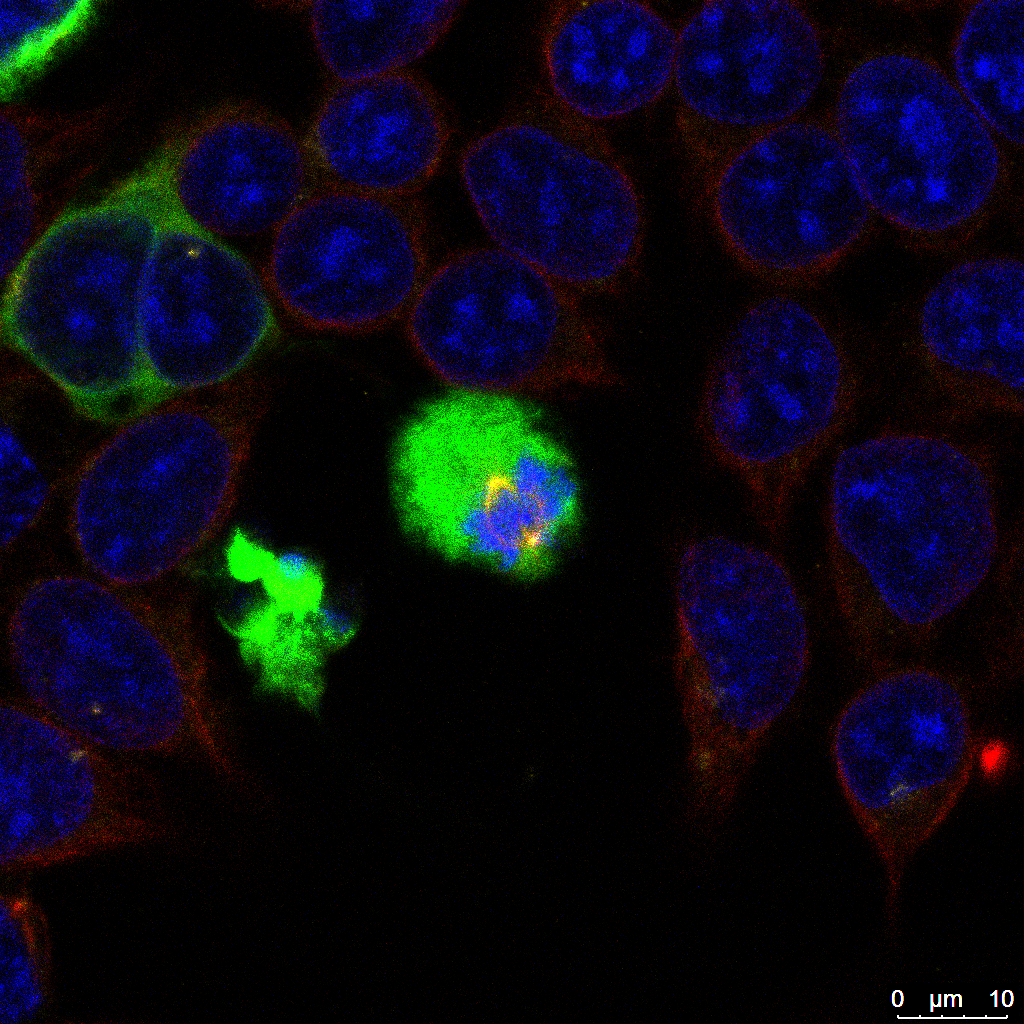

Supplement: Supplementary file 4 — Source data Fig. 2 [file 44319_2025_438_MOESM4_ESM.zip › SD figure 2/Fig. 2E/GFP-HDAC6-3D.tif]

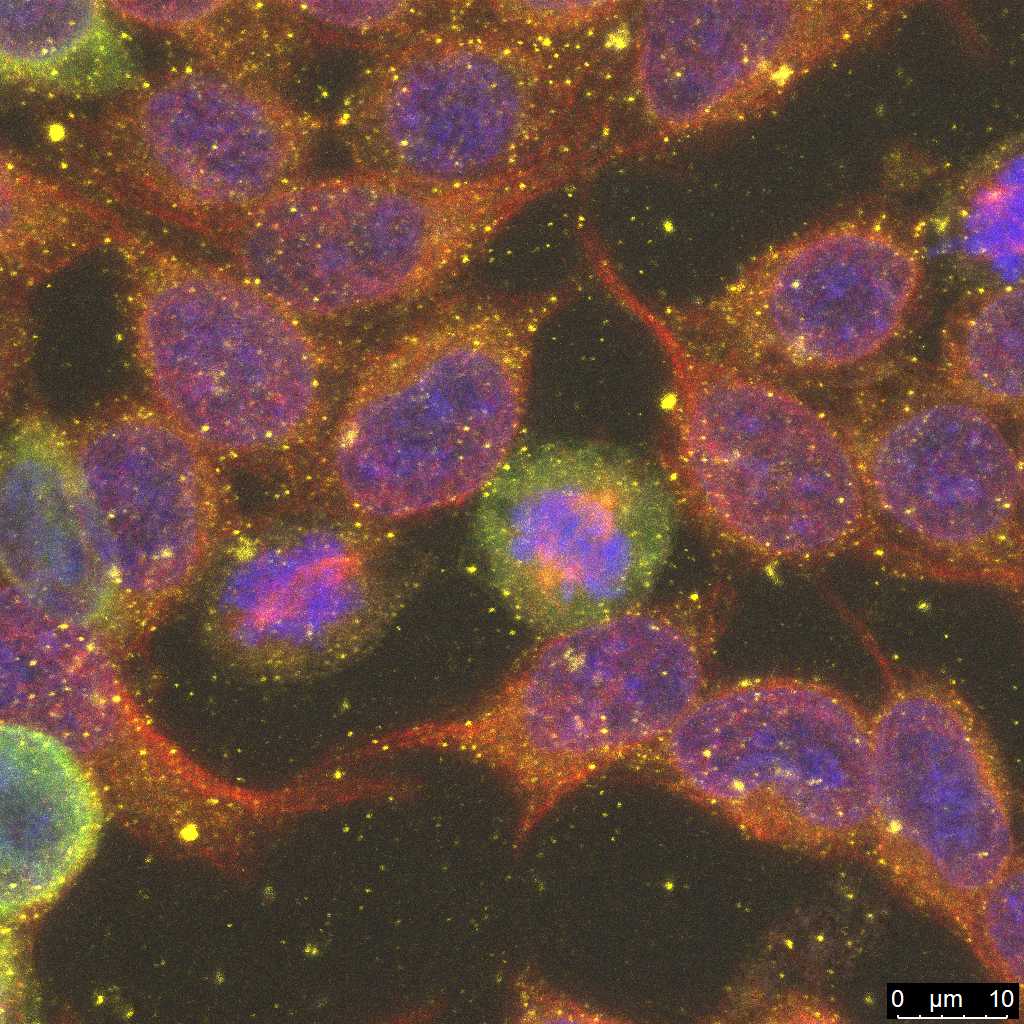

Supplement: Supplementary file 4 — Source data Fig. 2 [file 44319_2025_438_MOESM4_ESM.zip › SD figure 2/Fig. 2H/GFP.tif]

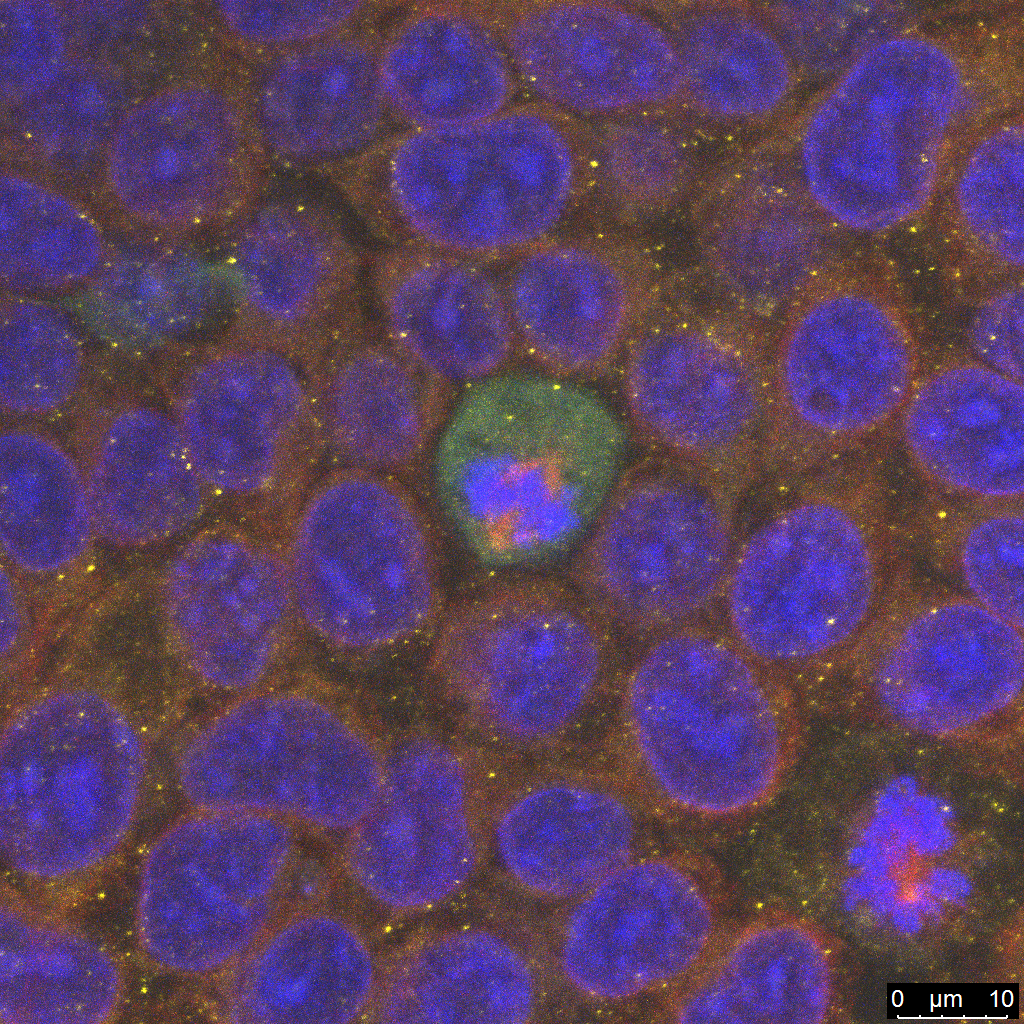

Supplement: Supplementary file 4 — Source data Fig. 2 [file 44319_2025_438_MOESM4_ESM.zip › SD figure 2/Fig. 2H/GFP-HDAC6.tif]

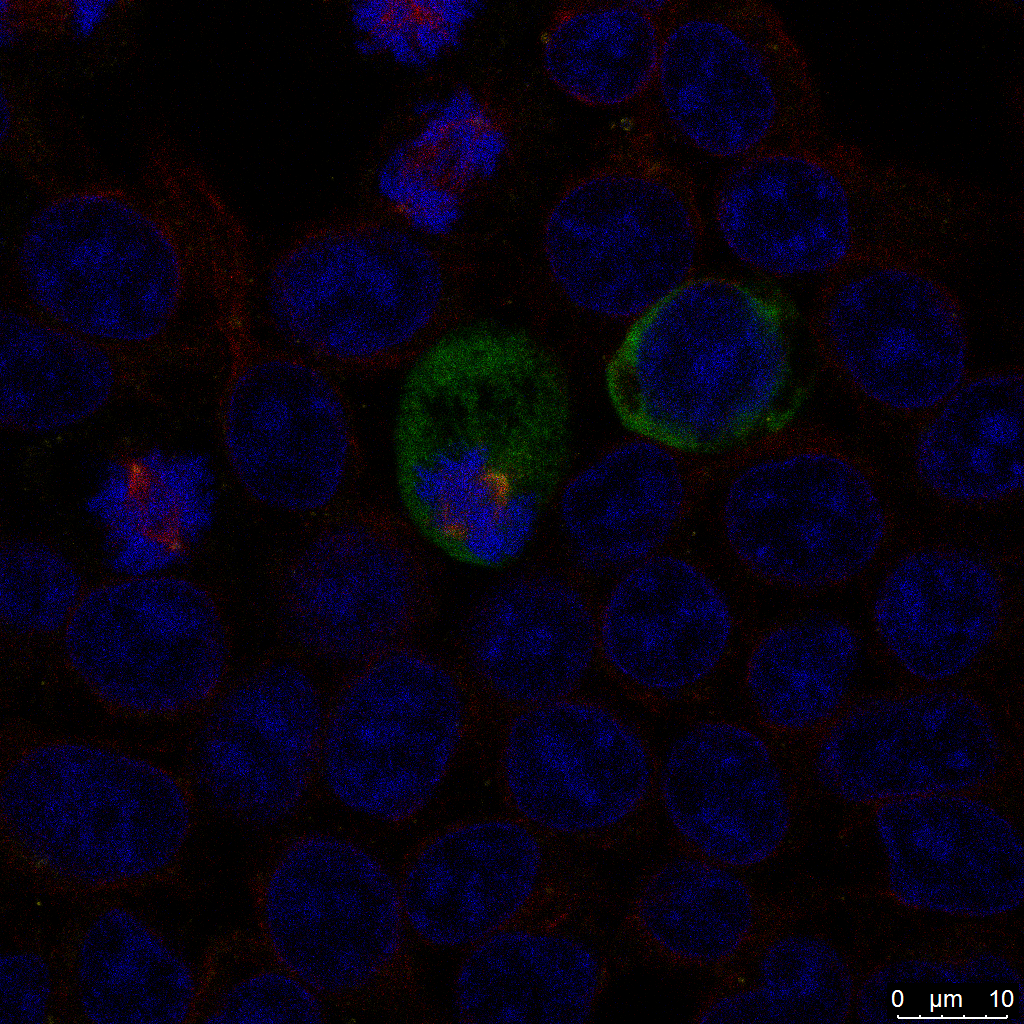

Supplement: Supplementary file 4 — Source data Fig. 2 [file 44319_2025_438_MOESM4_ESM.zip › SD figure 2/Fig. 2J/GFP-HDAC6-Control-0.45.tif]

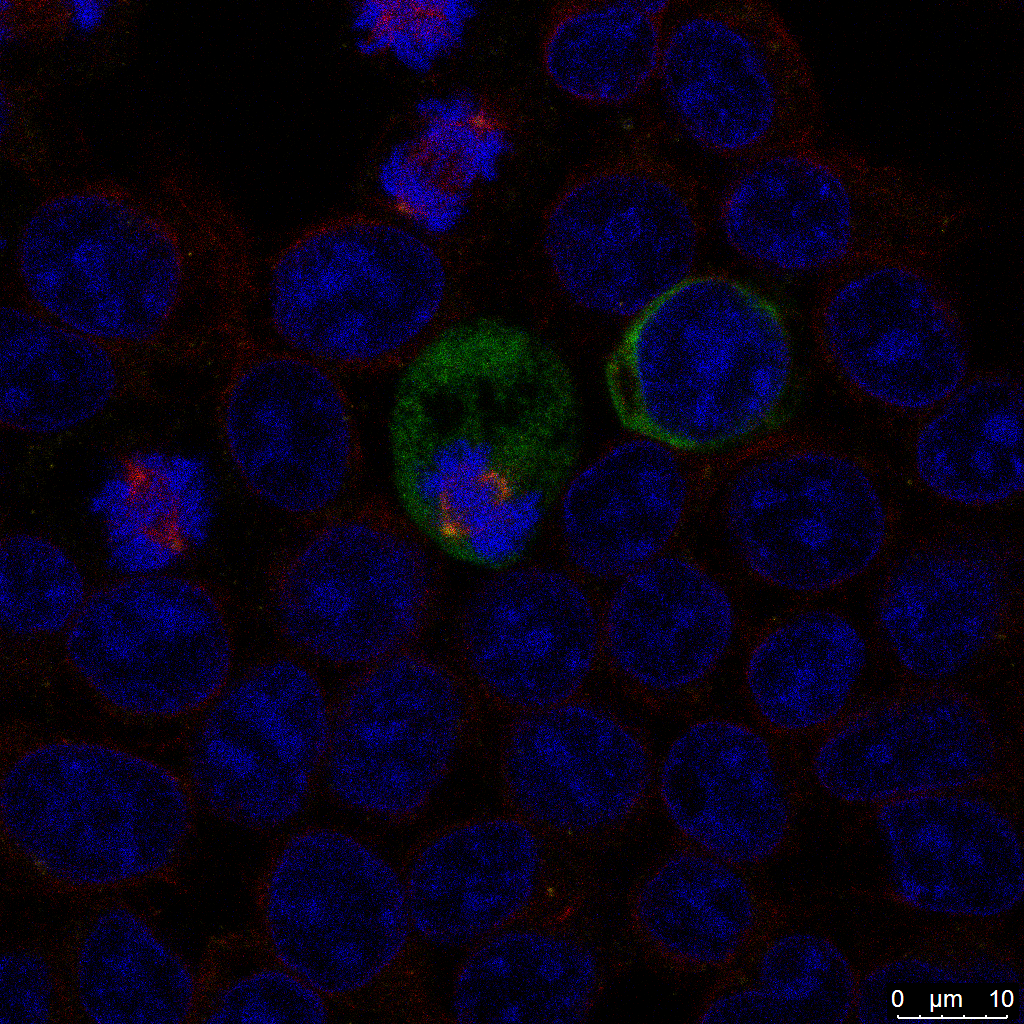

Supplement: Supplementary file 4 — Source data Fig. 2 [file 44319_2025_438_MOESM4_ESM.zip › SD figure 2/Fig. 2J/GFP-HDAC6-Control-0.9.tif]

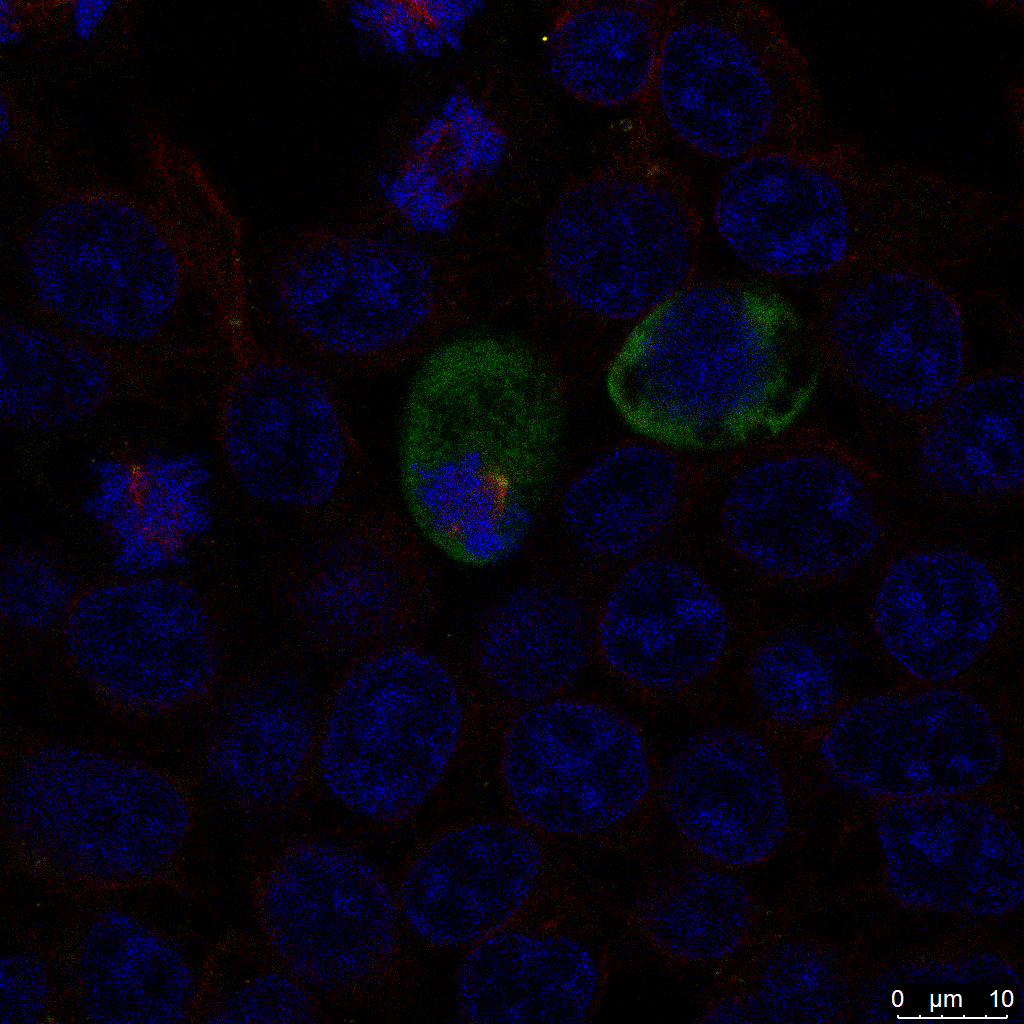

Supplement: Supplementary file 4 — Source data Fig. 2 [file 44319_2025_438_MOESM4_ESM.zip › SD figure 2/Fig. 2J/GFP-HDAC6-Control-0.tif]

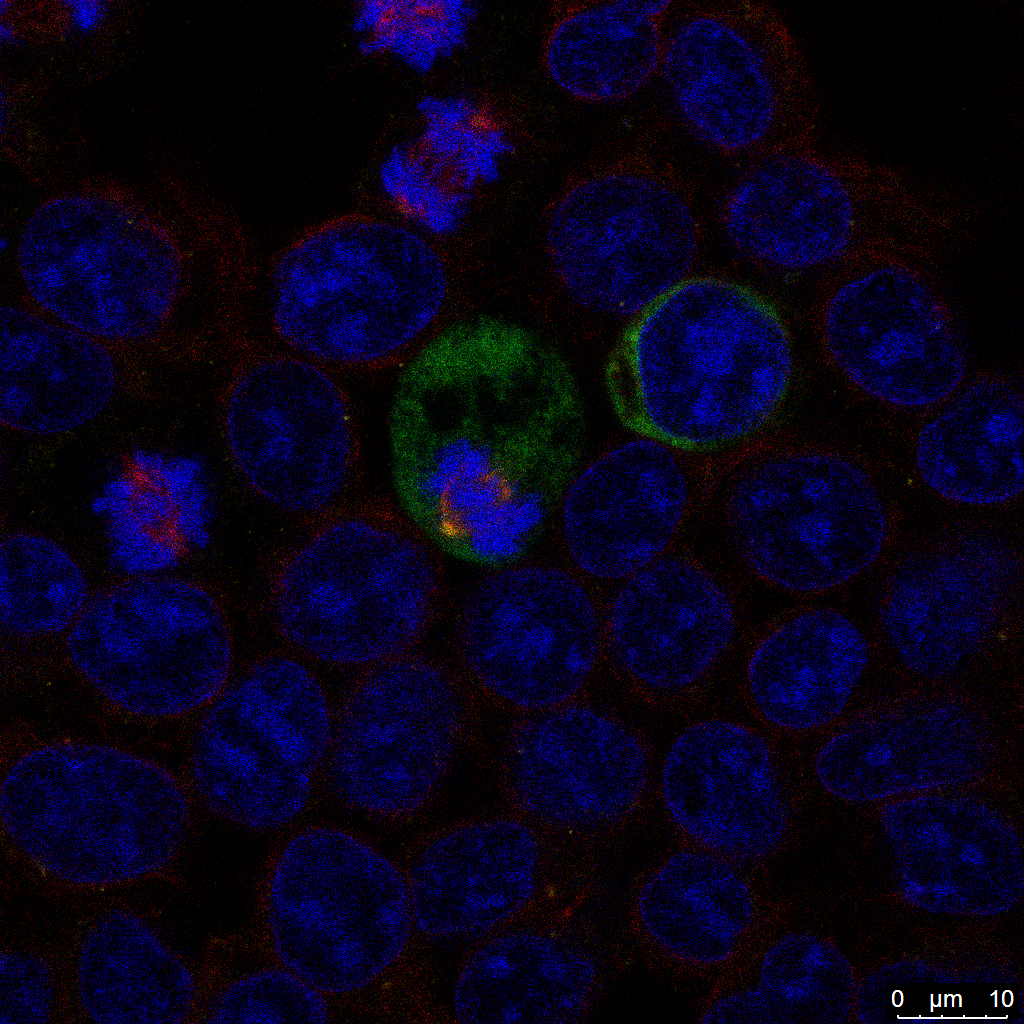

Supplement: Supplementary file 4 — Source data Fig. 2 [file 44319_2025_438_MOESM4_ESM.zip › SD figure 2/Fig. 2J/GFP-HDAC6-Control-1.35.tif]

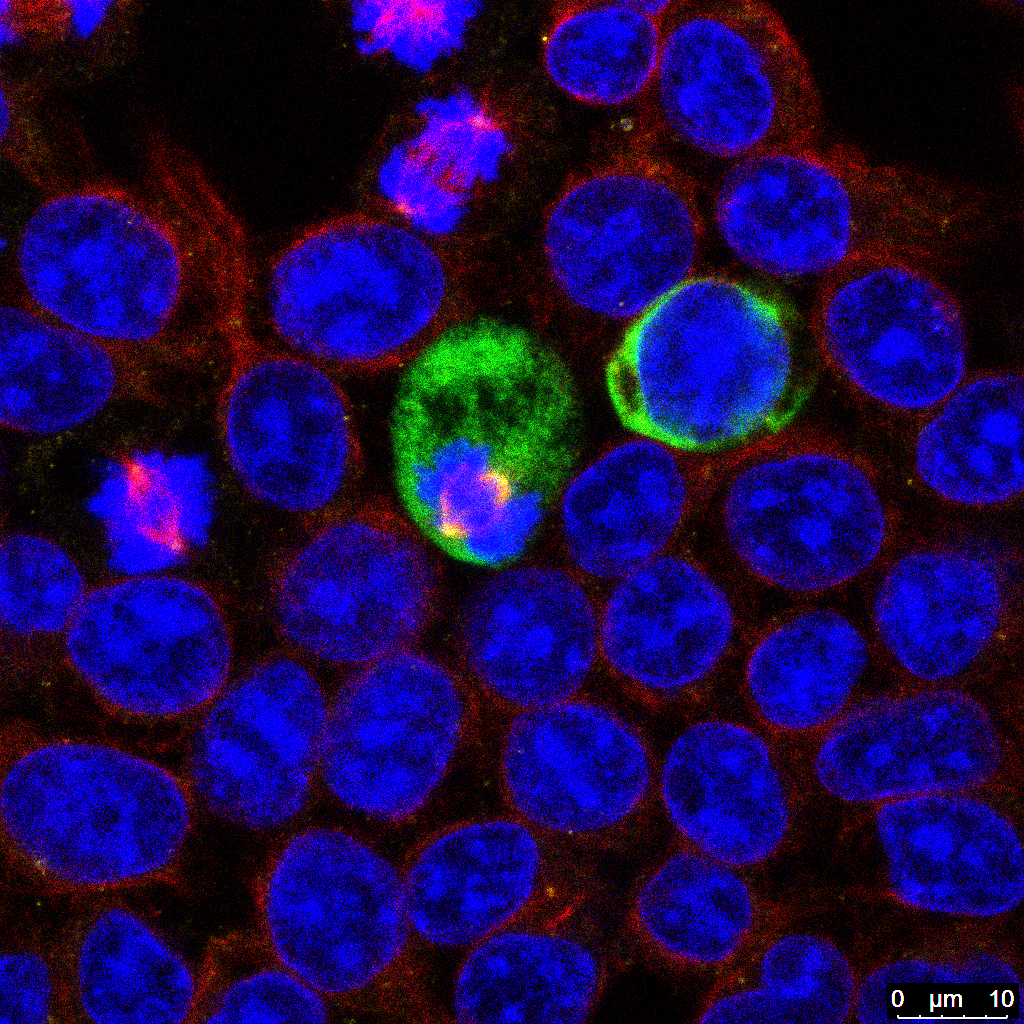

Supplement: Supplementary file 4 — Source data Fig. 2 [file 44319_2025_438_MOESM4_ESM.zip › SD figure 2/Fig. 2J/GFP-HDAC6-Control-3D.tif]

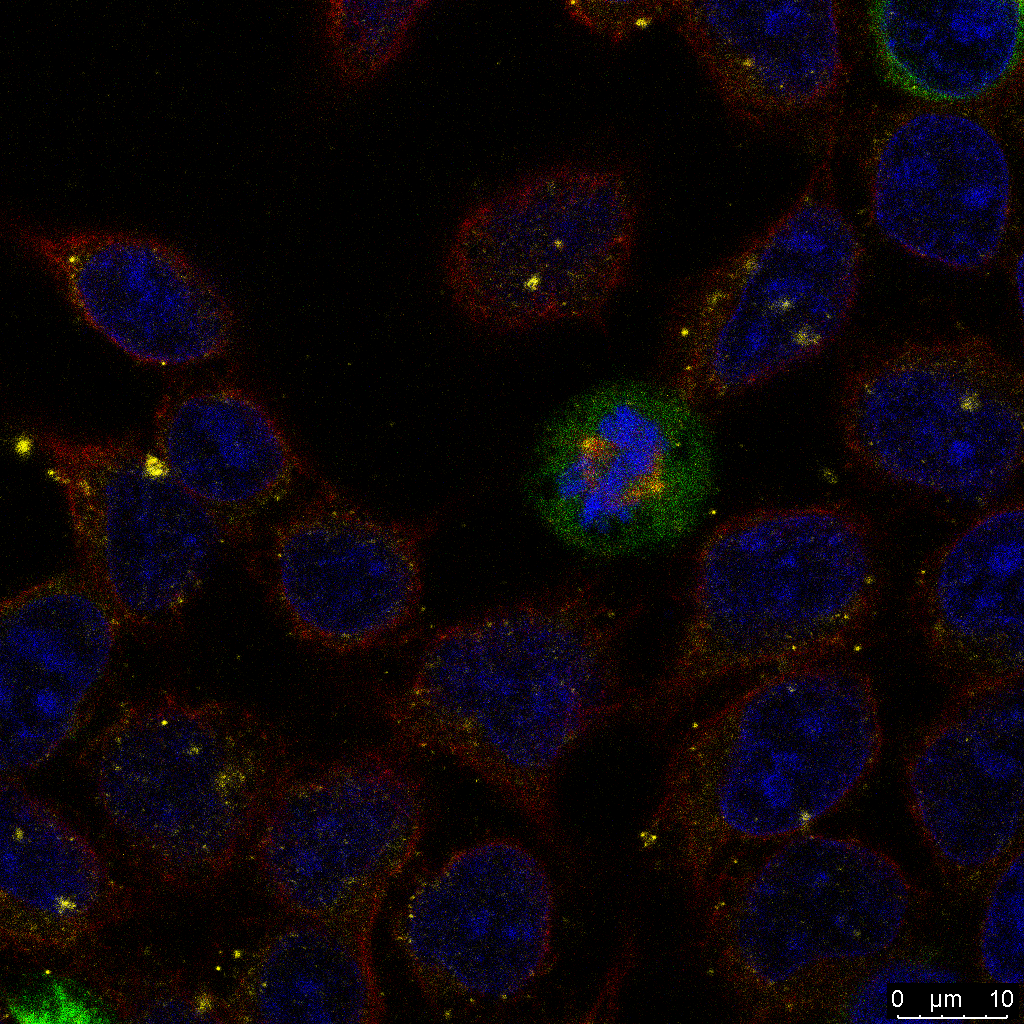

Supplement: Supplementary file 4 — Source data Fig. 2 [file 44319_2025_438_MOESM4_ESM.zip › SD figure 2/Fig. 2J/GFP-HDAC6-Tubacin-0.45.tif]

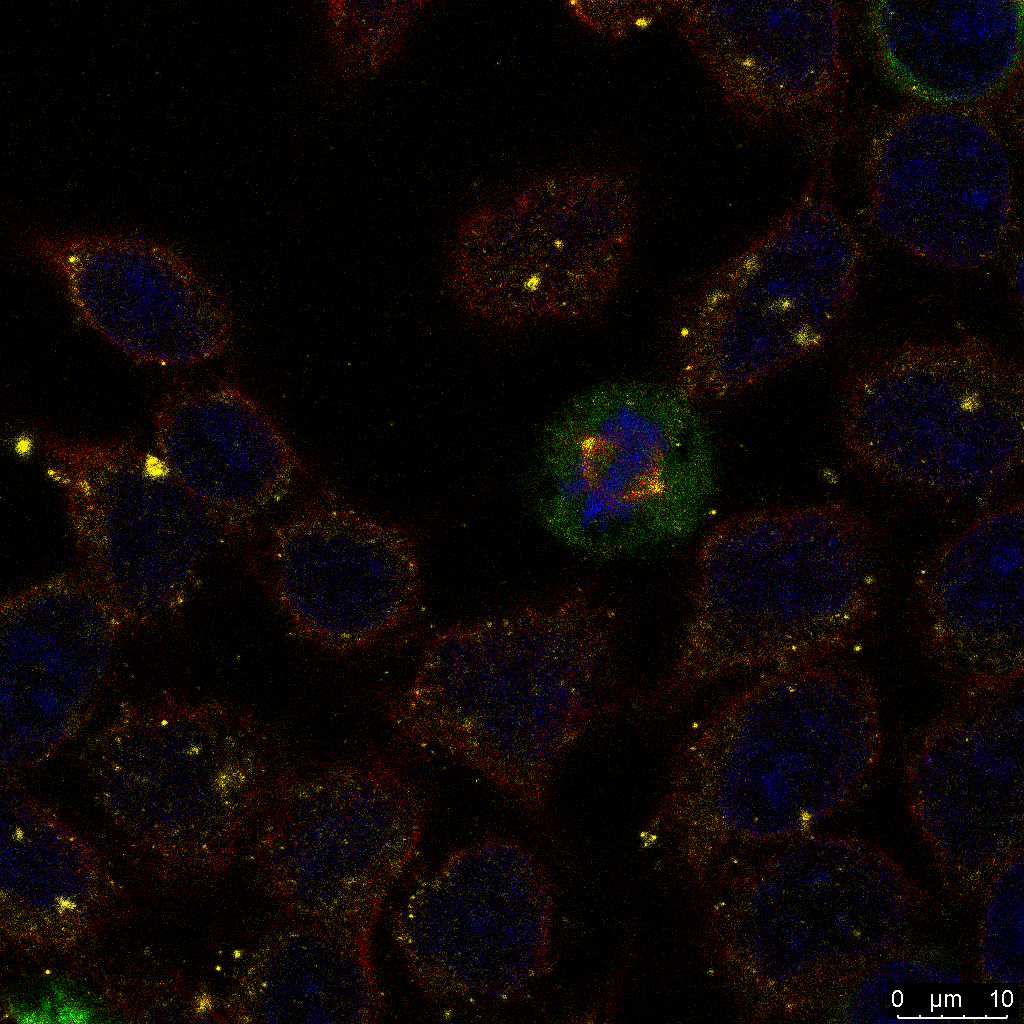

Supplement: Supplementary file 4 — Source data Fig. 2 [file 44319_2025_438_MOESM4_ESM.zip › SD figure 2/Fig. 2J/GFP-HDAC6-Tubacin-0.9.tif]

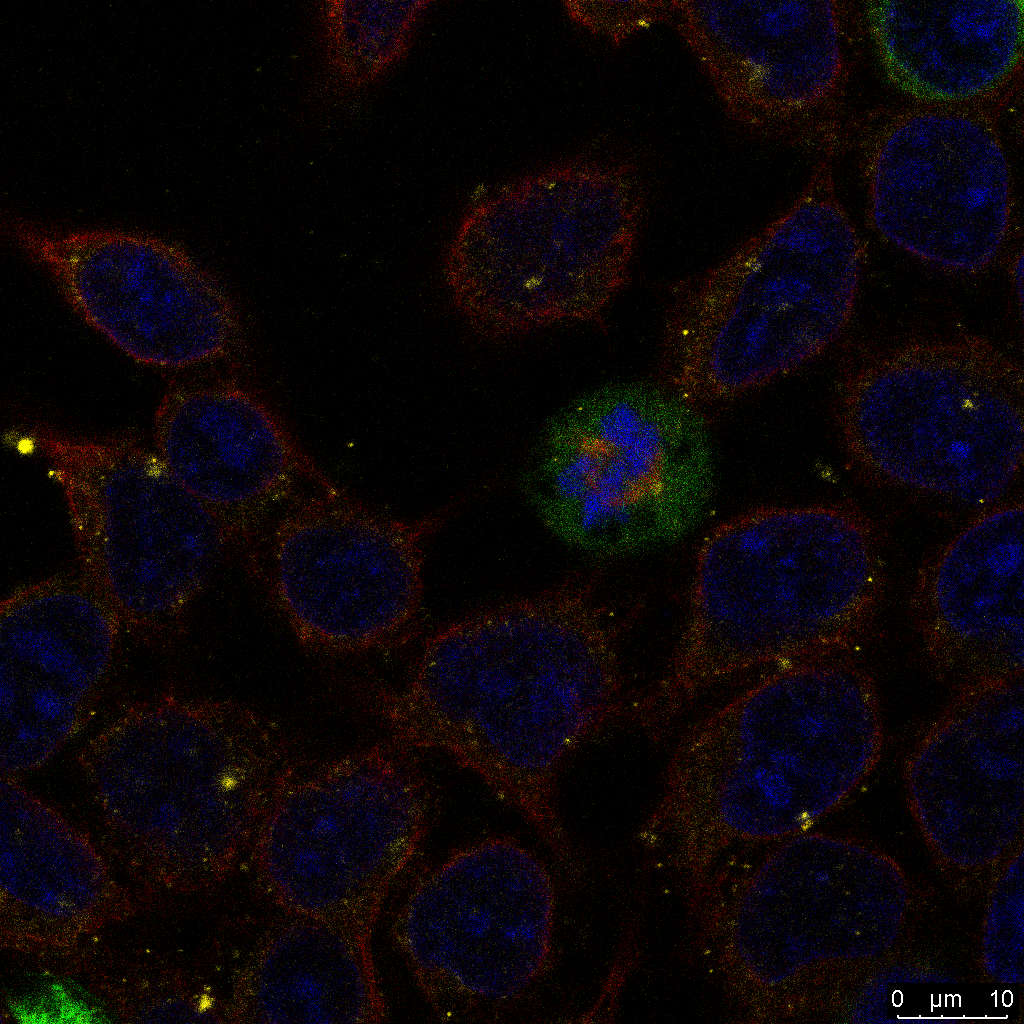

Supplement: Supplementary file 4 — Source data Fig. 2 [file 44319_2025_438_MOESM4_ESM.zip › SD figure 2/Fig. 2J/GFP-HDAC6-Tubacin-0.tif]

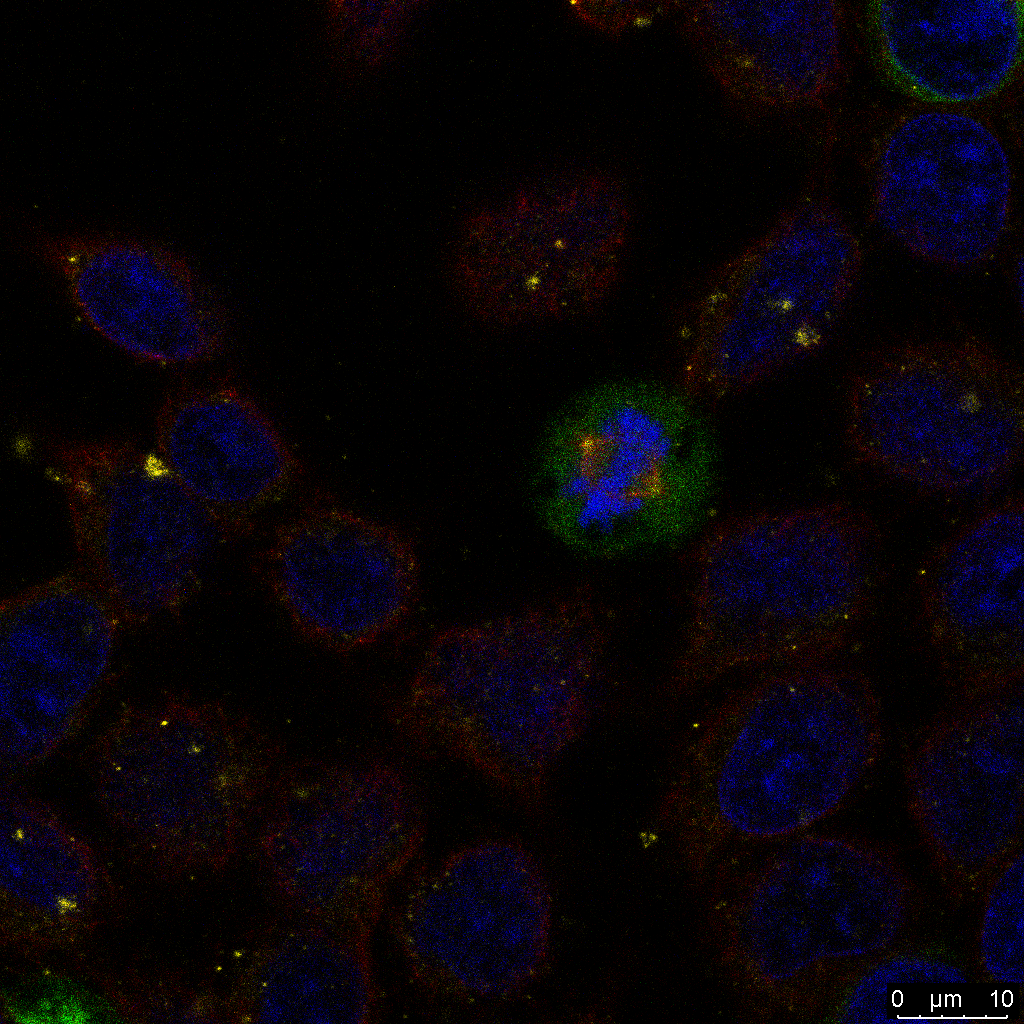

Supplement: Supplementary file 4 — Source data Fig. 2 [file 44319_2025_438_MOESM4_ESM.zip › SD figure 2/Fig. 2J/GFP-HDAC6-Tubacin-1.35.tif]

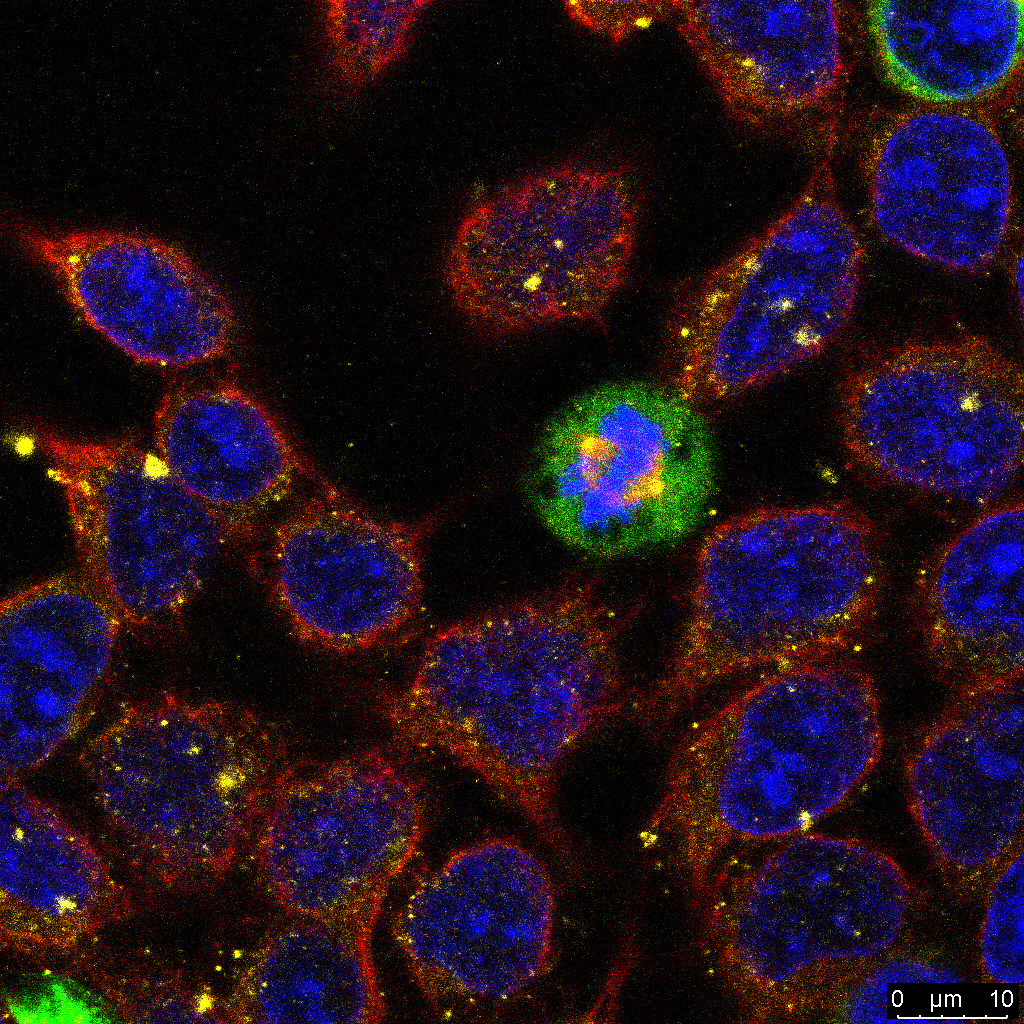

Supplement: Supplementary file 4 — Source data Fig. 2 [file 44319_2025_438_MOESM4_ESM.zip › SD figure 2/Fig. 2J/GFP-HDAC6-Tubacin-3D.tif]

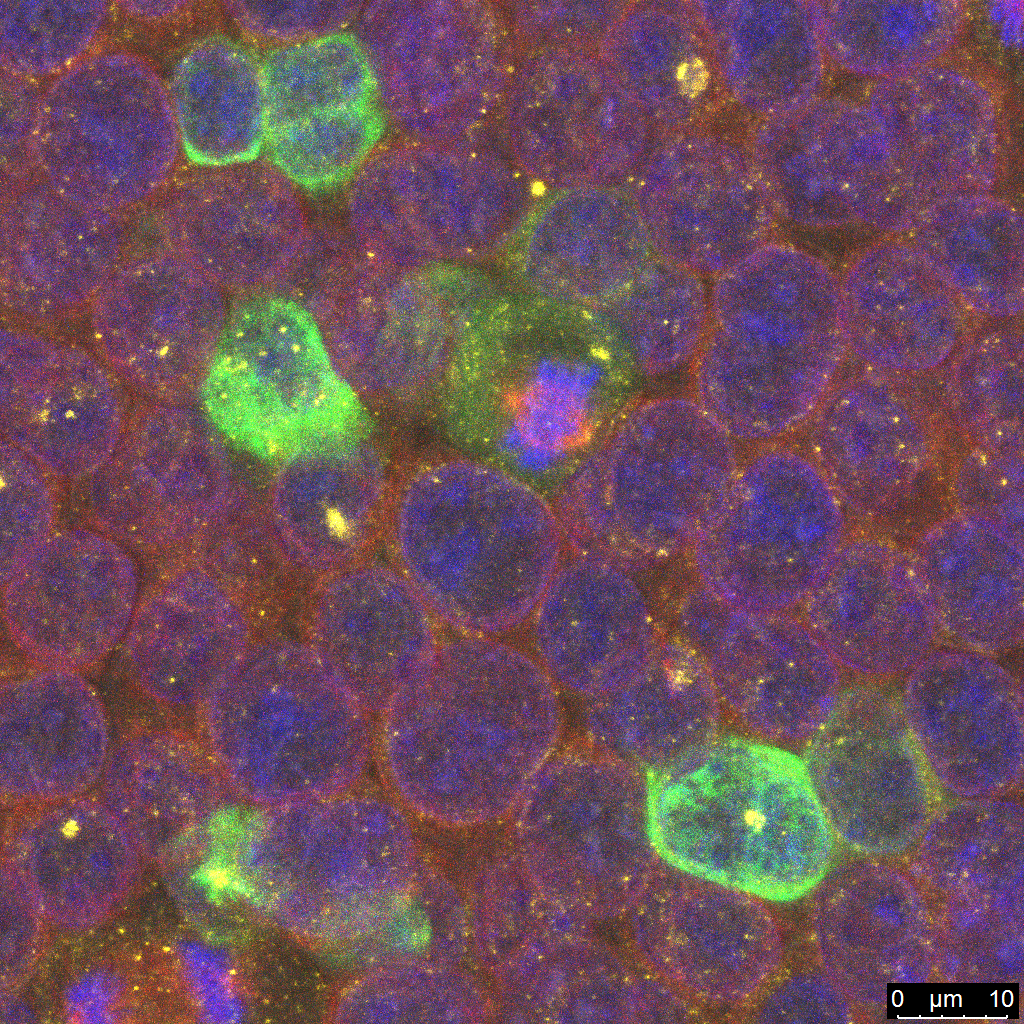

Supplement: Supplementary file 4 — Source data Fig. 2 [file 44319_2025_438_MOESM4_ESM.zip › SD figure 2/Fig. 2L/GFP-HDAC6-Control.tif]

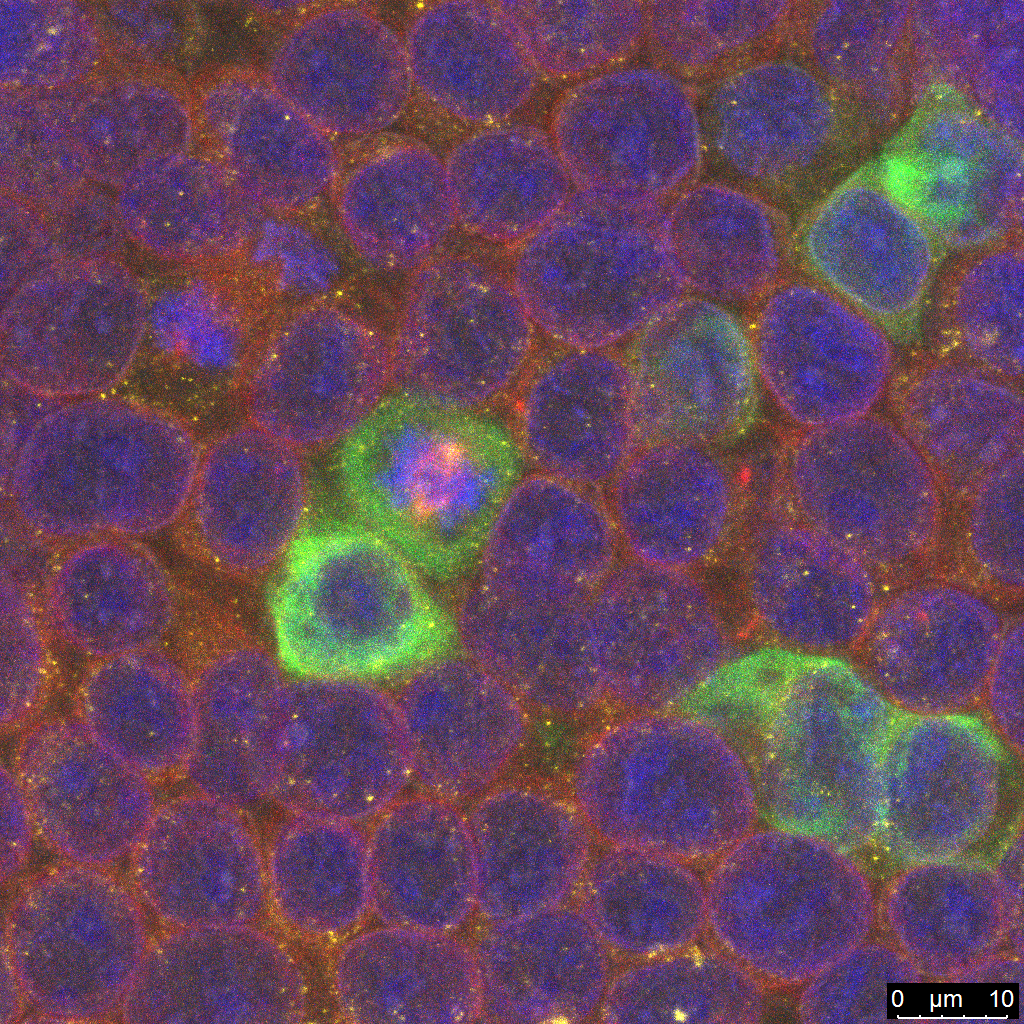

Supplement: Supplementary file 4 — Source data Fig. 2 [file 44319_2025_438_MOESM4_ESM.zip › SD figure 2/Fig. 2L/GFP-HDAC6-Tubacin.tif]

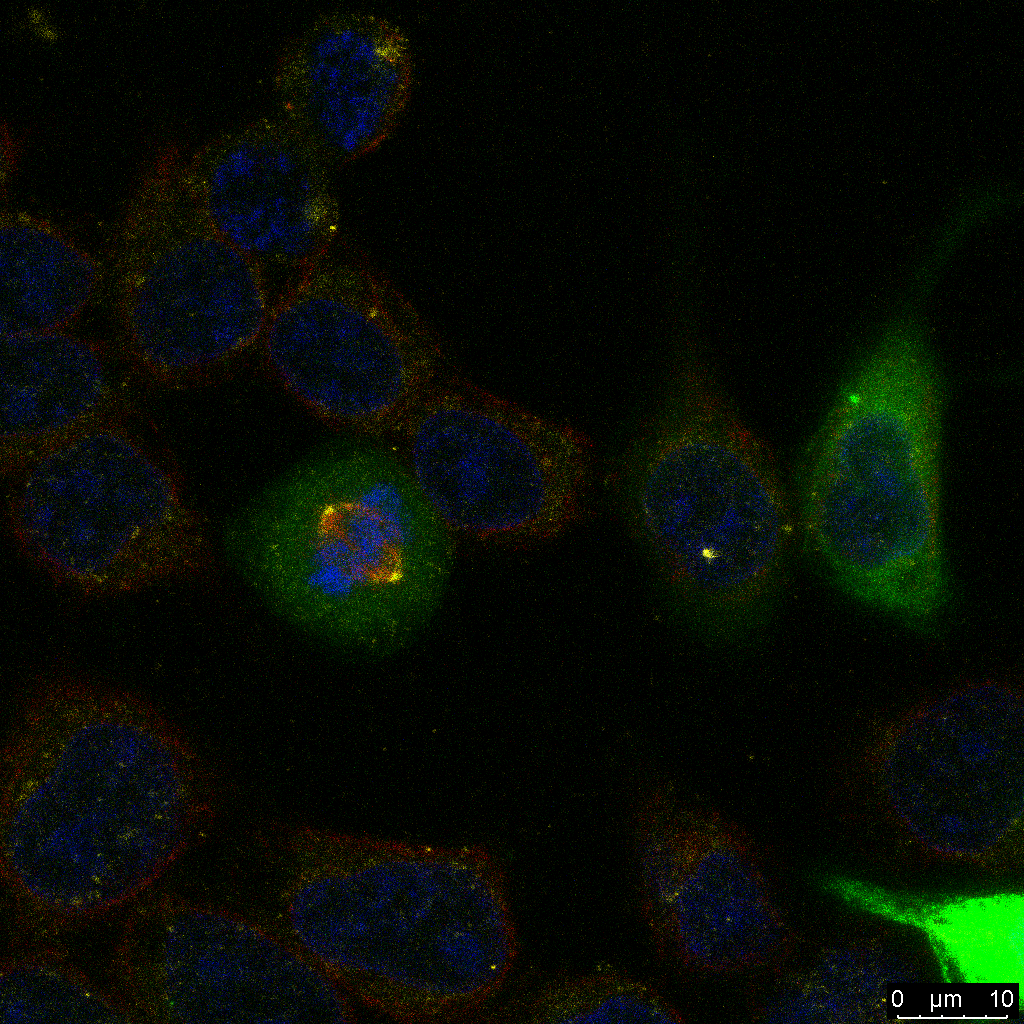

Supplement: Supplementary file 4 — Source data Fig. 2 [file 44319_2025_438_MOESM4_ESM.zip › SD figure 2/Fig. 2N/H215-610-0.45.tif]

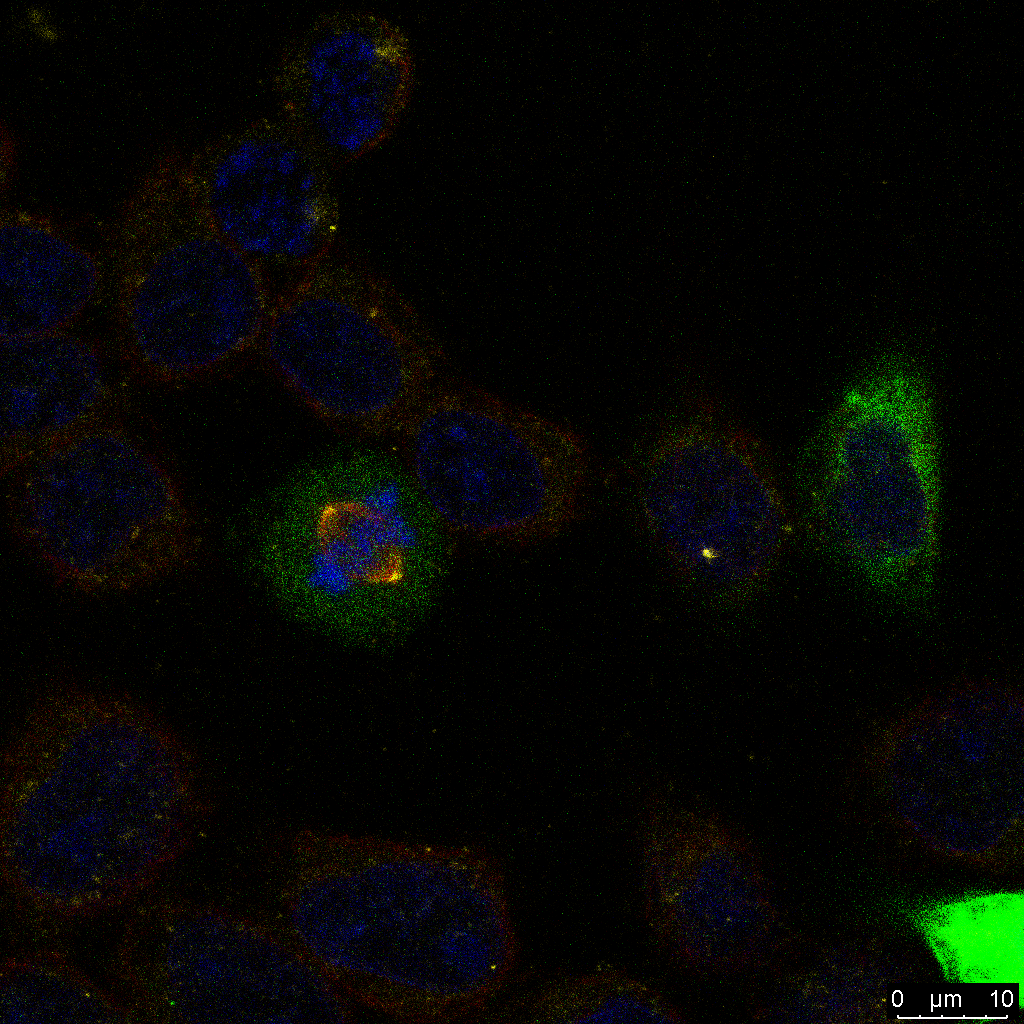

Supplement: Supplementary file 4 — Source data Fig. 2 [file 44319_2025_438_MOESM4_ESM.zip › SD figure 2/Fig. 2N/H215-610-0.9.tif]

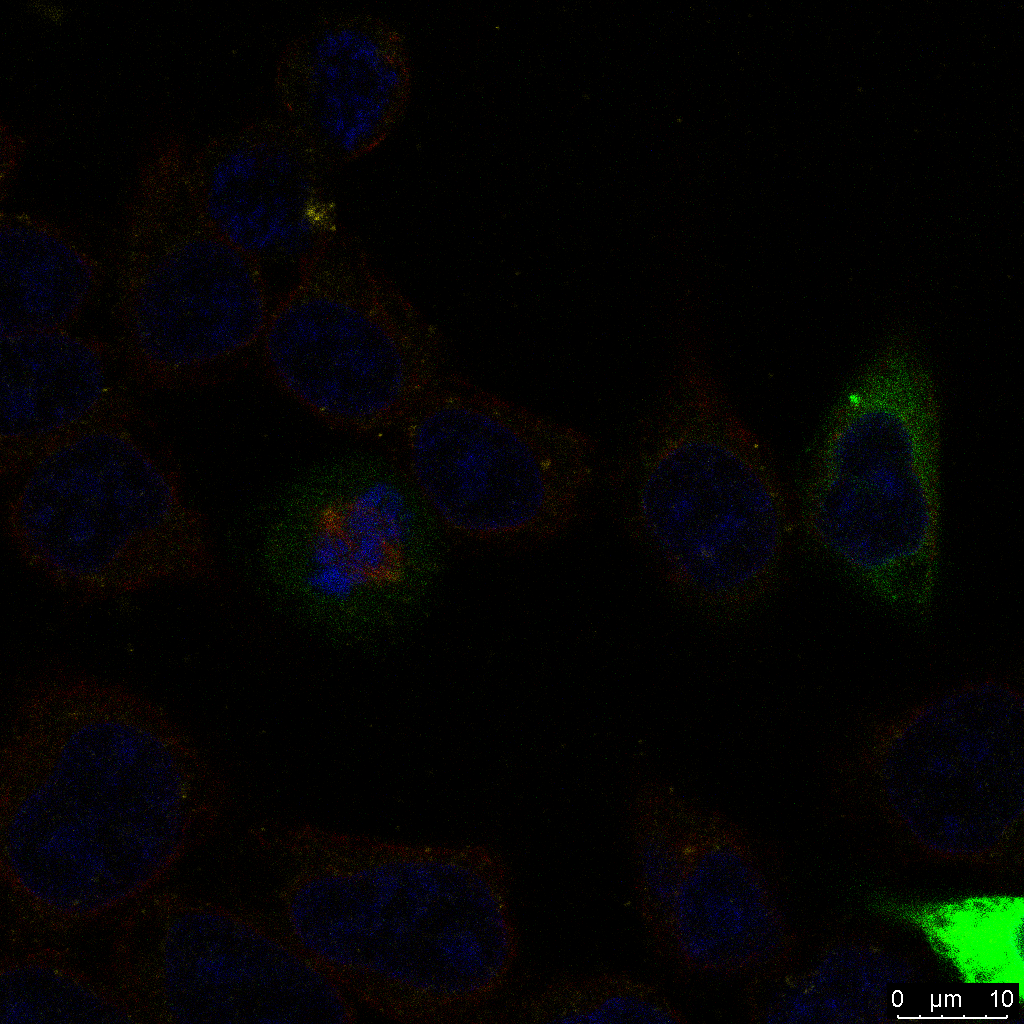

Supplement: Supplementary file 4 — Source data Fig. 2 [file 44319_2025_438_MOESM4_ESM.zip › SD figure 2/Fig. 2N/H215-610-0.tif]

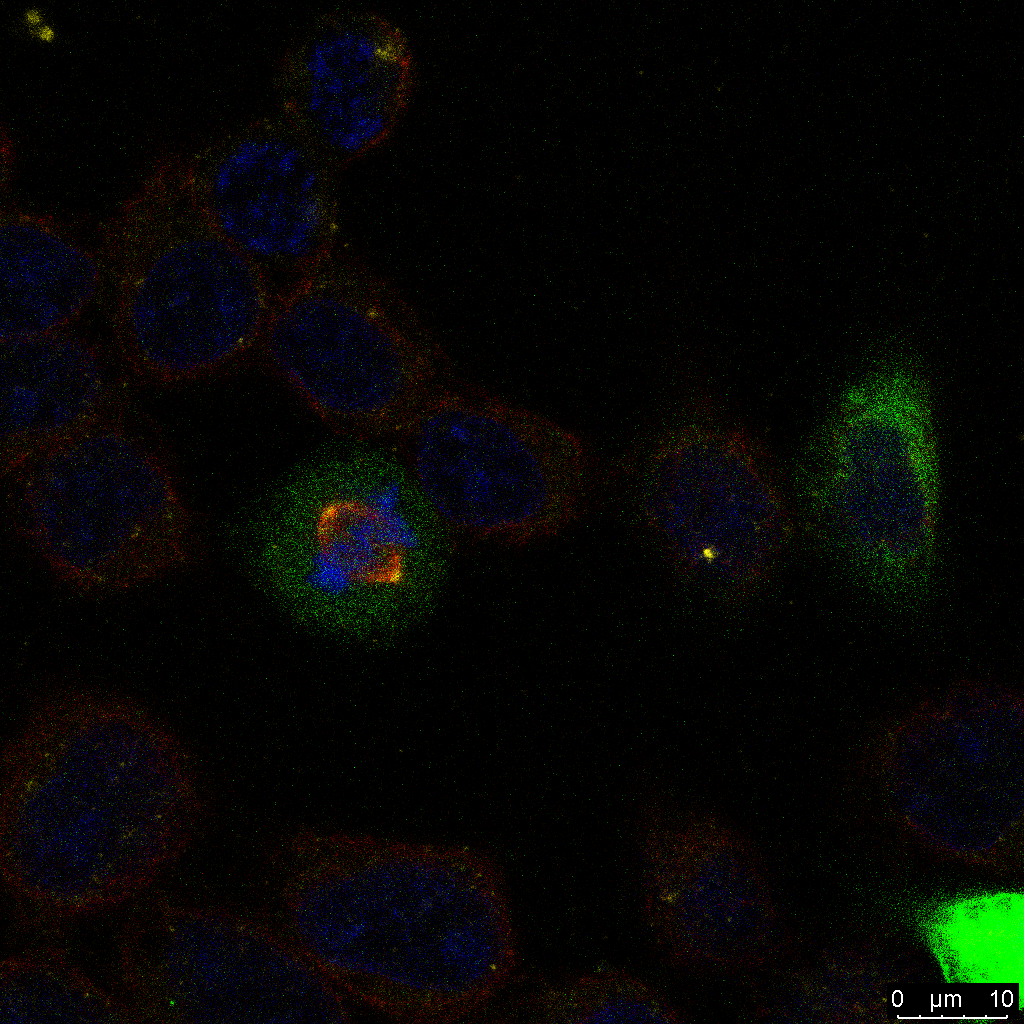

Supplement: Supplementary file 4 — Source data Fig. 2 [file 44319_2025_438_MOESM4_ESM.zip › SD figure 2/Fig. 2N/H215-610-1.35.tif]

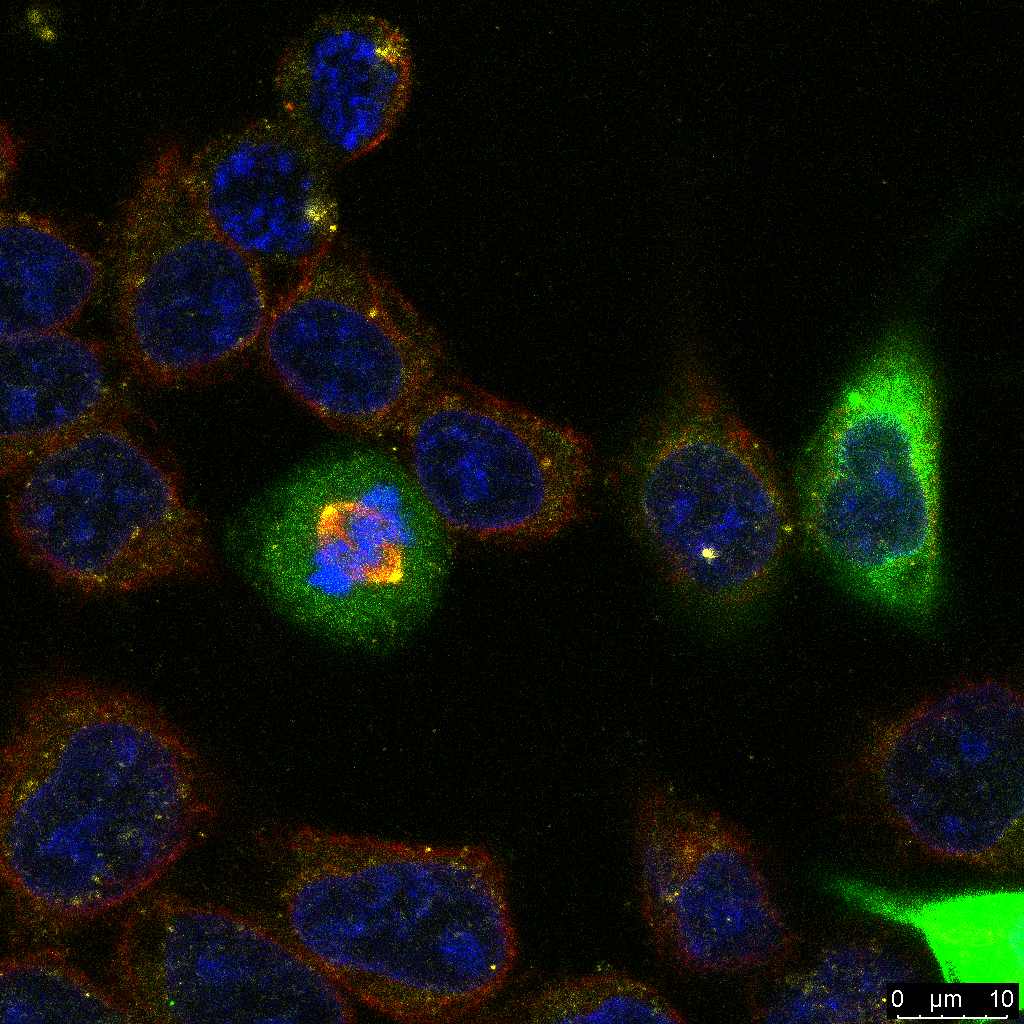

Supplement: Supplementary file 4 — Source data Fig. 2 [file 44319_2025_438_MOESM4_ESM.zip › SD figure 2/Fig. 2N/H215-610A-3D.tif]

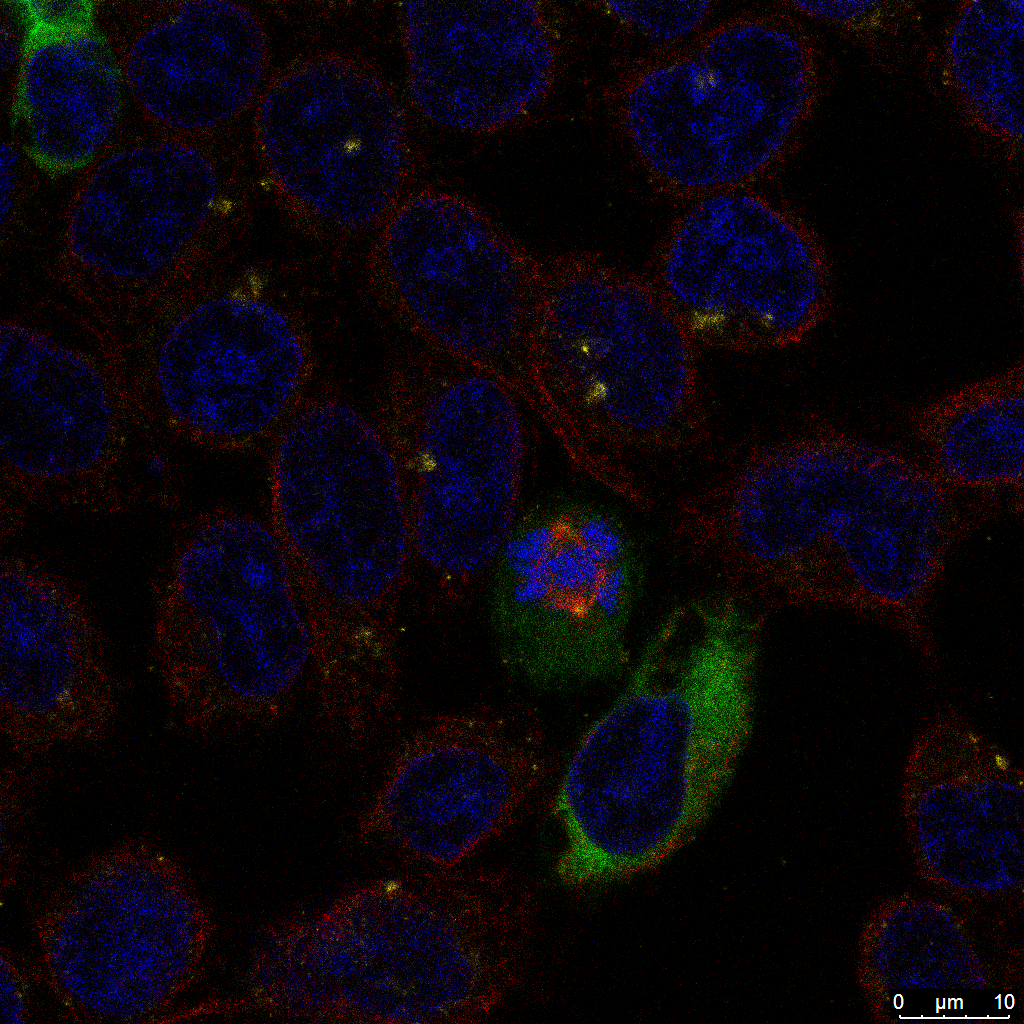

Supplement: Supplementary file 4 — Source data Fig. 2 [file 44319_2025_438_MOESM4_ESM.zip › SD figure 2/Fig. 2N/H215A-0.45.tif]

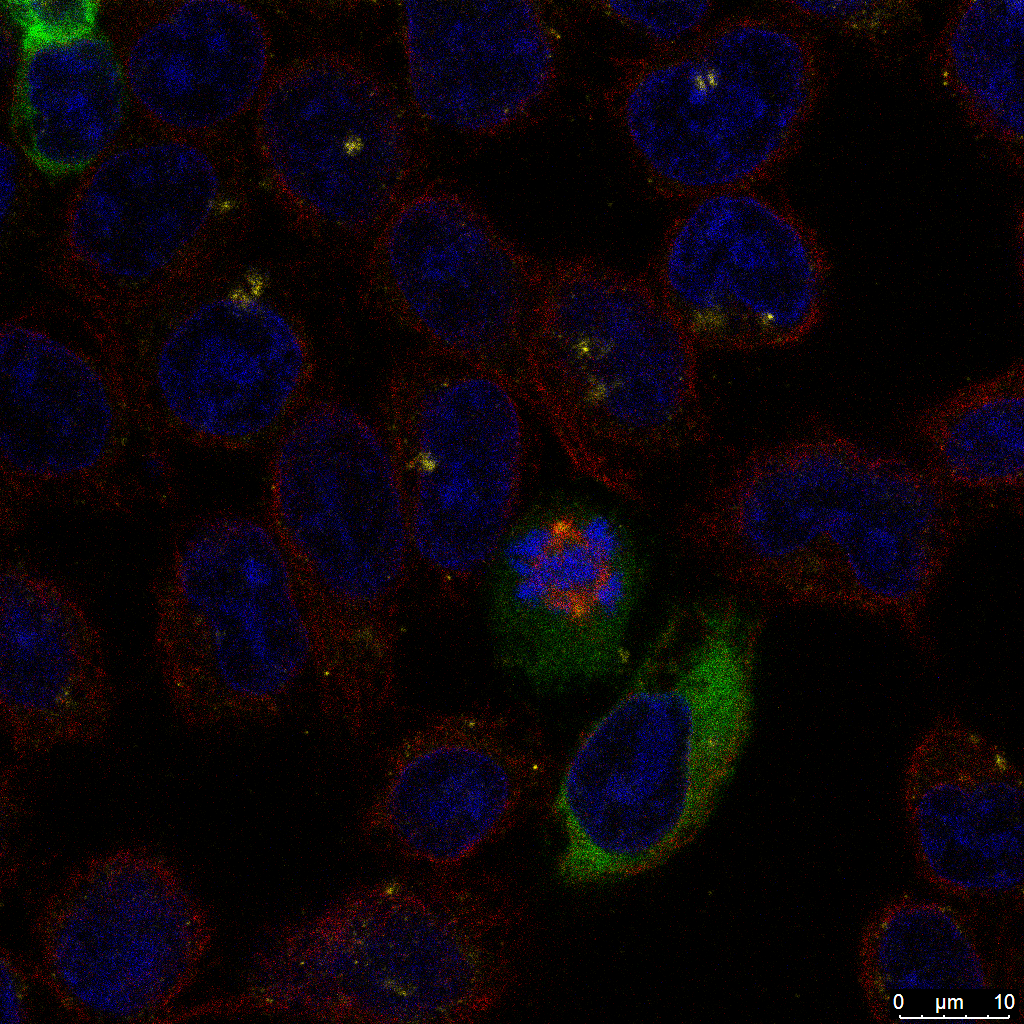

Supplement: Supplementary file 4 — Source data Fig. 2 [file 44319_2025_438_MOESM4_ESM.zip › SD figure 2/Fig. 2N/H215A-0.9.tif]

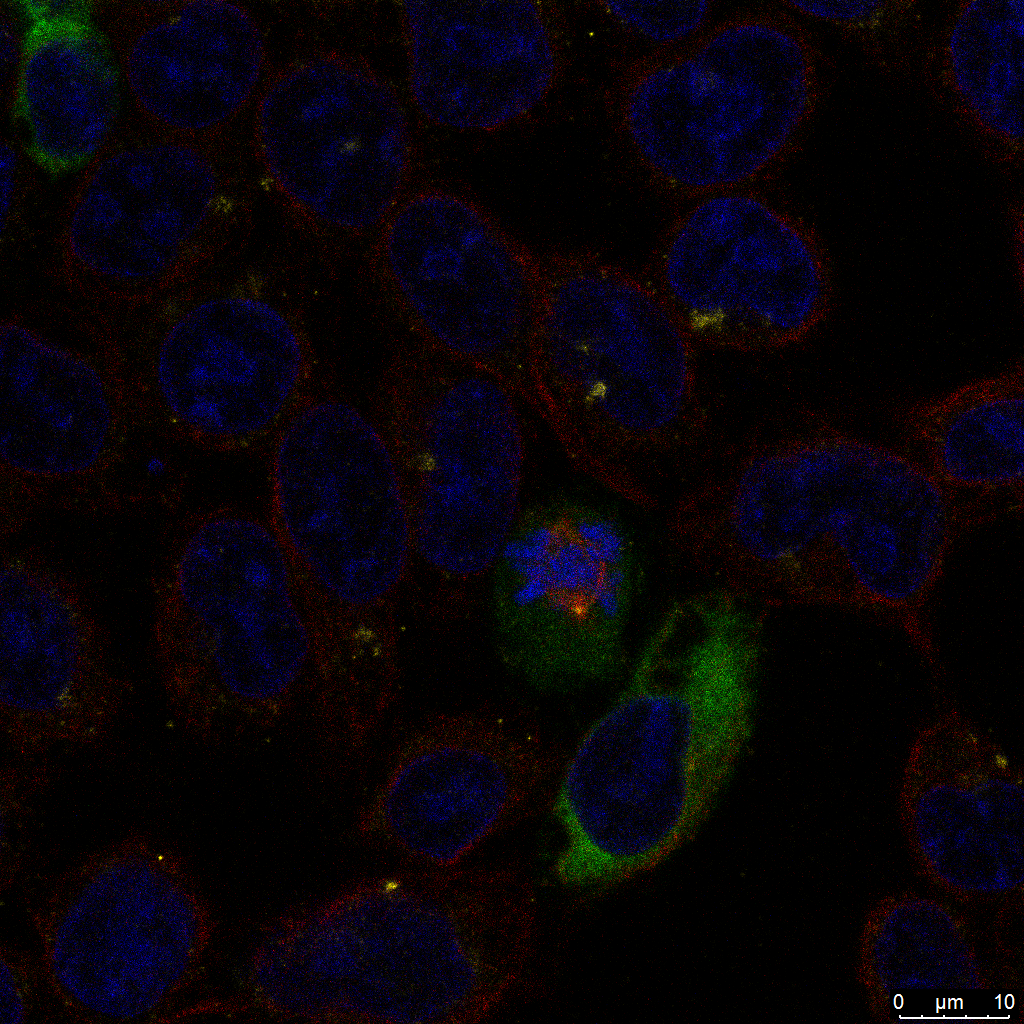

Supplement: Supplementary file 4 — Source data Fig. 2 [file 44319_2025_438_MOESM4_ESM.zip › SD figure 2/Fig. 2N/H215A-0.tif]

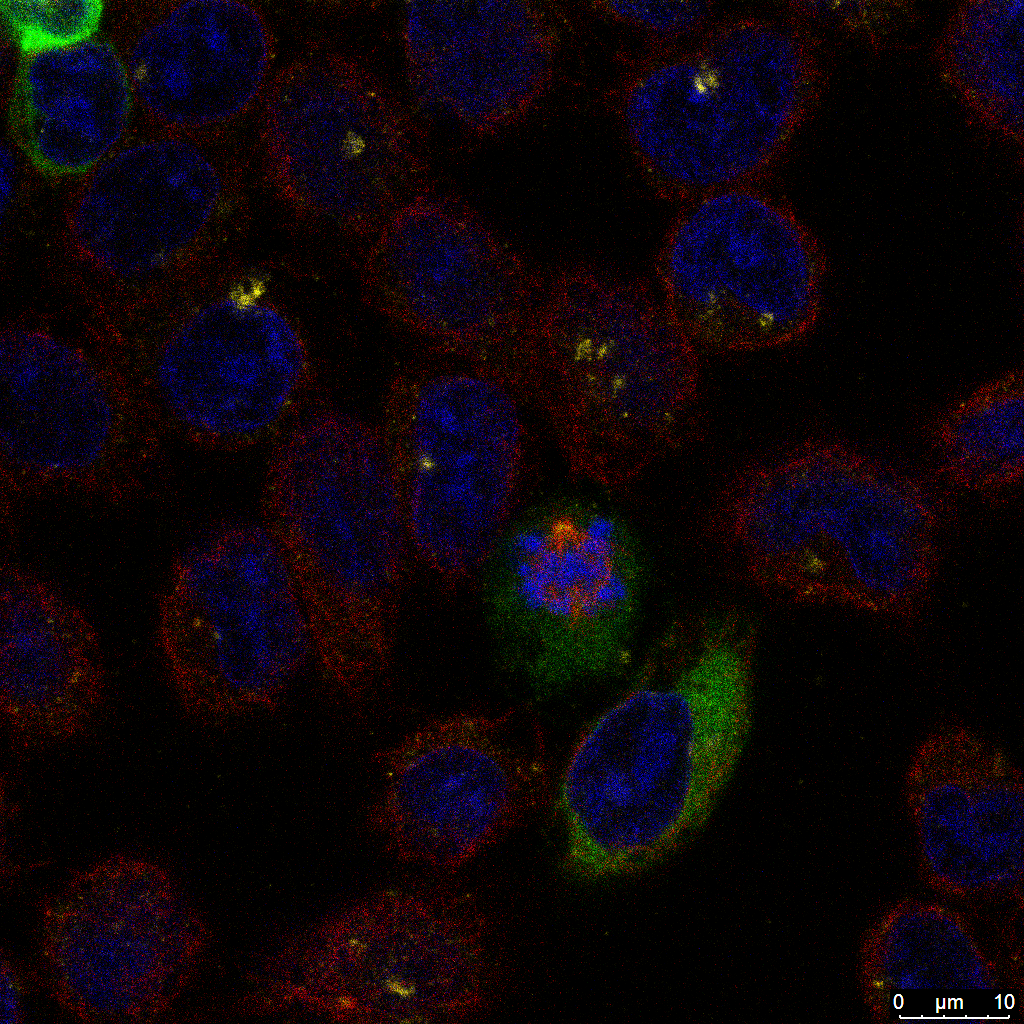

Supplement: Supplementary file 4 — Source data Fig. 2 [file 44319_2025_438_MOESM4_ESM.zip › SD figure 2/Fig. 2N/H215A-1.35.tif]

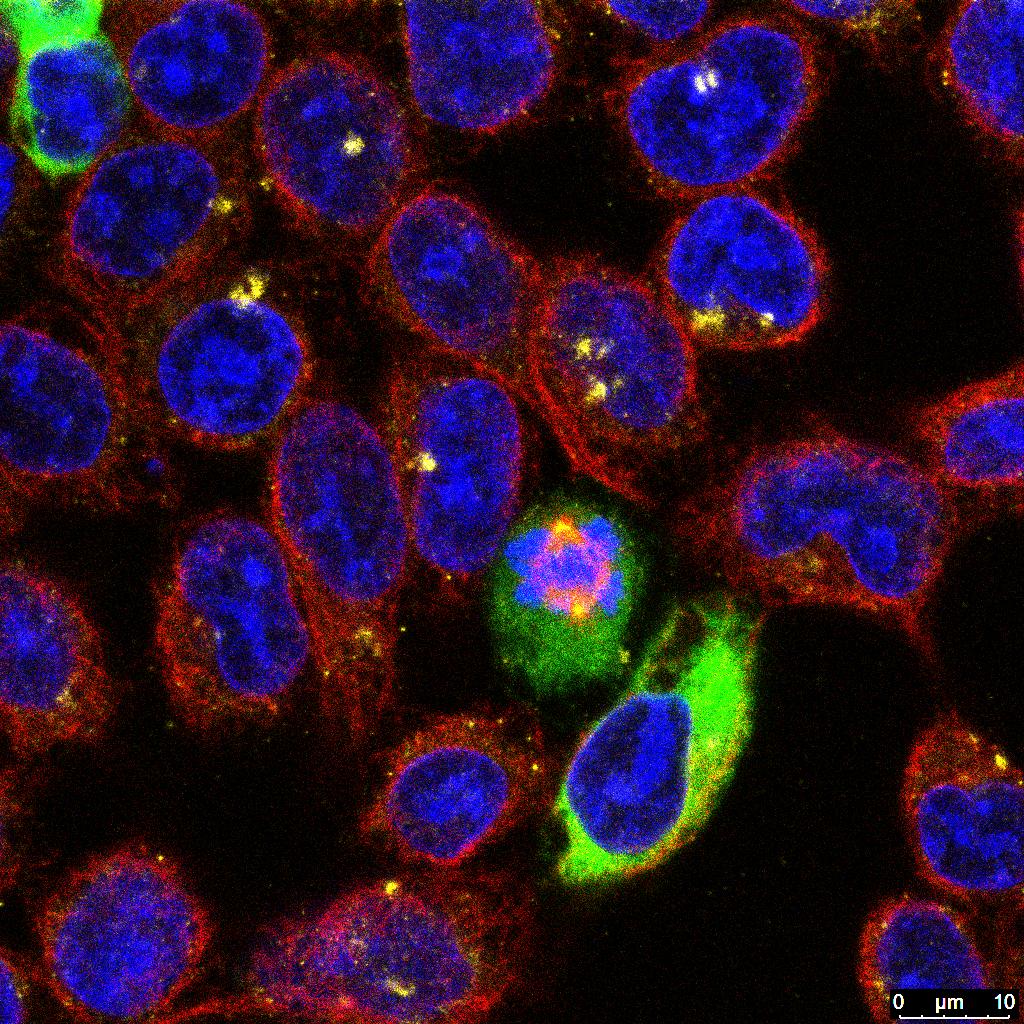

Supplement: Supplementary file 4 — Source data Fig. 2 [file 44319_2025_438_MOESM4_ESM.zip › SD figure 2/Fig. 2N/H215A-3D.tif]

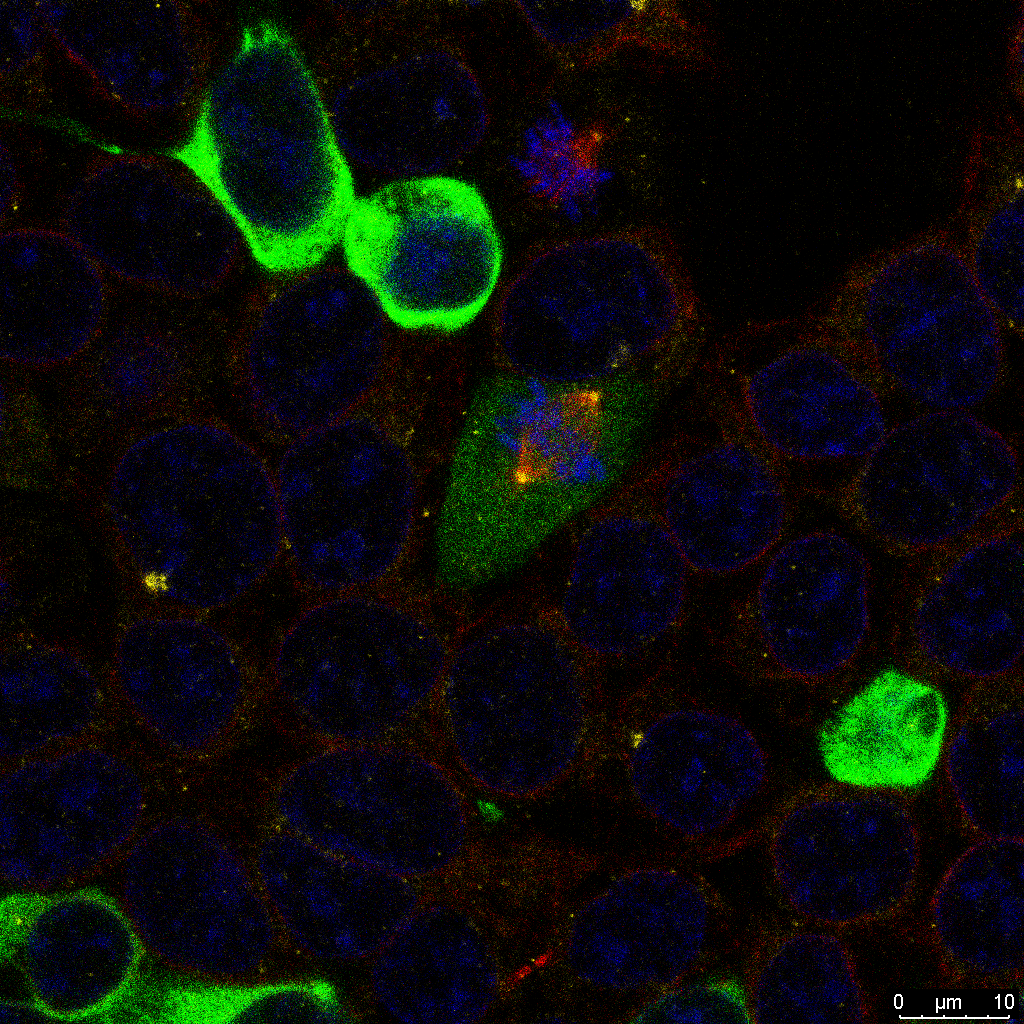

Supplement: Supplementary file 4 — Source data Fig. 2 [file 44319_2025_438_MOESM4_ESM.zip › SD figure 2/Fig. 2N/H610A-0.45.tif]

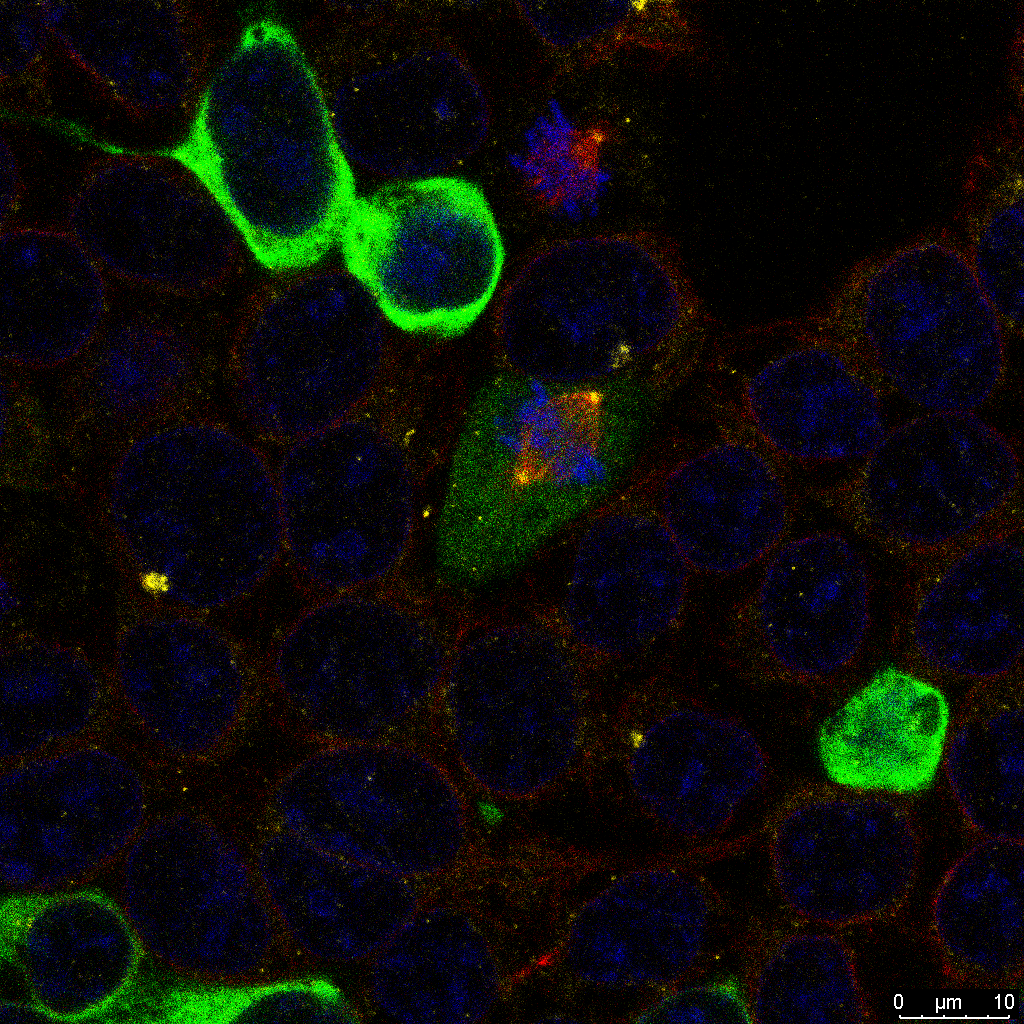

Supplement: Supplementary file 4 — Source data Fig. 2 [file 44319_2025_438_MOESM4_ESM.zip › SD figure 2/Fig. 2N/H610A-0.9.tif]

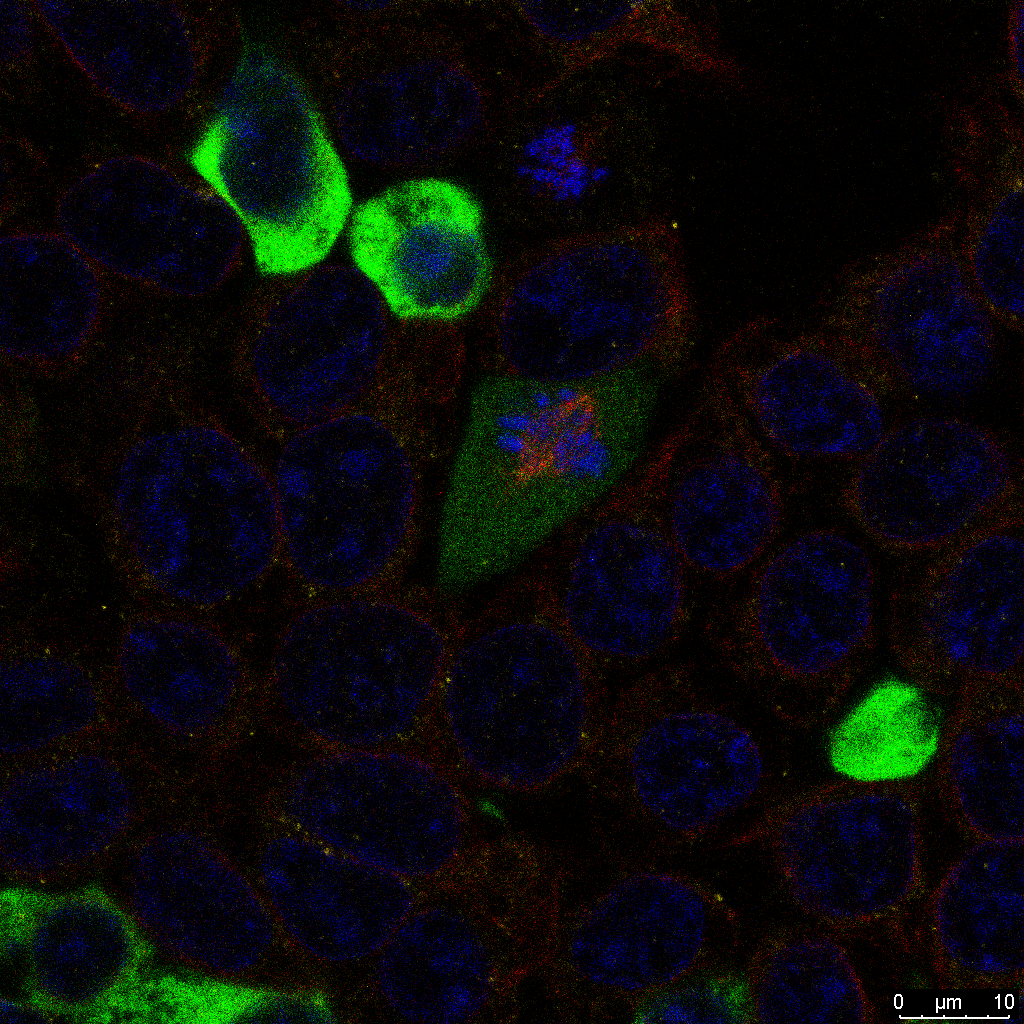

Supplement: Supplementary file 4 — Source data Fig. 2 [file 44319_2025_438_MOESM4_ESM.zip › SD figure 2/Fig. 2N/H610A-0.tif]

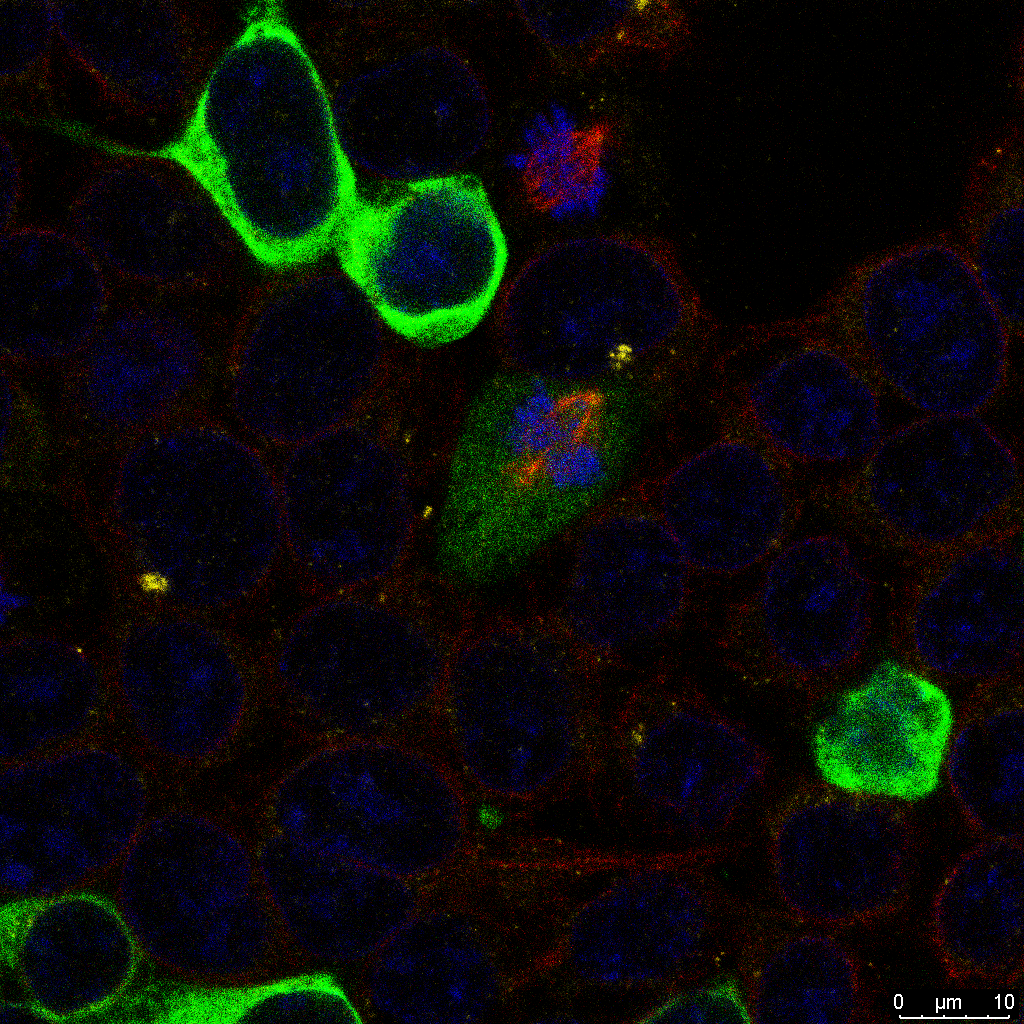

Supplement: Supplementary file 4 — Source data Fig. 2 [file 44319_2025_438_MOESM4_ESM.zip › SD figure 2/Fig. 2N/H610A-1.35.tif]

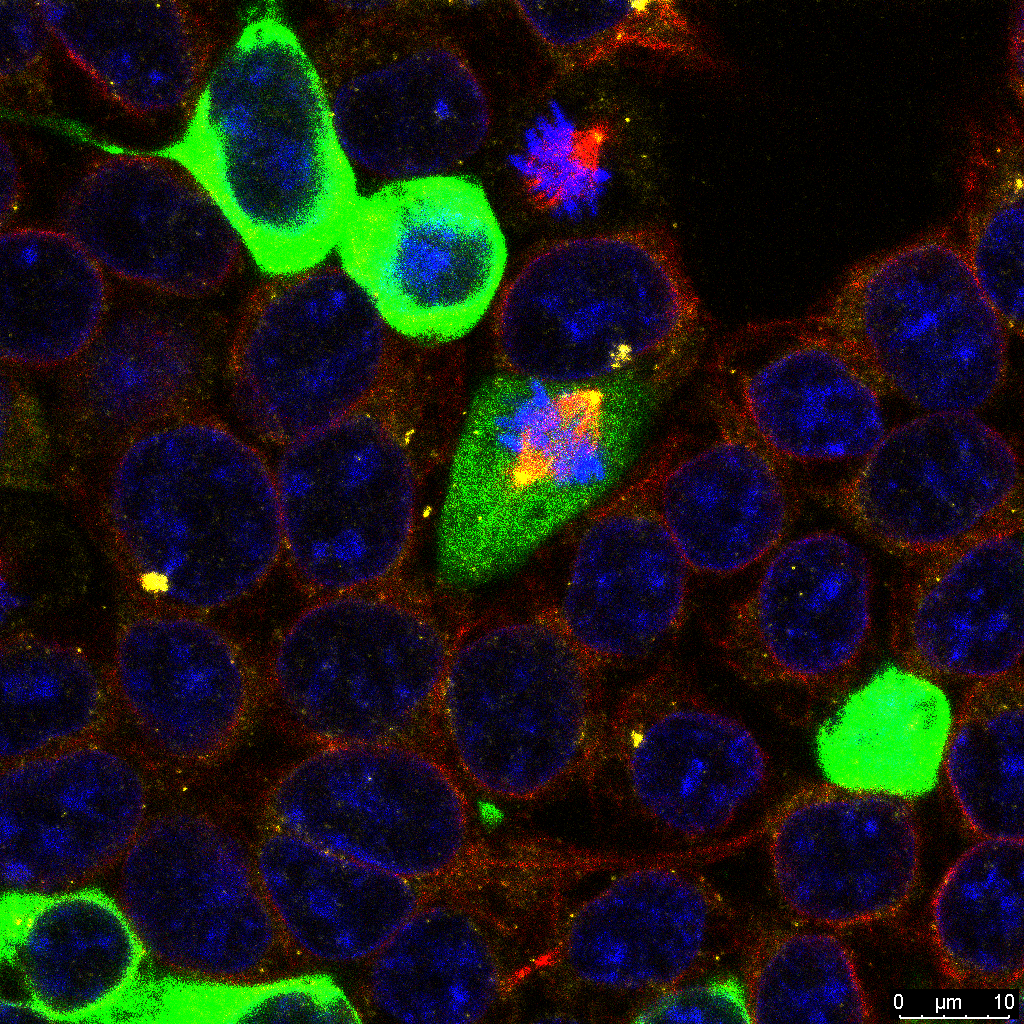

Supplement: Supplementary file 4 — Source data Fig. 2 [file 44319_2025_438_MOESM4_ESM.zip › SD figure 2/Fig. 2N/H610A-3D.tif]

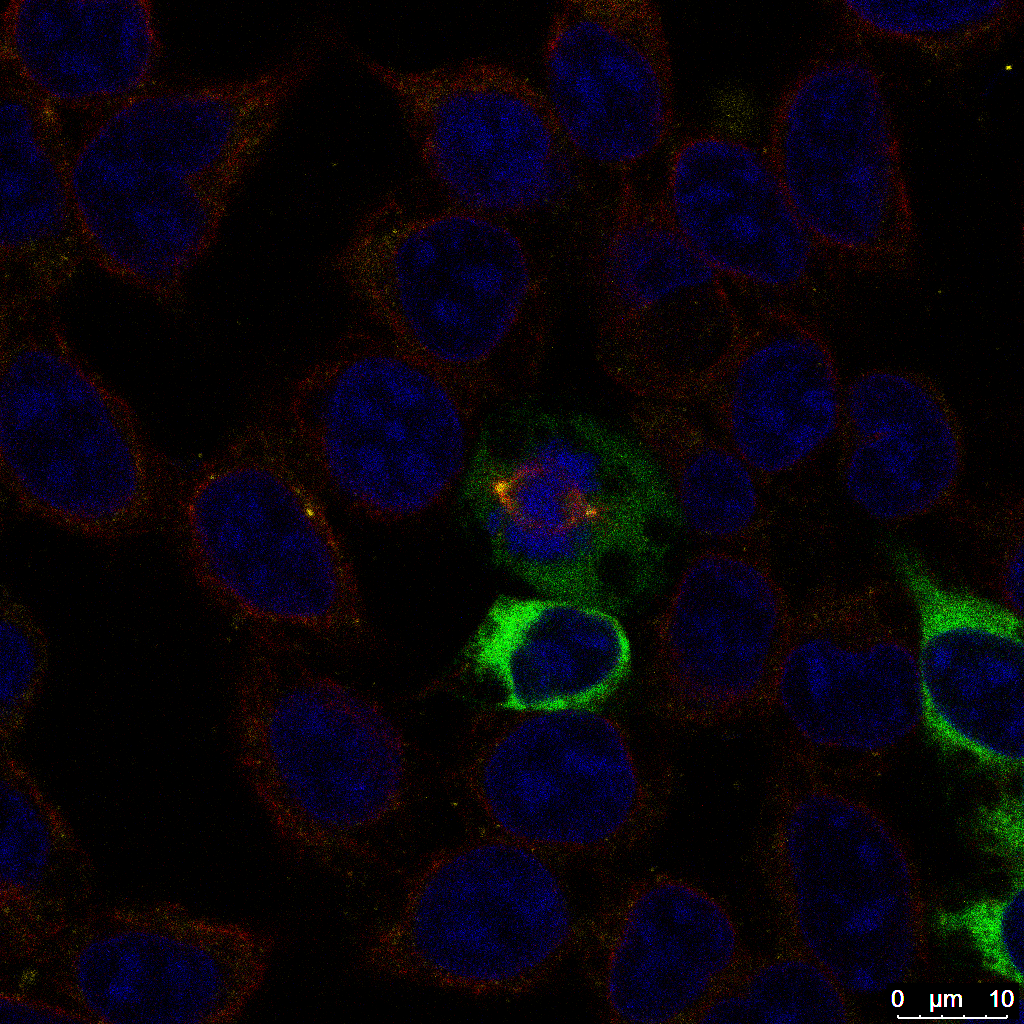

Supplement: Supplementary file 4 — Source data Fig. 2 [file 44319_2025_438_MOESM4_ESM.zip › SD figure 2/Fig. 2N/WT-0.45.tif]

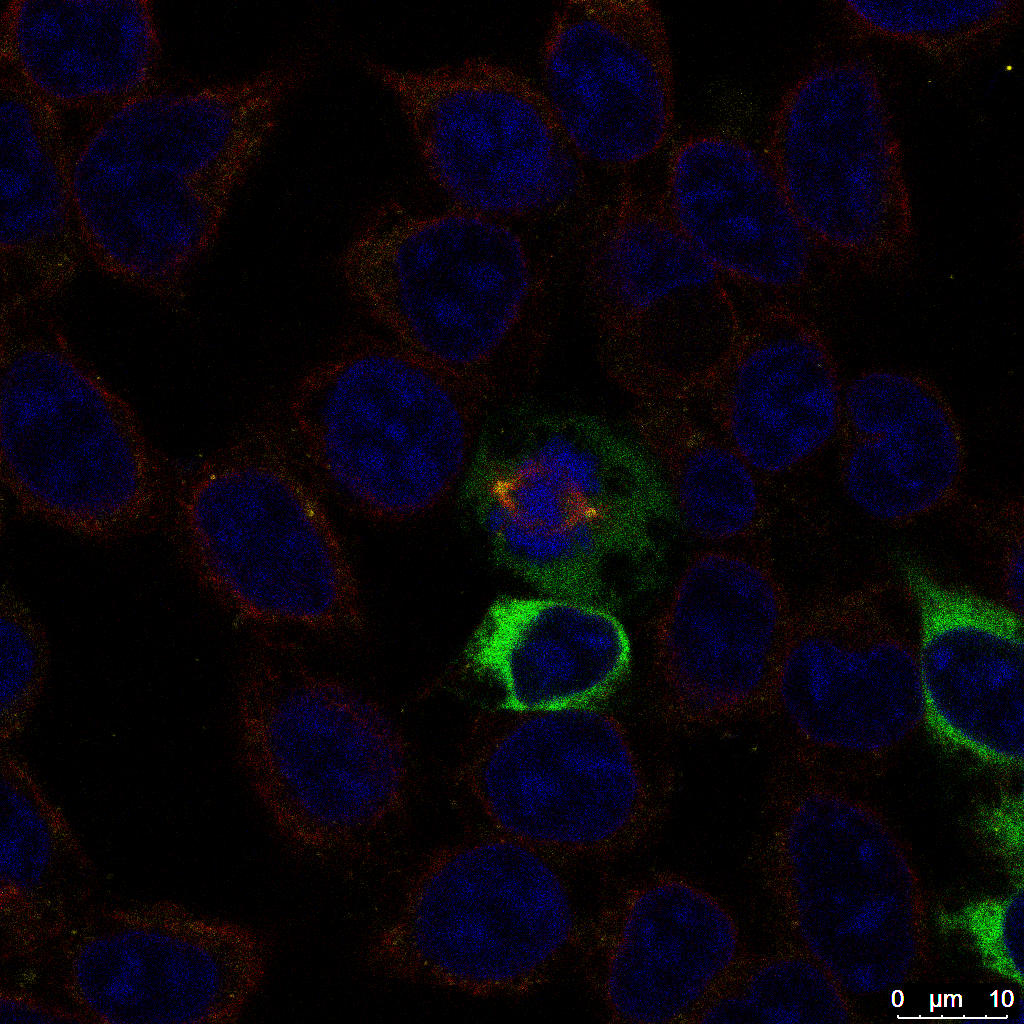

Supplement: Supplementary file 4 — Source data Fig. 2 [file 44319_2025_438_MOESM4_ESM.zip › SD figure 2/Fig. 2N/WT-0.9.tif]

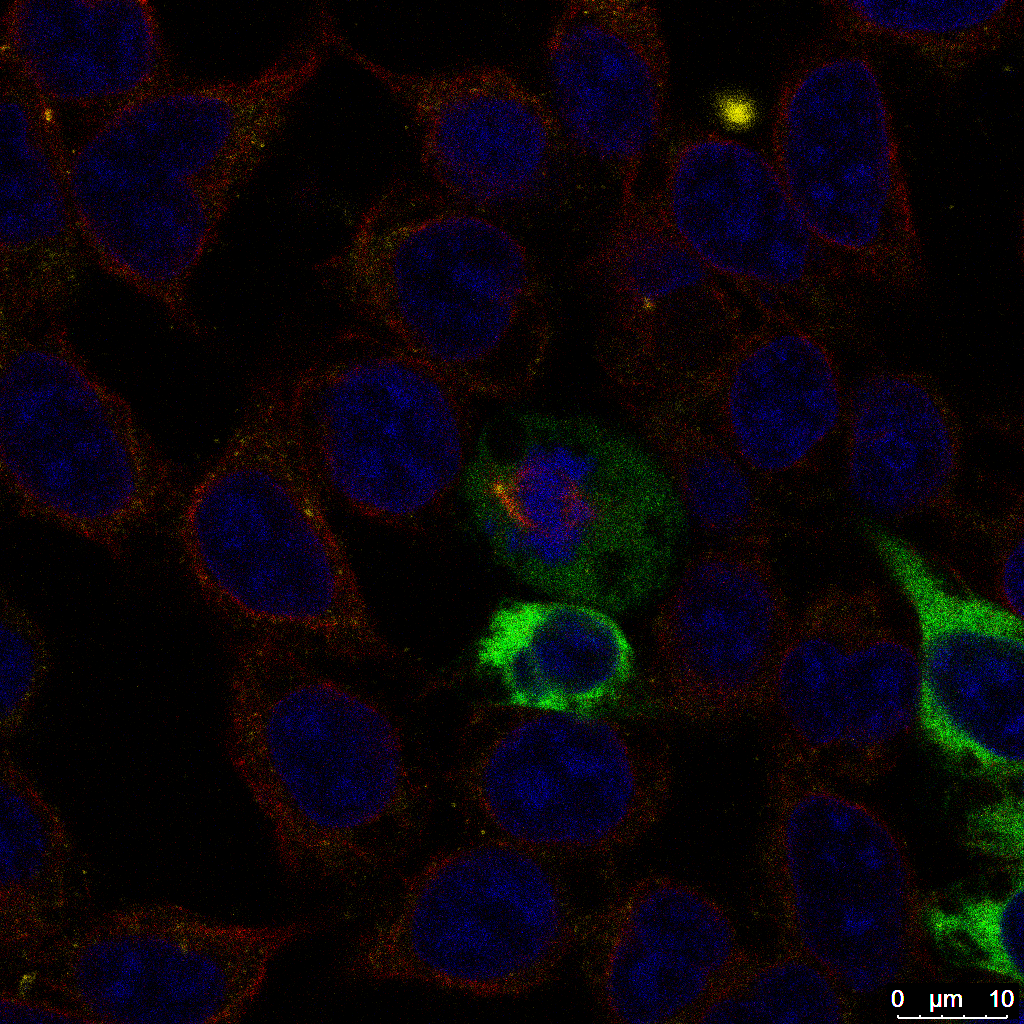

Supplement: Supplementary file 4 — Source data Fig. 2 [file 44319_2025_438_MOESM4_ESM.zip › SD figure 2/Fig. 2N/WT-0.tif]

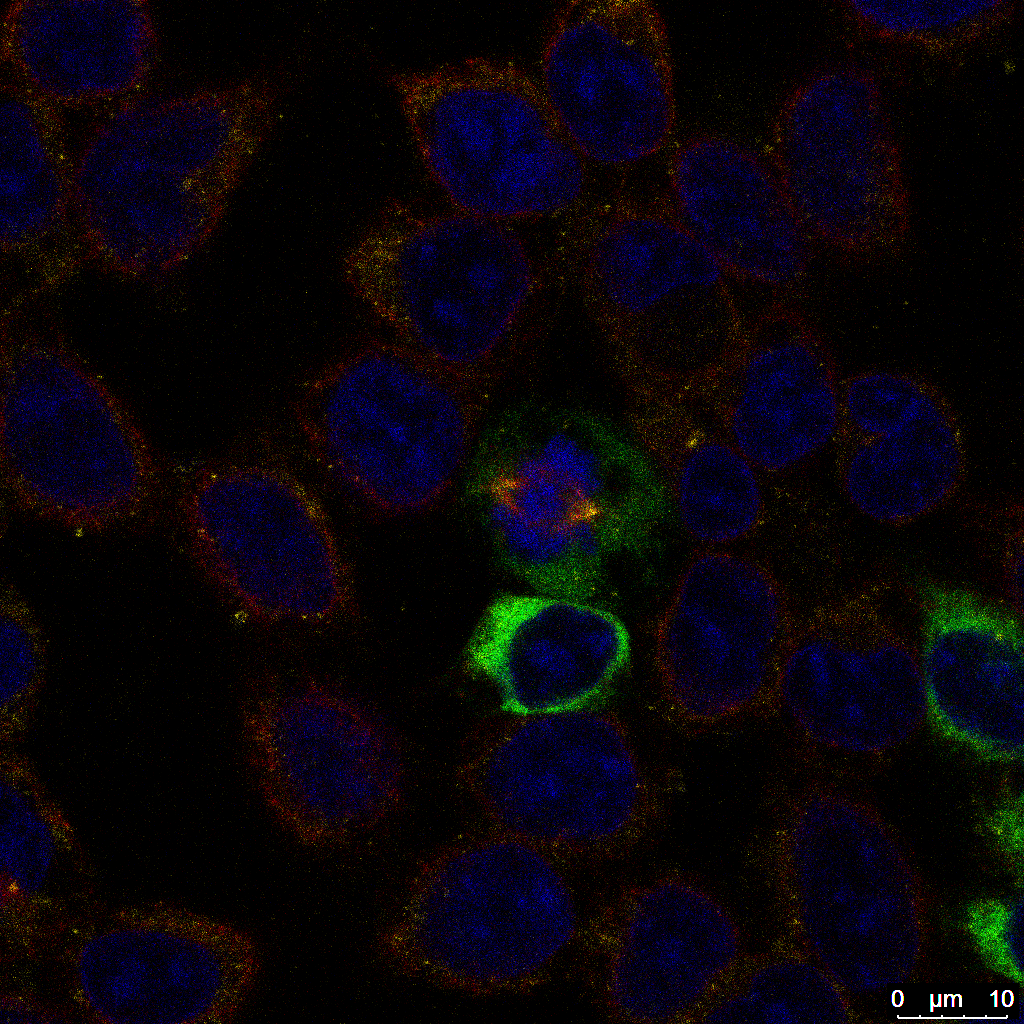

Supplement: Supplementary file 4 — Source data Fig. 2 [file 44319_2025_438_MOESM4_ESM.zip › SD figure 2/Fig. 2N/WT-1.35.tif]

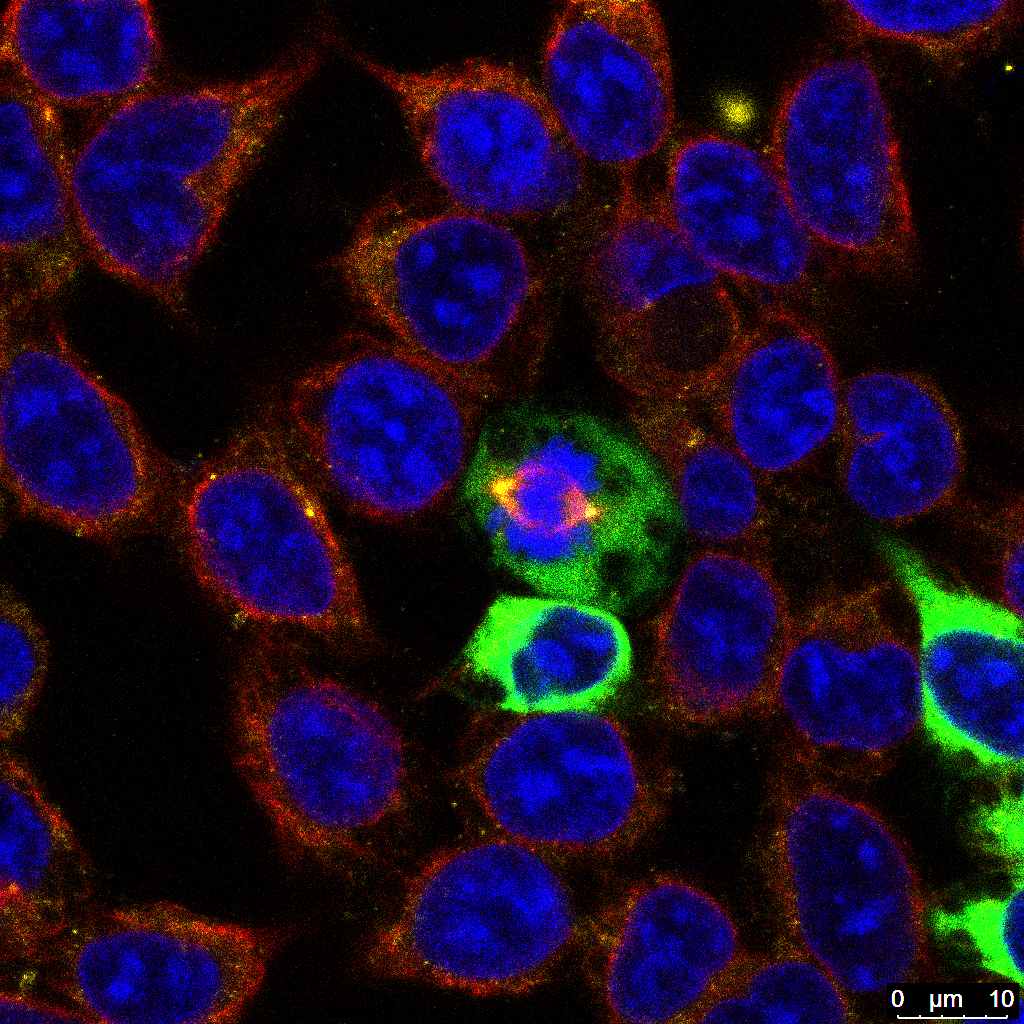

Supplement: Supplementary file 4 — Source data Fig. 2 [file 44319_2025_438_MOESM4_ESM.zip › SD figure 2/Fig. 2N/WT-3D.tif]

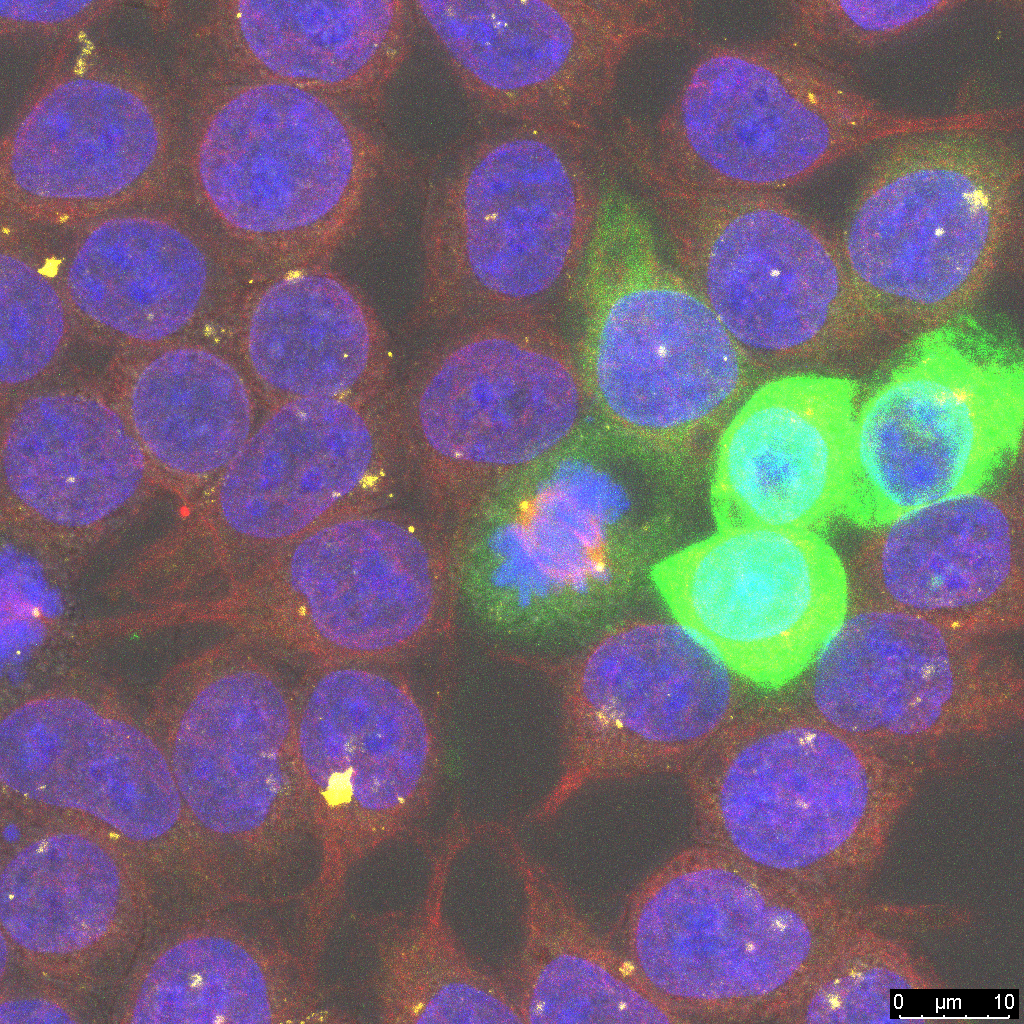

Supplement: Supplementary file 4 — Source data Fig. 2 [file 44319_2025_438_MOESM4_ESM.zip › SD figure 2/Fig. 2Q/H215-610A.tif]

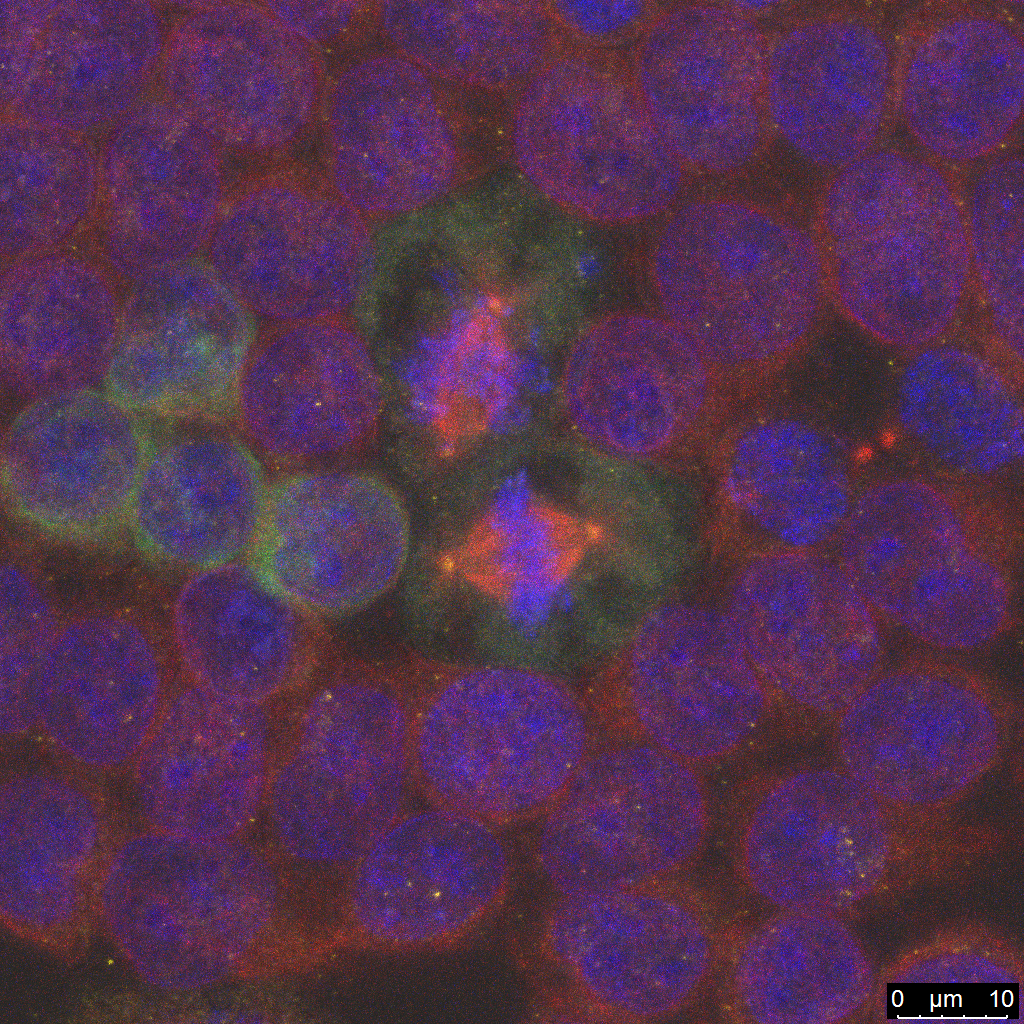

Supplement: Supplementary file 4 — Source data Fig. 2 [file 44319_2025_438_MOESM4_ESM.zip › SD figure 2/Fig. 2Q/H215A.tif]

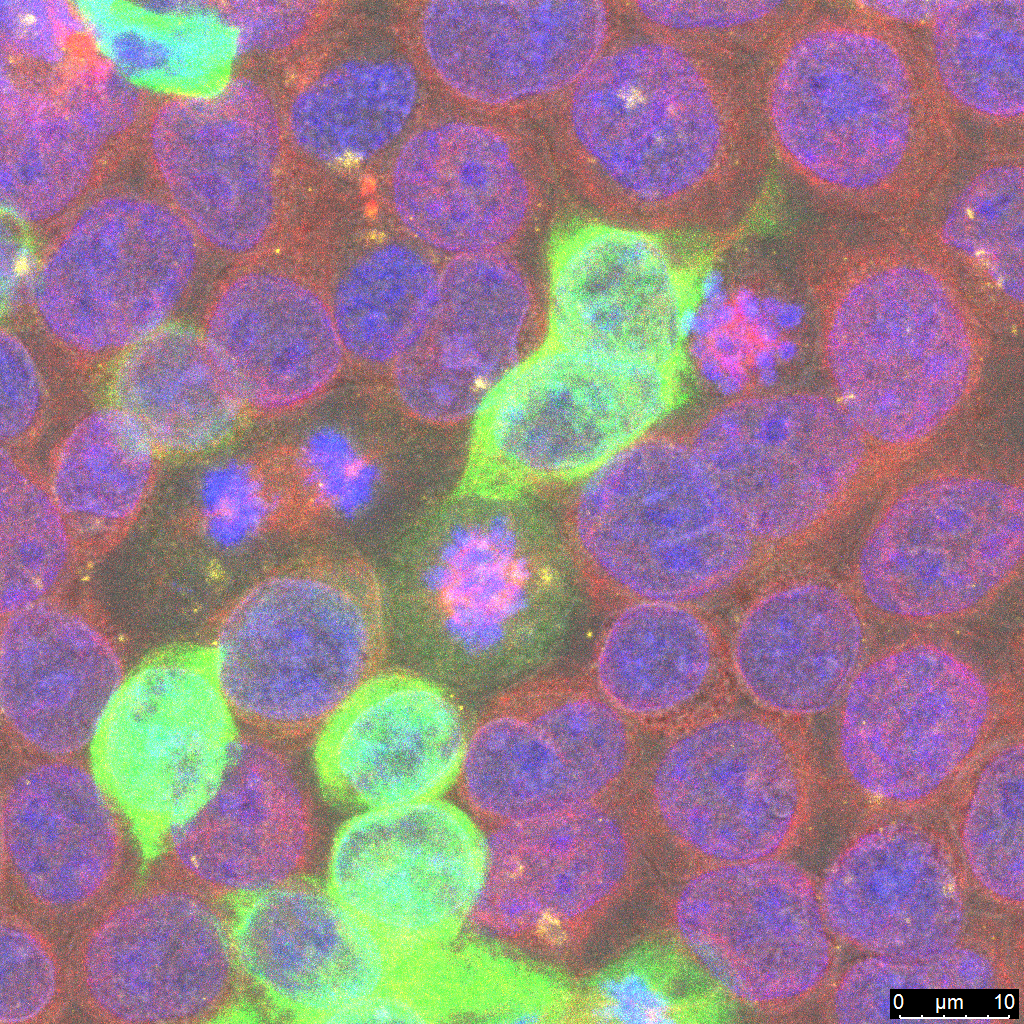

Supplement: Supplementary file 4 — Source data Fig. 2 [file 44319_2025_438_MOESM4_ESM.zip › SD figure 2/Fig. 2Q/H610A.tif]

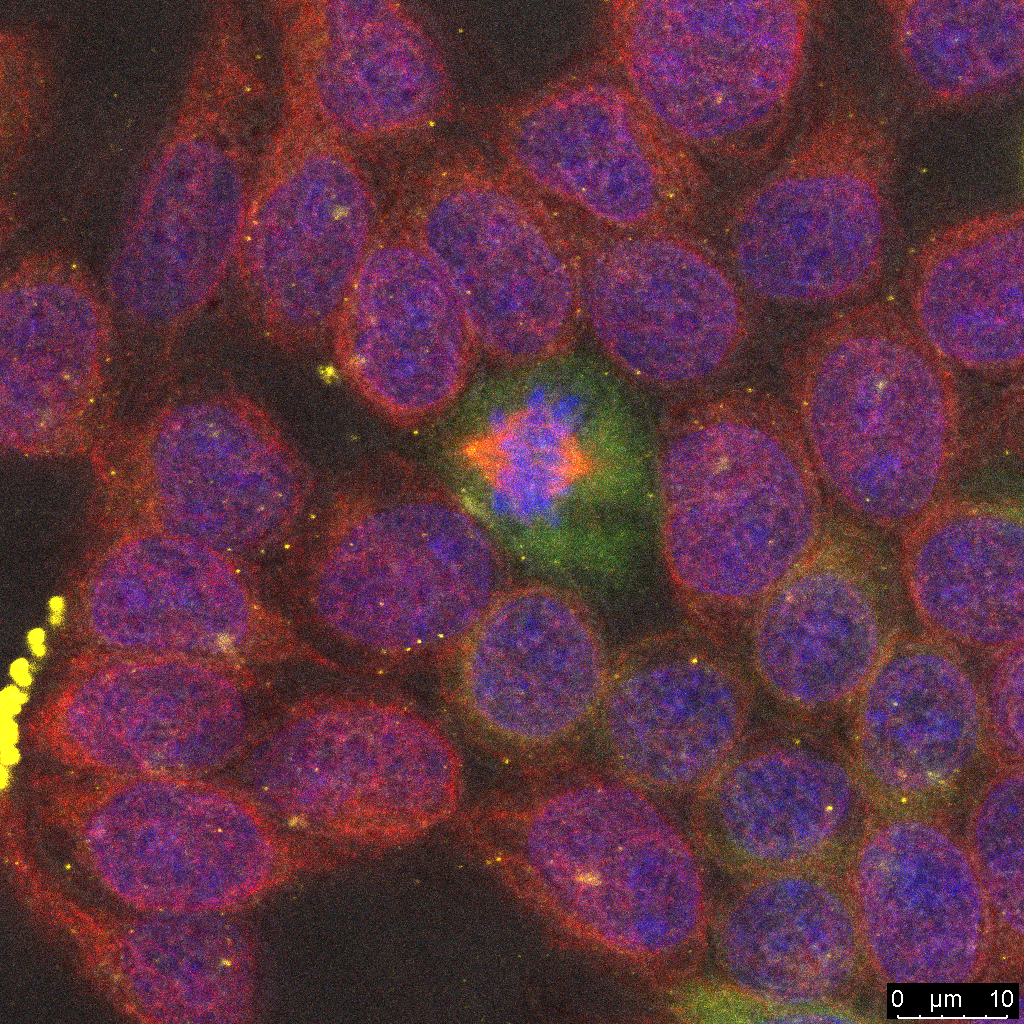

Supplement: Supplementary file 4 — Source data Fig. 2 [file 44319_2025_438_MOESM4_ESM.zip › SD figure 2/Fig. 2Q/WT.tif]

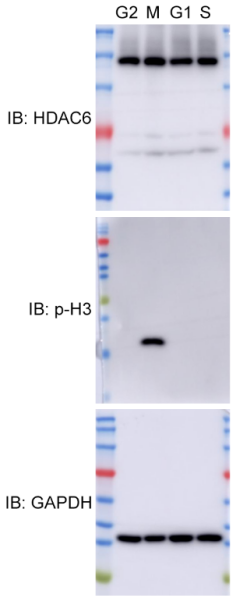

Supplement: Supplementary file 5 — Source data Fig. 3 [file 44319_2025_438_MOESM5_ESM.zip › SD figure 3/Fig. 3A/Fig. 3A.png]

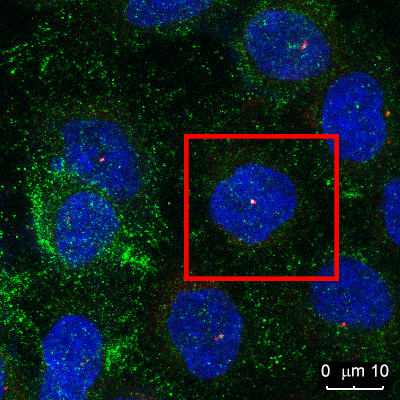

Supplement: Supplementary file 5 — Source data Fig. 3 [file 44319_2025_438_MOESM5_ESM.zip › SD figure 3/Fig. 3B/Interphase.tif]

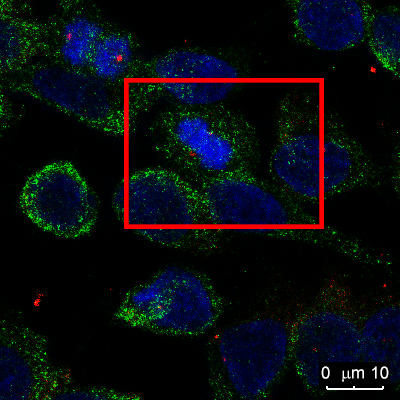

Supplement: Supplementary file 5 — Source data Fig. 3 [file 44319_2025_438_MOESM5_ESM.zip › SD figure 3/Fig. 3B/Metaphase.tif]

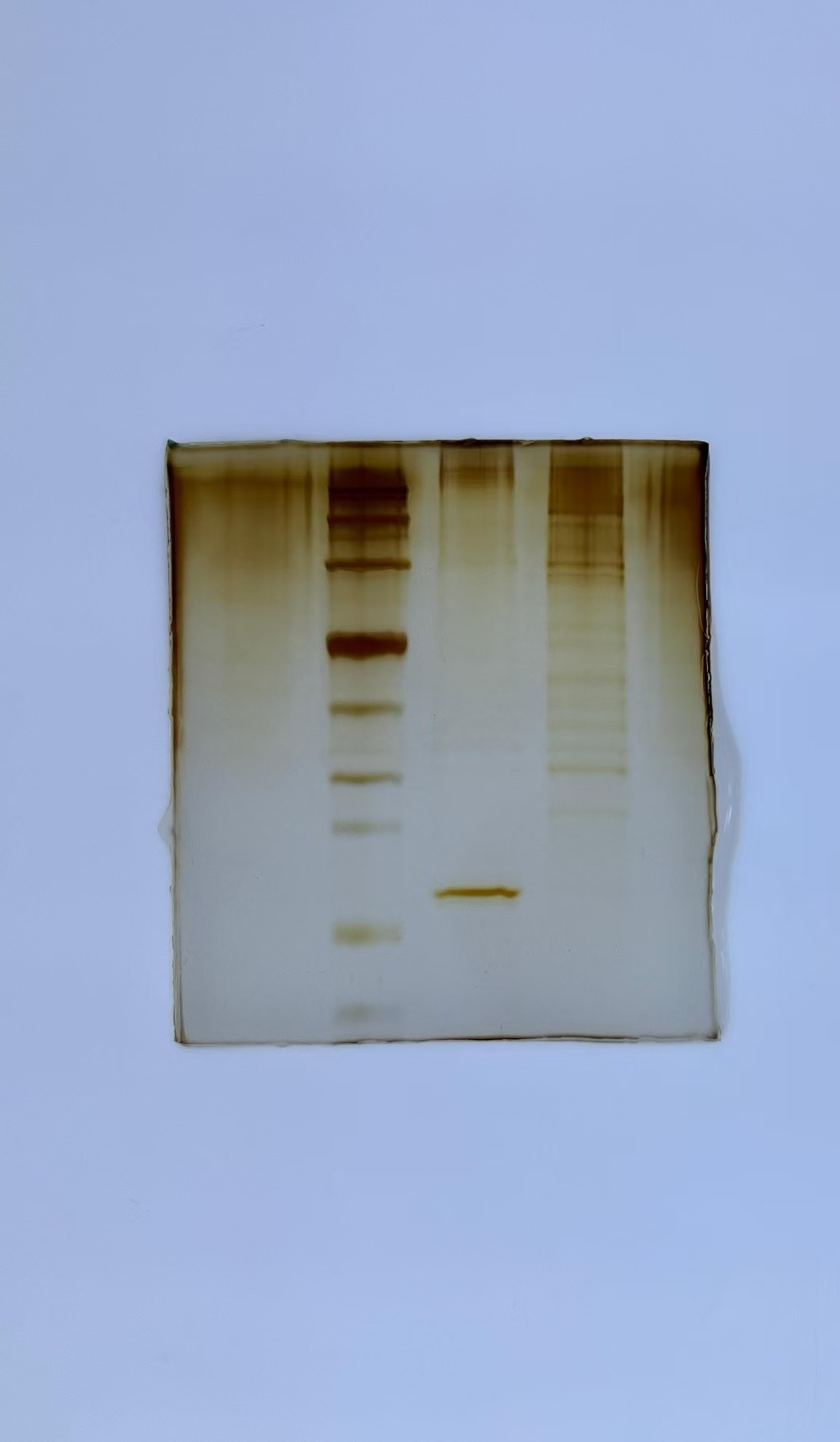

Supplement: Supplementary file 5 — Source data Fig. 3 [file 44319_2025_438_MOESM5_ESM.zip › SD figure 3/Fig. 3D/Fig. 3D.jpg]

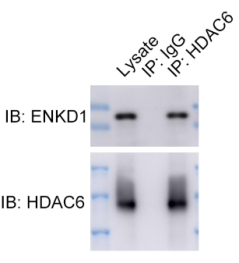

Supplement: Supplementary file 5 — Source data Fig. 3 [file 44319_2025_438_MOESM5_ESM.zip › SD figure 3/Fig. 3F/Fig. 3F.png]

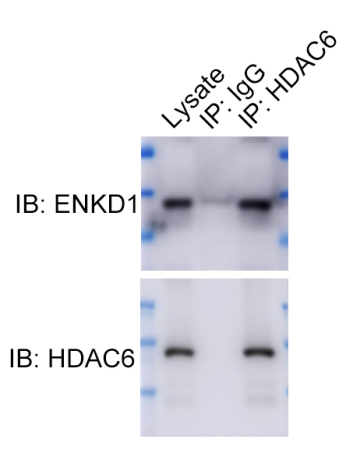

Supplement: Supplementary file 5 — Source data Fig. 3 [file 44319_2025_438_MOESM5_ESM.zip › SD figure 3/Fig. 3G/Fig. 3G.png]

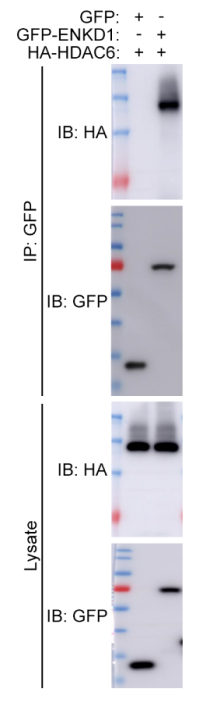

Supplement: Supplementary file 5 — Source data Fig. 3 [file 44319_2025_438_MOESM5_ESM.zip › SD figure 3/Fig. 3H/Fig. 3H.png]

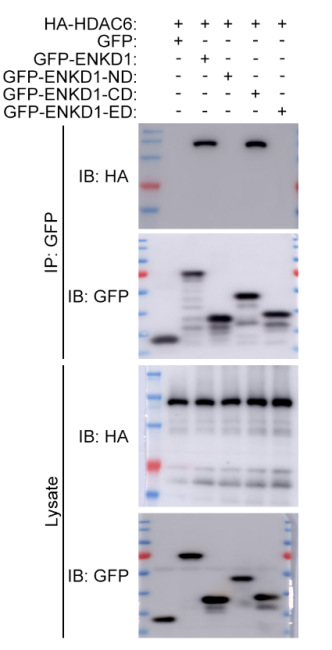

Supplement: Supplementary file 5 — Source data Fig. 3 [file 44319_2025_438_MOESM5_ESM.zip › SD figure 3/Fig. 3I/Fig. 3I.png]

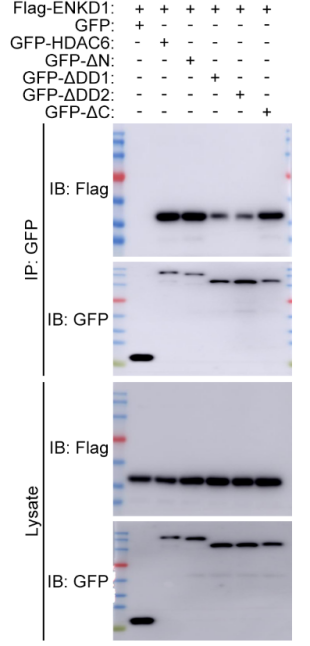

Supplement: Supplementary file 5 — Source data Fig. 3 [file 44319_2025_438_MOESM5_ESM.zip › SD figure 3/Fig. 3J/Fig. 3J.png]

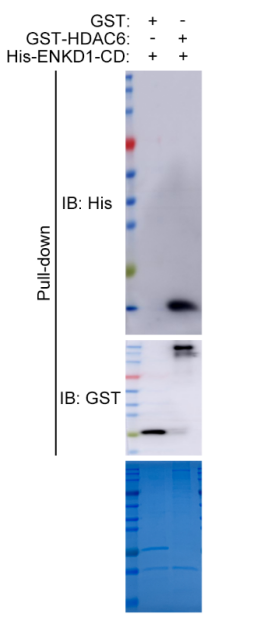

Supplement: Supplementary file 5 — Source data Fig. 3 [file 44319_2025_438_MOESM5_ESM.zip › SD figure 3/Fig. 3K/Fig. 3K.png]

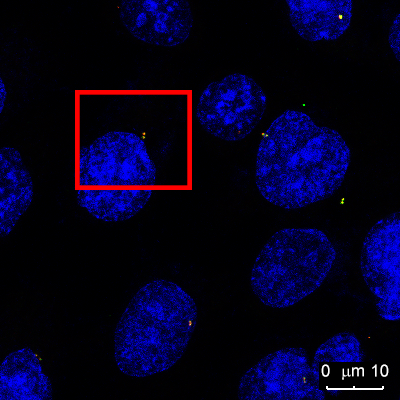

Supplement: Supplementary file 5 — Source data Fig. 3 [file 44319_2025_438_MOESM5_ESM.zip › SD figure 3/Fig. 3L/Interphase.tif]

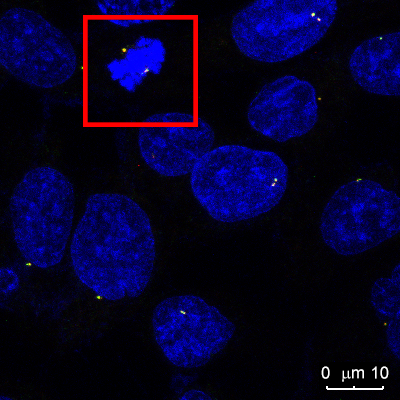

Supplement: Supplementary file 5 — Source data Fig. 3 [file 44319_2025_438_MOESM5_ESM.zip › SD figure 3/Fig. 3L/Metaphase.tif]

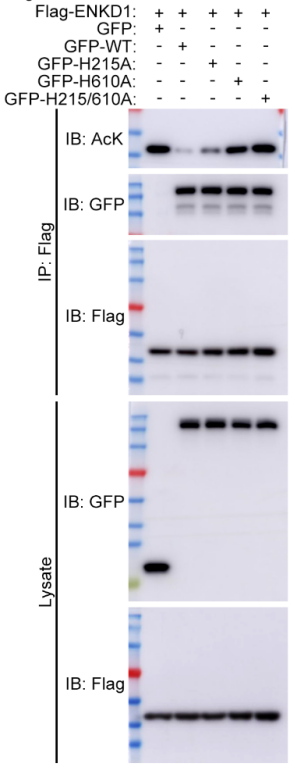

Supplement: Supplementary file 6 — Source data Fig. 4 [file 44319_2025_438_MOESM6_ESM.zip › SD figure 4/Fig. 4A/Fig. 4A.png]

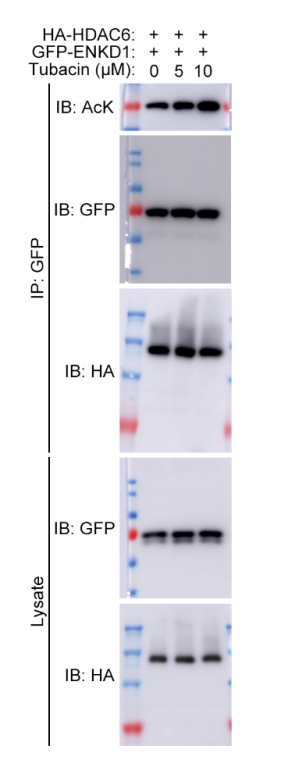

Supplement: Supplementary file 6 — Source data Fig. 4 [file 44319_2025_438_MOESM6_ESM.zip › SD figure 4/Fig. 4B/Fig. 4B.png]

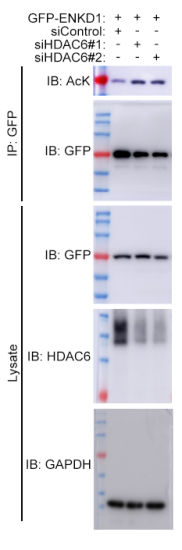

Supplement: Supplementary file 6 — Source data Fig. 4 [file 44319_2025_438_MOESM6_ESM.zip › SD figure 4/Fig. 4C/Fig. 4C.png]

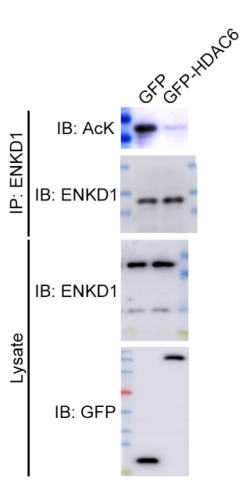

Supplement: Supplementary file 6 — Source data Fig. 4 [file 44319_2025_438_MOESM6_ESM.zip › SD figure 4/Fig. 4D/Fig. 4D.png]

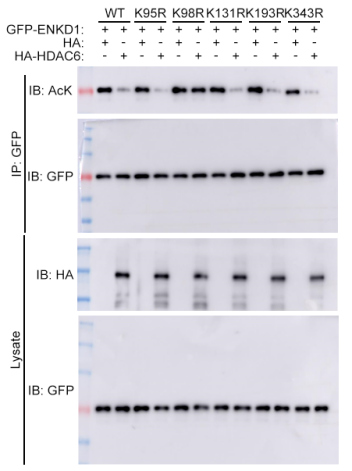

Supplement: Supplementary file 6 — Source data Fig. 4 [file 44319_2025_438_MOESM6_ESM.zip › SD figure 4/Fig. 4G/Fig. 4G.png]

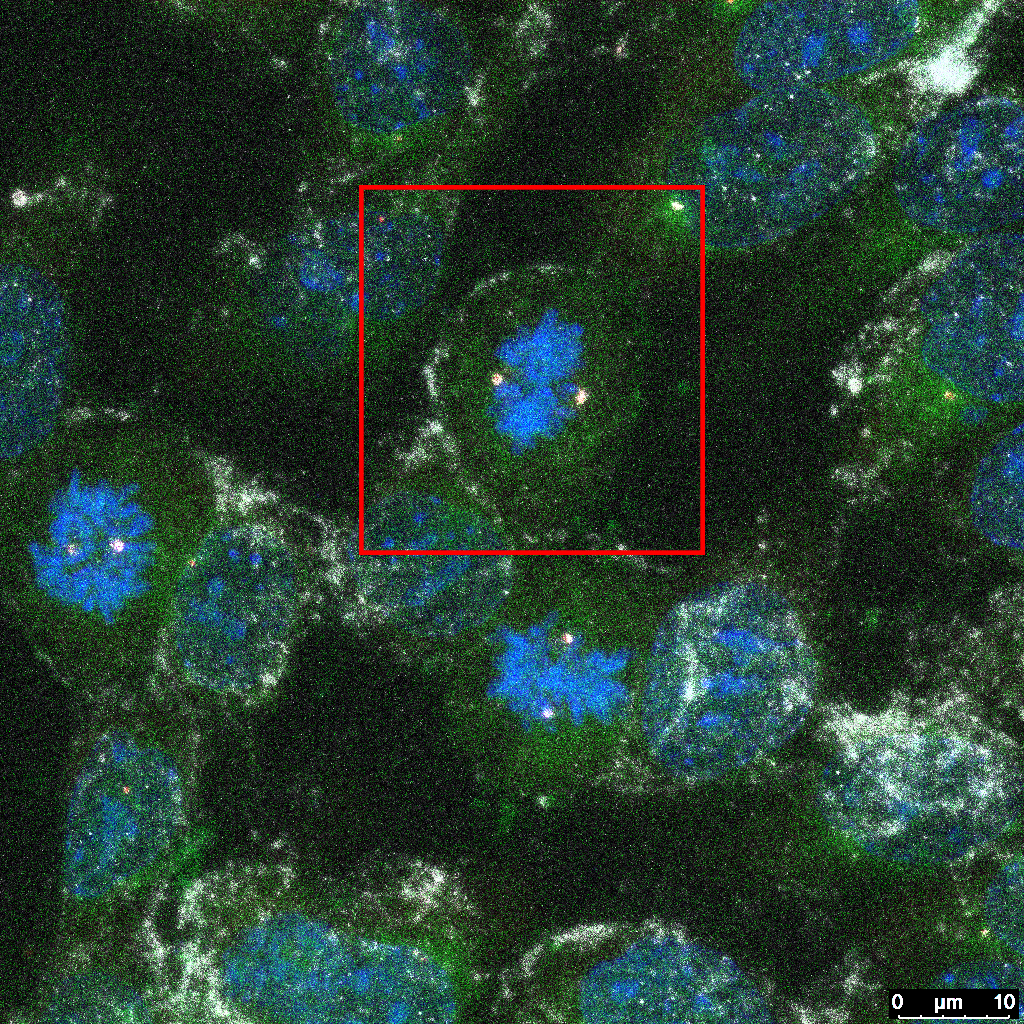

Supplement: Supplementary file 6 — Source data Fig. 4 [file 44319_2025_438_MOESM6_ESM.zip › SD figure 4/Fig. 4I/GFP.tif]

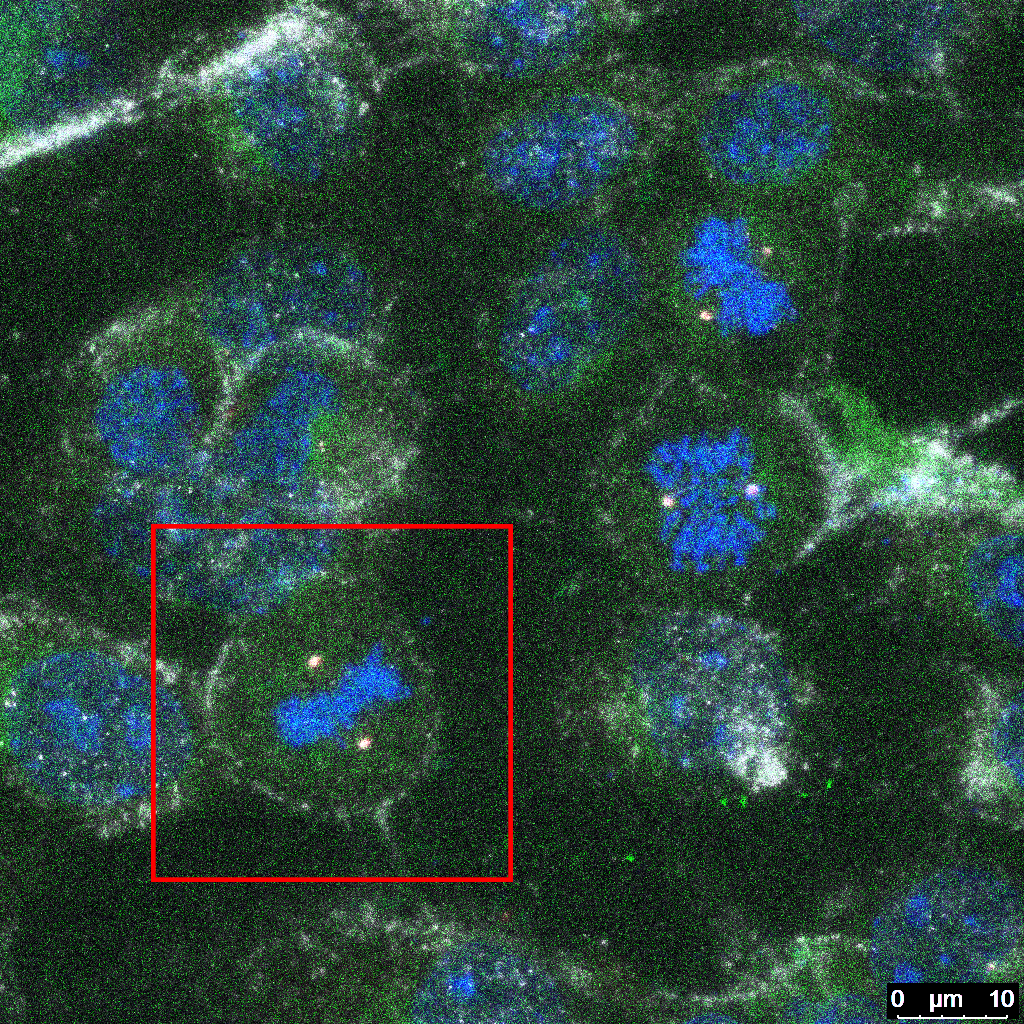

Supplement: Supplementary file 6 — Source data Fig. 4 [file 44319_2025_438_MOESM6_ESM.zip › SD figure 4/Fig. 4I/H215-610A.tif]

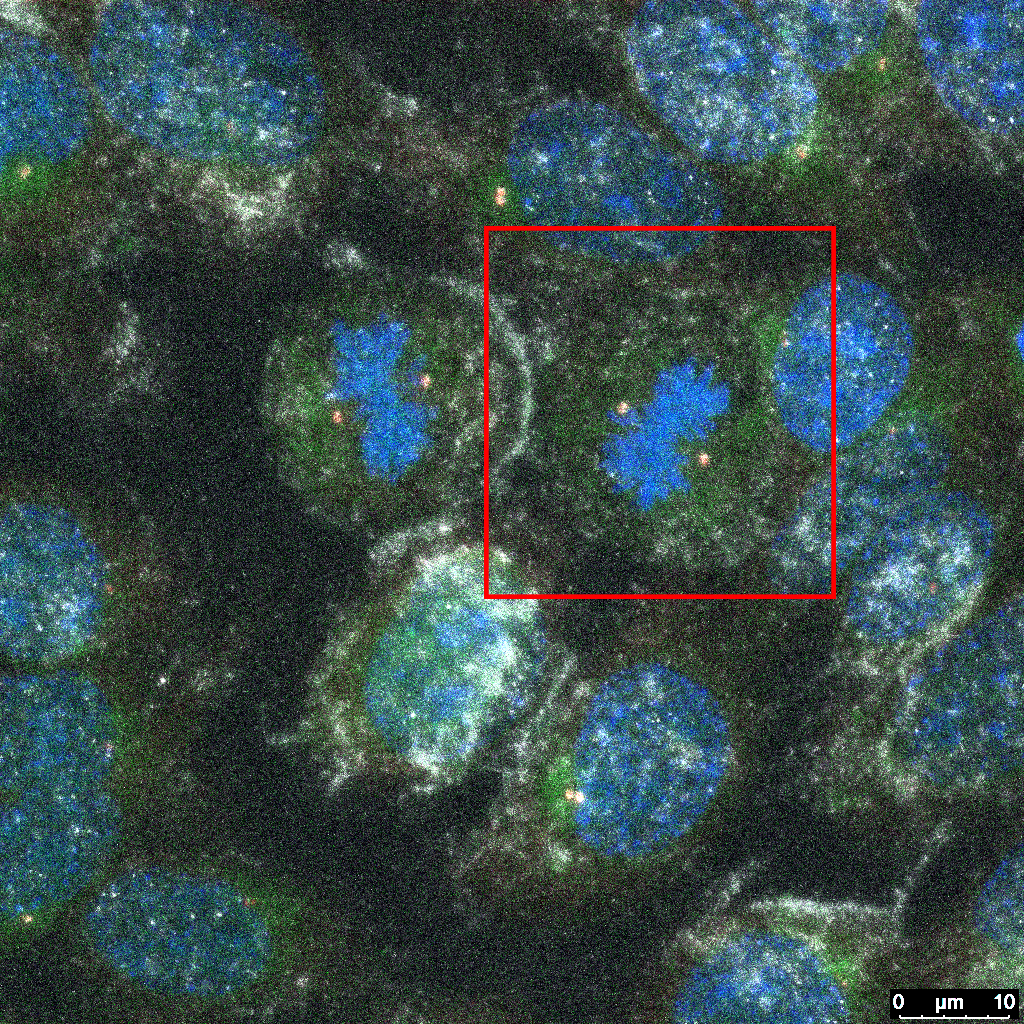

Supplement: Supplementary file 6 — Source data Fig. 4 [file 44319_2025_438_MOESM6_ESM.zip › SD figure 4/Fig. 4I/H215A.tif]

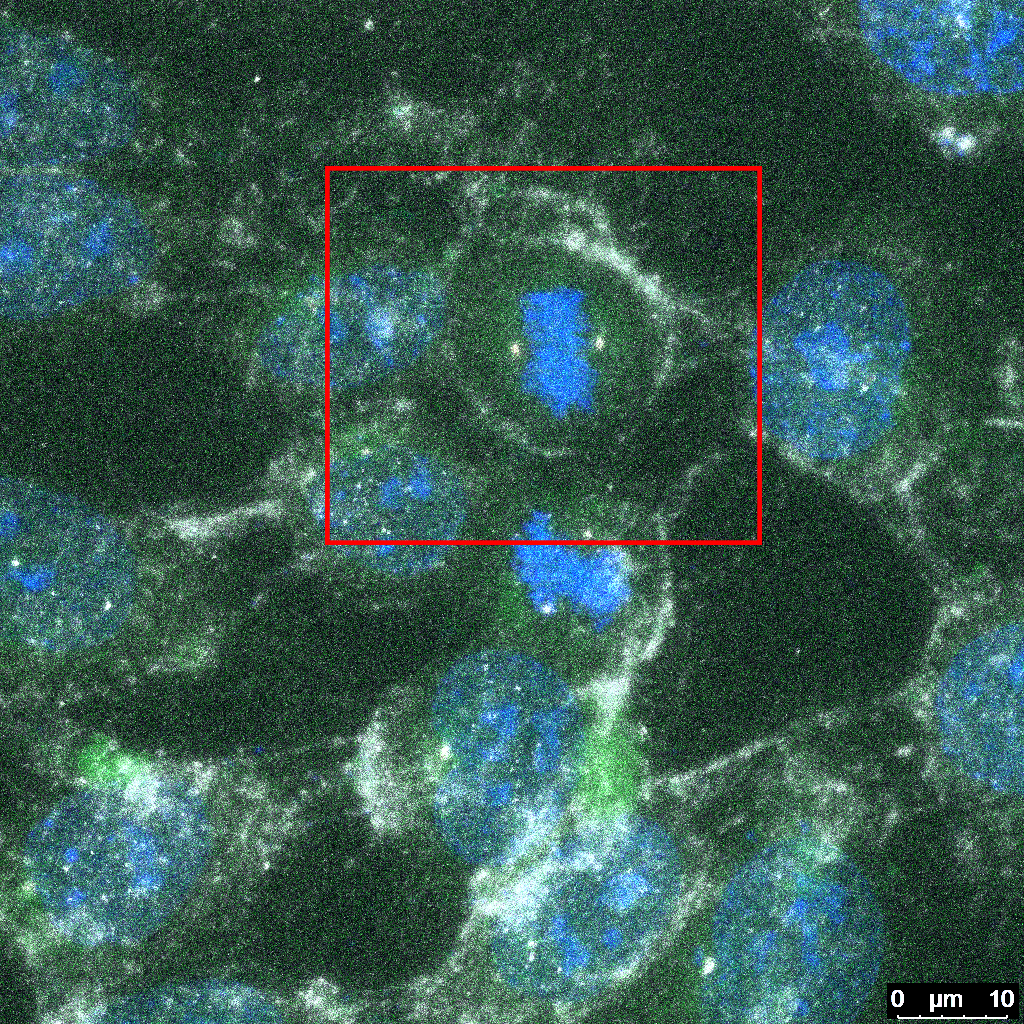

Supplement: Supplementary file 6 — Source data Fig. 4 [file 44319_2025_438_MOESM6_ESM.zip › SD figure 4/Fig. 4I/H610A.tif]

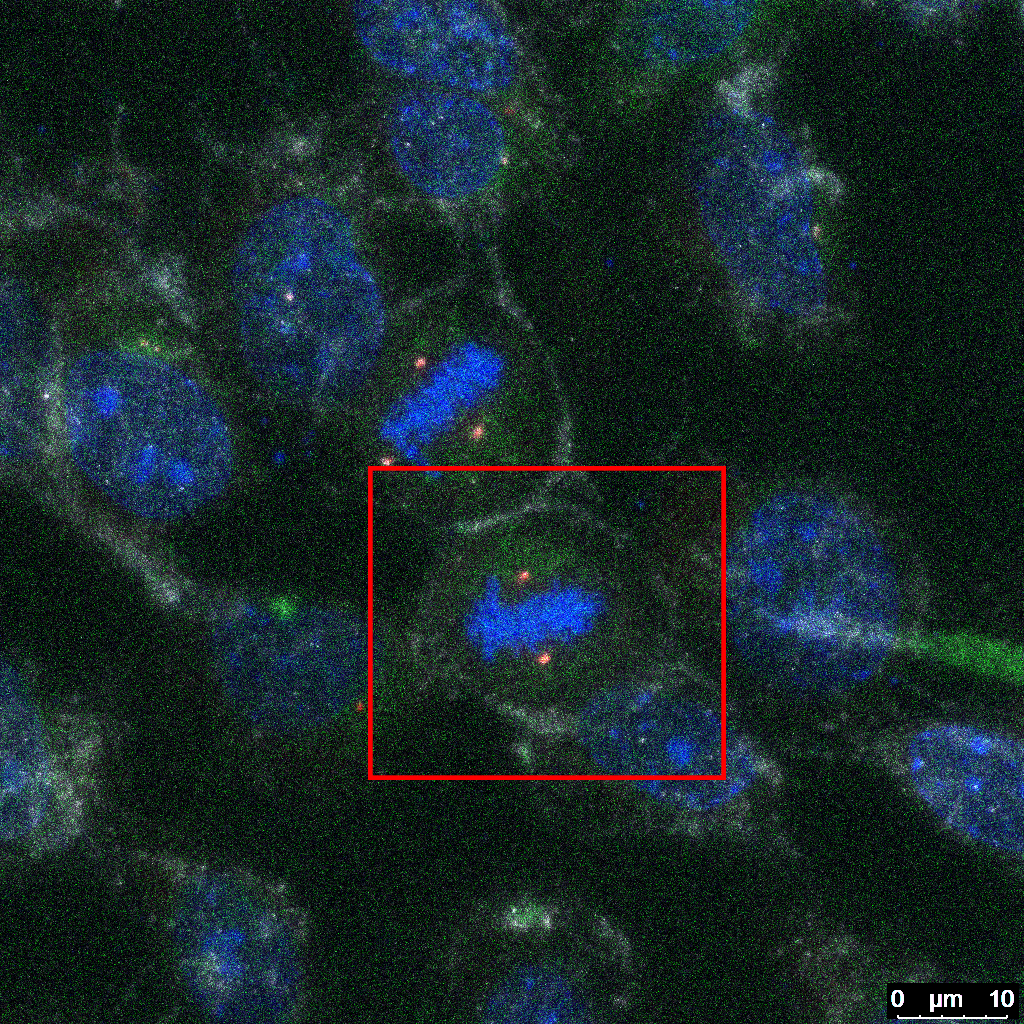

Supplement: Supplementary file 6 — Source data Fig. 4 [file 44319_2025_438_MOESM6_ESM.zip › SD figure 4/Fig. 4I/WT.tif]

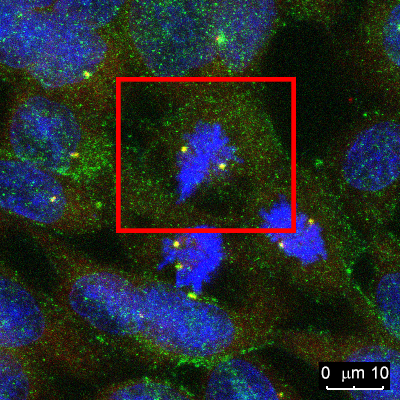

Supplement: Supplementary file 6 — Source data Fig. 4 [file 44319_2025_438_MOESM6_ESM.zip › SD figure 4/Fig. 4K/GFP-K98Q.tif]

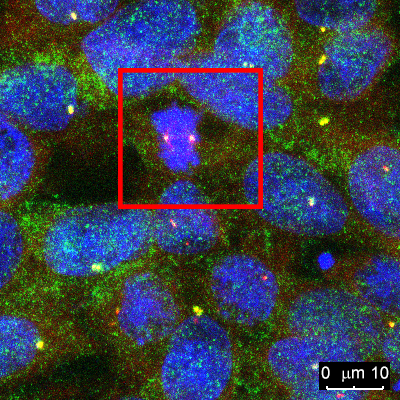

Supplement: Supplementary file 6 — Source data Fig. 4 [file 44319_2025_438_MOESM6_ESM.zip › SD figure 4/Fig. 4K/GFP-K98R.tif]

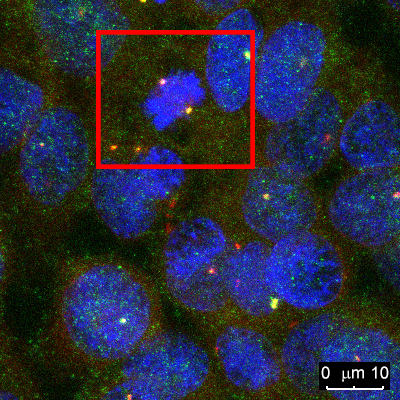

Supplement: Supplementary file 6 — Source data Fig. 4 [file 44319_2025_438_MOESM6_ESM.zip › SD figure 4/Fig. 4K/GFP-WT.tif]

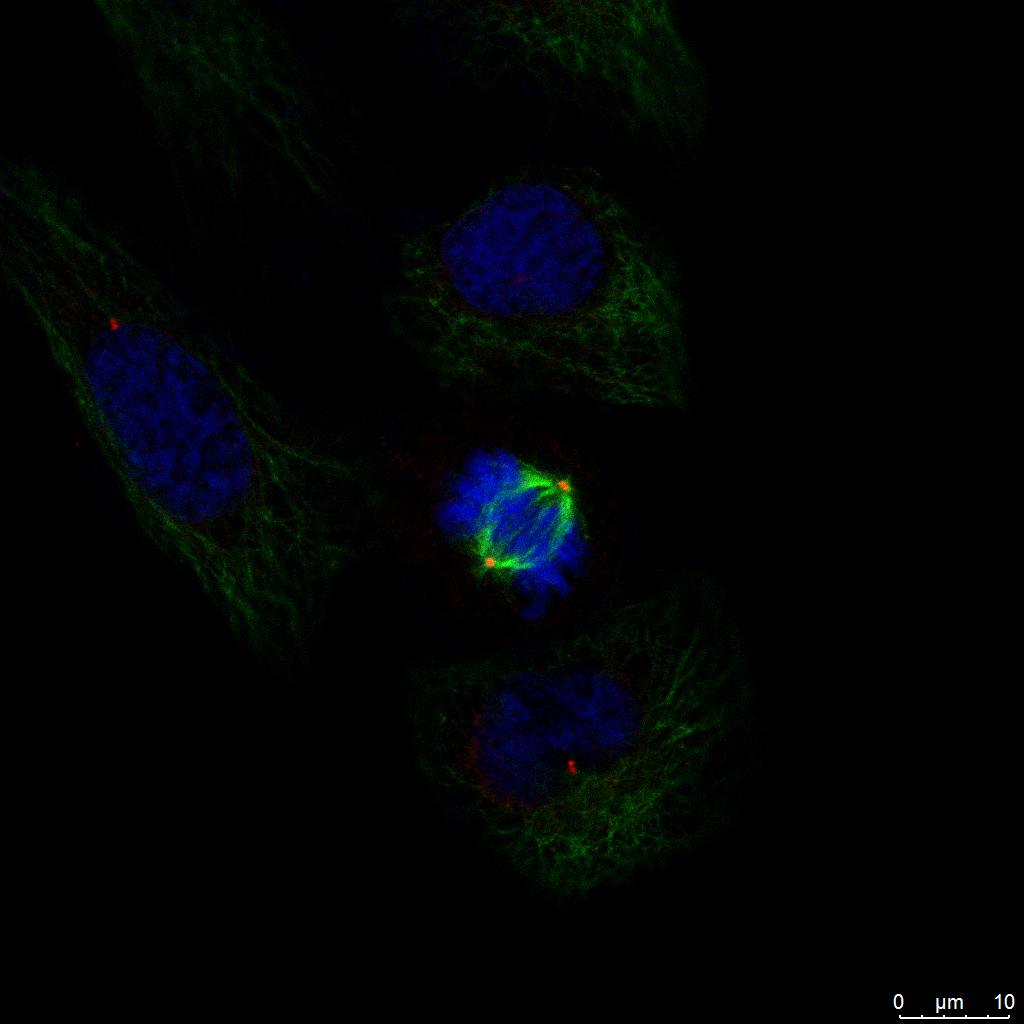

Supplement: Supplementary file 7 — Source data Fig. 5 [file 44319_2025_438_MOESM7_ESM.zip › SD figure 5/Fig. 5A/siControl-0.45.tif]

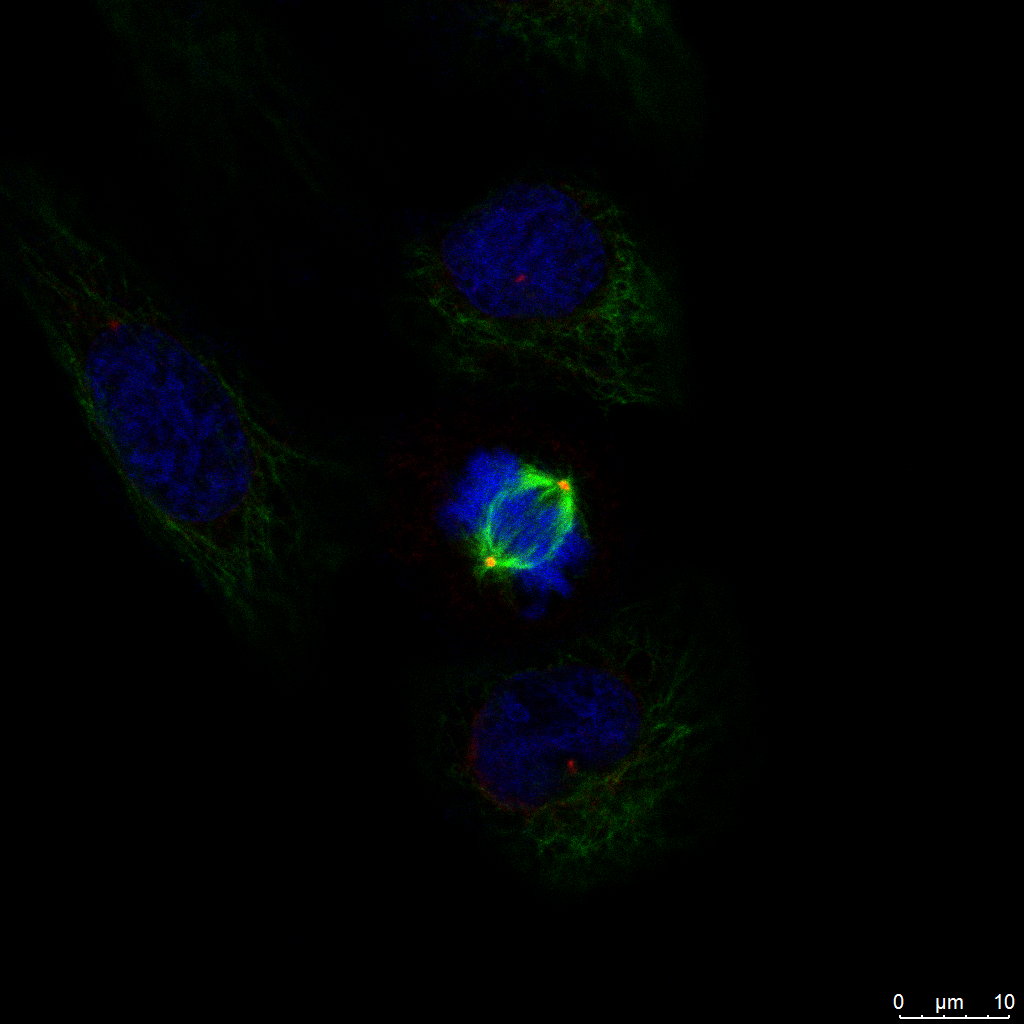

Supplement: Supplementary file 7 — Source data Fig. 5 [file 44319_2025_438_MOESM7_ESM.zip › SD figure 5/Fig. 5A/siControl-0.9.tif]

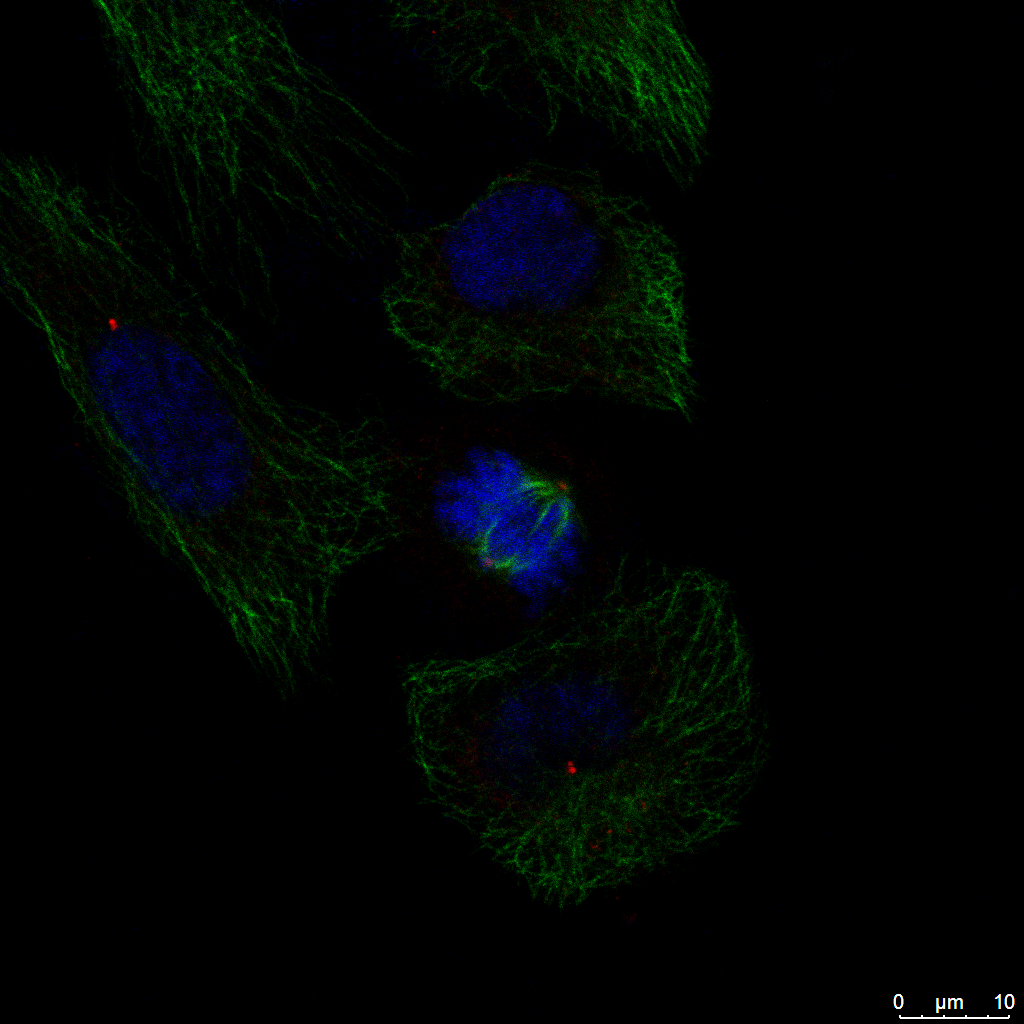

Supplement: Supplementary file 7 — Source data Fig. 5 [file 44319_2025_438_MOESM7_ESM.zip › SD figure 5/Fig. 5A/siControl-0.tif]

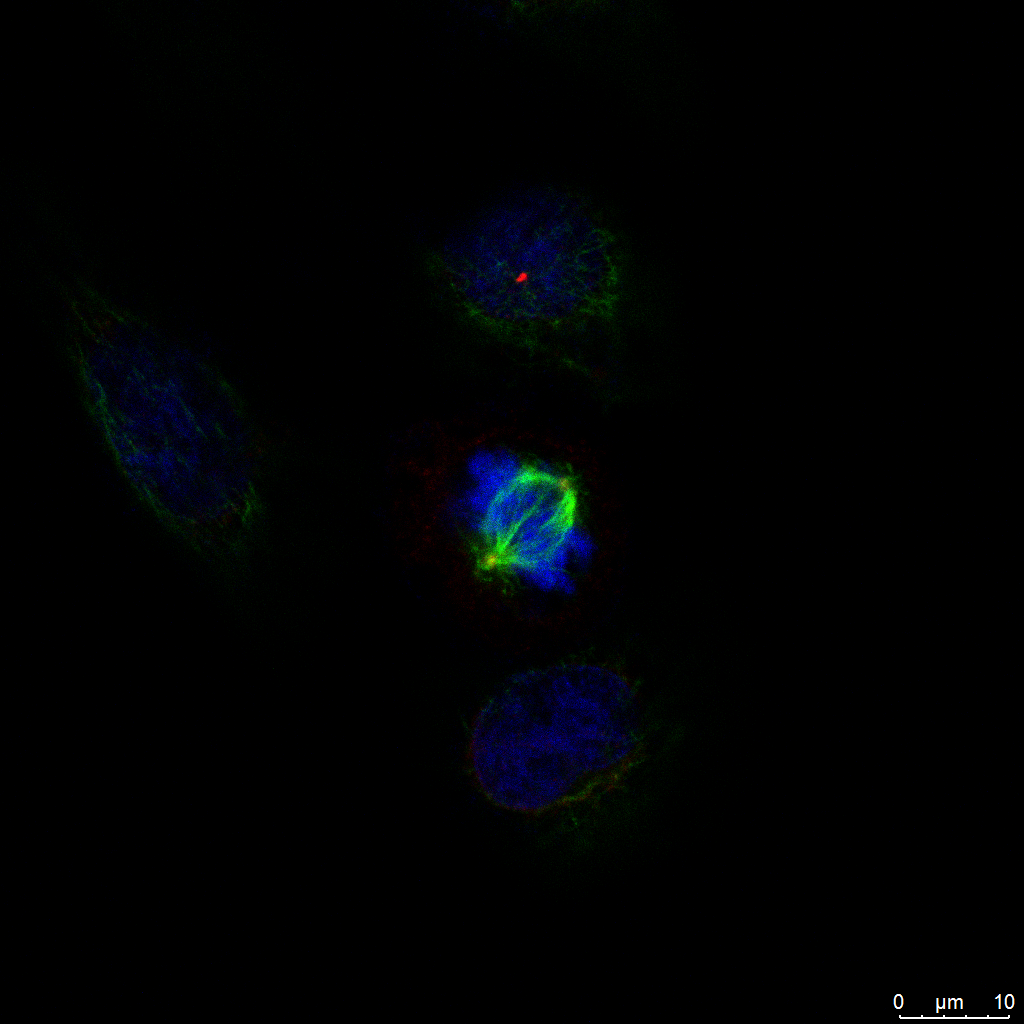

Supplement: Supplementary file 7 — Source data Fig. 5 [file 44319_2025_438_MOESM7_ESM.zip › SD figure 5/Fig. 5A/siControl-1.35.tif]

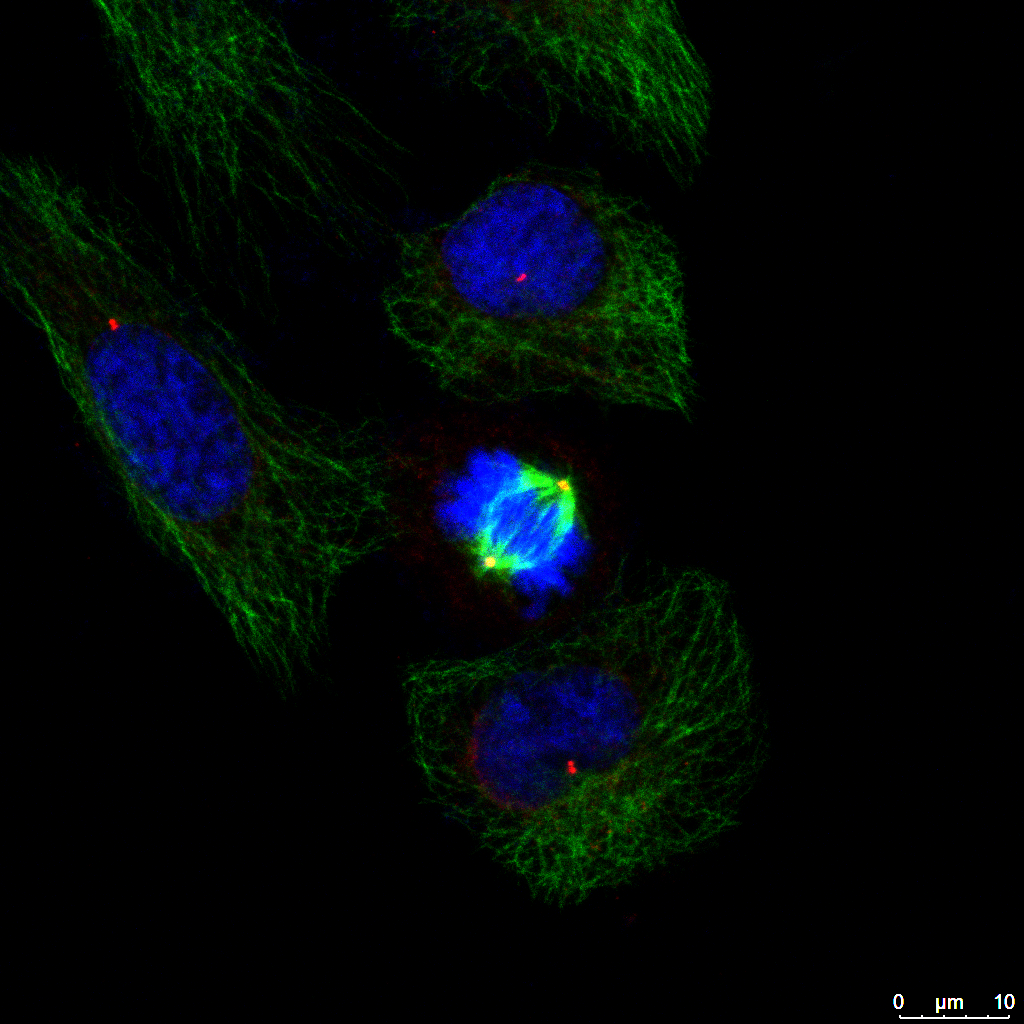

Supplement: Supplementary file 7 — Source data Fig. 5 [file 44319_2025_438_MOESM7_ESM.zip › SD figure 5/Fig. 5A/siControl-3D.tif]

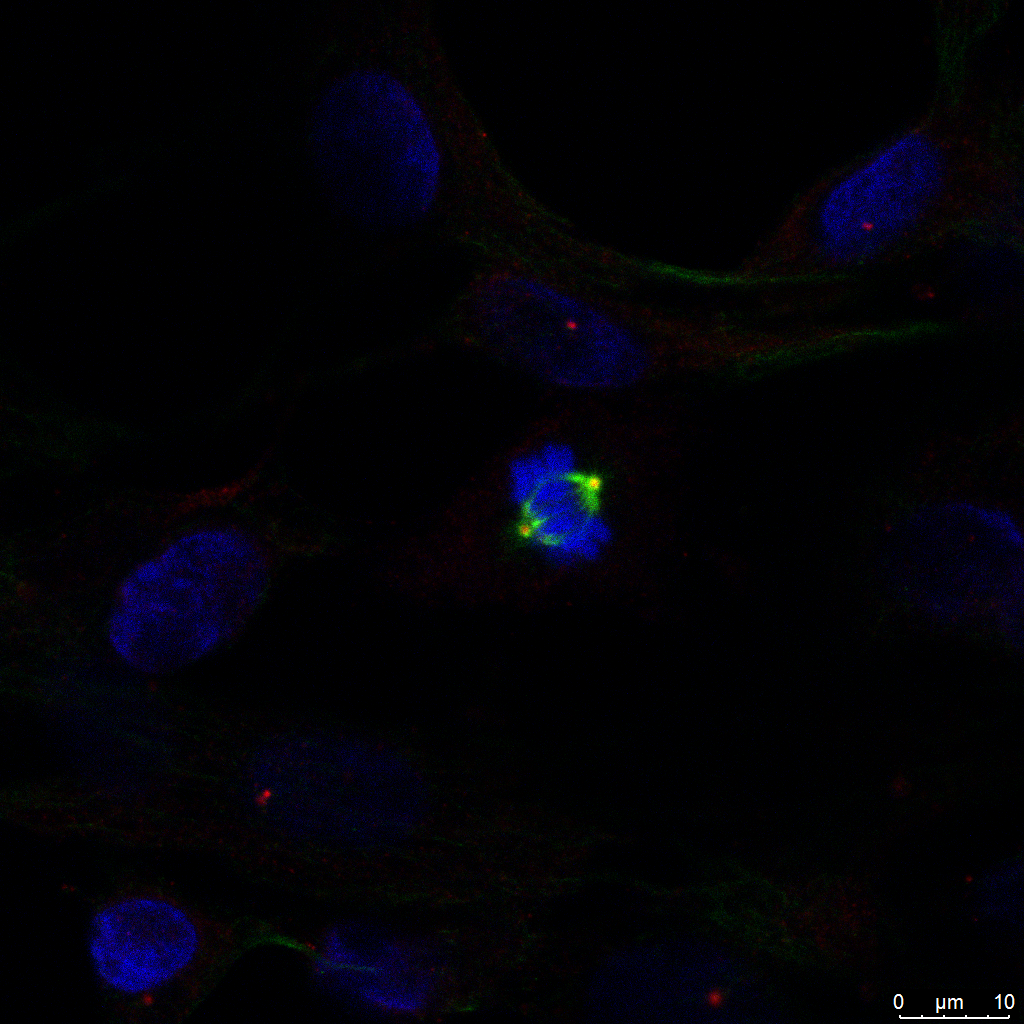

Supplement: Supplementary file 7 — Source data Fig. 5 [file 44319_2025_438_MOESM7_ESM.zip › SD figure 5/Fig. 5A/siENKD1#1-0.45.tif]

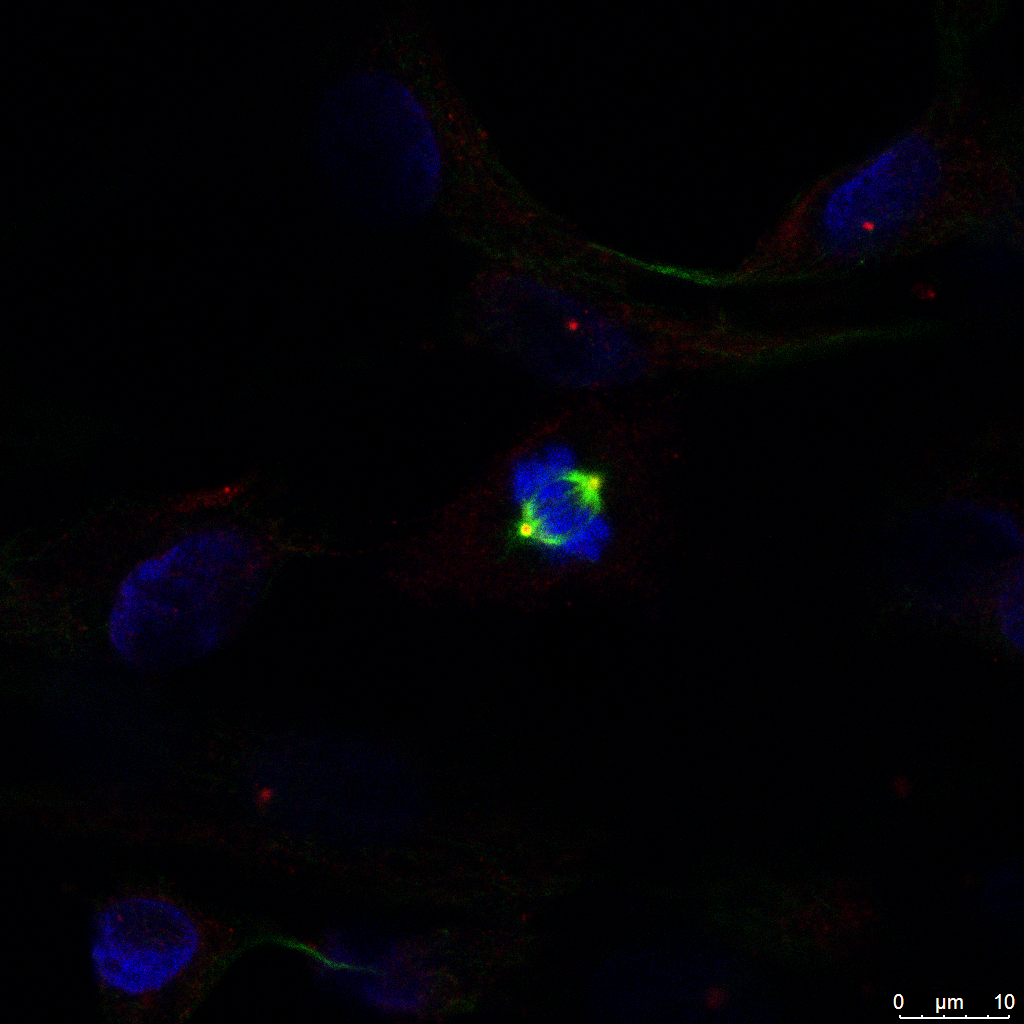

Supplement: Supplementary file 7 — Source data Fig. 5 [file 44319_2025_438_MOESM7_ESM.zip › SD figure 5/Fig. 5A/siENKD1#1-0.9.tif]

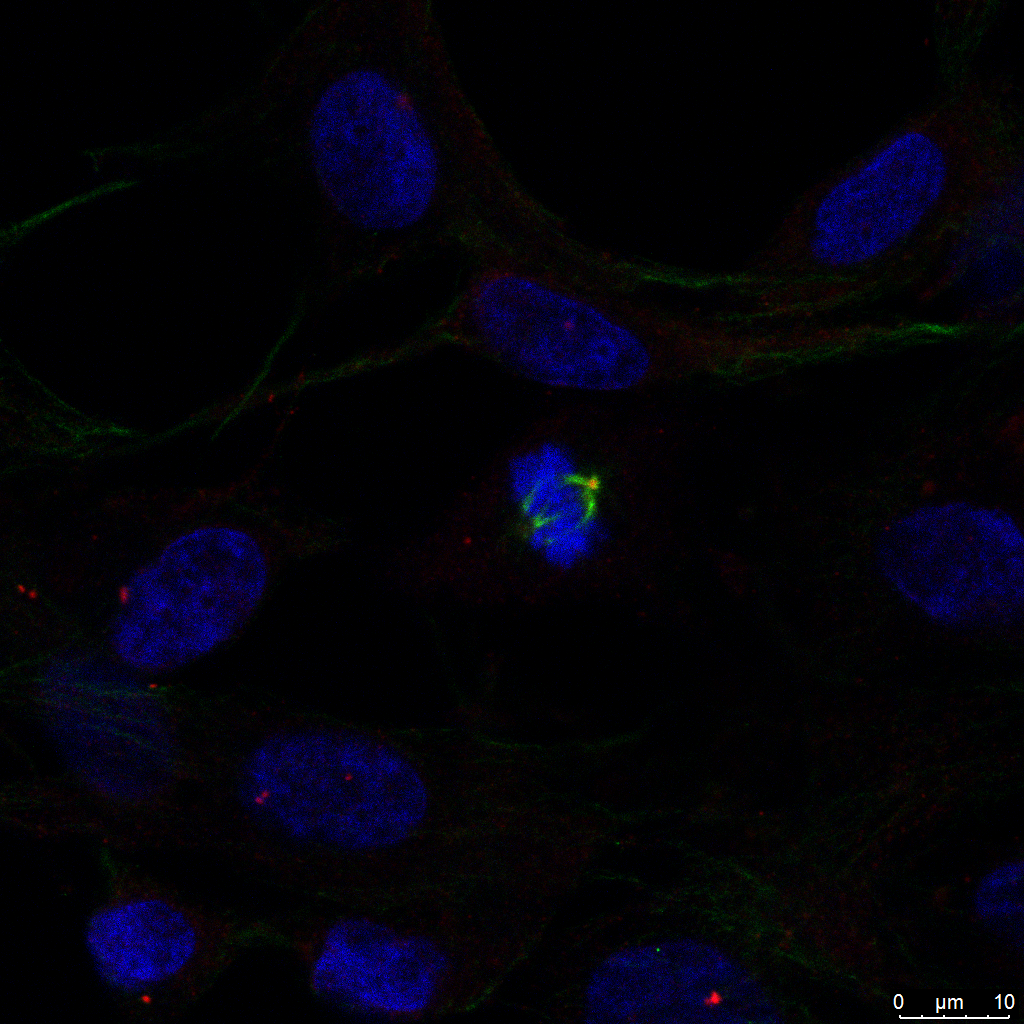

Supplement: Supplementary file 7 — Source data Fig. 5 [file 44319_2025_438_MOESM7_ESM.zip › SD figure 5/Fig. 5A/siENKD1#1-0.tif]

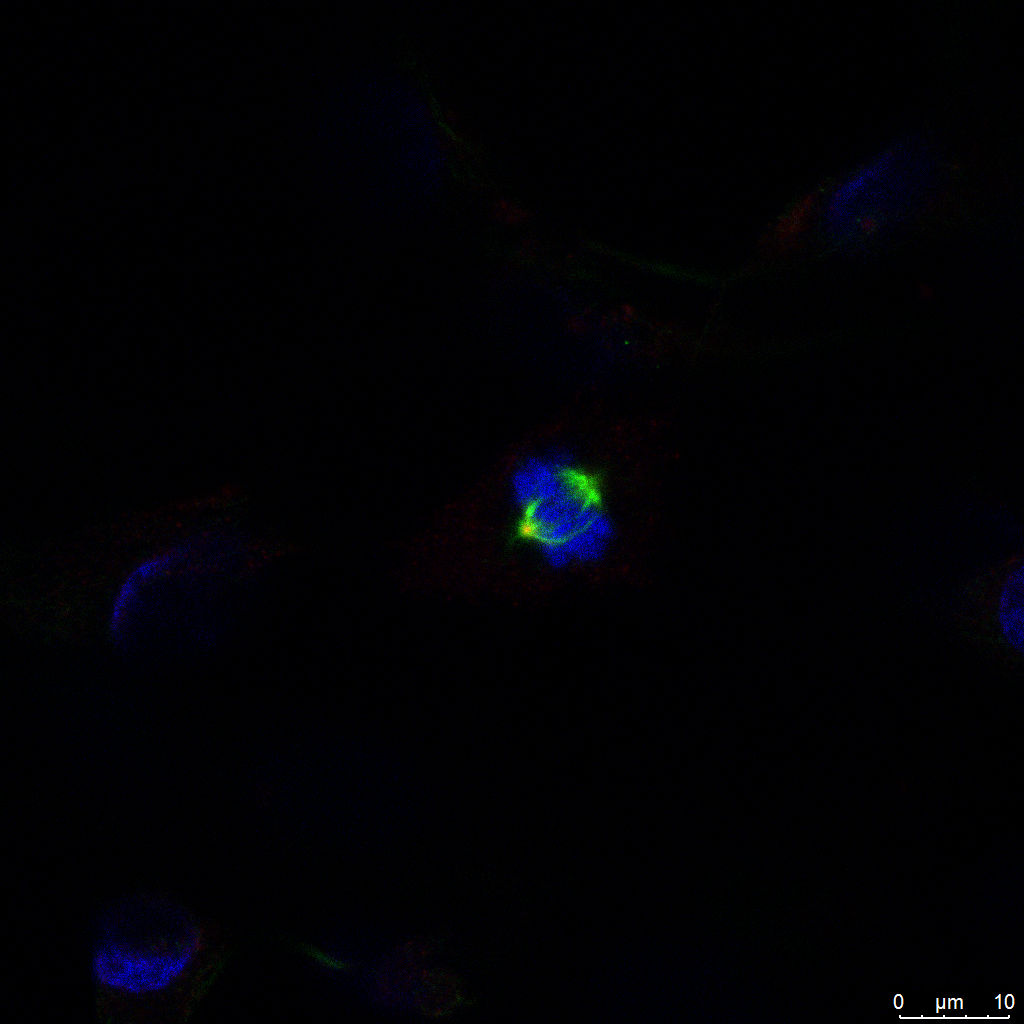

Supplement: Supplementary file 7 — Source data Fig. 5 [file 44319_2025_438_MOESM7_ESM.zip › SD figure 5/Fig. 5A/siENKD1#1-1.35.tif]

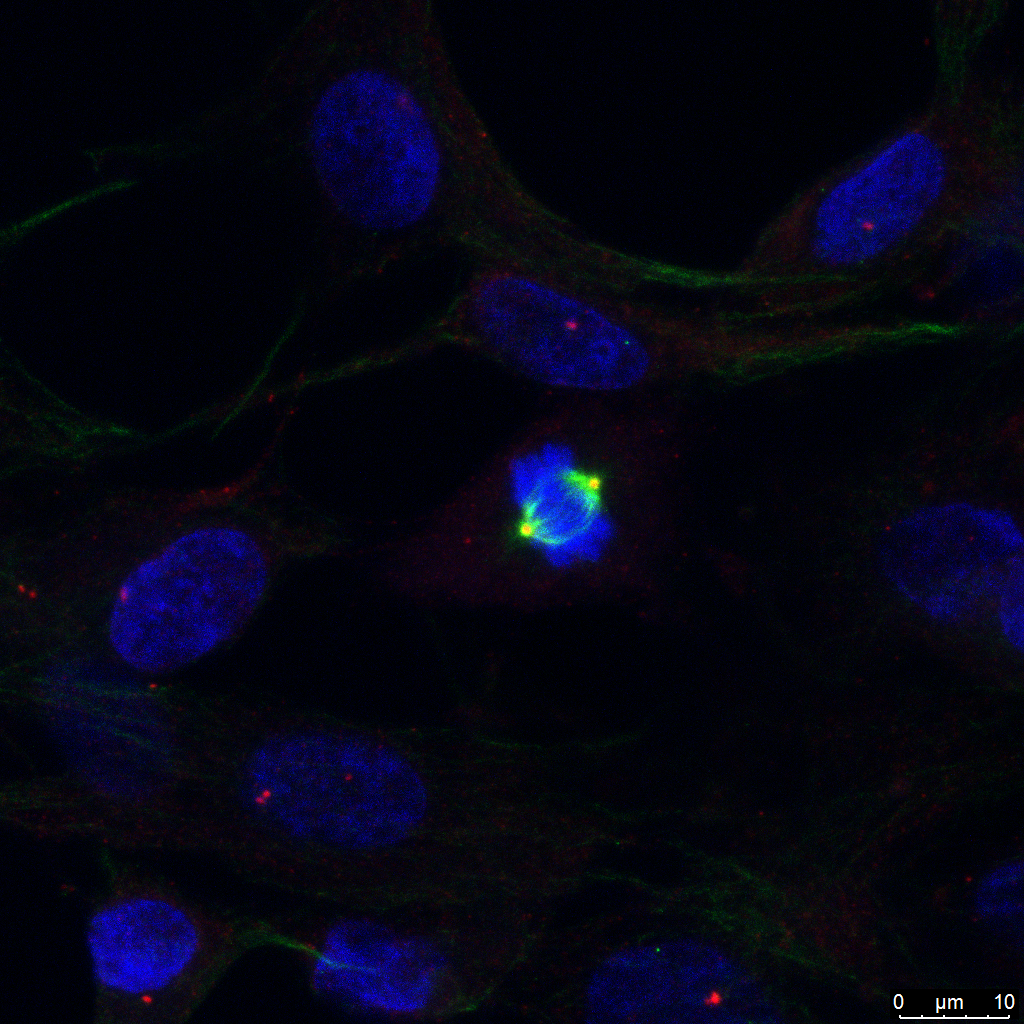

Supplement: Supplementary file 7 — Source data Fig. 5 [file 44319_2025_438_MOESM7_ESM.zip › SD figure 5/Fig. 5A/siENKD1#1-3D.tif]
