# Supplementary figures and images for: HDAC6 deacetylates ENKD1 to regulate mitotic spindle behavior and corneal epithelial homeostasis (part 2 of 2)
Source: EMBO Rep. 2025 Mar 28;26(10):2597–621. doi: 10.1038/s44319-025-00438-0 (PMC12116779; doi:10.1038/s44319-025-00438-0)

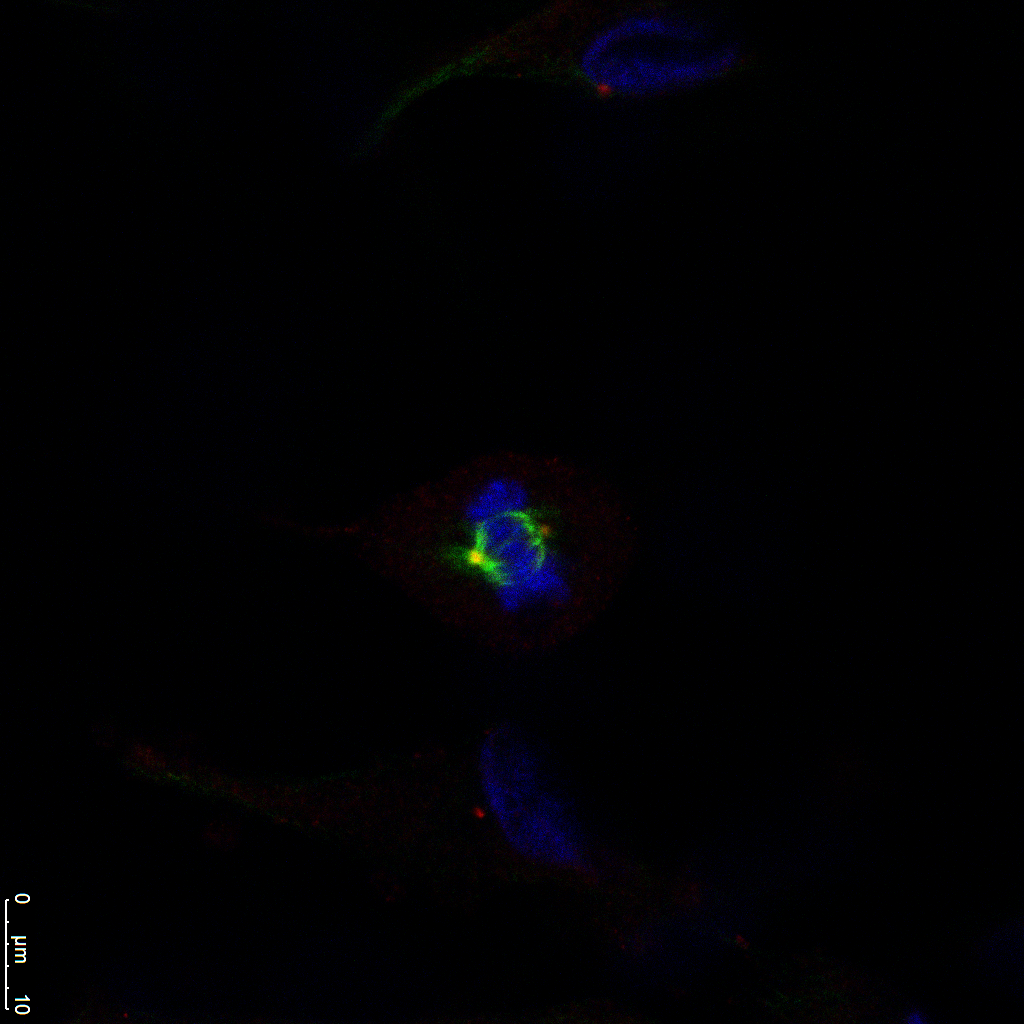

Supplement: Supplementary file 7 — Source data Fig. 5 [file 44319_2025_438_MOESM7_ESM.zip › SD figure 5/Fig. 5A/siENKD1#2-0.45.tif]

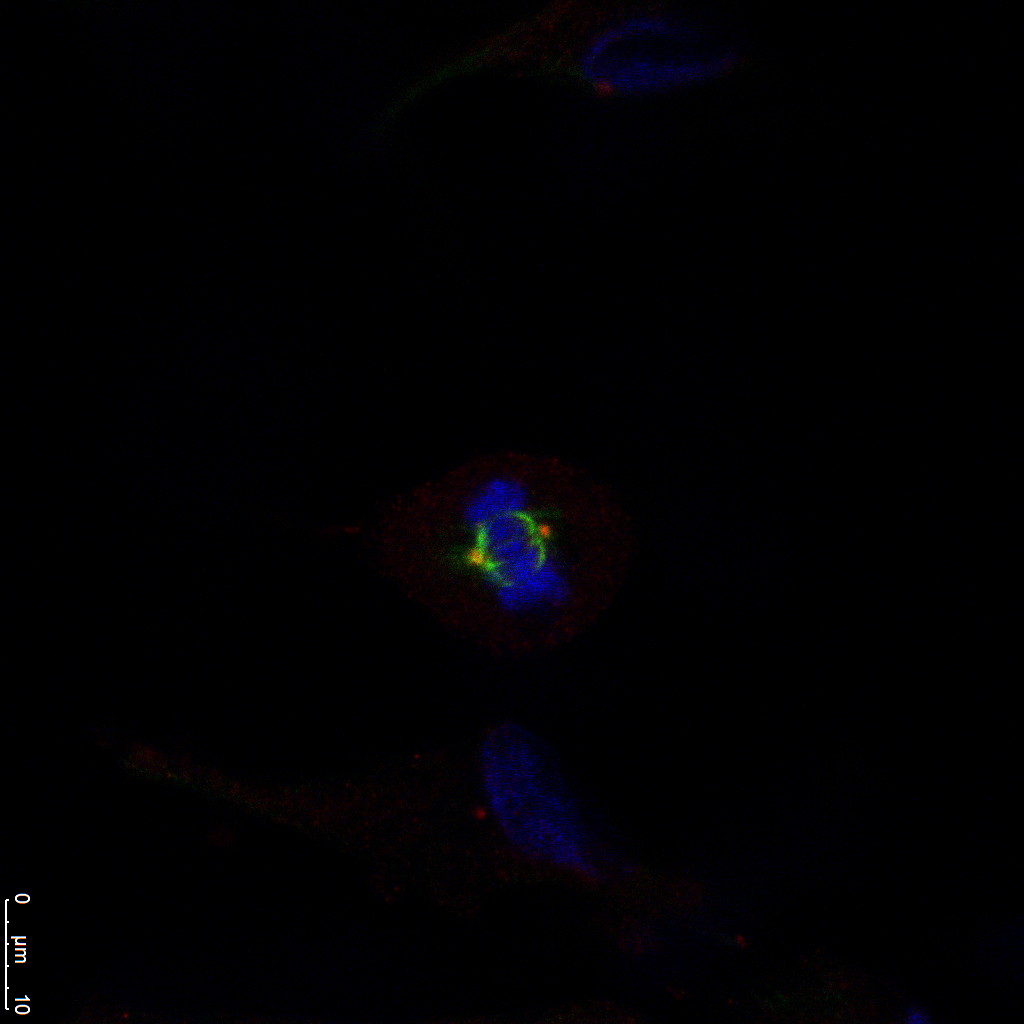

Supplement: Supplementary file 7 — Source data Fig. 5 [file 44319_2025_438_MOESM7_ESM.zip › SD figure 5/Fig. 5A/siENKD1#2-0.9.tif]

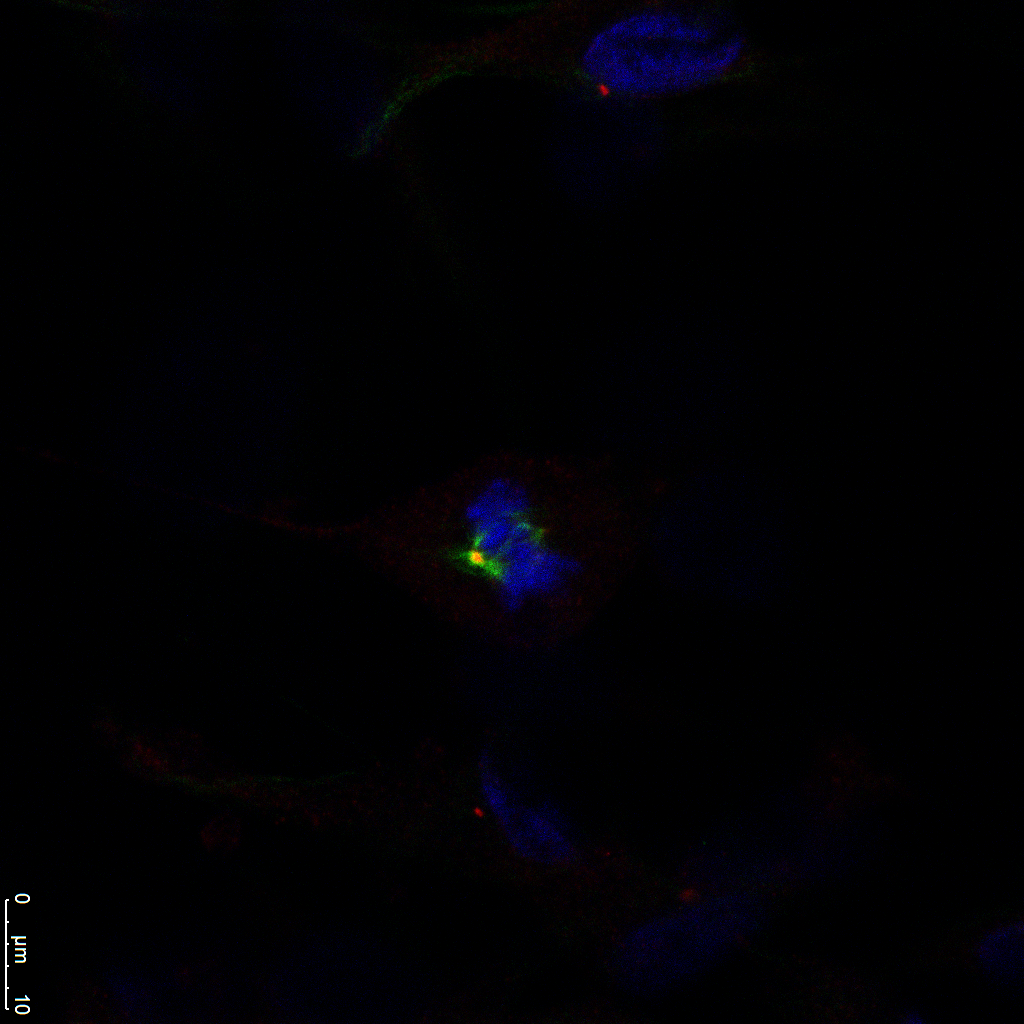

Supplement: Supplementary file 7 — Source data Fig. 5 [file 44319_2025_438_MOESM7_ESM.zip › SD figure 5/Fig. 5A/siENKD1#2-0.tif]

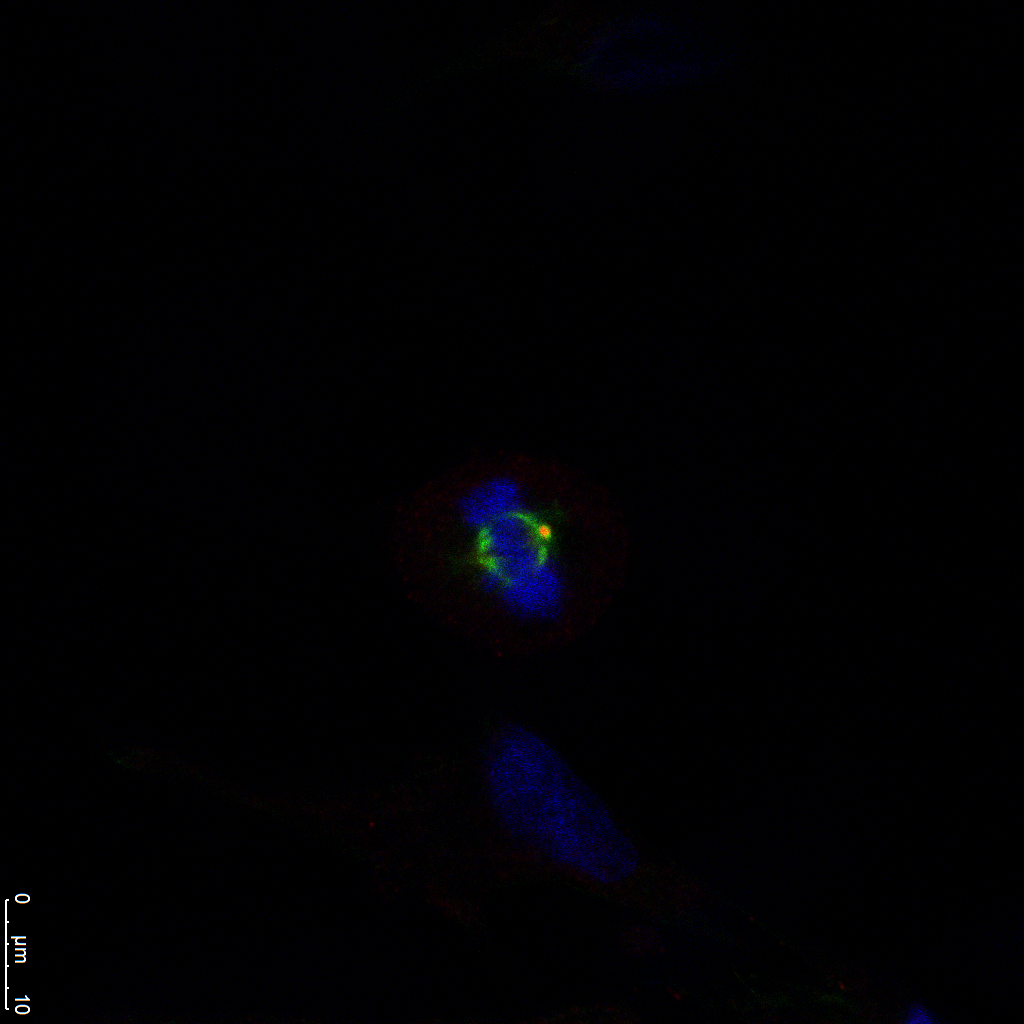

Supplement: Supplementary file 7 — Source data Fig. 5 [file 44319_2025_438_MOESM7_ESM.zip › SD figure 5/Fig. 5A/siENKD1#2-1.35.tif]

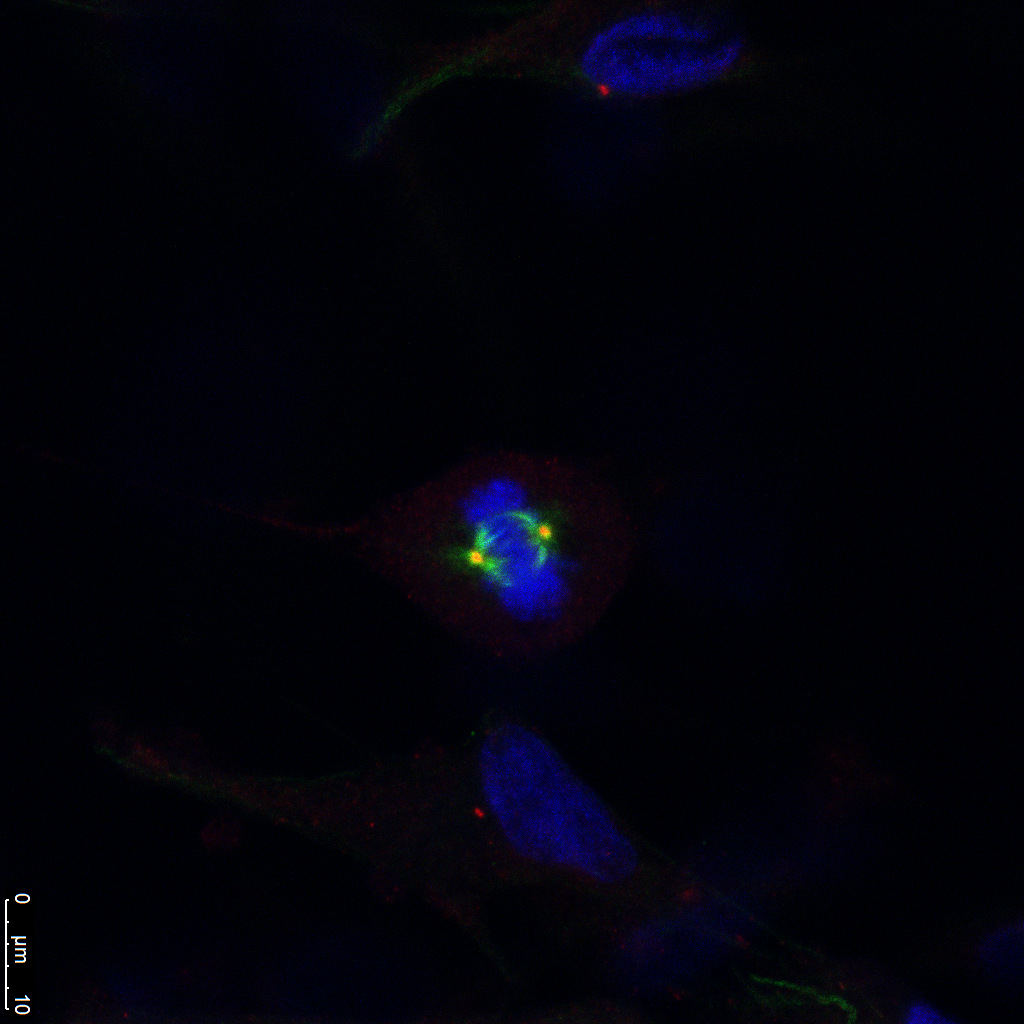

Supplement: Supplementary file 7 — Source data Fig. 5 [file 44319_2025_438_MOESM7_ESM.zip › SD figure 5/Fig. 5A/siENKD1#2-3D.tif]

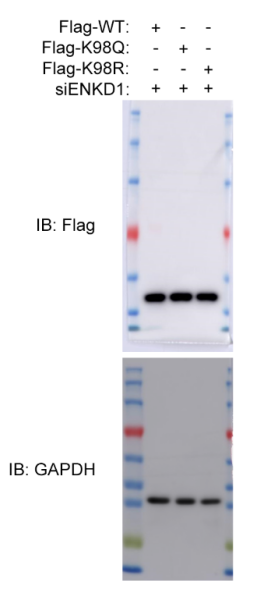

Supplement: Supplementary file 7 — Source data Fig. 5 [file 44319_2025_438_MOESM7_ESM.zip › SD figure 5/Fig. 5C/Fig. 5C.png]

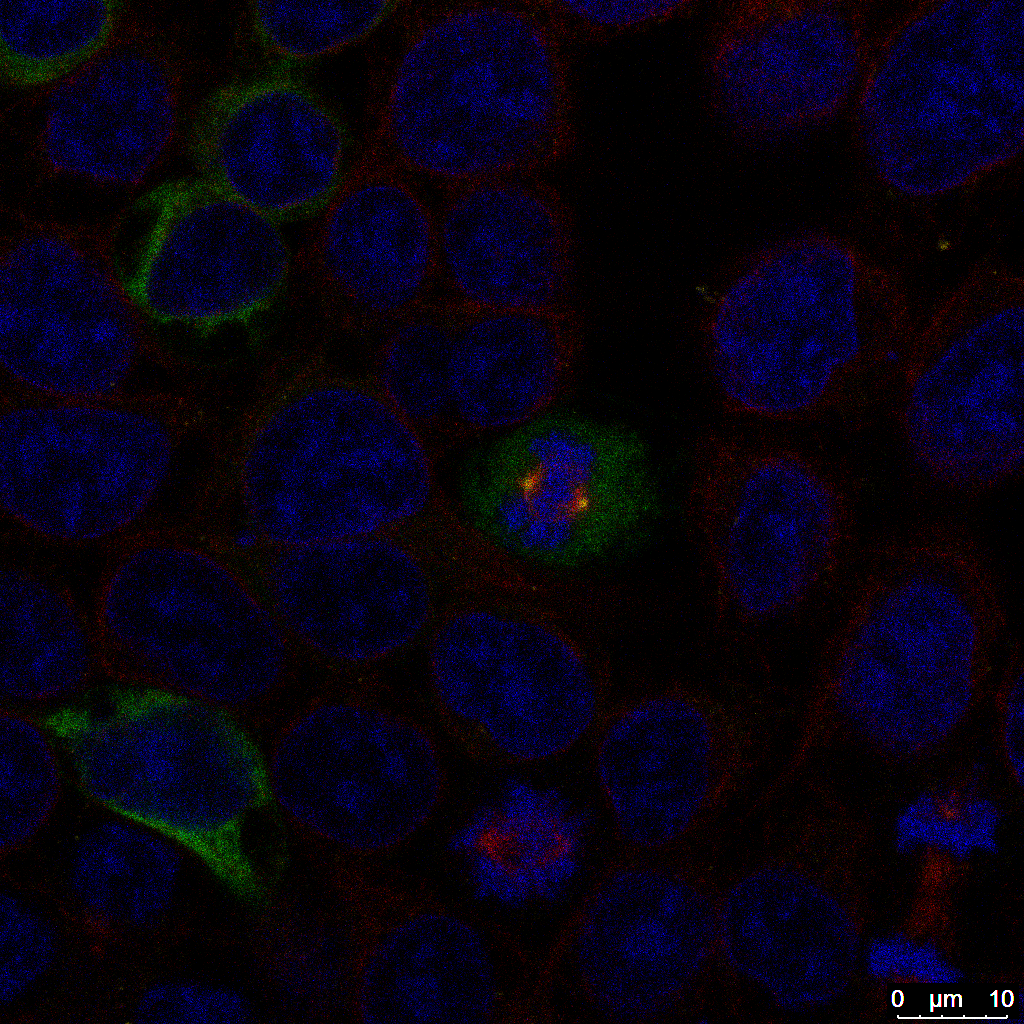

Supplement: Supplementary file 7 — Source data Fig. 5 [file 44319_2025_438_MOESM7_ESM.zip › SD figure 5/Fig. 5E/GFP-K98Q-0.45.tif]

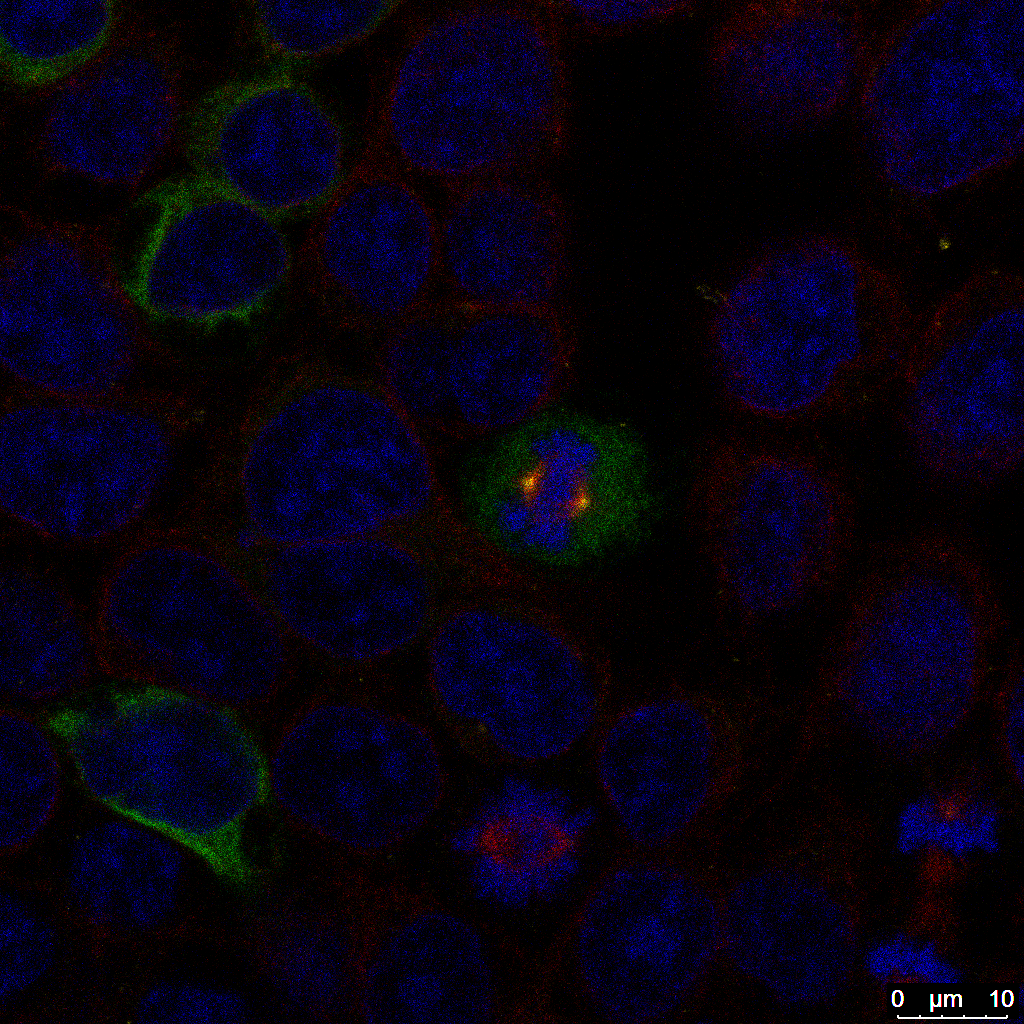

Supplement: Supplementary file 7 — Source data Fig. 5 [file 44319_2025_438_MOESM7_ESM.zip › SD figure 5/Fig. 5E/GFP-K98Q-0.9.tif]

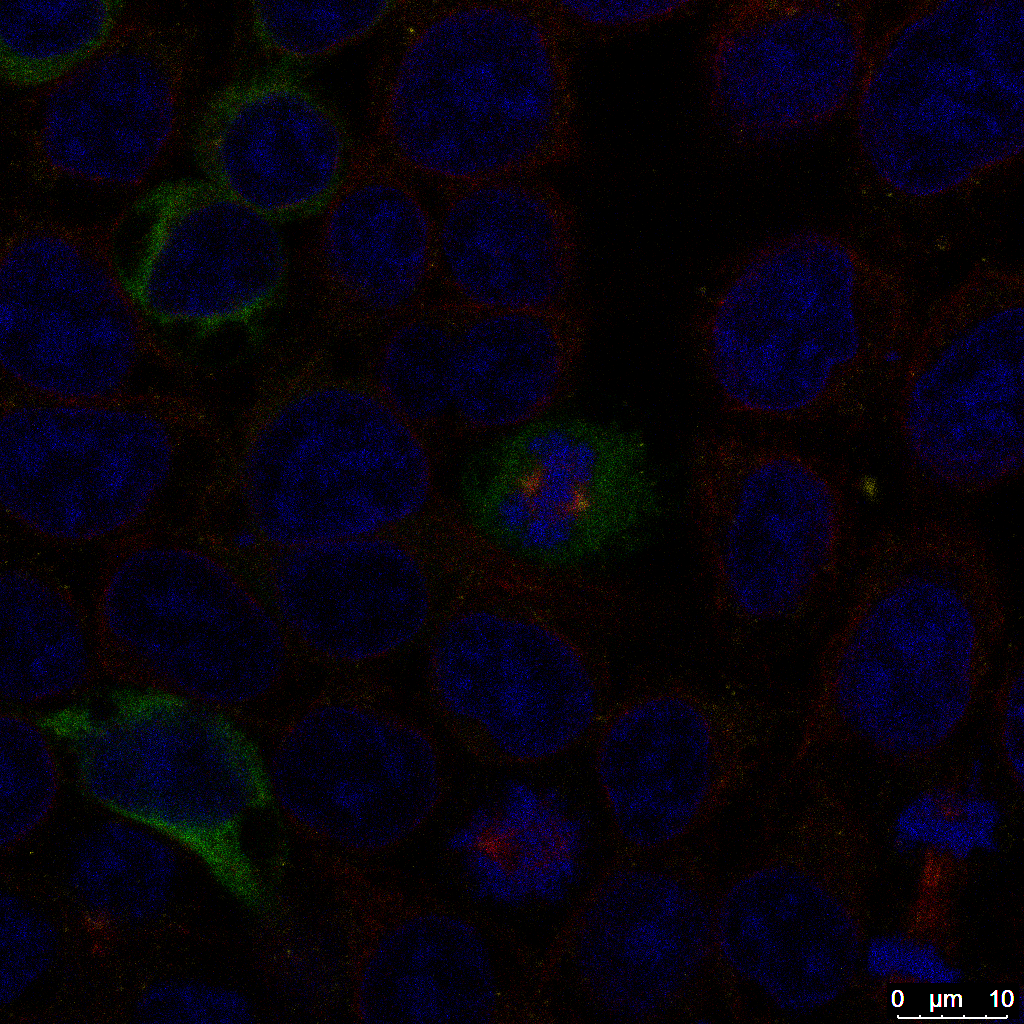

Supplement: Supplementary file 7 — Source data Fig. 5 [file 44319_2025_438_MOESM7_ESM.zip › SD figure 5/Fig. 5E/GFP-K98Q-0.tif]

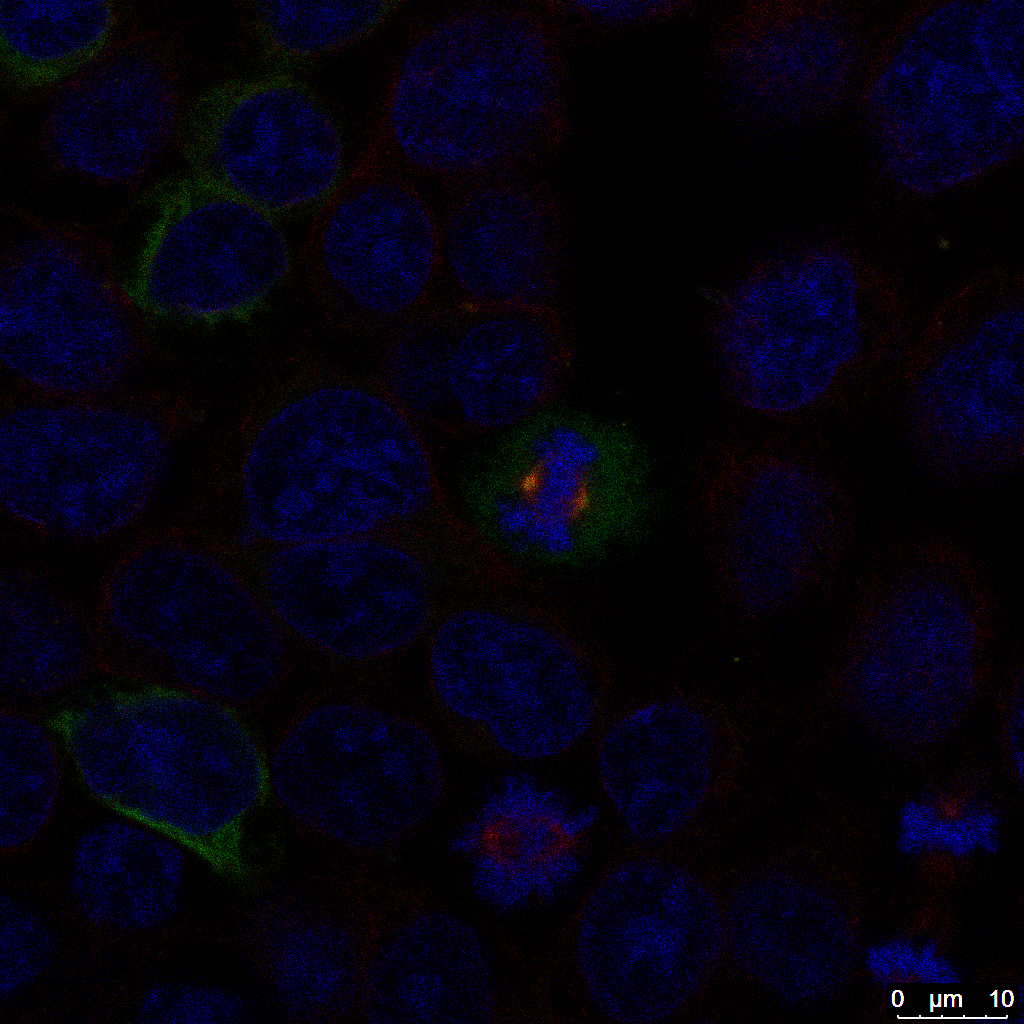

Supplement: Supplementary file 7 — Source data Fig. 5 [file 44319_2025_438_MOESM7_ESM.zip › SD figure 5/Fig. 5E/GFP-K98Q-1.35.tif]

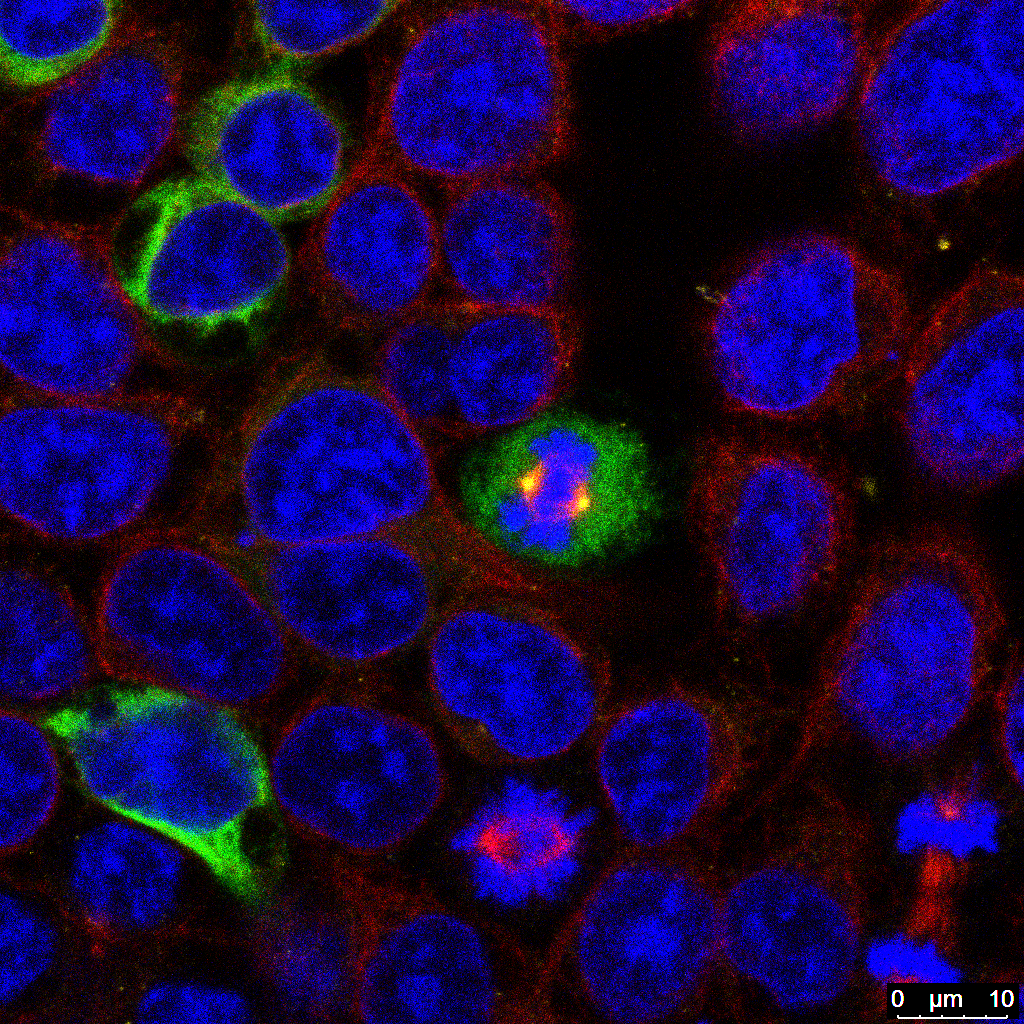

Supplement: Supplementary file 7 — Source data Fig. 5 [file 44319_2025_438_MOESM7_ESM.zip › SD figure 5/Fig. 5E/GFP-K98Q-3D.tif]

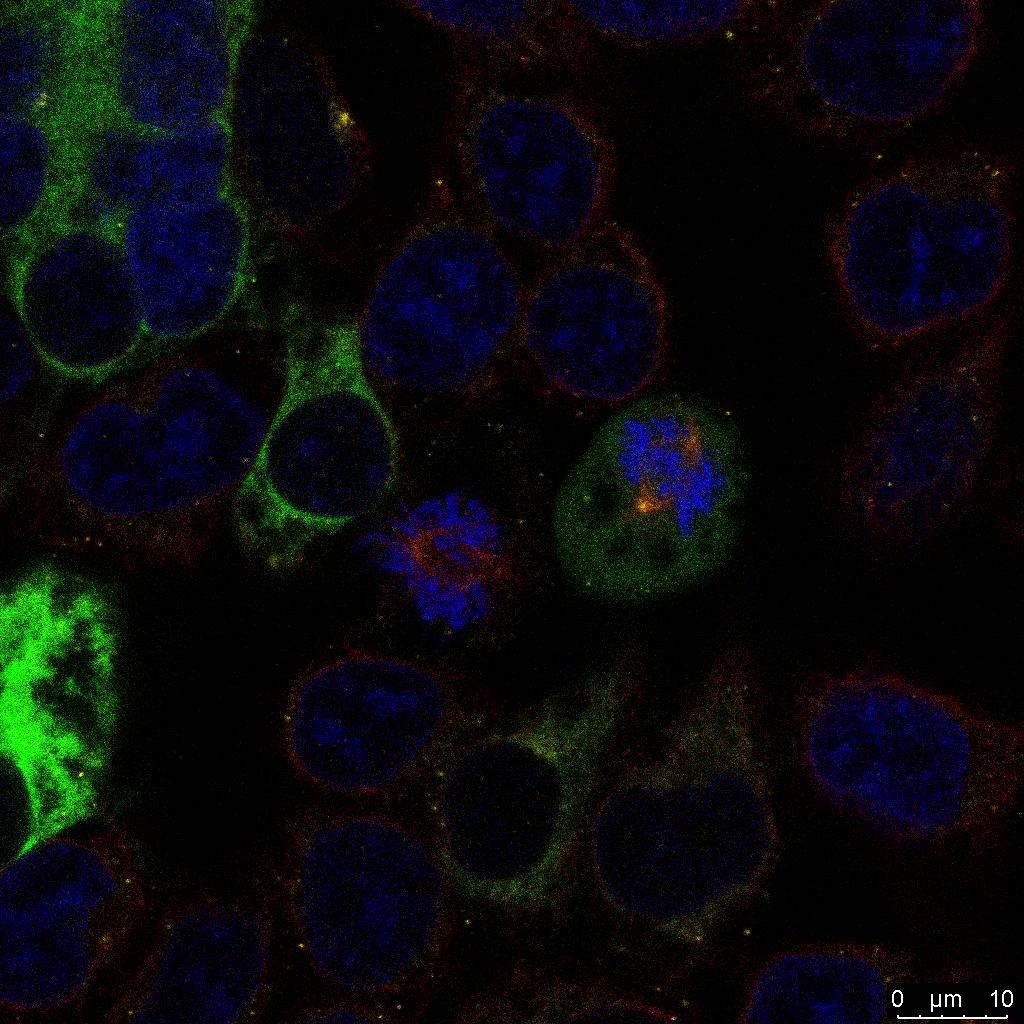

Supplement: Supplementary file 7 — Source data Fig. 5 [file 44319_2025_438_MOESM7_ESM.zip › SD figure 5/Fig. 5E/GFP-K98R-0.45.tif]

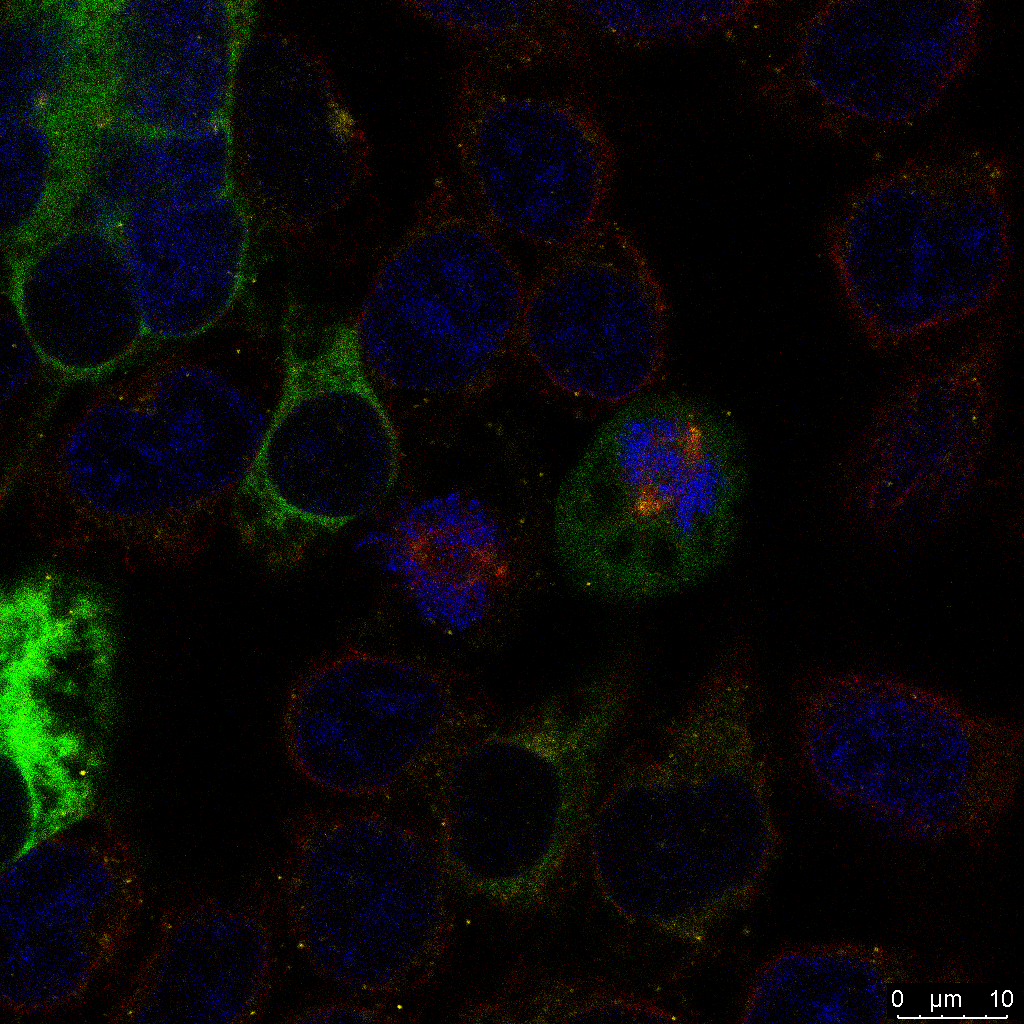

Supplement: Supplementary file 7 — Source data Fig. 5 [file 44319_2025_438_MOESM7_ESM.zip › SD figure 5/Fig. 5E/GFP-K98R-0.9.tif]

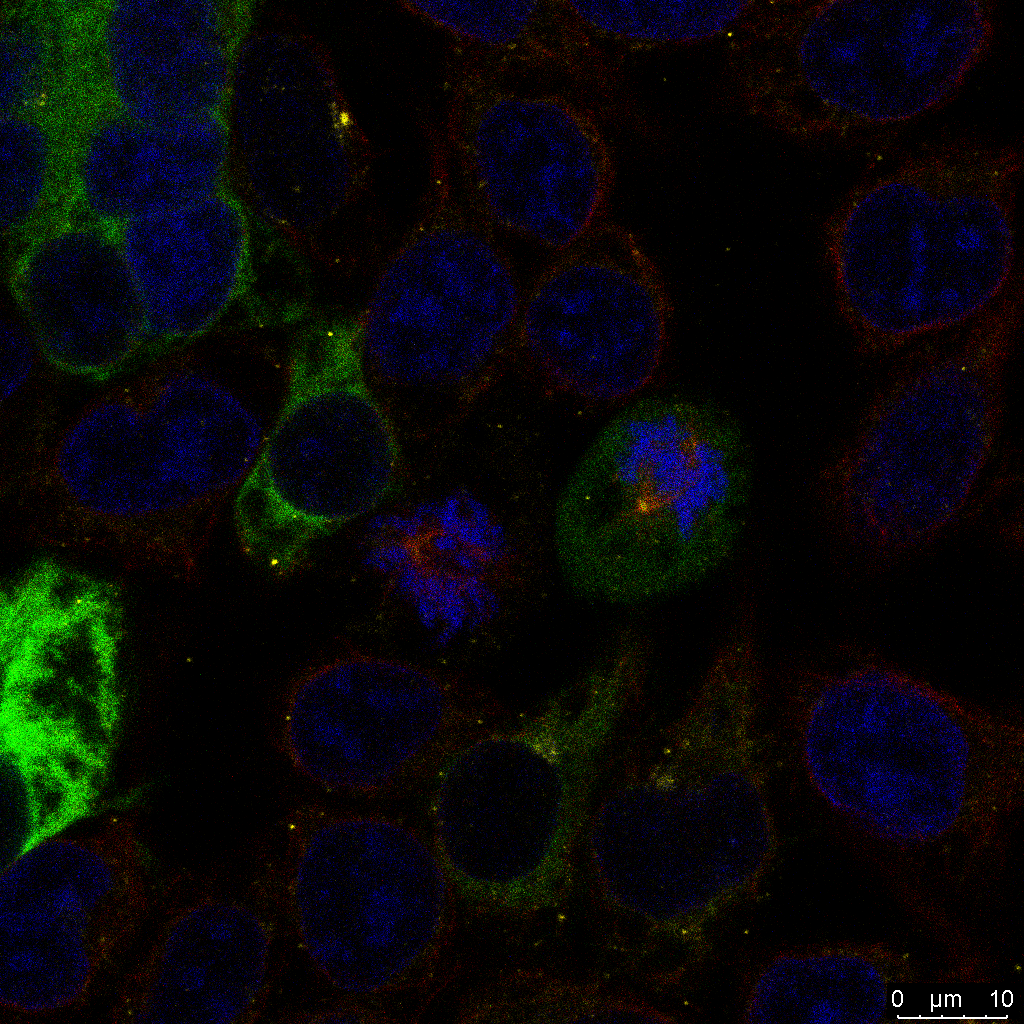

Supplement: Supplementary file 7 — Source data Fig. 5 [file 44319_2025_438_MOESM7_ESM.zip › SD figure 5/Fig. 5E/GFP-K98R-0.tif]

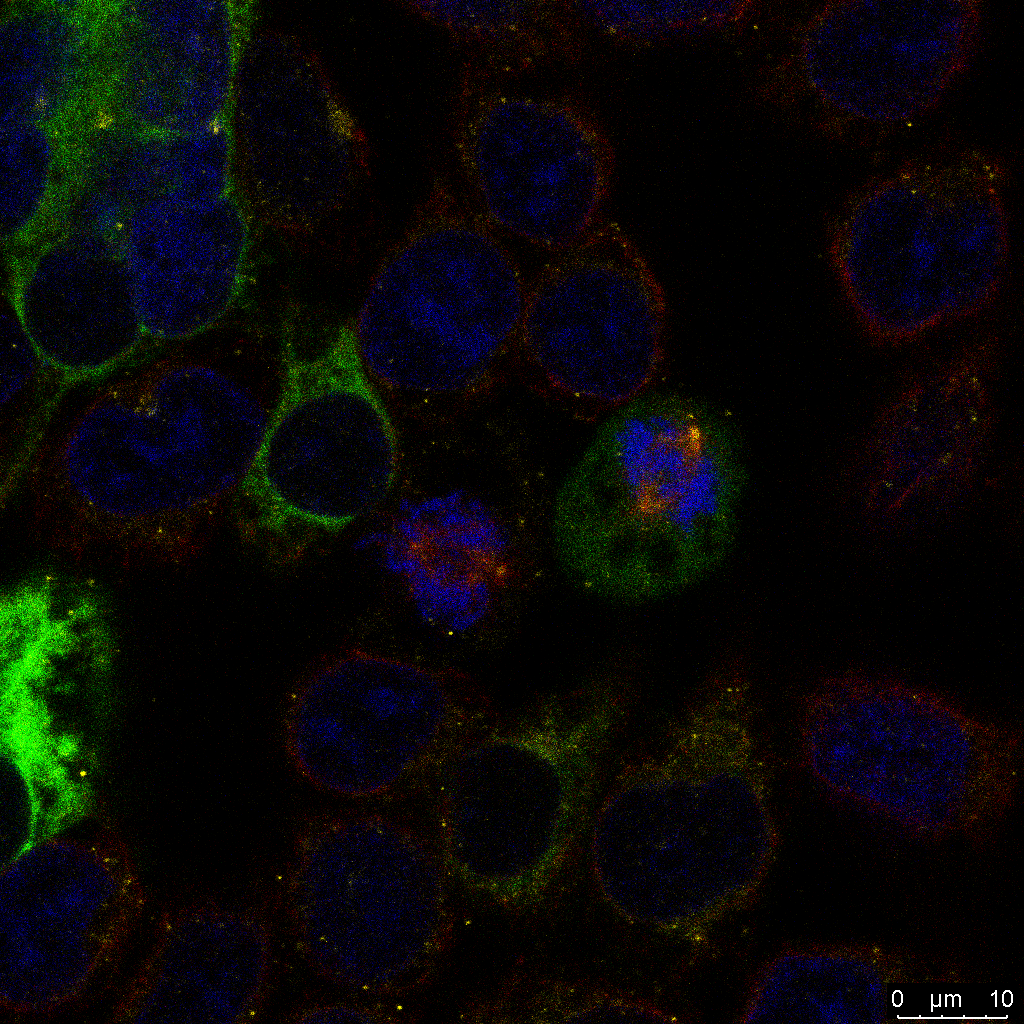

Supplement: Supplementary file 7 — Source data Fig. 5 [file 44319_2025_438_MOESM7_ESM.zip › SD figure 5/Fig. 5E/GFP-K98R-1.35.tif]

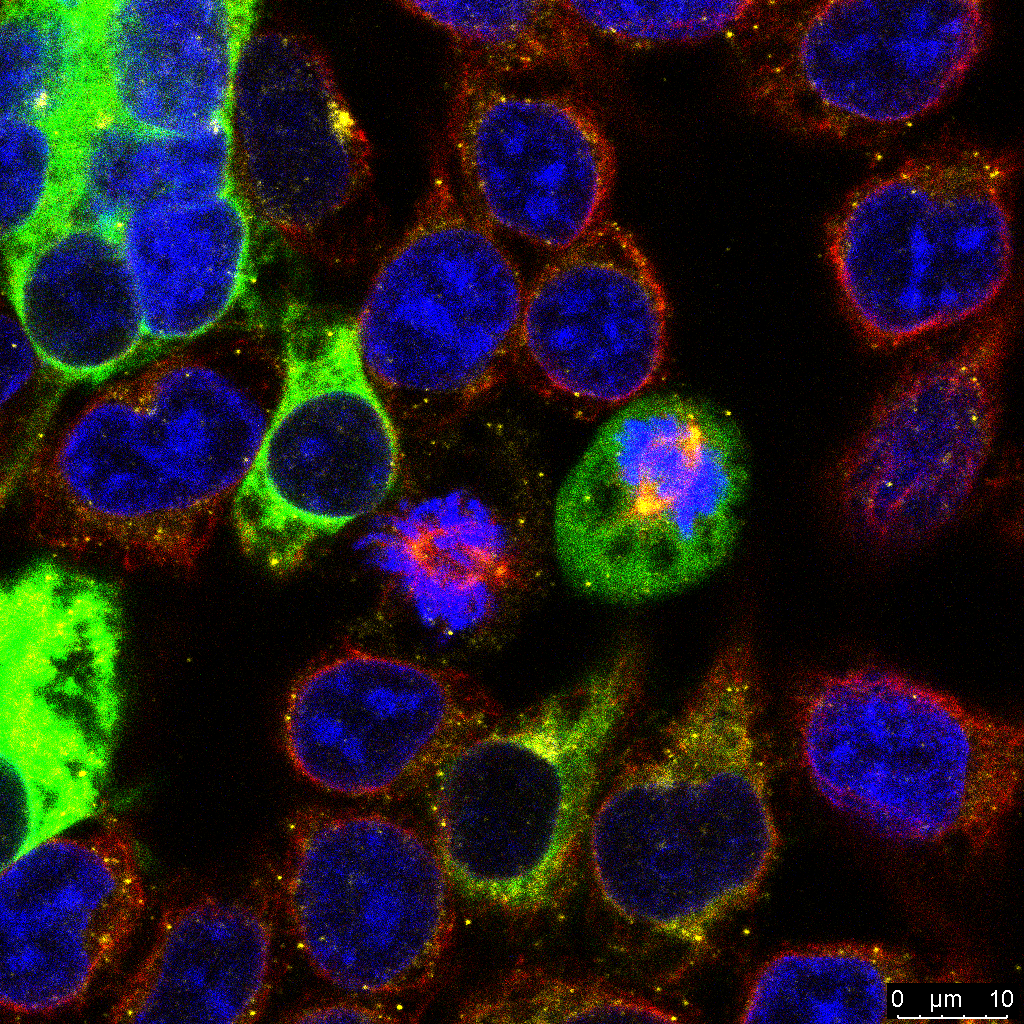

Supplement: Supplementary file 7 — Source data Fig. 5 [file 44319_2025_438_MOESM7_ESM.zip › SD figure 5/Fig. 5E/GFP-K98R-3D.tif]

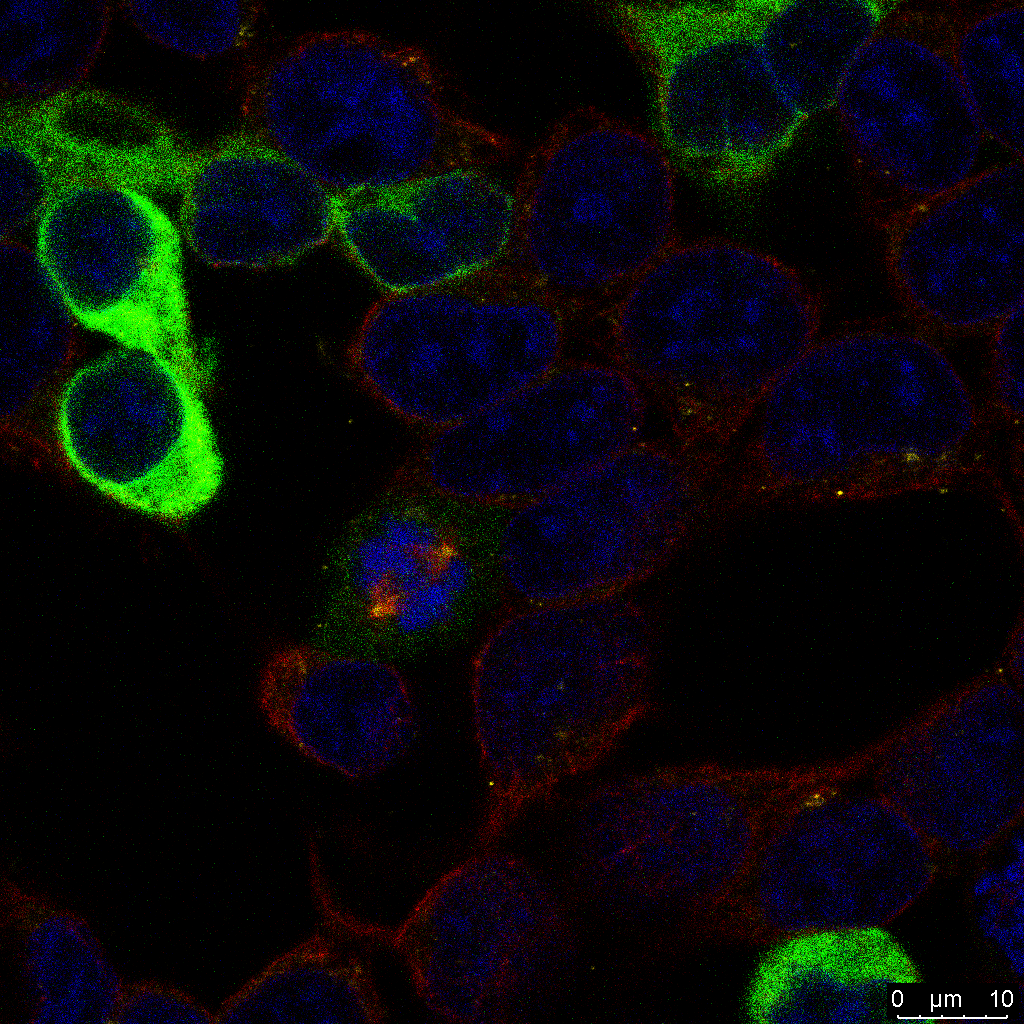

Supplement: Supplementary file 7 — Source data Fig. 5 [file 44319_2025_438_MOESM7_ESM.zip › SD figure 5/Fig. 5E/GFP-WT-0.45.tif]

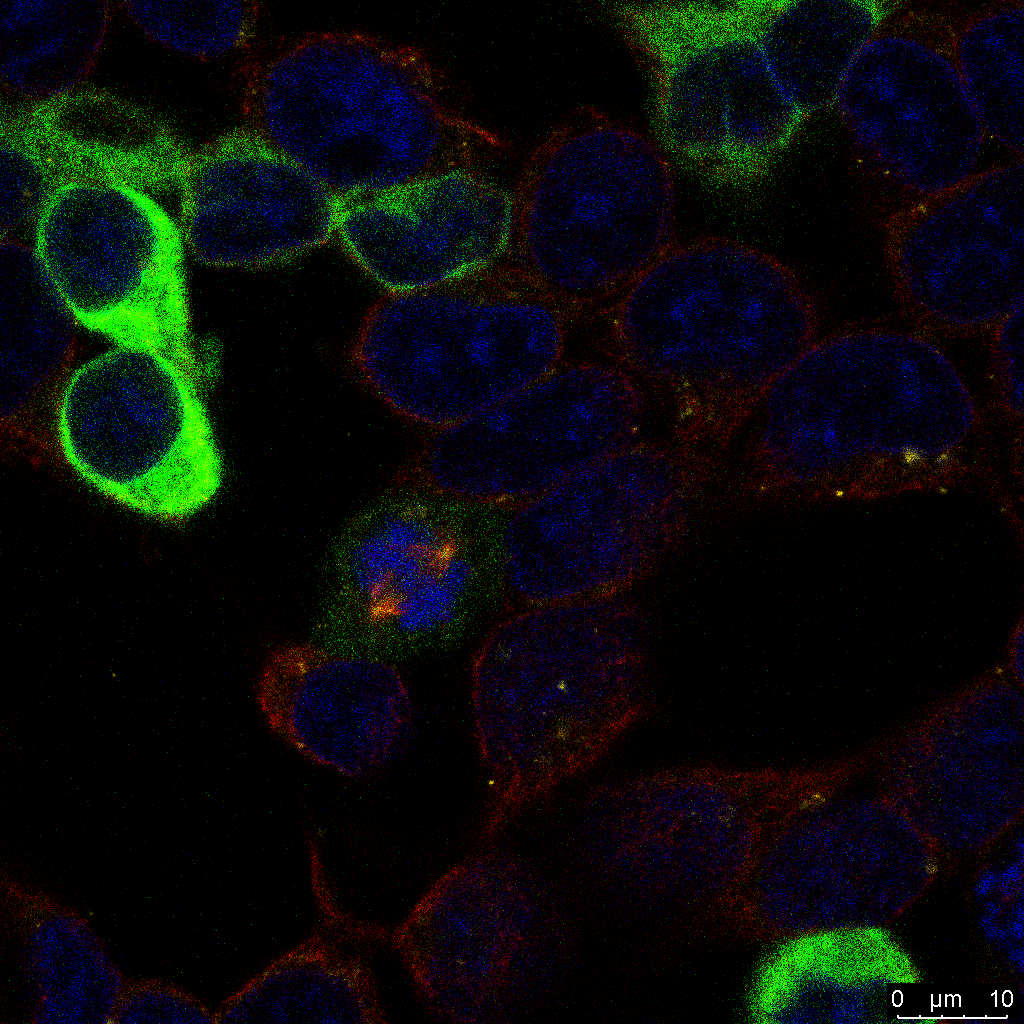

Supplement: Supplementary file 7 — Source data Fig. 5 [file 44319_2025_438_MOESM7_ESM.zip › SD figure 5/Fig. 5E/GFP-WT-0.9.tif]

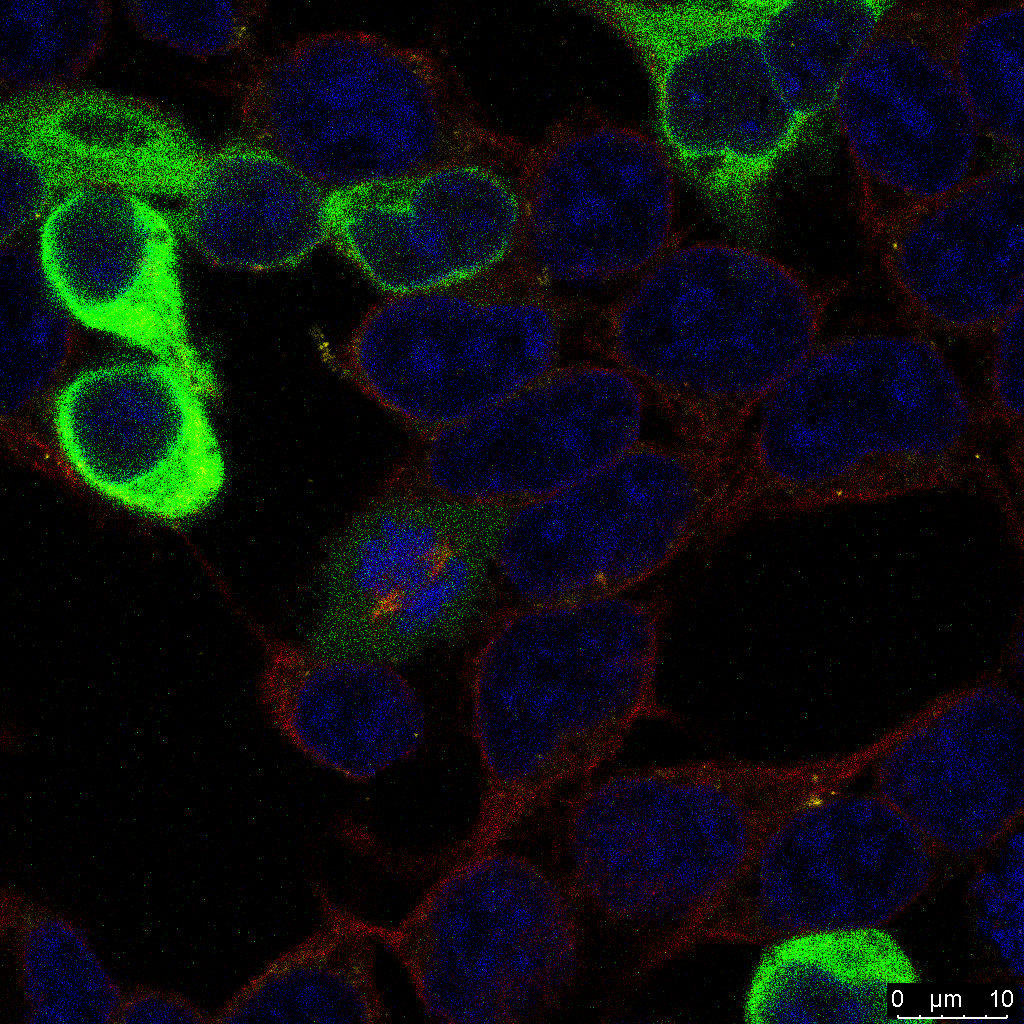

Supplement: Supplementary file 7 — Source data Fig. 5 [file 44319_2025_438_MOESM7_ESM.zip › SD figure 5/Fig. 5E/GFP-WT-0.tif]

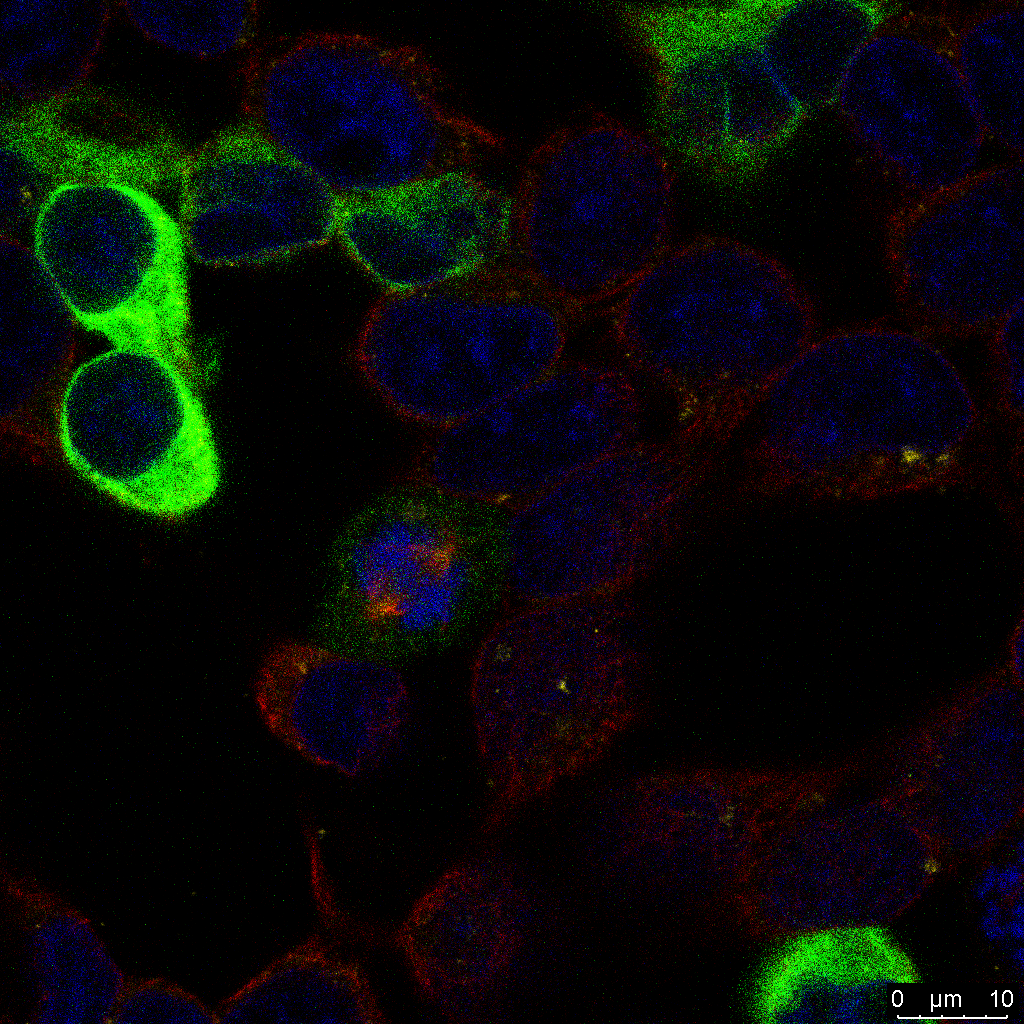

Supplement: Supplementary file 7 — Source data Fig. 5 [file 44319_2025_438_MOESM7_ESM.zip › SD figure 5/Fig. 5E/GFP-WT-1.35.tif]

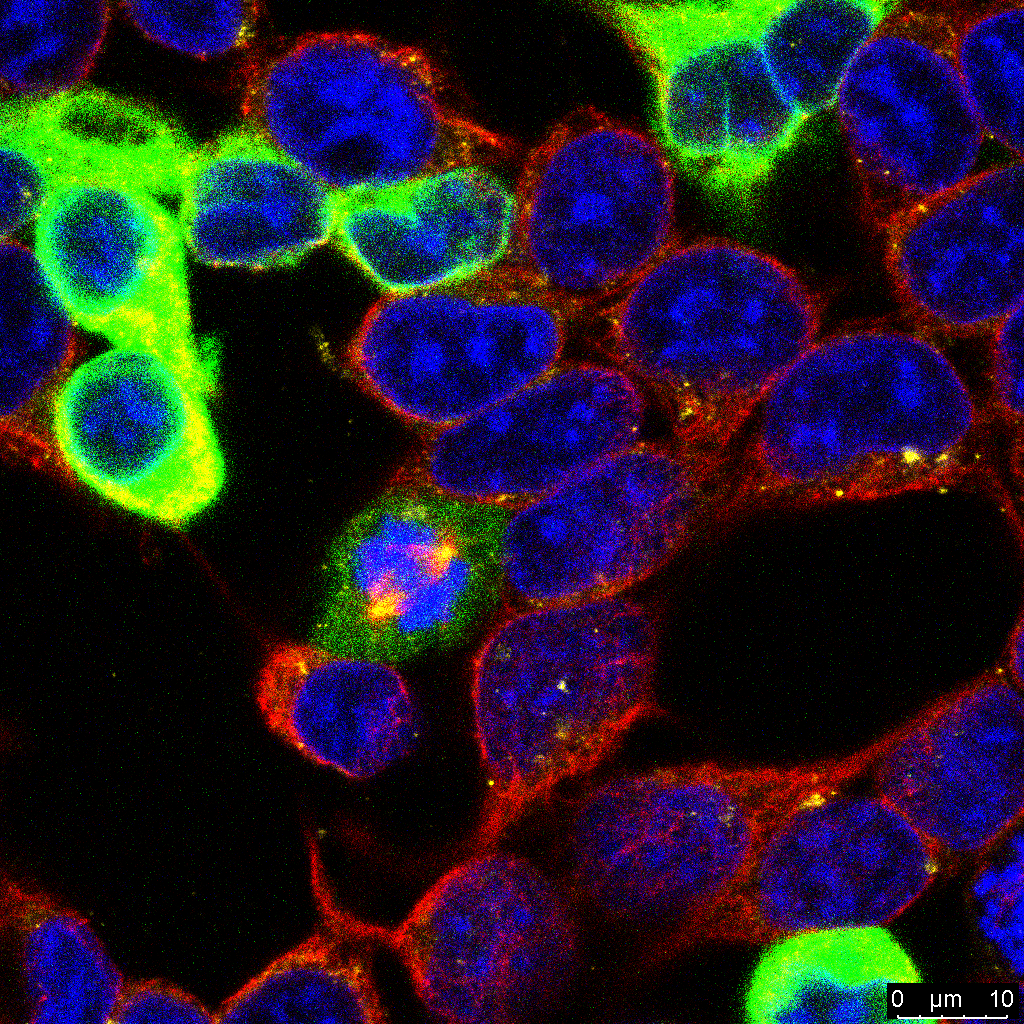

Supplement: Supplementary file 7 — Source data Fig. 5 [file 44319_2025_438_MOESM7_ESM.zip › SD figure 5/Fig. 5E/GFP-WT-3D.tif]

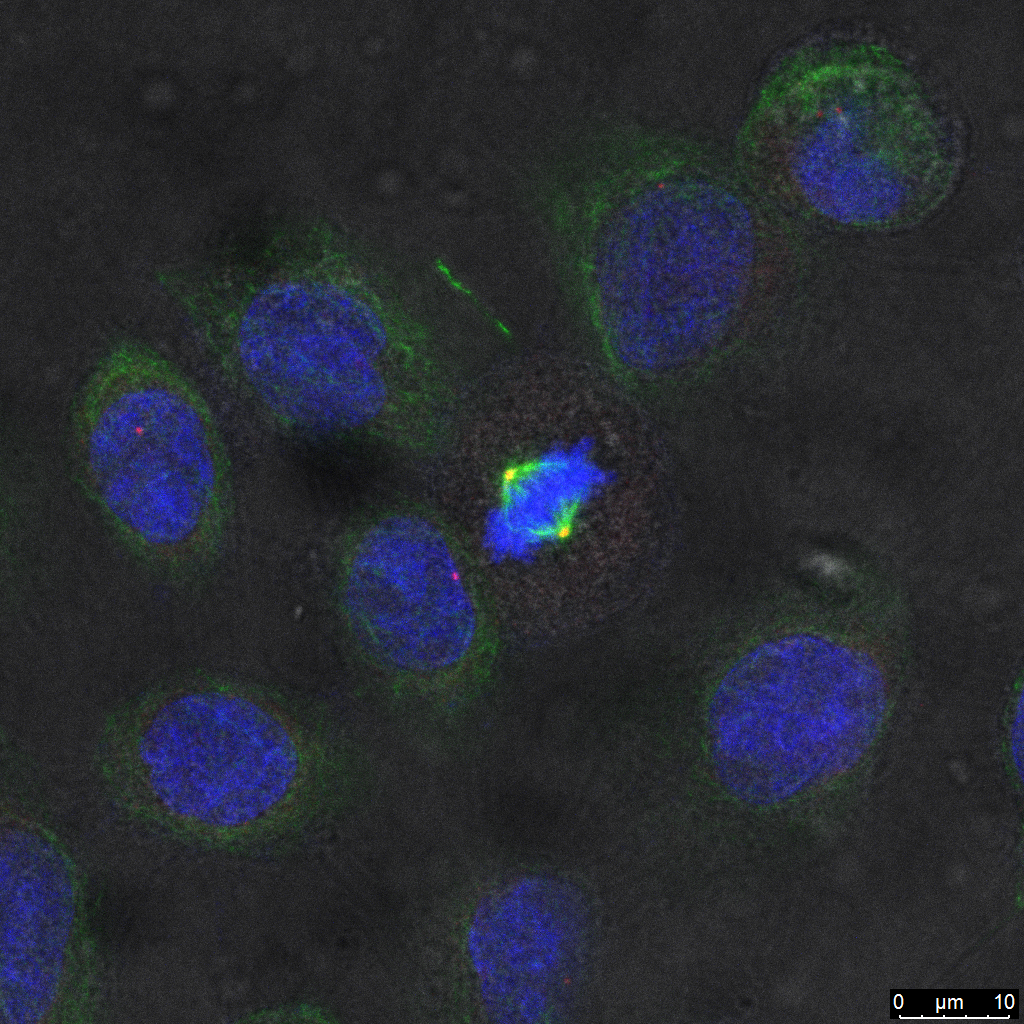

Supplement: Supplementary file 7 — Source data Fig. 5 [file 44319_2025_438_MOESM7_ESM.zip › SD figure 5/Fig. 5H/siControl.tif]

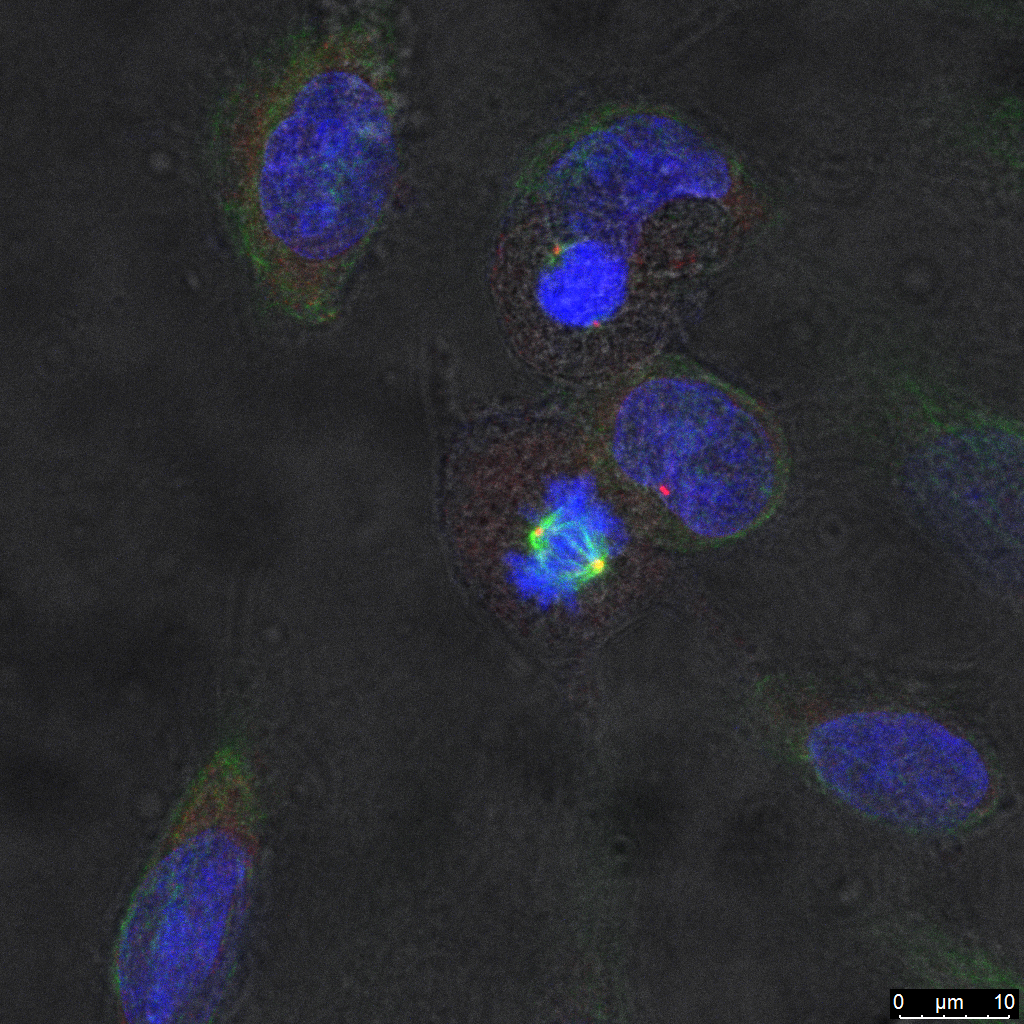

Supplement: Supplementary file 7 — Source data Fig. 5 [file 44319_2025_438_MOESM7_ESM.zip › SD figure 5/Fig. 5H/siENKD1#1.tif]

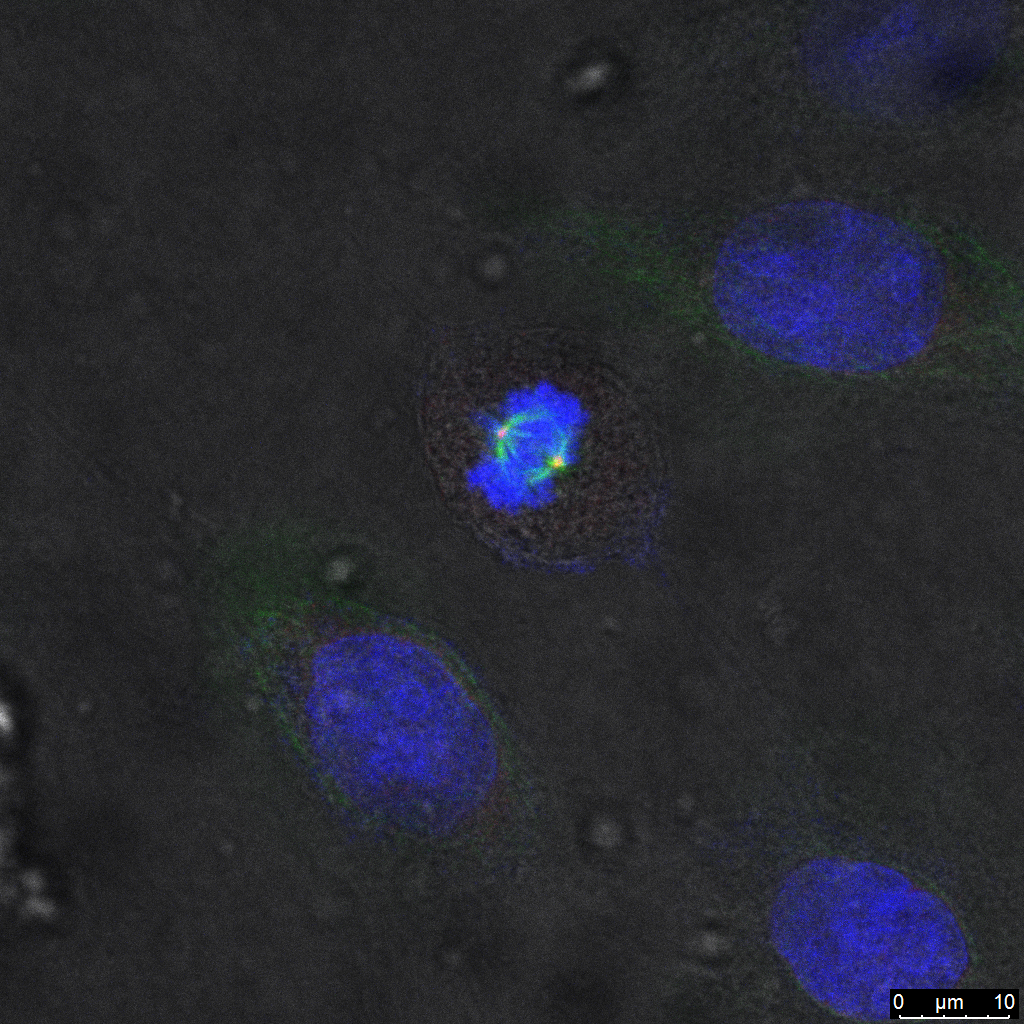

Supplement: Supplementary file 7 — Source data Fig. 5 [file 44319_2025_438_MOESM7_ESM.zip › SD figure 5/Fig. 5H/siENKD1#2.tif]

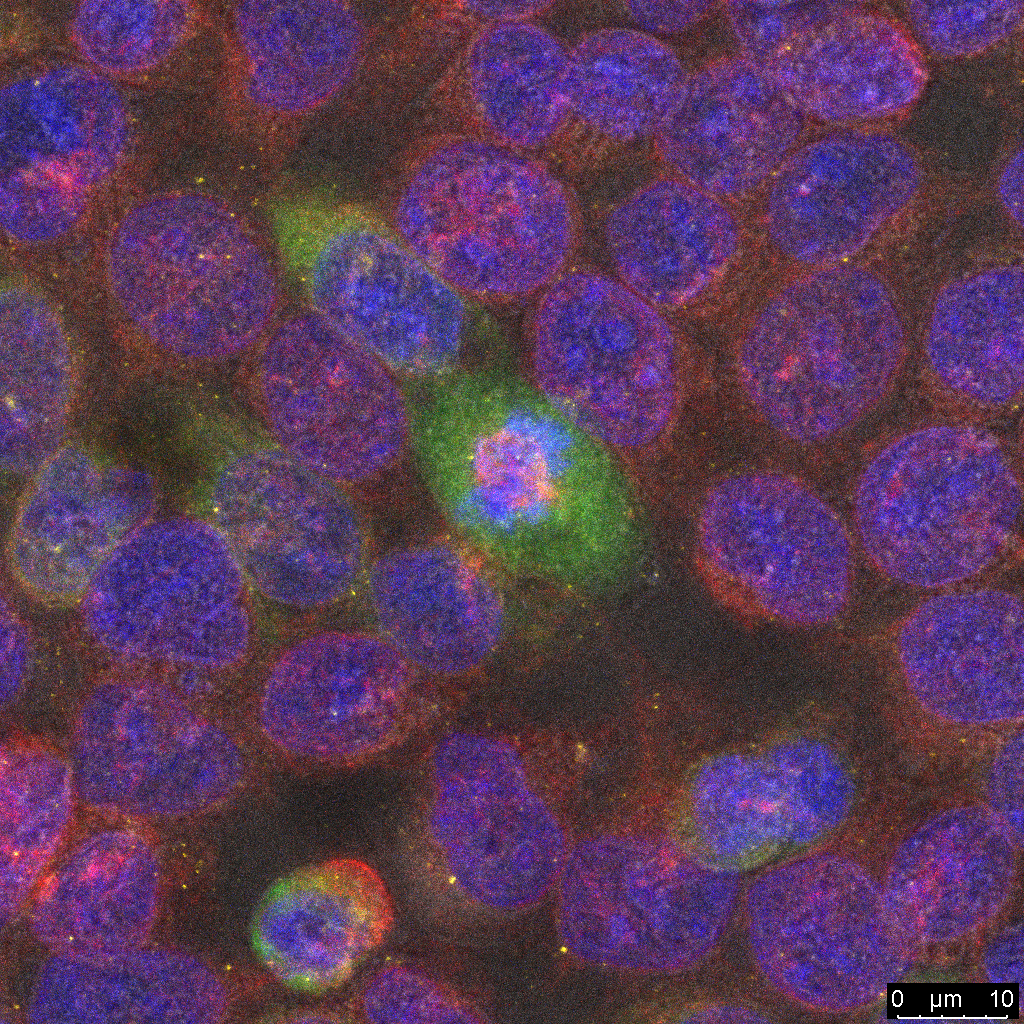

Supplement: Supplementary file 7 — Source data Fig. 5 [file 44319_2025_438_MOESM7_ESM.zip › SD figure 5/Fig. 5K/GFP-K98Q.tif]

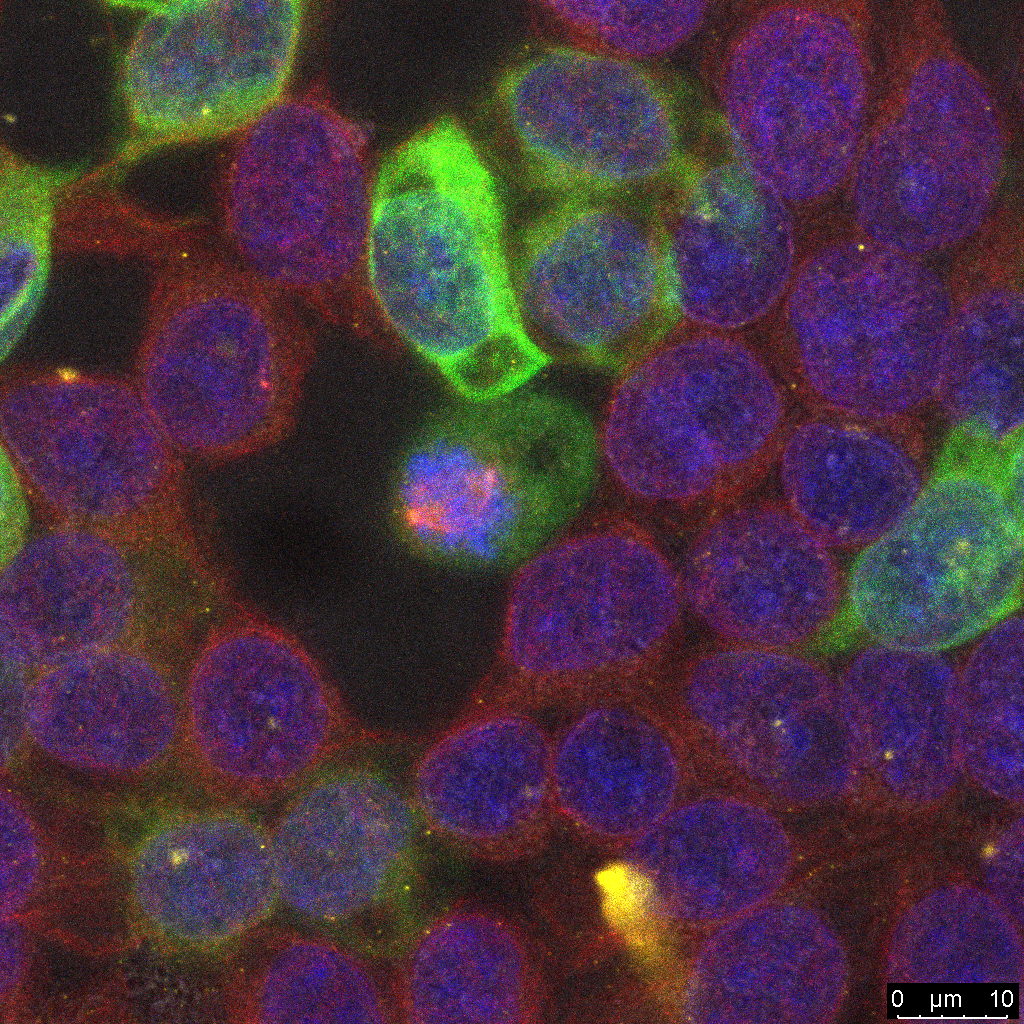

Supplement: Supplementary file 7 — Source data Fig. 5 [file 44319_2025_438_MOESM7_ESM.zip › SD figure 5/Fig. 5K/GFP-K98R.tif]

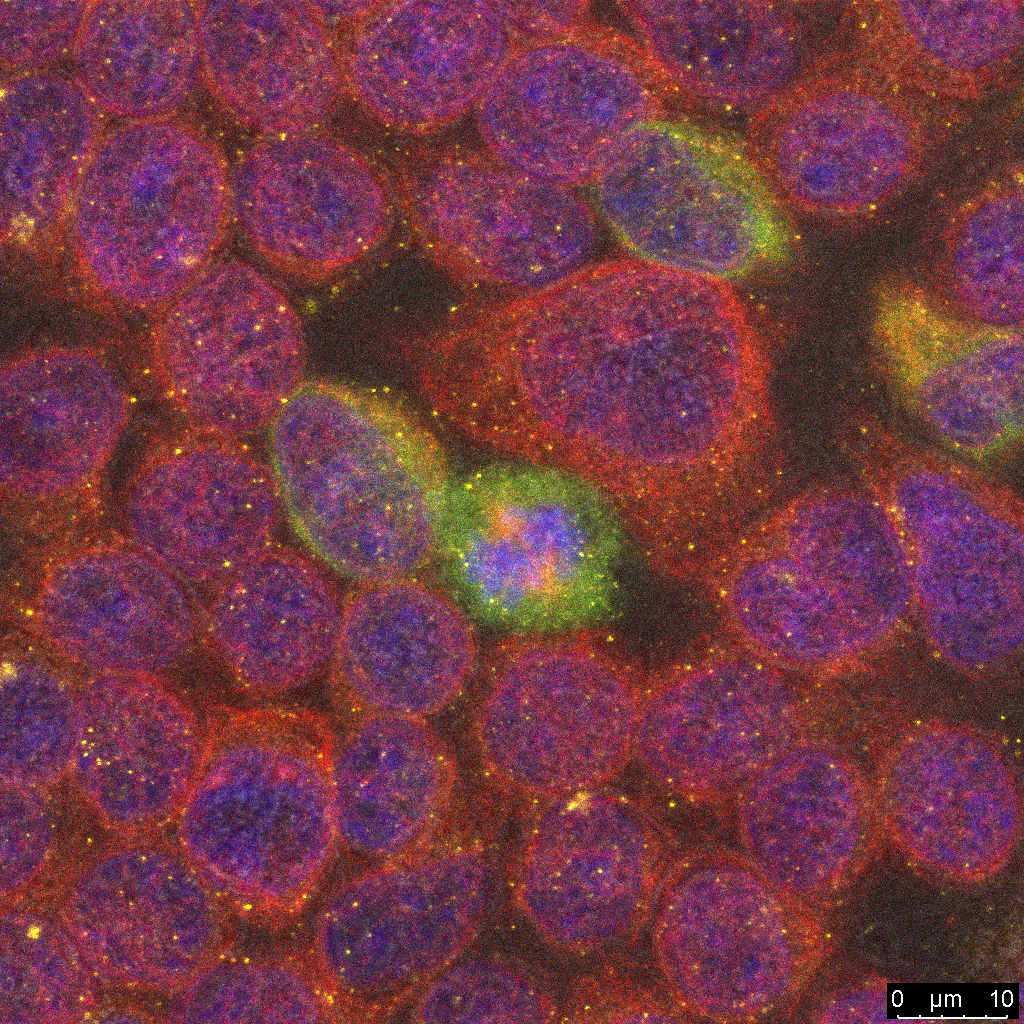

Supplement: Supplementary file 7 — Source data Fig. 5 [file 44319_2025_438_MOESM7_ESM.zip › SD figure 5/Fig. 5K/GFP-WT.tif]

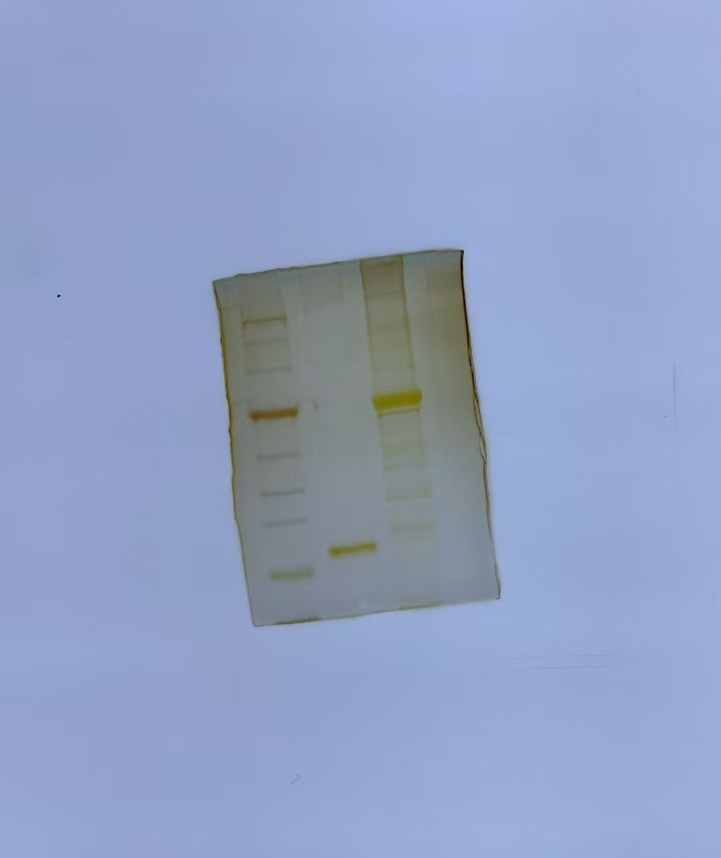

Supplement: Supplementary file 8 — Source data Fig. 6 [file 44319_2025_438_MOESM8_ESM.zip › SD figure 6/Fig. 6A/Fig. 6A.jpg]

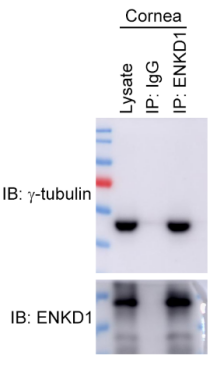

Supplement: Supplementary file 8 — Source data Fig. 6 [file 44319_2025_438_MOESM8_ESM.zip › SD figure 6/Fig. 6C/Fig. 6C.png]

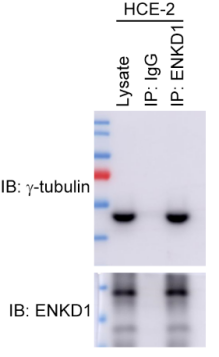

Supplement: Supplementary file 8 — Source data Fig. 6 [file 44319_2025_438_MOESM8_ESM.zip › SD figure 6/Fig. 6D/Fig. 6D.png]

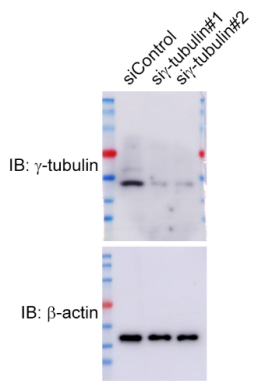

Supplement: Supplementary file 8 — Source data Fig. 6 [file 44319_2025_438_MOESM8_ESM.zip › SD figure 6/Fig. 6E/Fig. 6E.png]

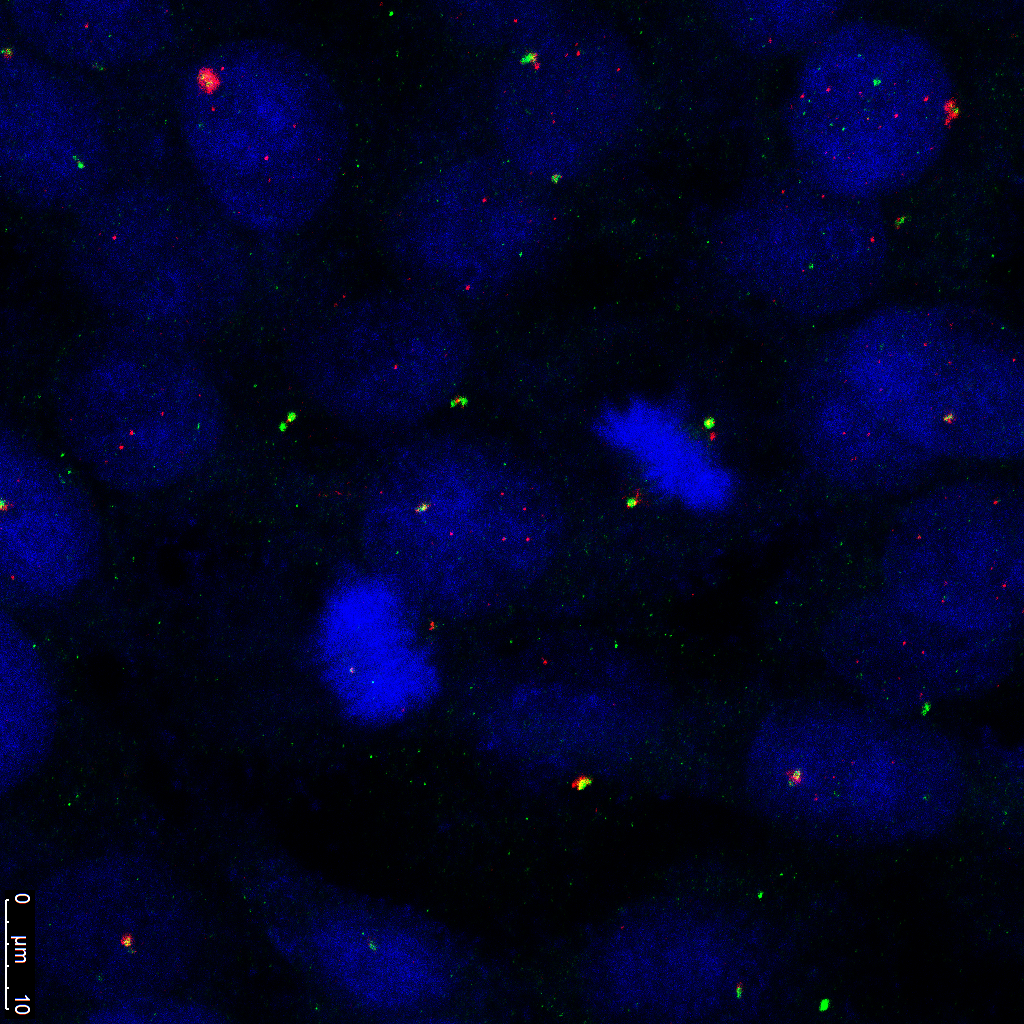

Supplement: Supplementary file 8 — Source data Fig. 6 [file 44319_2025_438_MOESM8_ESM.zip › SD figure 6/Fig. 6F/siControl.tif]

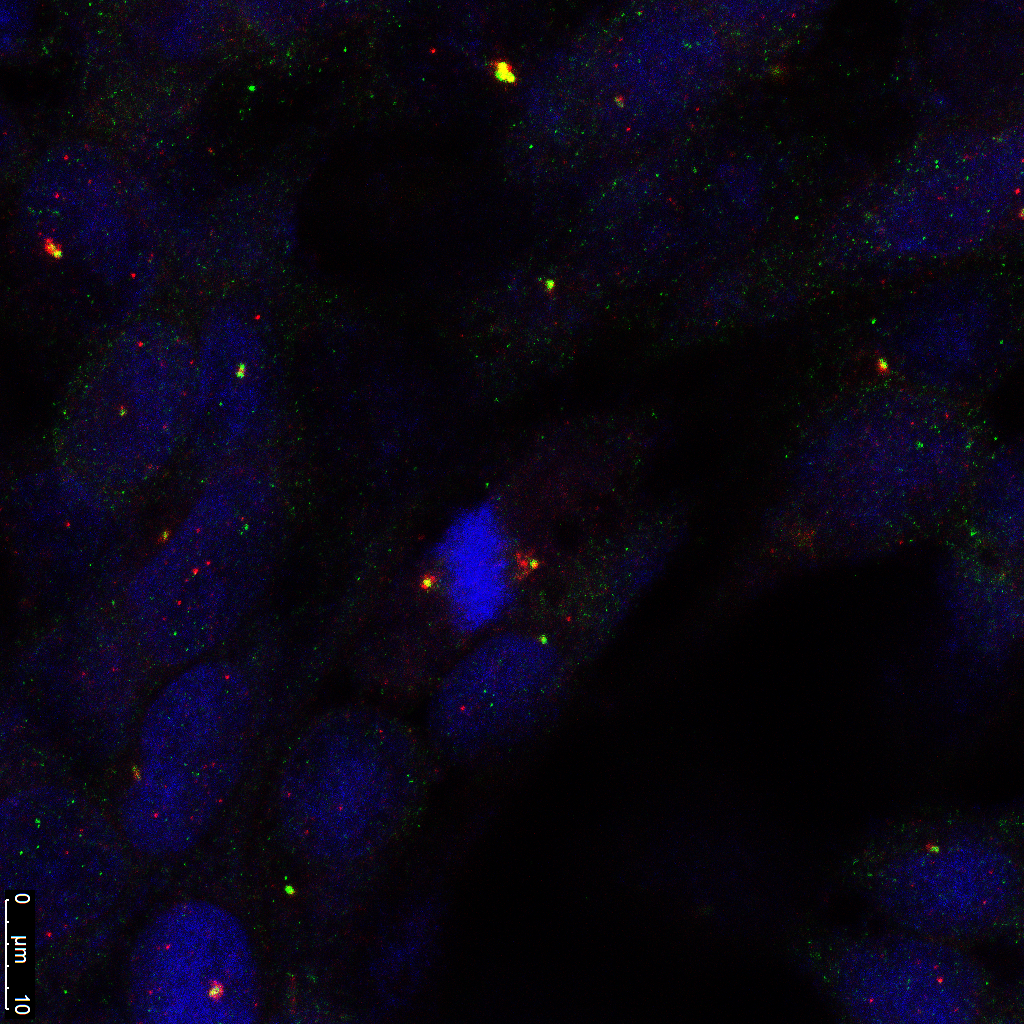

Supplement: Supplementary file 8 — Source data Fig. 6 [file 44319_2025_438_MOESM8_ESM.zip › SD figure 6/Fig. 6F/sia├-tubulin#1.tif]

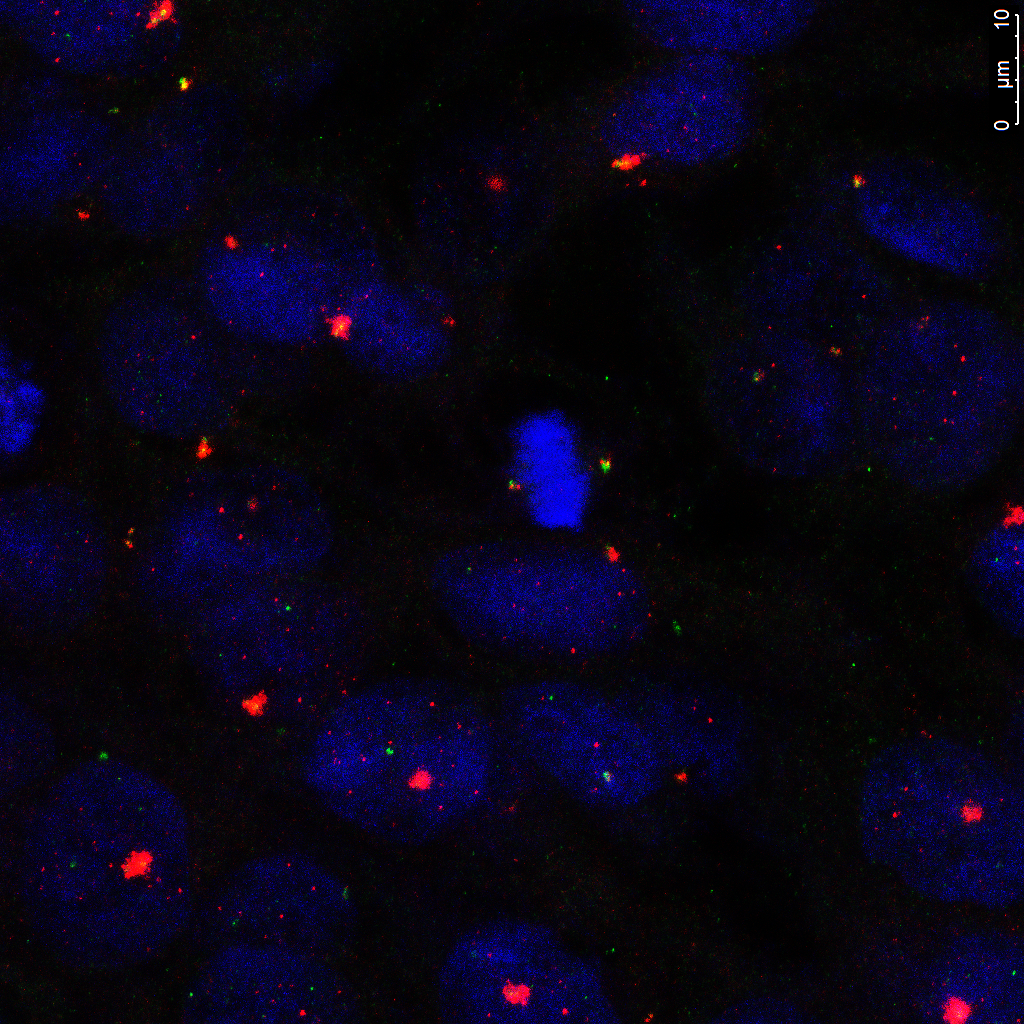

Supplement: Supplementary file 8 — Source data Fig. 6 [file 44319_2025_438_MOESM8_ESM.zip › SD figure 6/Fig. 6F/sia├-tubulin#2.tif]

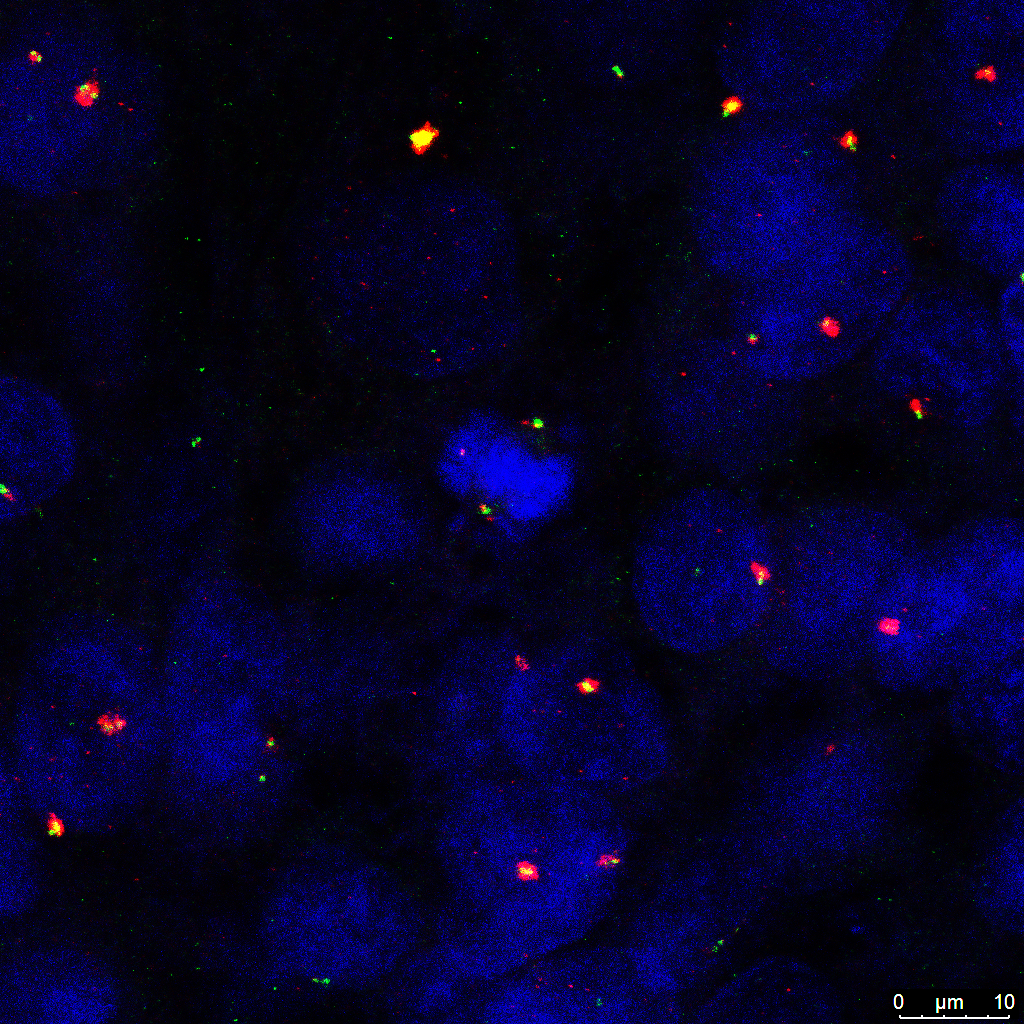

Supplement: Supplementary file 8 — Source data Fig. 6 [file 44319_2025_438_MOESM8_ESM.zip › SD figure 6/Fig. 6H/siControl.tif]

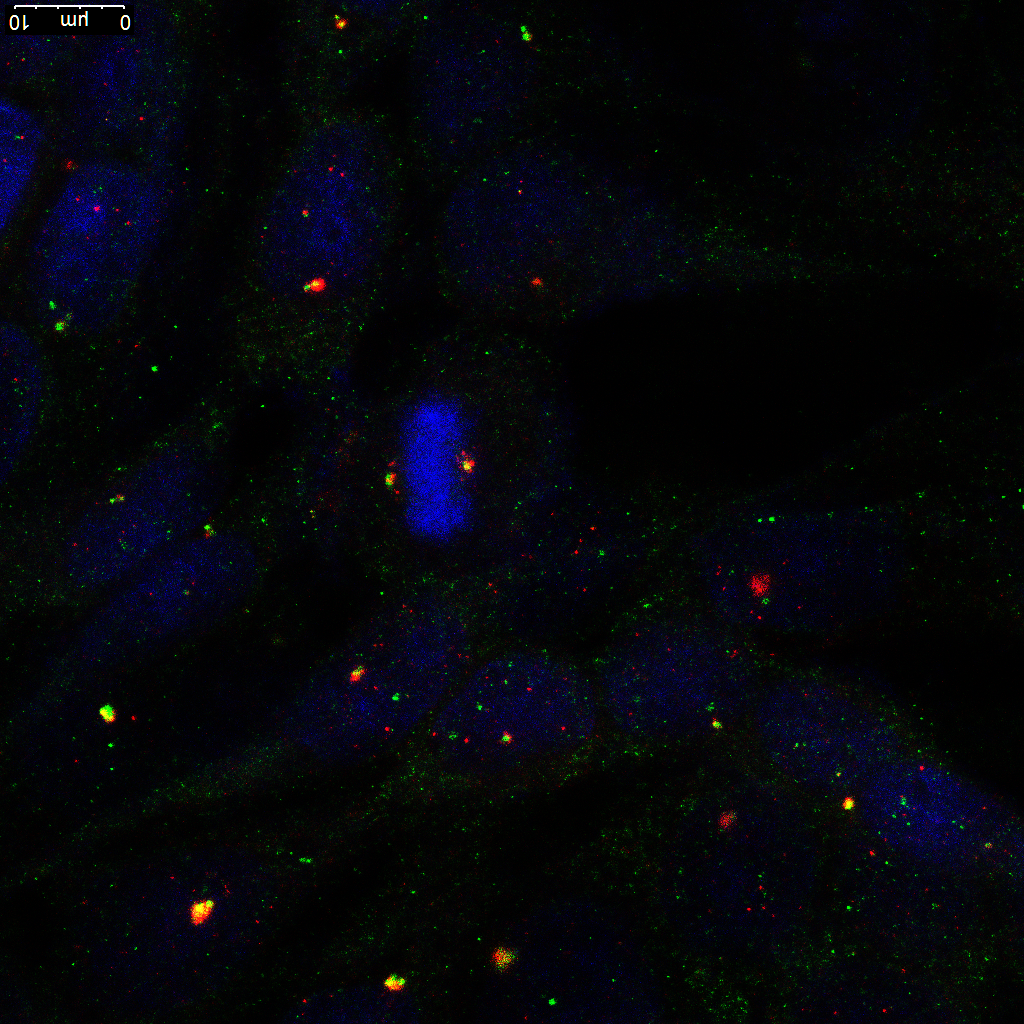

Supplement: Supplementary file 8 — Source data Fig. 6 [file 44319_2025_438_MOESM8_ESM.zip › SD figure 6/Fig. 6H/siENKD1#1.tif]

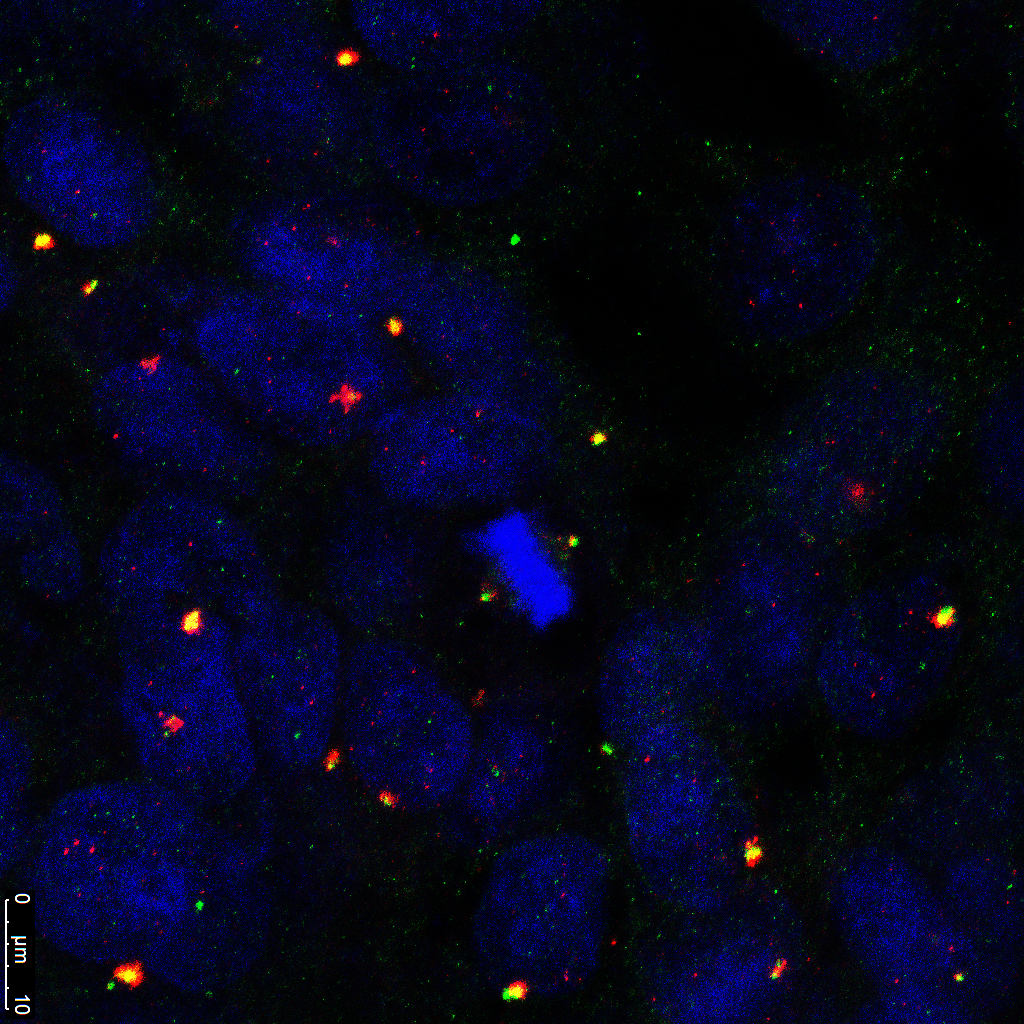

Supplement: Supplementary file 8 — Source data Fig. 6 [file 44319_2025_438_MOESM8_ESM.zip › SD figure 6/Fig. 6H/siENKD1#2.tif]

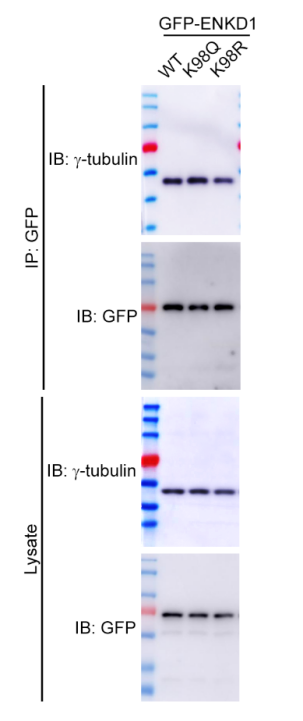

Supplement: Supplementary file 8 — Source data Fig. 6 [file 44319_2025_438_MOESM8_ESM.zip › SD figure 6/Fig. 6I/Fig. 6I.png]

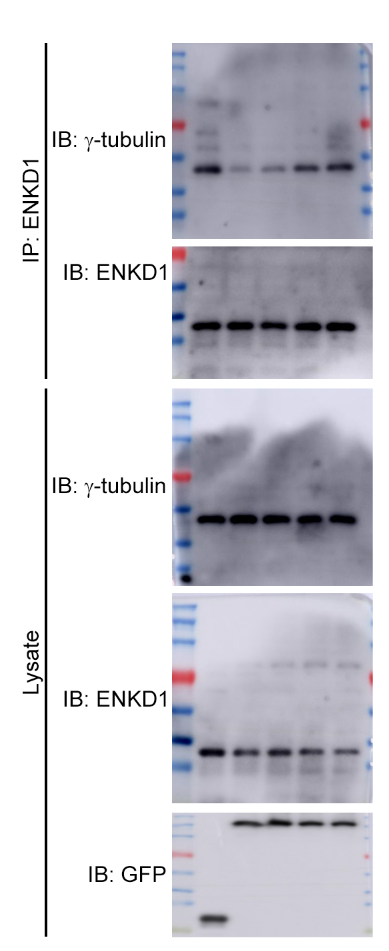

Supplement: Supplementary file 8 — Source data Fig. 6 [file 44319_2025_438_MOESM8_ESM.zip › SD figure 6/Fig. 6K/Fig. 6K.png]

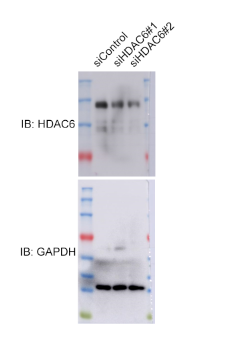

Supplement: Supplementary file 9 — EV Figures Source Data [file 44319_2025_438_MOESM9_ESM.zip › EV_Figures_Source data/Appendix S1/Fig. S1A/Fig. S1A.png]

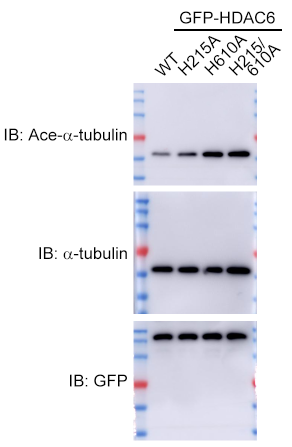

Supplement: Supplementary file 9 — EV Figures Source Data [file 44319_2025_438_MOESM9_ESM.zip › EV_Figures_Source data/Appendix S2/Fig. S2A/Fig. S2A.png]

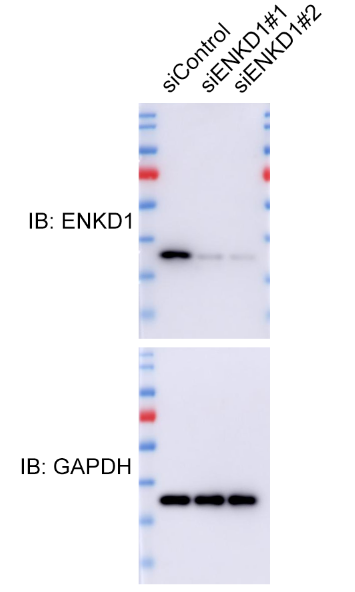

Supplement: Supplementary file 9 — EV Figures Source Data [file 44319_2025_438_MOESM9_ESM.zip › EV_Figures_Source data/Appendix S4/Fig. S4A/Fig. S4A.png]

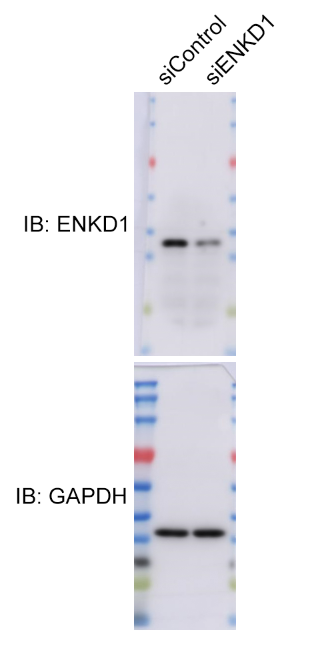

Supplement: Supplementary file 9 — EV Figures Source Data [file 44319_2025_438_MOESM9_ESM.zip › EV_Figures_Source data/Appendix S4/Fig. S4D/Fig. S4D.png]

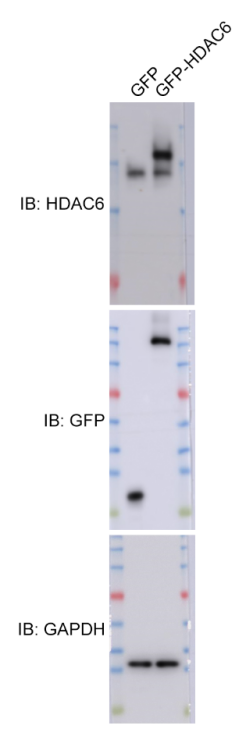

Supplement: Supplementary file 9 — EV Figures Source Data [file 44319_2025_438_MOESM9_ESM.zip › EV_Figures_Source data/Figure EV1/Fig. EV1G/Fig. EV1G.png]

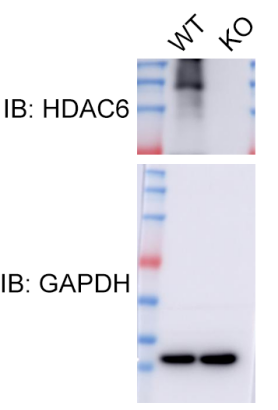

Supplement: Supplementary file 9 — EV Figures Source Data [file 44319_2025_438_MOESM9_ESM.zip › EV_Figures_Source data/Figure EV1/Fig. EV1L/Fig. EV1L.png]

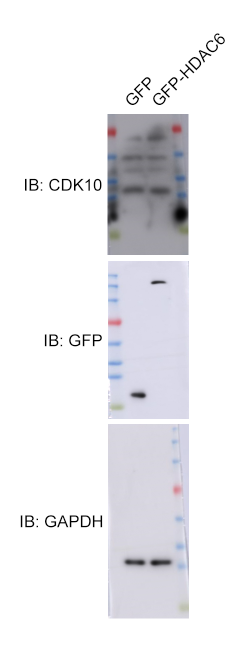

Supplement: Supplementary file 9 — EV Figures Source Data [file 44319_2025_438_MOESM9_ESM.zip › EV_Figures_Source data/Figure EV2/Fig. EV2C/Fig. EV2C.png]

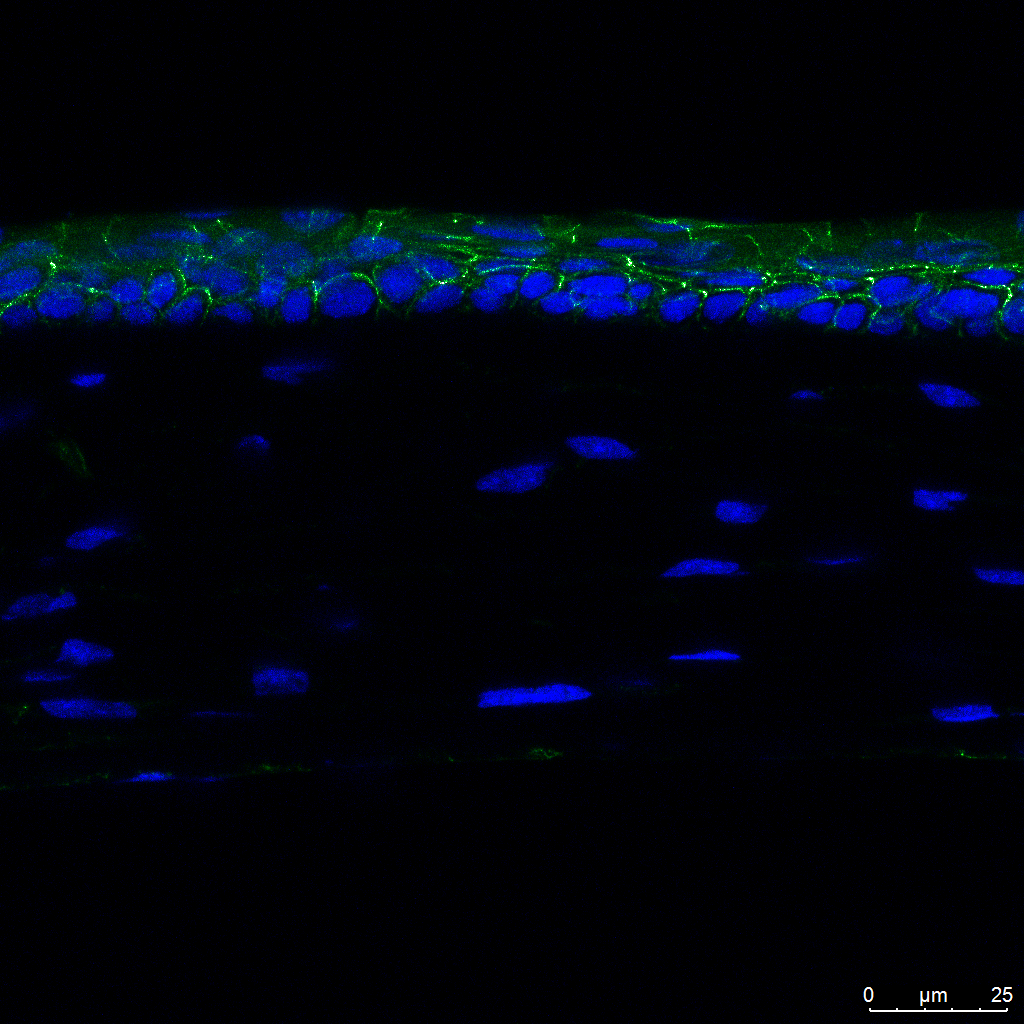

Supplement: Supplementary file 9 — EV Figures Source Data [file 44319_2025_438_MOESM9_ESM.zip › EV_Figures_Source data/Figure EV2/Fig. EV2H/KO.tif]

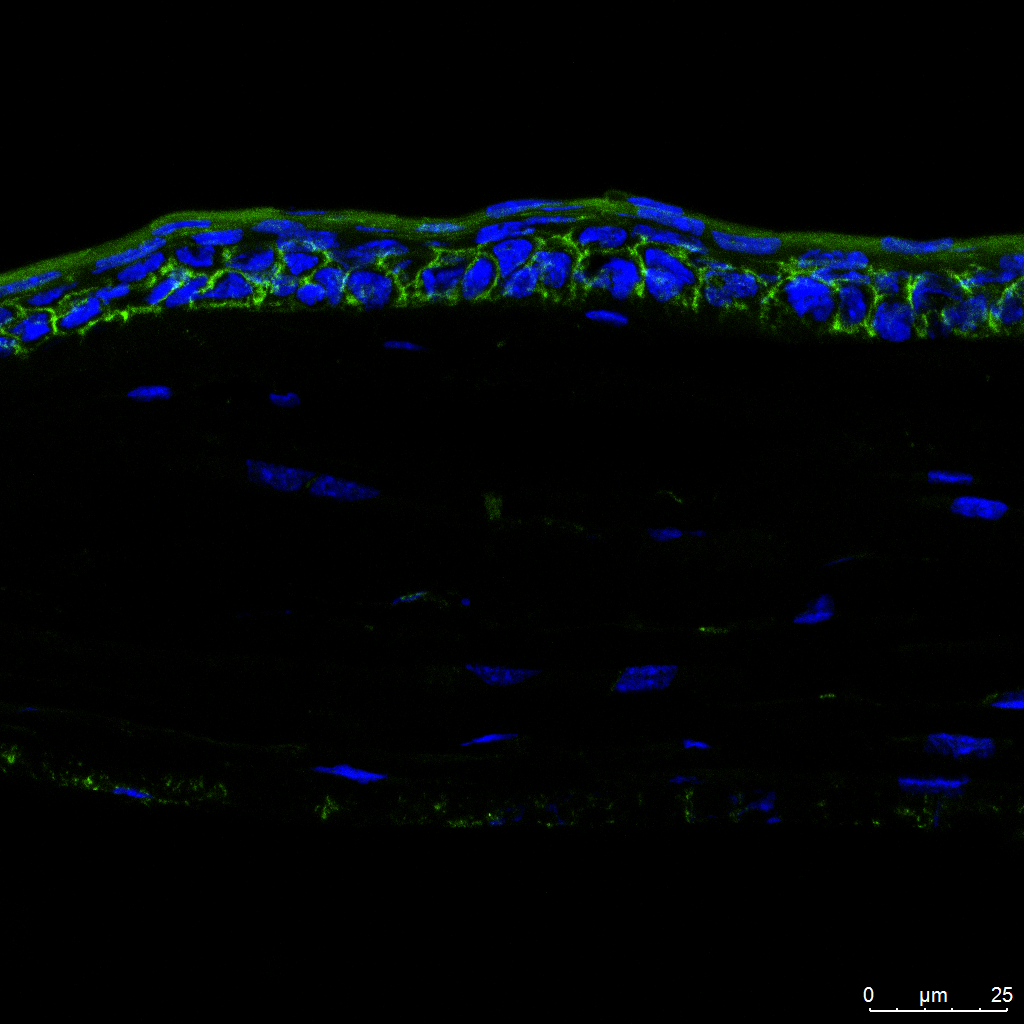

Supplement: Supplementary file 9 — EV Figures Source Data [file 44319_2025_438_MOESM9_ESM.zip › EV_Figures_Source data/Figure EV2/Fig. EV2H/WT.tif]

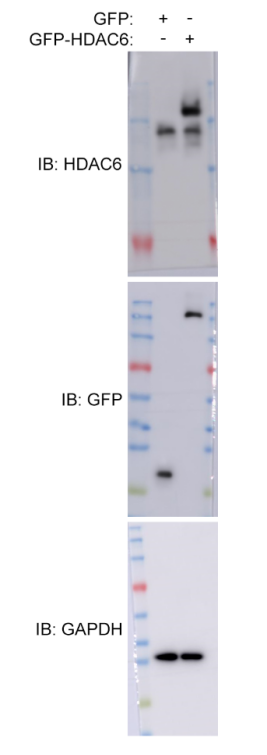

Supplement: Supplementary file 9 — EV Figures Source Data [file 44319_2025_438_MOESM9_ESM.zip › EV_Figures_Source data/Figure EV3/Fig. EV3A/Fig. EV3A.png]

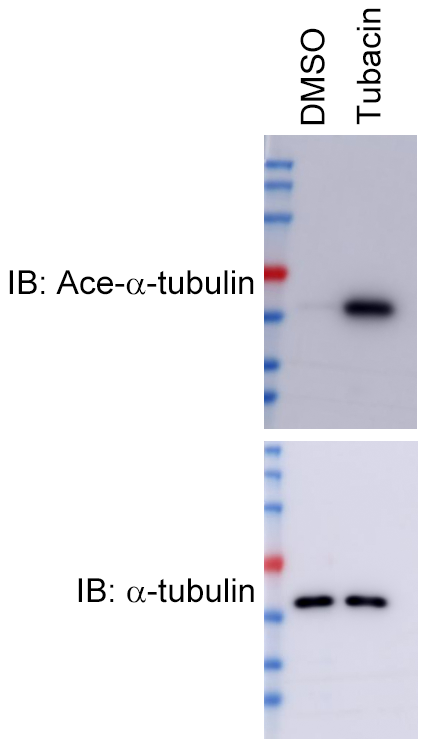

Supplement: Supplementary file 9 — EV Figures Source Data [file 44319_2025_438_MOESM9_ESM.zip › EV_Figures_Source data/Figure EV3/Fig. EV3F/Fig. EV3F.png]

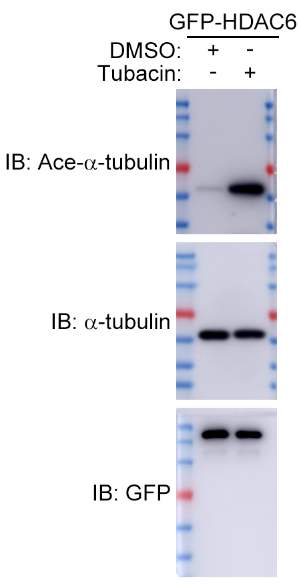

Supplement: Supplementary file 9 — EV Figures Source Data [file 44319_2025_438_MOESM9_ESM.zip › EV_Figures_Source data/Figure EV3/Fig. EV3M/Fig. EV3M.png]

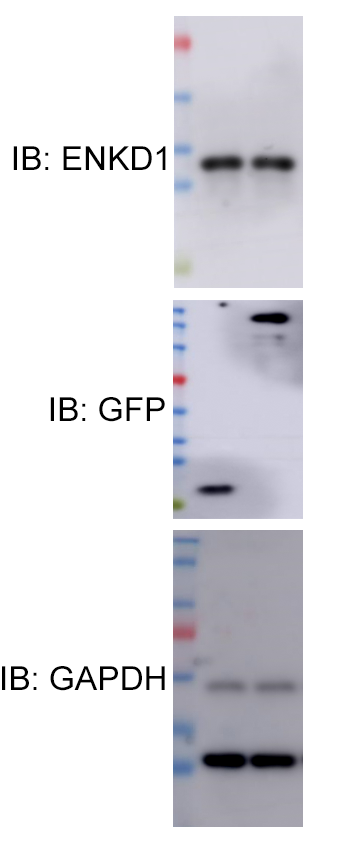

Supplement: Supplementary file 9 — EV Figures Source Data [file 44319_2025_438_MOESM9_ESM.zip › EV_Figures_Source data/Figure EV4/Fig. EV4F/Fig. EV4F.png]

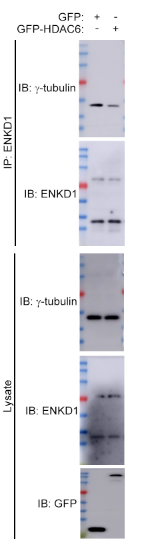

Supplement: Supplementary file 9 — EV Figures Source Data [file 44319_2025_438_MOESM9_ESM.zip › EV_Figures_Source data/Figure EV5/Fig. EV5B/Fig. EV5B.png]
