# Supplementary material for: HDAC6 deacetylates ENKD1 to regulate mitotic spindle behavior and corneal epithelial homeostasis
Source: EMBO Rep. 2025 Mar 28;26(10):2597–621. doi: 10.1038/s44319-025-00438-0 (PMC12116779; doi:10.1038/s44319-025-00438-0)
Supplement: Supplementary file 10 — Expanded View Figures [file 44319_2025_438_MOESM10_ESM.pdf]

Expanded View Figures

Figure EV1. Depletion of HDAC6 does not significantly affect the corneal epithelium.

(**A**) Volcano plots showing the number of genes differentially regulated between healthy ($n = 3$ samples), KC ($n = 3$ samples), BK ($n = 3$ samples), and VK ($n = 3$ samples) corneas. Comparisons between the three different experimental groups are shown (healthy *vs.* KC, healthy *vs.* BK, and healthy *vs.* VK). The blue dots represent downregulated genes and the red dots represent upregulated genes. (**B**) Top 9 enriched terms in the GO analysis of differentially expressed genes listed in the three different experimental groups (healthy vs KC, healthy vs BK, healthy vs VK). (**C–E**) Quantification of *Il-6*, *Il-10*, and *Il-1β* mRNA levels by RT-qPCR in corneas from healthy or BK mice ($n = 3$ independent experiments). All data are normalized to *Gapdh* mRNA levels. *Il-6*, $P = 0.0004$; *Il-10*, $P = 0.006$; *Il-1β*, $P = 0.0006$. (**F**) Immunofluorescence images of the cornea from control or HDAC6 adenovirus-injected mice. Scale bar, 30 μm. (**G**, **H**) Immunoblot analysis of HDAC6, GFP, and GAPDH in corneas from control or HDAC6 adenovirus-injected mice. The red arrow indicates GFP-HDAC6, and the green arrow indicates endogenous HDAC6 (**G**). The relative levels of GFP-HDAC6 and endogenous HDAC6 were determined by densitometry (**H**, $n = 3$ independent experiments) (**I**, **J**) Quantification of the thickness of the stroma (**I**, $n = 30$ images from three independent experiments) and endothelium (**J**, $n = 30$ images from three independent experiments) from control or HDAC6 adenovirus-injected mice. Scale bar, 15 μm. (**K**) Schematic illustration of the strategy used for generating *Hdac6* knockout mice. Exons 10–13 of the mouse *Hdac6* gene were replaced by a vector containing neomycin (Neo) and zeocin (Zeo) cassettes. (**L**) Immunoblotting of HDAC6 and GAPDH in wild-type and *Hdac6* knockout mice. (**M–P**) H&E staining images (**M**) and quantification of the thickness of the epithelium (**N**, $n = 15$ images from three independent experiments), stroma (**O**, $n = 15$ images from three independent experiments) and endothelium (**P**, $n = 15$ images from three independent experiments) in wild-type and *Hdac6* knockout mice. Scale bar, 30 μm. Data are presented as mean ± SEM. Unpaired two-tailed Student's *t* test was performed for (**C**, **D**, **E**, **I**, **J**, **N**, **O**, **P**). $^{**}P < 0.01$; $^{***}P < 0.001$; ns, not significant. Differential gene expression analysis was performed using the DESeq2 package in R for (**A**). Enrichment analysis was performed using Metascape (http://www.metascape.org/) for (**B**). Source data are available online for this figure.

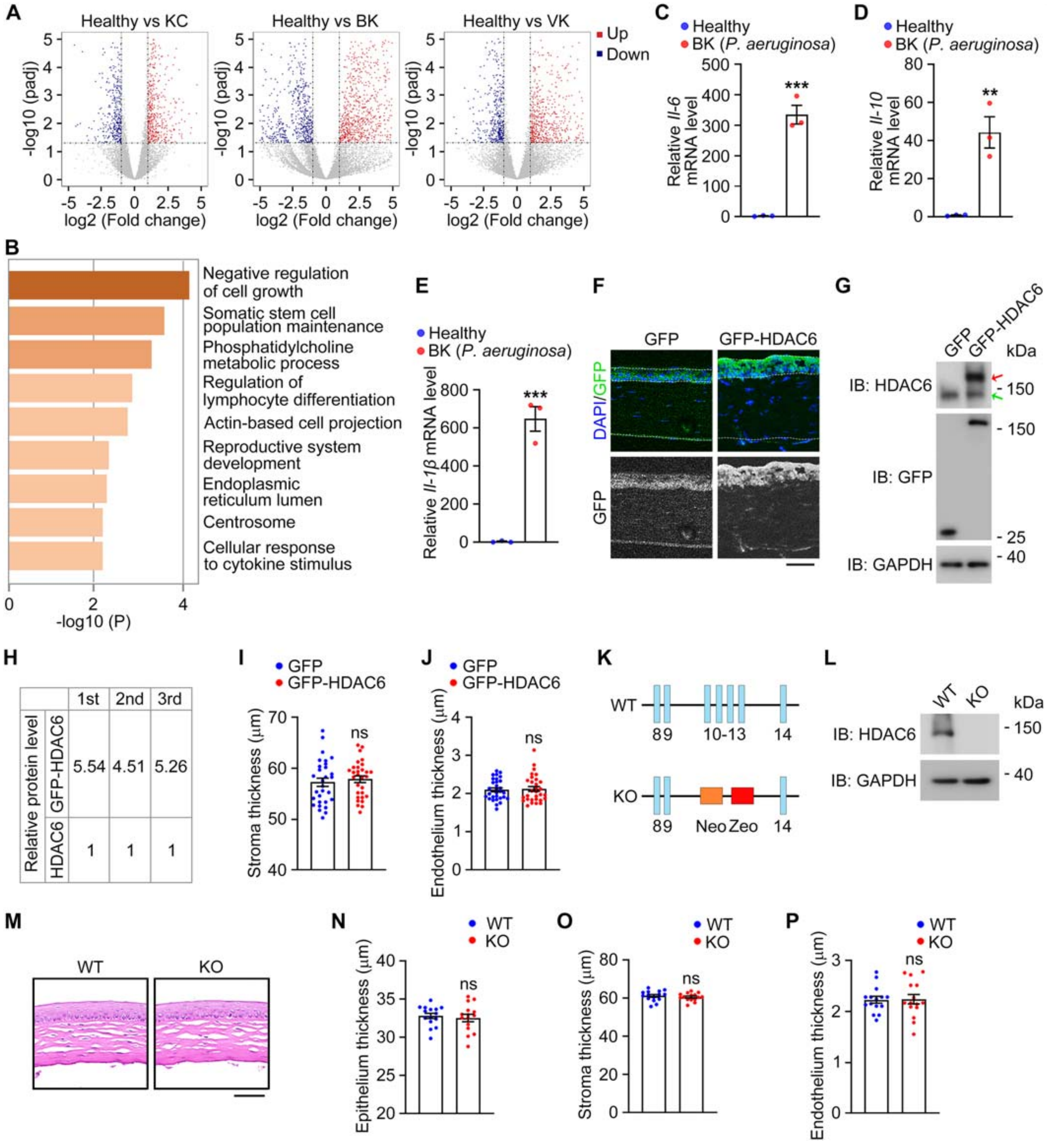
A
Healthy vs KC
Healthy vs BK
Healthy vs VK
Up
Down
-log10 (padj)
log2 (Fold change)
B
Negative regulation of cell growth
Somatic stem cell population maintenance
Phosphatidylcholine metabolic process
Regulation of lymphocyte differentiation
Actin-based cell projection
Reproductive system development
Endoplasmic reticulum lumen
Centrosome
Cellular response to cytokine stimulus
-log10 (P)
C
Healthy
BK (P. aeruginosa)
Relative Il-6 mRNA level
D
Relative Il-10 mRNA level
E
Relative Il-1β mRNA level
F
GFP
GFP-HDAC6
DAPI/GFP
G
kDa
IB: HDAC6
IB: GFP
IB: GAPDH
H
1st
2nd
3rd
Relative protein level
GFP-HDAC6 5.54 4.51 5.26
HDAC6 1 1 1
I
Stroma thickness (μm)
J
Endothelium thickness (μm)
ns
K
WT
KO
89
10-13
14
Neo
Zeo
L
M
N
Epithelium thickness (μm)
O
P

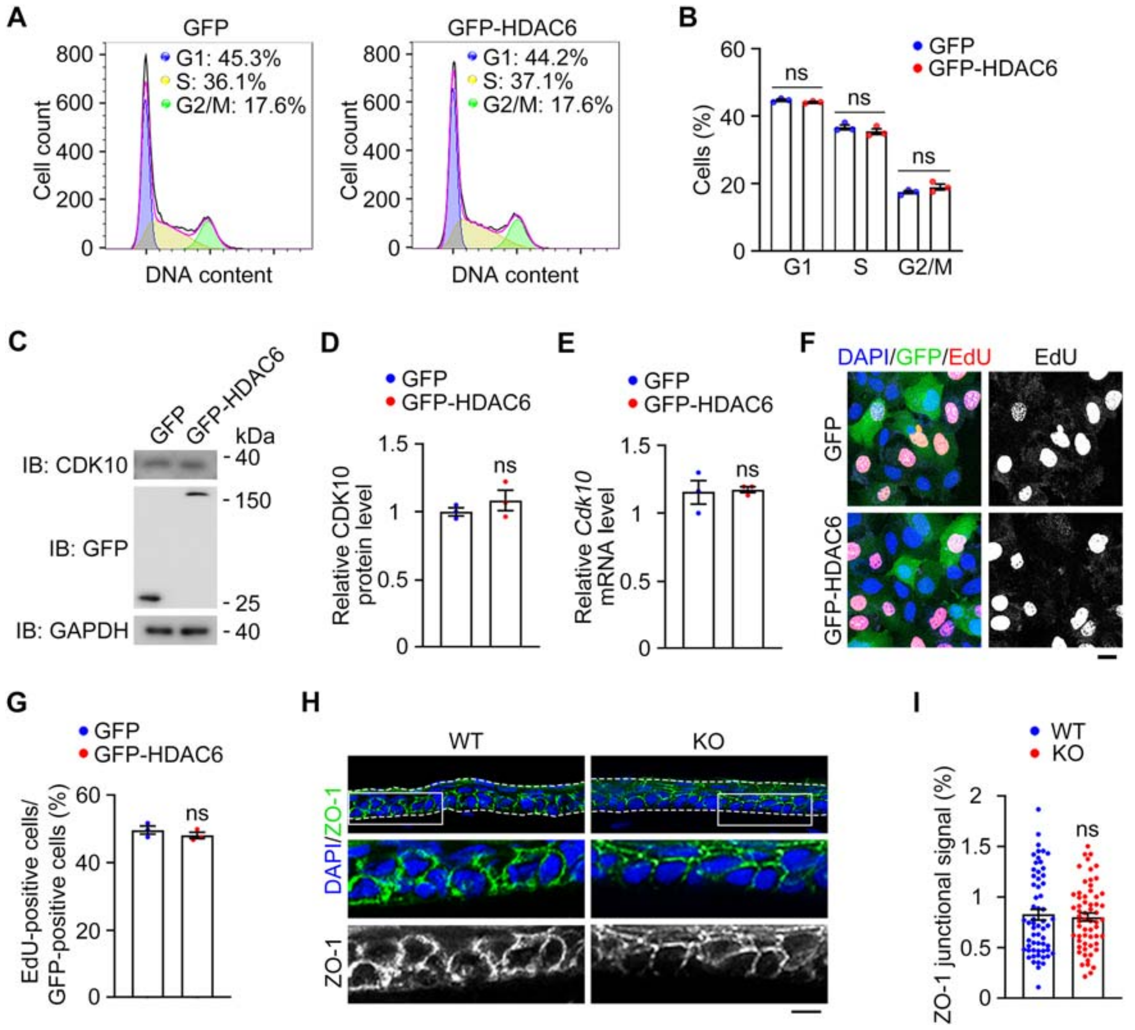

Figure EV2. HDAC6 overexpression does not affect cell cycle progression or cell proliferation.

(**A**, **B**) Flow cytometric analysis of the cell cycle (**A**) and quantification of the percentage of cells at G1, S, or G2/M phase (**B**, $n = 3$ independent experiments) for HCE-2 cells treated with GFP or GFP-HDAC6 adenoviruses. (**C**, **D**) Immunoblotting (**C**) and quantification (**D**, $n = 3$ independent experiments) showing the level of CDK10 in corneas from control or HDAC6 adenovirus-injected mice. The intensity of the CDK10 band was normalized to that of the GAPDH band. (**E**) Quantification of the *Cdk10* mRNA level in corneas from control or HDAC6 adenovirus-injected mice ($n = 3$ independent experiments). All data are normalized to the *Gapdh* mRNA level. (**F**, **G**) Immunofluorescence images (**F**) and quantification (**G**, $n = 3$ independent experiments) of the EdU-positive HCE-2 cells treated with GFP or GFP-HDAC6 adenoviruses. (**H**, **I**) Immunofluorescence images (**H**) and quantification of the ZO-1 junctional signals (**I**, $n = 60$ cells from three independent experiments) in wild-type and *Hdac6* knockout mice, stained with the ZO-1 antibody and DAPI. Scale bar, 30 µm. Data are presented as mean ± SEM. Unpaired two-tailed Student's *t* test was performed. ns not significant. Source data are available online for this figure.

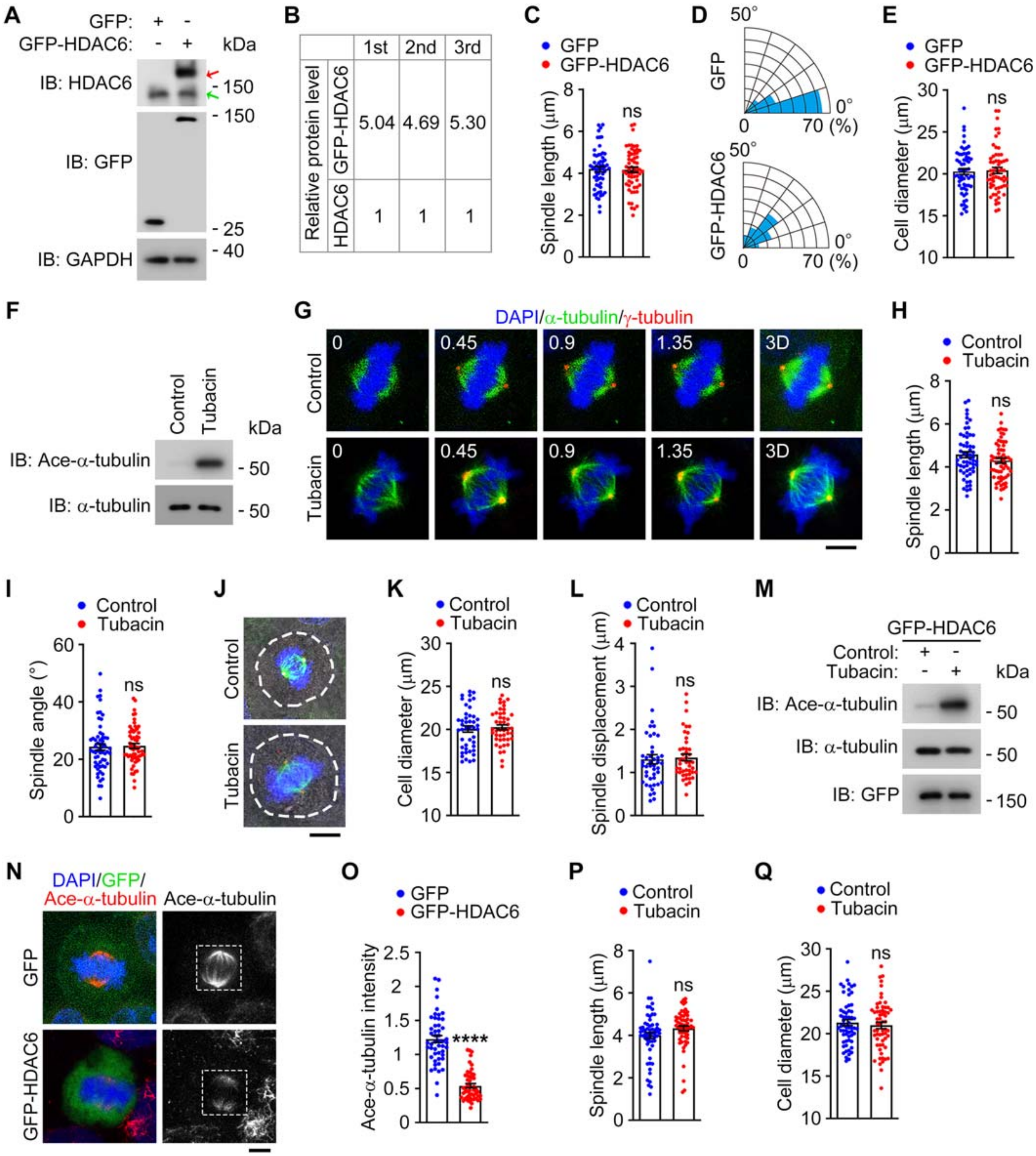
A
GFP: + -
GFP-HDAC6: - +
kDa
IB: HDAC6
- 150
- 150
IB: GFP
- 25
IB: GAPDH
- 40
B
1st 2nd 3rd
Relative protein level
GFP-HDAC6 5.04 4.69 5.30
HDAC6 1 1 1
C
GFP
GFP-HDAC6
Spindle length (μm)
ns
D
50°
GFP
0°
0 70 (%)
GFP-HDAC6
E
GFP
GFP-HDAC6
Cell diameter (μm)
ns
F
Control Tubacin
kDa
IB: Ace-α-tubulin - 50
IB: α-tubulin - 50
G
DAPI/α-tubulin/γ-tubulin
Control
Tubacin
0 0.45 0.9 1.35 3D
H
Control
Tubacin
Spindle length (μm)
ns
I
Control
Tubacin
Spindle angle (°)
ns
J
Control
Tubacin
K
Control
Tubacin
Cell diameter (μm)
ns
L
Control
Tubacin
Spindle displacement (μm)
ns
M
GFP-HDAC6
Control: + -
Tubacin: - +
kDa
IB: Ace-α-tubulin - 50
IB: α-tubulin - 50
IB: GFP - 150
N
DAPI/GFP/
Ace-α-tubulin
Ace-α-tubulin
GFP
GFP-HDAC6
O
GFP
GFP-HDAC6
Ace-α-tubulin intensity
****
P
Control
Tubacin
Spindle length (μm)
ns
Q
Control
Tubacin
Cell diameter (μm)
ns

 © The Author(s)

Figure EV3. Inhibition of HDAC6 does not affect spindle orientation or positioning.

(**A**, **B**) Immunoblot analysis of HDAC6, GFP, and GAPDH in HCE-2 cells transfected with GFP-HDAC6 or GFP. The red arrow indicates GFP-HDAC6, and the green arrow indicates endogenous HDAC6 (**A**). The relative levels of GFP-HDAC6 and endogenous HDAC6 were determined by densitometry (**B**, $n = 3$ independent experiments). (**C–E**) Quantification of spindle length (**C**, $n = 60$ images from three independent experiments), spindle angle distribution (**D**, $n = 60$ images from three independent experiments), and cell diameter (**E**, $n = 60$ images from three independent experiments) of metaphase HCE-2 cells transfected with GFP-HDAC6 or GFP. (**F**) Immunoblot analysis of acetylated α-tubulin and α-tubulin in HCE-2 cells treated with DMSO or tubacin. (**G–I**) Immunofluorescence images (**G**) and quantifications of spindle length (H, n = 60 images from three independent experiments) and spindle angle (**I**, $n = 60$ images from three independent experiments) in metaphase HCE-2 cells treated with DMSO or tubacin, and stained with antibodies against α-tubulin and γ-tubulin and DAPI. Scale bar, 4 µm. (**J–L**) Immunofluorescence/bright-field images (**J**) and quantification of cell diameter (**K**, $n = 45$ images from three independent experiments) and spindle displacement distance (**L**, $n = 45$ images from three independent experiments) in metaphase HCE-2 cells treated with DMSO or tubacin, and stained with antibodies against α-tubulin and γ-tubulin and DAPI. The white circle indicates the cell boundary (**J**). Scale bar, 6 µm. (**M**) Immunoblot analysis of acetylated α-tubulin, α-tubulin and GFP in HCE-2 cells transfected with GFP-HDAC6 and treated with DMSO or tubacin. (**N**, **O**) Immunofluorescence images (**N**) and quantification of acetylated α-tubulin intensity (**O**, $n = 50$ images from three independent experiments) of metaphase HCE-2 cells transfected with GFP-HDAC6 or GFP, and stained with antibodies against acetylated α-tubulin and DAPI. The area inside the white box was used for quantitative analysis (**N**). Scale bar, 4 µm. $P < 0.0001$. (**P**, **Q**) Quantification of spindle length (**P**, $n = 60$ images from three independent experiments) and cell diameter (**Q**, $n = 60$ images from three independent experiments) from metaphase HCE-2 cells transfected with GFP-HDAC6 and treated with tubacin (10 µM). Data are presented as mean ± SEM. Unpaired two-tailed Student's *t* test was performed. ****$P < 0.0001$; ns not significant. Source data are available online for this figure.

© The Author(s)

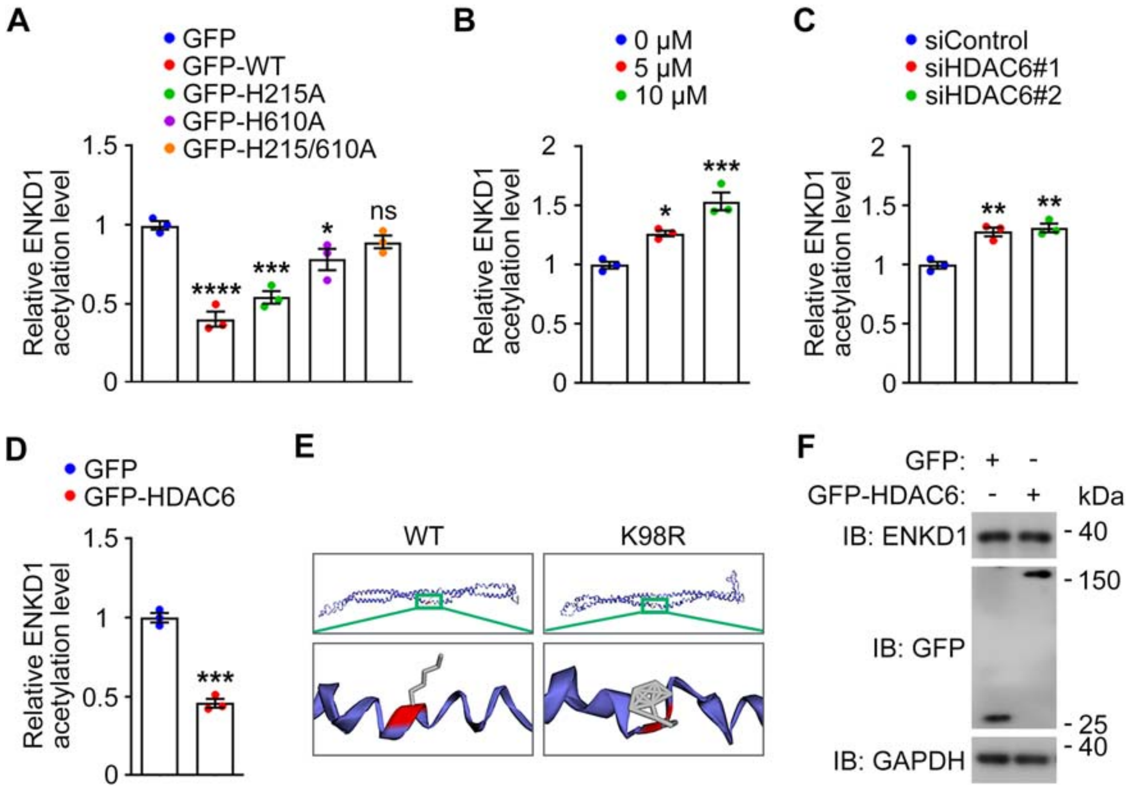

Figure EV4. HDAC6 mediates the deacetylation of ENKD1.

(**A**) Quantification of the ENKD1 acetylation level (*n* = 3 independent experiments) in HEK293T cells transfected with GFP, GFP-HDAC6 wild-type, H215A, H610A, or H215/610A, together with Flag-ENKD1. GFP-WT, $P < 0.0001$; GFP-H215A, $P = 0.0002$; GFP-H610A, $P = 0.025$; GFP-H215/610A, $P = 0.3596$. (**B**) Quantification of the ENKD1 acetylation level (*n* = 3 independent experiments) in HEK293T cells transfected with HA-HDAC6 and GFP-ENKD1, and treated with different concentrations of tubacin. 5 μM, $P = 0.0149$; 10 μM, $P = 0.0004$. (**C**) Quantification of the ENKD1 acetylation level (n = 3 independent experiments) in HEK293T cells transfected with control or HDAC6 siRNAs, together with GFP-ENKD1. siHDAC6#1, $P = 0.0021$; siHDAC6#2, $P = 0.0011$. (**D**) Quantification of the ENKD1 acetylation level (*n* = 3 independent experiments) in corneal tissues injected with control or HDAC6 adenoviruses. $P = 0.0002$. (**E**) Three-dimensional structure of ENKD1 wild-type and K98R mutant predicted with the I-TASSER software. The red color represents the K98 residue, while the gray color represents the side chain. (**F**) Immunoblot analysis of ENKD1, GFP, and GAPDH in HCE-2 cells transfected with GFP or GFP-HDAC6. Data are presented as mean ± SEM. Non-parametric one-way ANOVA with post hoc analysis was performed for (**A–C**). Unpaired two-tailed Student's *t* test was performed for (**D**). $^{*}P < 0.05$; $^{**}P < 0.01$; $^{***}P < 0.001$; $^{****}P < 0.0001$; ns not significant. Source data are available online for this figure.

© The Author(s)

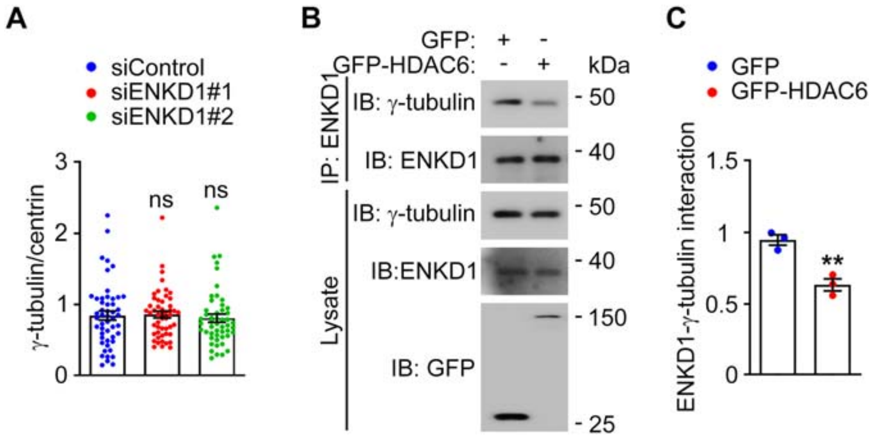

Figure EV5. HDAC6 inhibits the interaction of ENKD1 with γ-tubulin.

(**A**) Quantification of γ-tubulin intensity ($n = 50$ images from three independent experiments) in HCE-2 cells transfected with control or ENKD1 siRNAs. (**B**, **C**) Immunoprecipitation and immunoblotting (**B**) and quantification (**C**, $n = 3$ independent experiments) showing the ENKD1-γ-tubulin interaction in the corneal tissues injected with control or HDAC6 adenoviruses. The intensity of each γ-tubulin band was normalized to that of the ENKD1 band. $P = 0.0048$. Data are presented as mean ± SEM. Non-parametric one-way ANOVA with post hoc analysis was performed for (**A**). Unpaired two-tailed Student's *t* test was performed for (**C**). $^{**}P < 0.01$; ns not significant. Source data are available online for this figure.
